# Facile approach to benzo[*d*]imidazole-pyrrolo[1,2-*a*]pyrazine hybrid structures through double cyclodehydration and aromatization and their unique optical properties with blue emission†

**DOI:** 10.1039/d0ra01140a

**Published:** 2020-02-18

**Authors:** Gi Hun Bae, Suzi Kim, Na Keum Lee, Anuradha Dagar, Jeong Hwa Lee, Jeeyeon Lee, Ikyon Kim

**Affiliations:** College of Pharmacy, Yonsei Institute of Pharmaceutical Sciences, Yonsei University 85 Songdogwahak-ro, Yeonsu-gu Incheon 21983 Republic of Korea ikyonkim@yonsei.ac.kr +82 32 749 4105 +82 32 749 4515; College of Pharmacy, Research Institute of Pharmaceutical Sciences, Seoul National University 1 Gwanak-ro, Gwanak-gu Seoul 08826 Republic of Korea jyleeut@snu.ac.kr +82 2 884 8334 +82 2 880 2471

## Abstract

A modular approach to polycyclic N-fused heteroaromatics is described. Acid-catalyzed reactions of various 1-(2-oxo-2-arylethyl)-1*H*-pyrrole-2-carbaldehydes with several *o*-phenylenediamines provided facile access to a number of new benzo[*d*]imidazole-pyrrolo[1,2-*a*]pyrazine hybrid structures through double cyclodehydration and aromatization. Optical characterization of the synthesized compounds revealed unique emission properties, with deep blue emission in the aggregated and solid states, and a dramatic substituent effect was observed. Fusion of an additional benzene ring into the benzo[4,5]imidazo[1,2-*a*]pyrrolo[2,1-*c*]pyrazine scaffold resulted in a remarkable increase in the intensity of blue fluorescence from the solution along with good cell permeability and negligible phototoxicity, indicating the potential for bioimaging applications.

## Abstract 2

A modular approach to polycyclic N-fused heteroaromatics is described.

## Graphical abstract

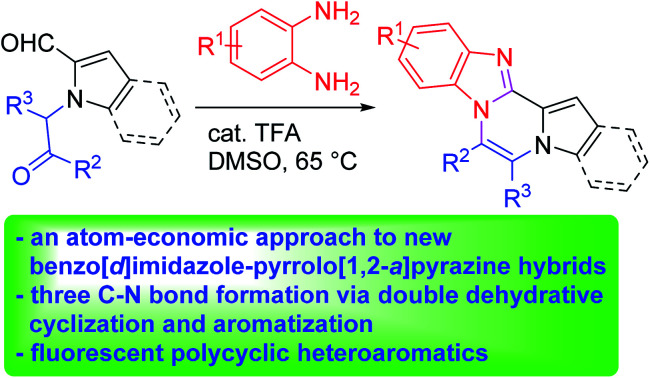

## Introduction

Novel organic fluorophores are critical tools in biomedical research and play an important role in disease diagnosis and bioimaging applications.^1–4^ Due to the detrimental aggregation-caused quenching (ACQ) effects of conventional fluorophores, various approaches to achieve stable and efficient emission have been explored.^5,6^ Along this line, aggregation-induced emission (AIE) and aggregation-induced emission enhancement (AIEE) have drawn much attention recently, as dramatic enhancements of emission in the aggregated or solid state have been observed.^7–9^ Although AIE luminogens have shown the potential to prevent quenching at high concentrations or in the solid state, practical issues remain, such as solubility and cell permeability, which need to be overcome to facilitate practical applications, particularly in various bioassays.^6^ Furthermore, the AIE effect has challenged our current understanding of the photophysical properties of photoluminescence. Therefore, novel fluorophores with unique photophysical features are of great significance in practical applications. Blue or deep-blue emissive materials are particularly valuable in organic light-emitting diodes (OLEDs), but good blue or deep-blue emitters (AIEgens) are still rare due to their wide energy gaps.^10–13^

Among the approaches reported to date, combinatorial synthesis approaches with high content imaging screening systems have shown great advantages to effectively accelerate the development of novel emissive materials, since the context of the cellular system is unpredictable in terms of the permeability, photostability and cytotoxicity of fluorophores.^14,15^

**Fig. 1:**
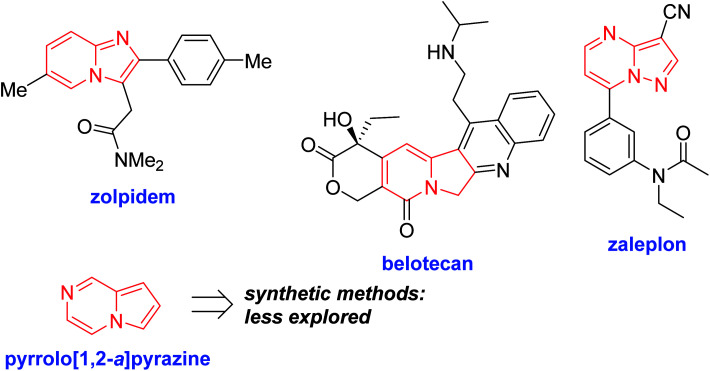
Nitrogen-fused heteroaromatics.

Nitrogen-fused heterocycles are an important structural motif frequently found in a number of natural products, pharmaceuticals, and dyes. Among them, a number of N-fused 5,6-ring systems, such as imidazo[1,2-*a*]pyridine,^16^ indolizine,^17^ and pyrazolo[1,5-*a*]pyrimidine,^18^ have received much attention, as shown in Fig. 1.

**Scheme 1:**
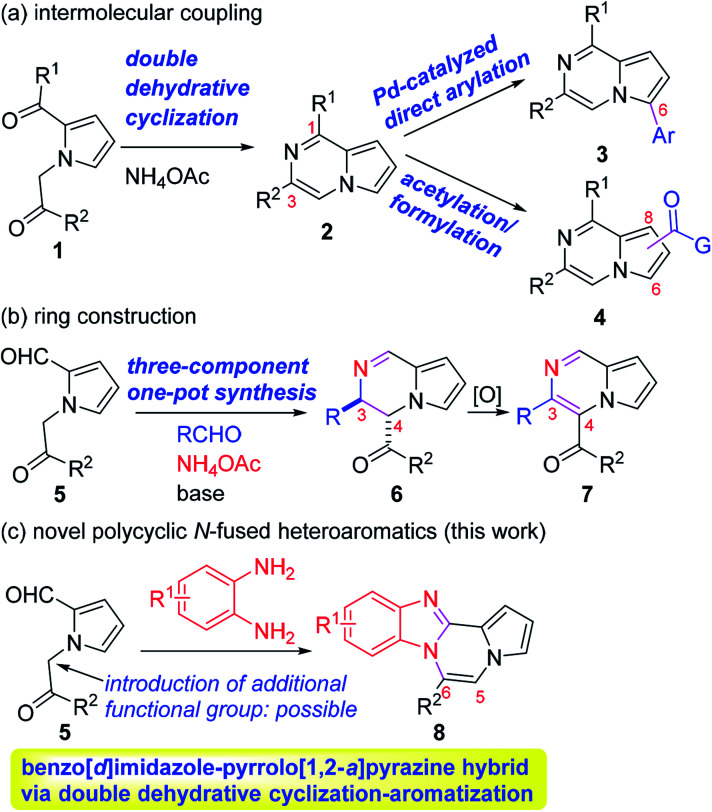
Synthetic approaches to *N*-fused heterocycles.

Accordingly, numerous synthetic methods for these scaffolds have been well established, including ring annulation and intermolecular cross-coupling.^19–22^ Although a number of medicinal agents based on pyrrolo[1,2-*a*]pyrazine, another N-fused heterobicyclic system, have been disclosed in the literature, synthetic approaches to prepare this scaffold have been relatively underdeveloped, which prompted us to investigate the chemistry of pyrrolo[1,2-*a*]pyrazine. In this context, we have been involved in the design and synthesis of new chemical spaces based on the pyrrolo[1,2-*a*]pyrazine core (Scheme 1). As a means to decorate the basic skeleton *via* intermolecular cross-coupling reactions, we have studied Pd-catalyzed direct (hetero)arylation^23,24^ and electrophilic acetylation/formylation^25,26^ of various pyrrolo[1,2-*a*]pyrazines (2), which were easily prepared by double cyclodehydration of 1 in the presence of NH_4_OAc (Scheme 1a). More recently, we have developed atom-economical synthetic routes to prepare novel pyrrolo[1,2-*a*]pyrazine derivatives (6–7) from 5*via* tandem ring cyclization sequences (Scheme 1b).^27^ Ammonium acetate was used as an amine source for 2, 6, and 7. As an extension, we hypothesized that *o*-phenylenediamine might be employed in the reaction with 5 as an alternative amine source, leading to pyrrolo[1,2-*a*]pyrazine-fused polycyclic heteroaromatic compound 8 (Scheme 1c). There is only one literature example, by Nagarajan and coworkers, on the synthesis of this heterocyclic core in which *N*-propargyl-substituted pyrrole-2-carboxaldehydes were used under Cu catalysis.^28^ While this method gave rise to the desired products with a methyl group at the C6 position, reactions with unsymmetrical *o*-phenylenediamines resulted in a mixture of regioisomers. Moreover, regioisomeric mixtures were obtained with arylpropargyl-substituted substrates as a result of incomplete isomerization. In contrast, our strategy could lead to the target skeleton with not only methyl but also various aryl moieties at the C6 site, demonstrating versatility of our method. Since an additional functional group can be introduced to the α position of the ketone of our substrate 5, we deemed that our approach to this skeleton would be more flexible, affording a wide variety of derivatives with substituent(s) at the C5 and/or C6 site(s). Mechanistically, dehydrative condensation of *o*-phenylenediamine with the aldehyde of 5 would form dihydrobenzo[*d*]imidazole, which would further react with other carbonyl moieties in 5 to give rise to tetracyclic compound 8 after air oxidation. Overall, we anticipated that a polycyclic heteroaromatic ring system would be constructed by a one-pot domino protocol consisting of double cyclodehydration and aromatization.

Furthermore, as we expected that the tetracyclic skeleton formed as a hybrid structure of benzo[*d*]imidazole and pyrrolo[1,2-*a*]pyrazine,^29–32^ two versatile pharmacophores in medicinal chemistry, might exhibit intriguing photophysical properties, we decided to embark on the synthesis and optical characterization of this scaffold, which is described herein.

## Results and discussion

### Design and synthesis

The reaction was optimized with 5a^33^ and *o*-phenylenediamine (Table 1). Reactions in AcOH or AcOH/toluene (1 : 1) at 90 °C gave 8a in 54–55% yields (entries 1 and 2). While the use of DBSA (dodecylbenzenesulfonic acid) or PTSA as a catalyst improved the yield of 8a (entries 3–5), reactions in the presence of a Lewis acid furnished the product at a yield of 32–55% (entries 6–8). Finally, we were pleased to find that desired product 8a was obtained in 80% yield by initial treatment of 5a and *o*-phenylenediamine in TFA/DMSO at room temperature for 8 h followed by warming at 65 °C for an additional 3 h (entry 9).^34^ The reaction in a closed vial gave similar results as that in open air. Intermediate I was identified at room temperature, and subsequent heating facilitated dehydrative cyclization to furnish 8a.

**Table 1:** Reaction optimization^a^

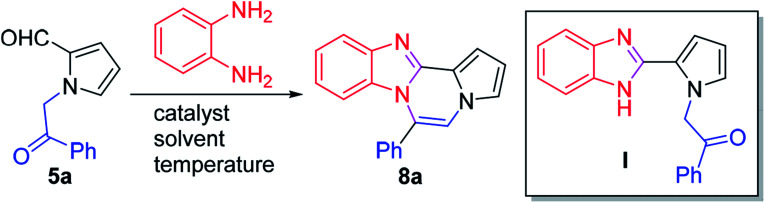				
Entry	Catalyst (equiv.)	Solvent	Temperature (°C)	Yield^b^ (%)
1	—	AcOH	90	54
2	—	AcOH/toluene (1 : 1)	90	55
3	DBSA (0.1)	DMF	150	60
4	DBSA (0.1)	Toluene	130	63
5	PTSA (0.1)	Toluene	130	62
6	In(OTf)_3_ (0.1)	DCE	90	47
7	Sc(OTf)_3_ (0.1)	DCE	90	32
8	Bi(OTf)_3_ (0.1)	DCE	90	55
9^c^	TFA (0.3)	DMSO	rt to 65	80

a A mixture of 5a (0.188 mmol) and *o*-phenylenediamine (0.244 mmol, 1.3 equiv.) in solvent (1 mL) was stirred at the indicated temperature for 12 h. The reaction mixture was stirred at rt for 16 h.

b Isolated yield (%).

c After a mixture of 5 (0.188 mmol) and *o*-phenylenediamine (0.244 mmol, 1.3 equiv.) in TFA (0.056 mmol, 0.3 equiv.)/DMSO (1 mL) was stirred at rt for 8 h, the reaction mixture was stirred at 65 °C for an additional 3 h.

As shown in Table 2, reactions of 5a with several *o*-phenylenediamines were performed under optimized conditions to furnish the corresponding 6-arylbenzo[4,5]imidazo[1,2-*a*]pyrrolo[2,1-*c*]pyrazines (8a–8g) in good to excellent yields. Notably, the use of unsymmetrical *o*-phenylenediamines such as 4-fluoro-*o*-phenylenediamine and 4-chloro-*o*-phenylenediamine under these conditions gave rise to regioisomers 8c and 8e, respectively. The structure of 8c was unambiguously confirmed by X-ray crystallographic analysis (CCDC number: 1919367, Fig. 2). 6-Phenylnaphtho[2′,3′:4,5]imidazo[1,2-*a*]pyrrolo[2,1-*c*]pyrazine (8h) was obtained from the reaction with 2,3-diaminonaphthalene.

**Table 2:** Synthesis of 8a–8h^a^^,^^b^

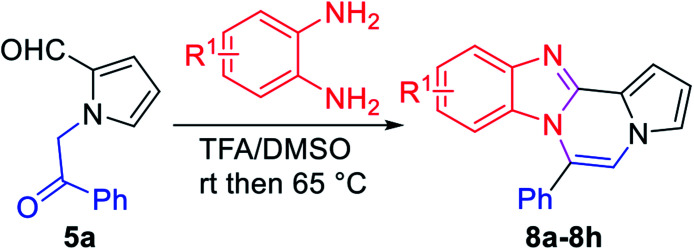		
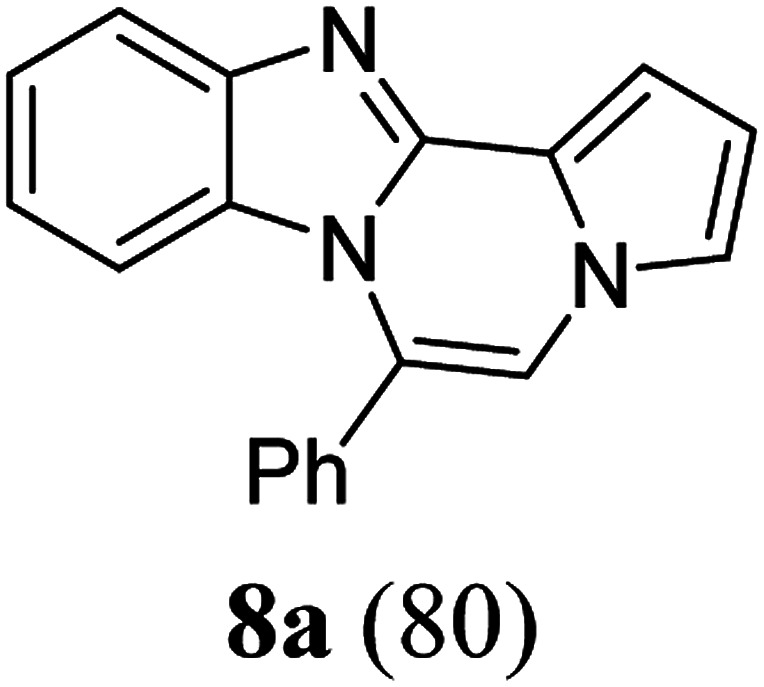	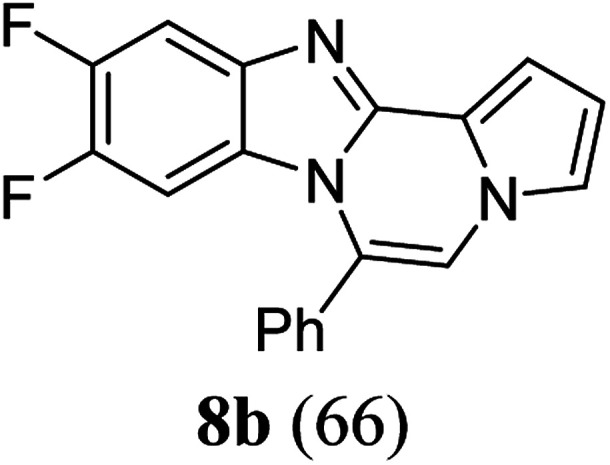	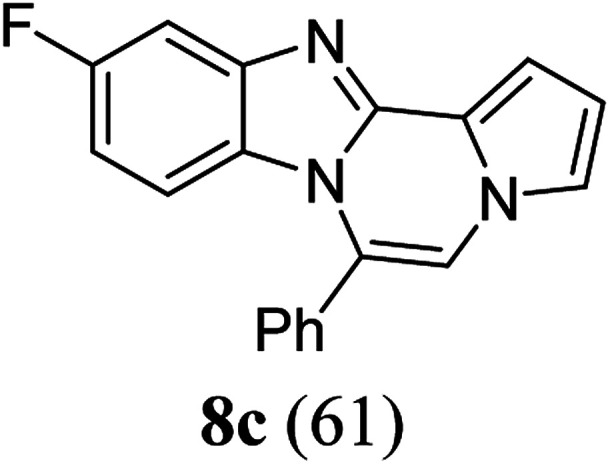
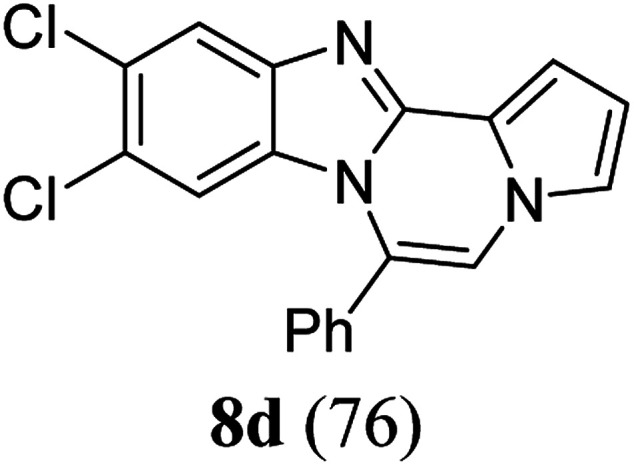	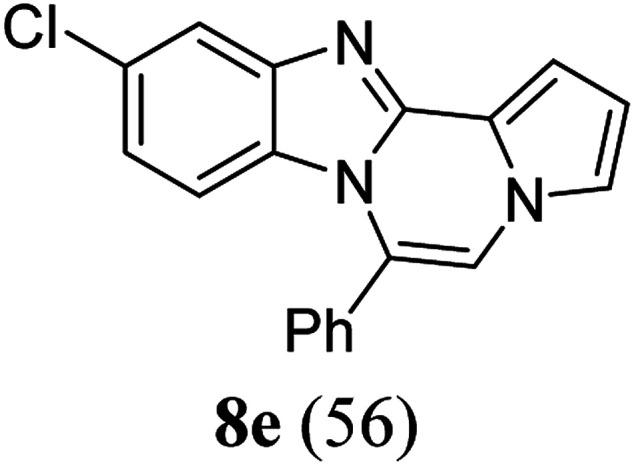	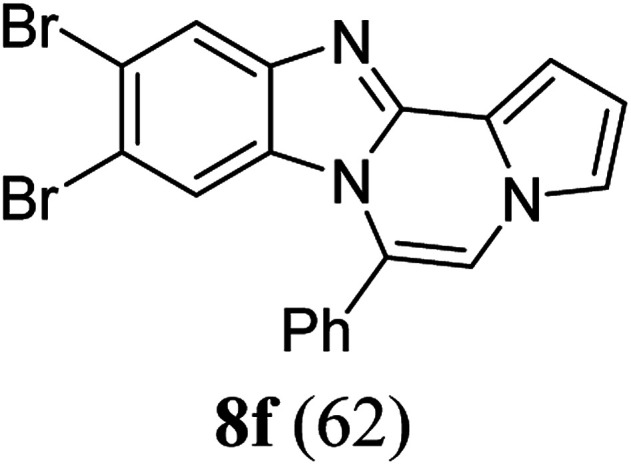
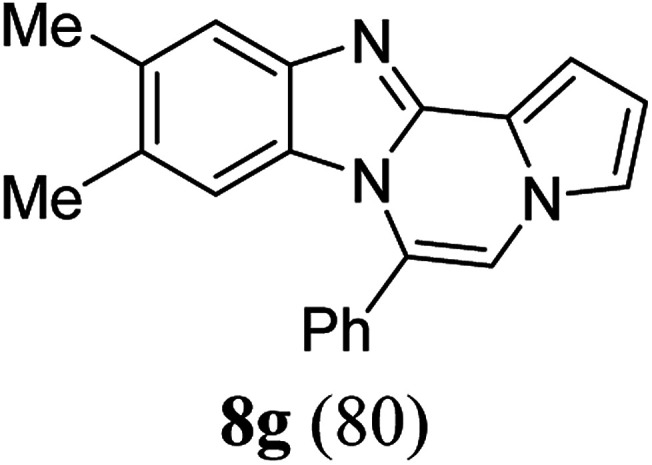	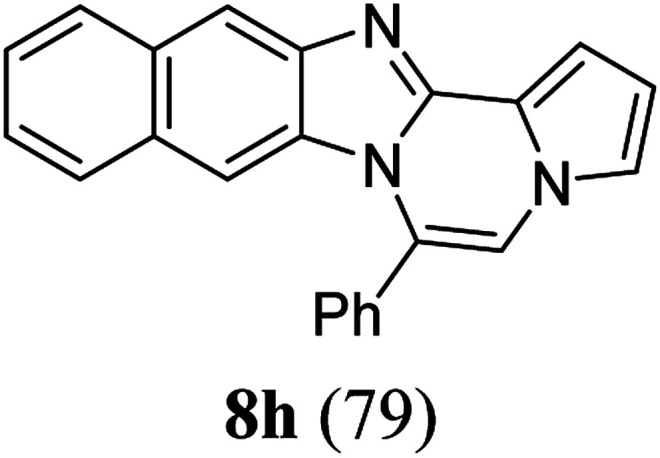	

a After a mixture of 5a (0.188 mmol) and *o*-phenylenediamine (0.244 mmol, 1.3 equiv.) in TFA (0.056 mmol, 0.3 equiv.)/DMSO (1 mL) was stirred at rt for 8 h, the reaction mixture was stirred at 65 °C for an additional 3 h.

b Isolated yield (%).

**Fig. 2:**
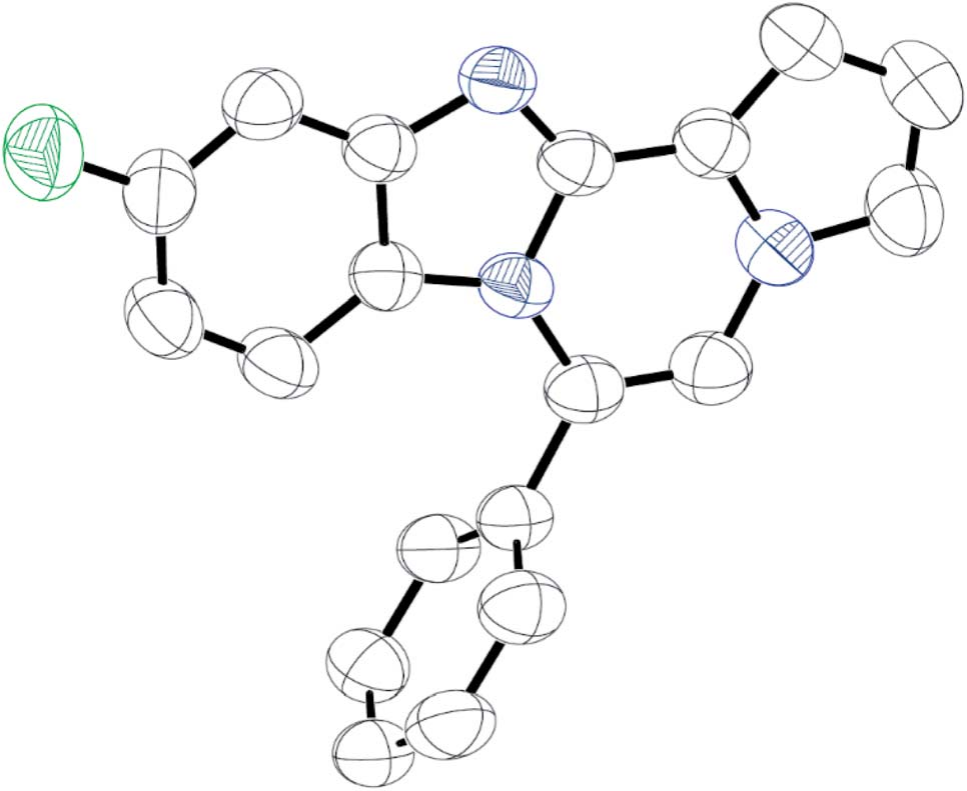
Crystal structure of 8c.

More diverse benzo[*d*]imidazole-pyrrolo[1,2-*a*]pyrazine hybrids (8i–8ai) were synthesized with 5b–i. The results are shown in Table 3, exhibiting a wide range of functional group tolerances under these reaction conditions. DBSA/toluene was used in some cases where TFA/DMSO afforded low chemical yields (8n, 8p, 8v, 8x, and 8y).

**Table 3:** Synthesis of 8i–8ai^a^

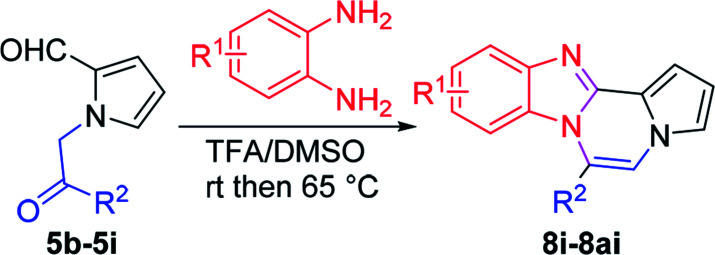			
5	8		Yield^b^ (%)
5b (R^2^ = 4-FC_6_H_4_)	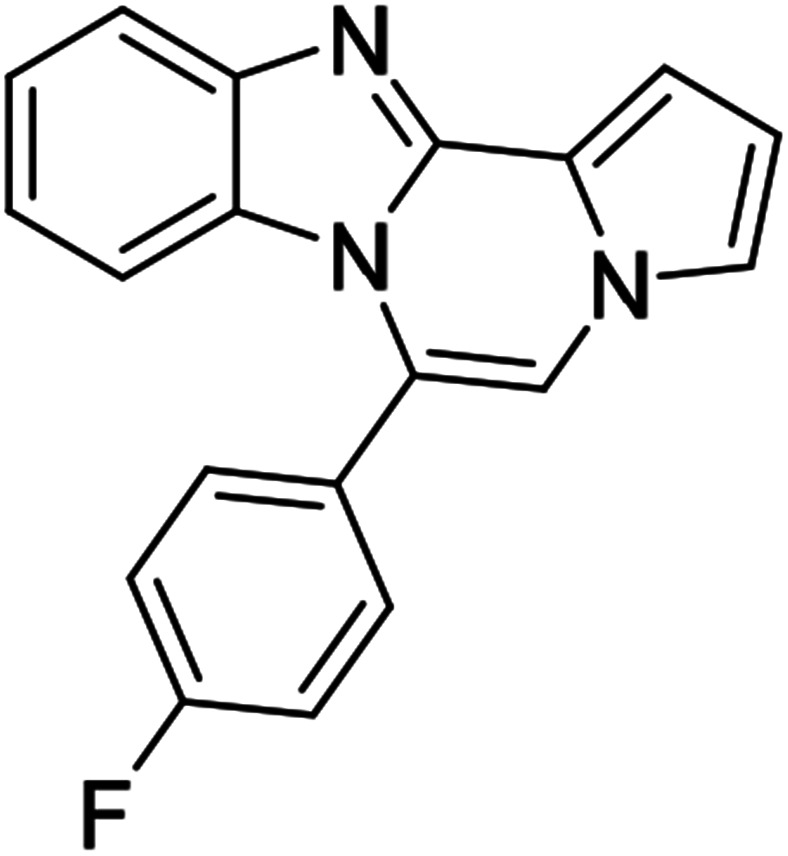	8i	65
5b	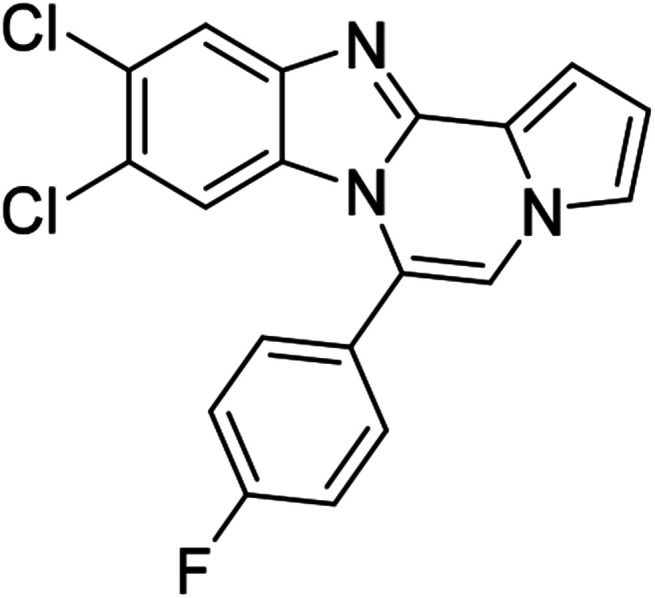	8j	45
5b	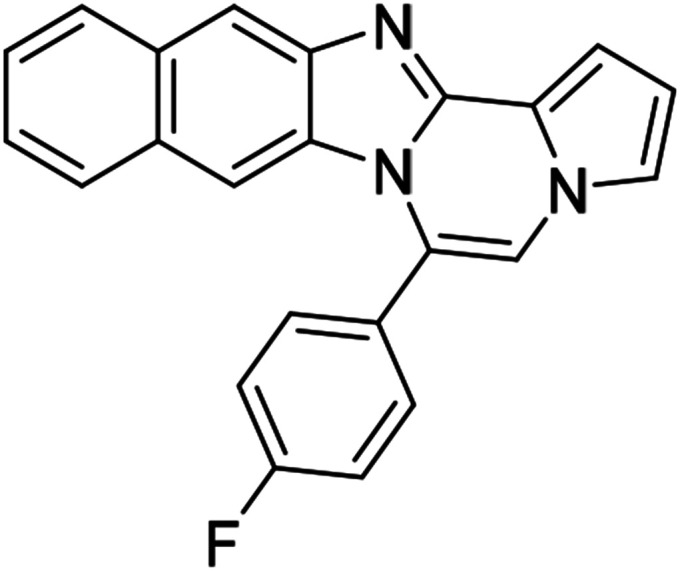	8k	87
5c (R^2^ = 4-ClC_6_H_4_)	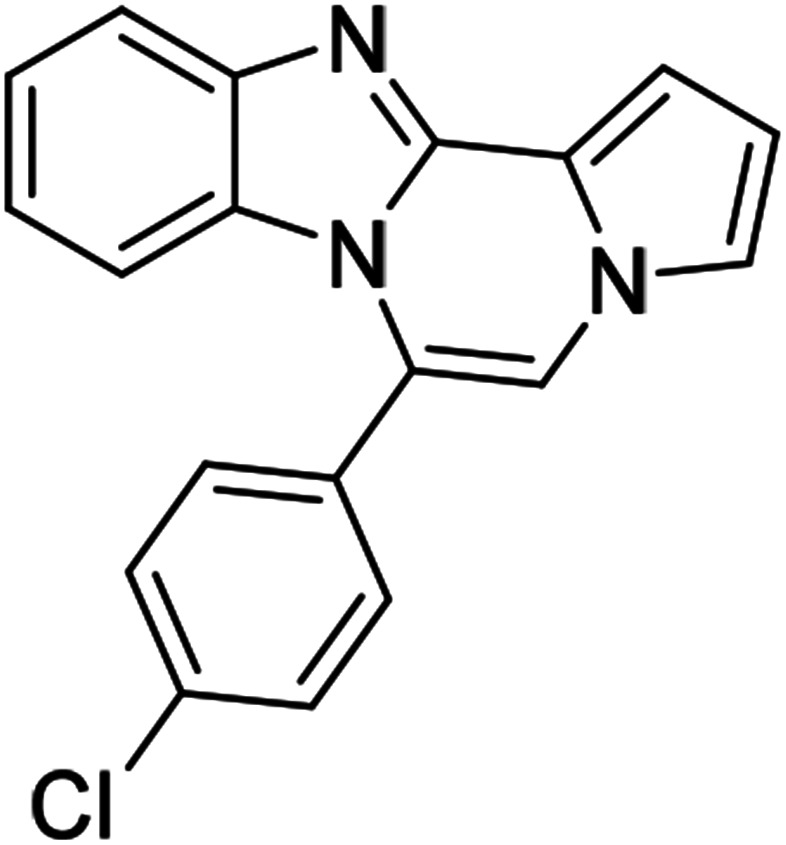	8m	86
5c	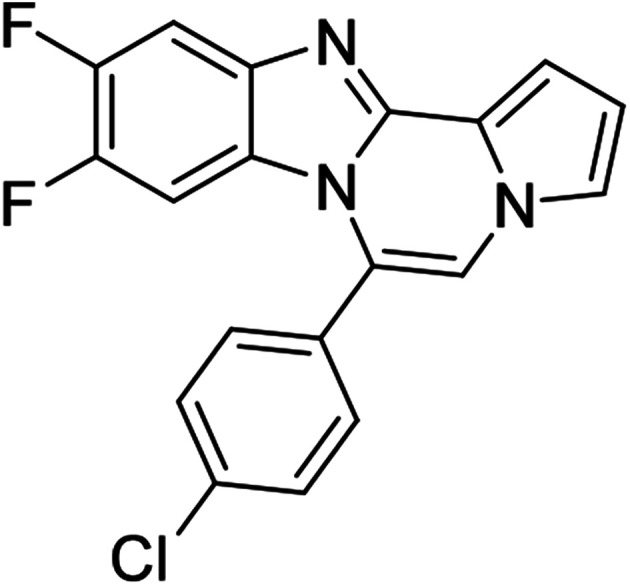	8n	70^c^
5c	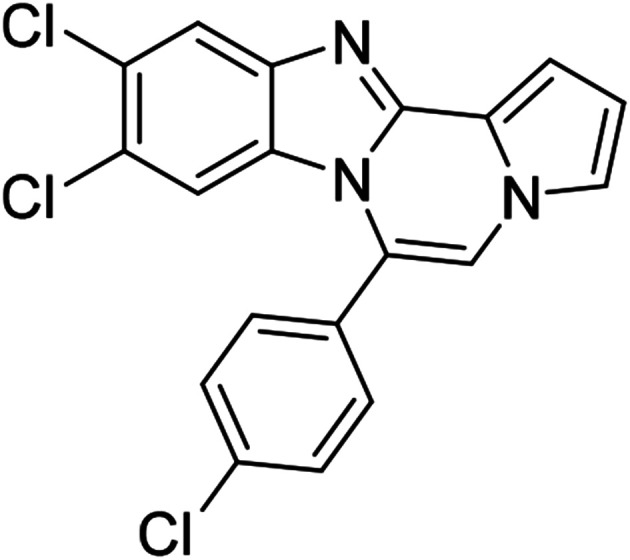	8o	81
5c	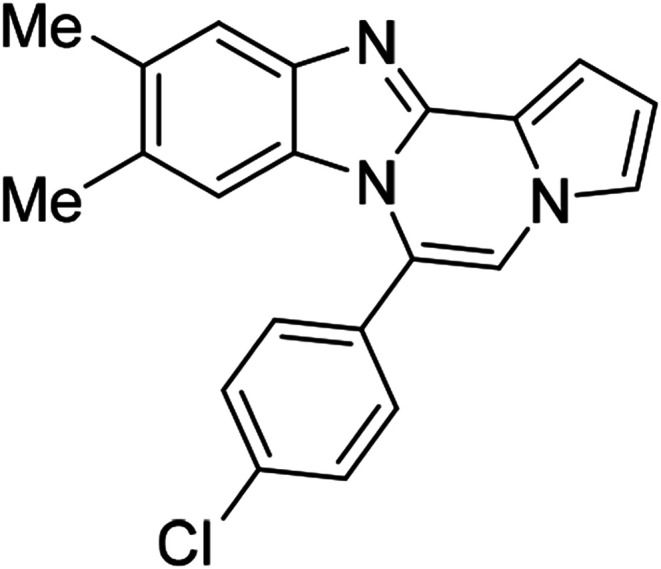	8p	63^c^
5c	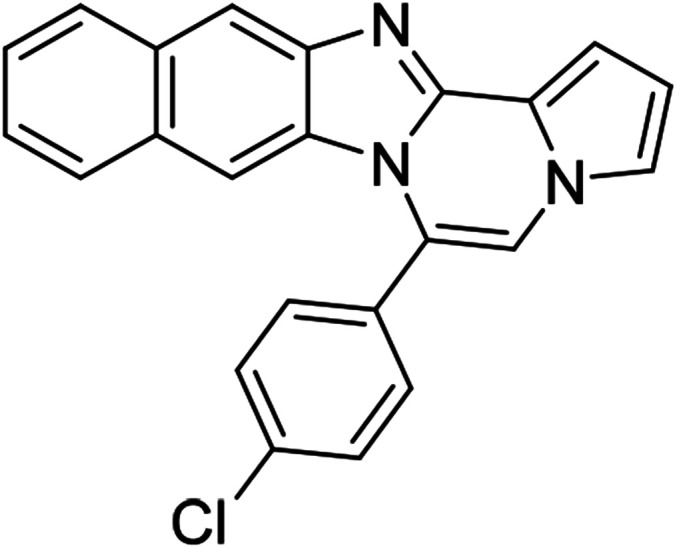	8q	72
5d (R^2^ = 4-BrC_6_H_4_)	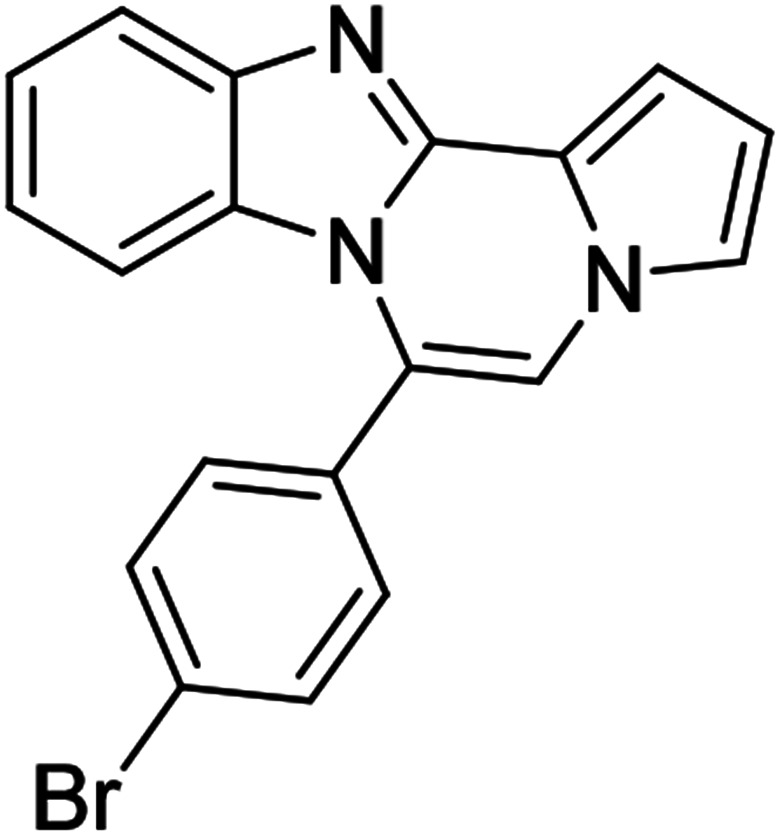	8r	68
5d	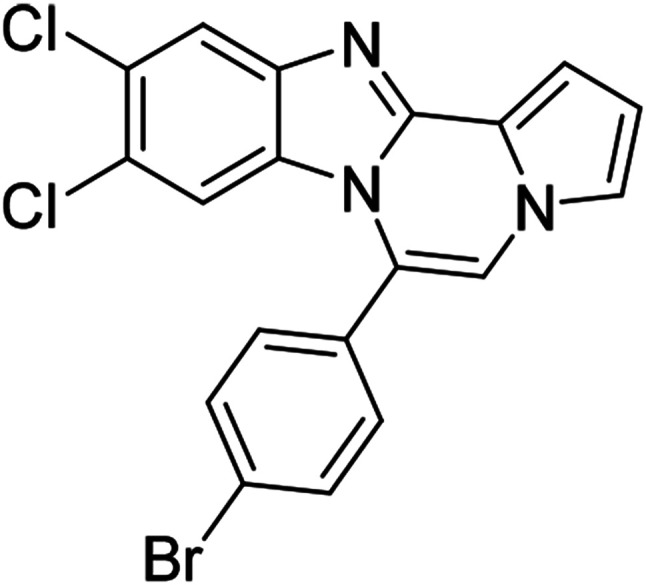	8s	78
5d	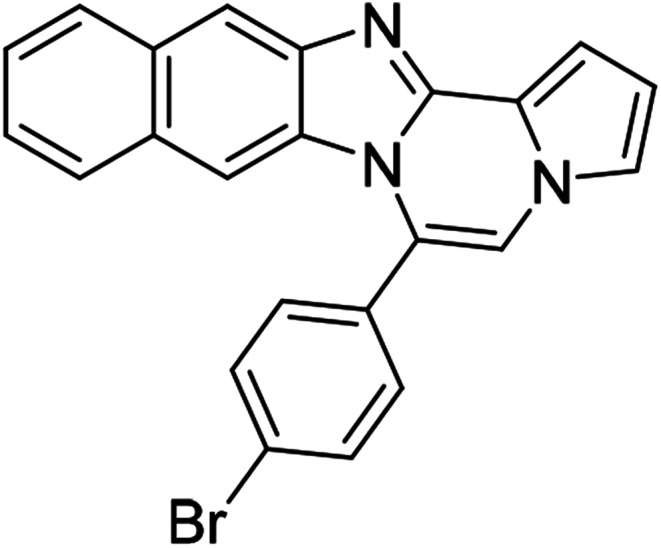	8t	84
5e (R^2^ = 4-MeOC_6_H_4_)	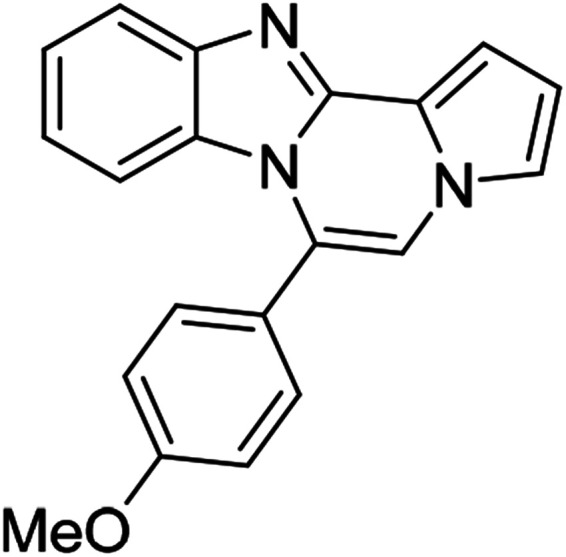	8u	73
5e	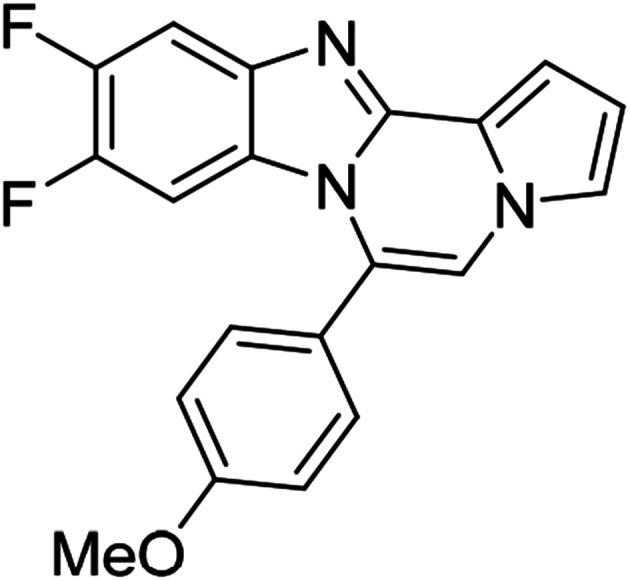	8v	54^c^
5e	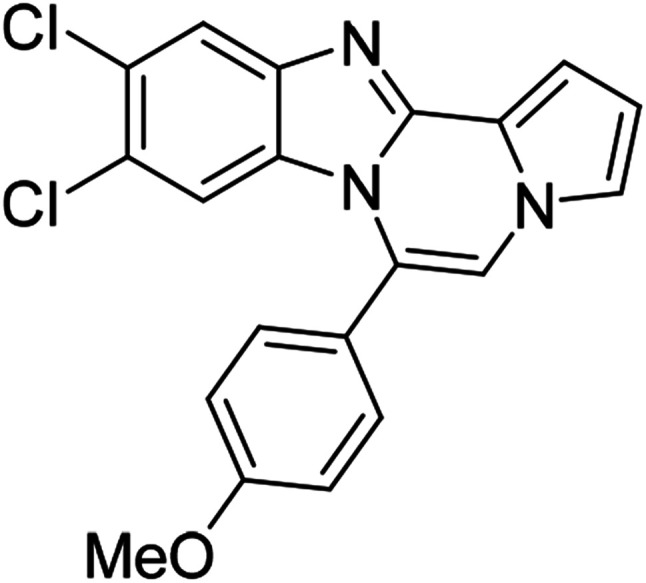	8w	91
5e	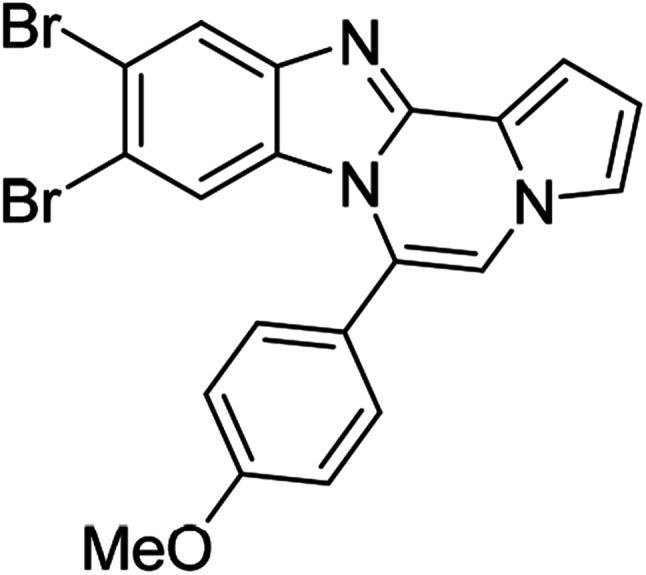	8x	51^c^
5e	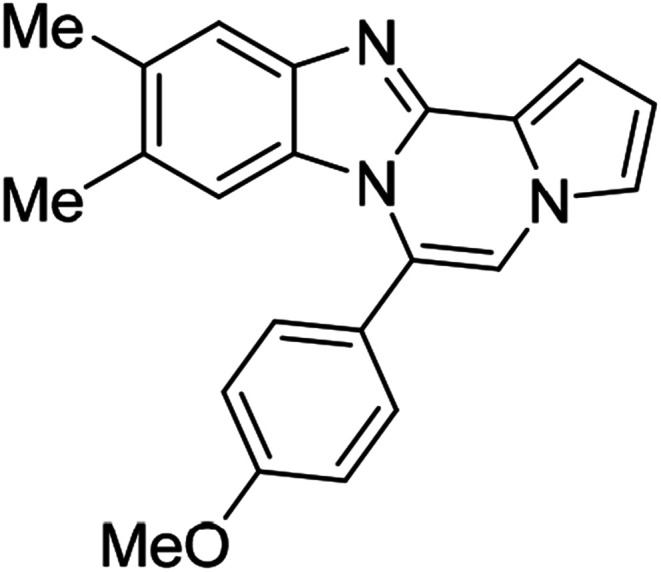	8y	63^c^
5e	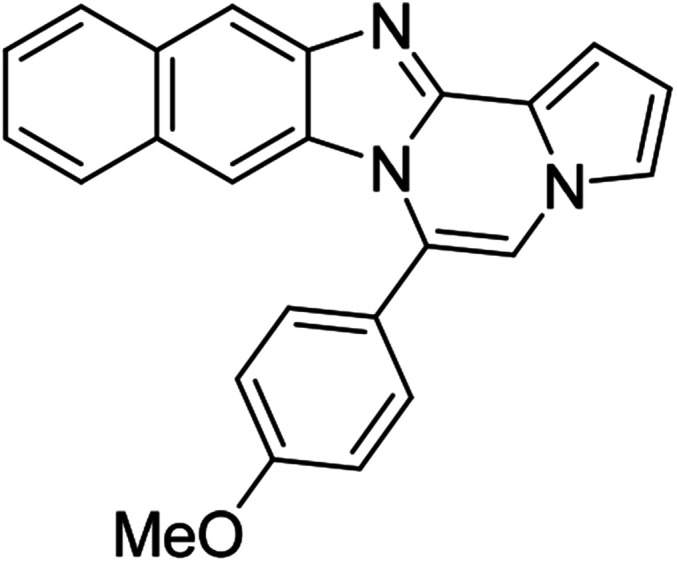	8z	81
5f (R^2^ = 2,5-(MeO)_2_C_6_H_3_)	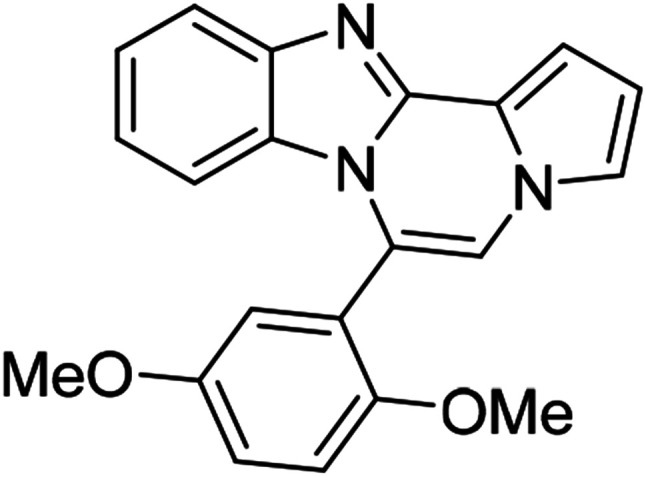	8aa	78
5f	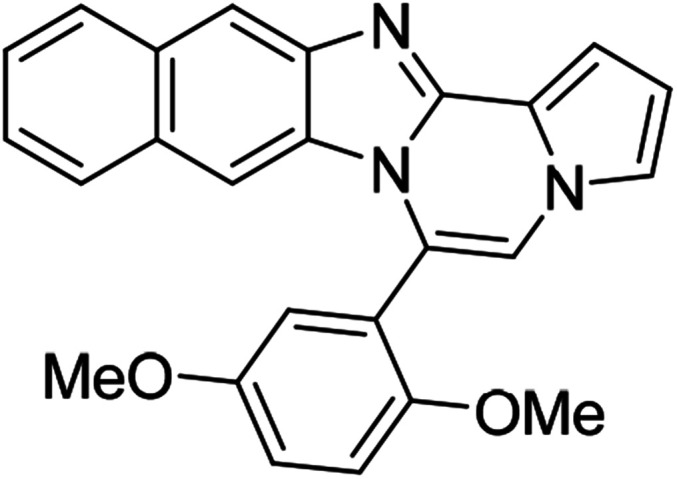	8ab	81
5g (R^2^ = 4-PhC_6_H_4_)	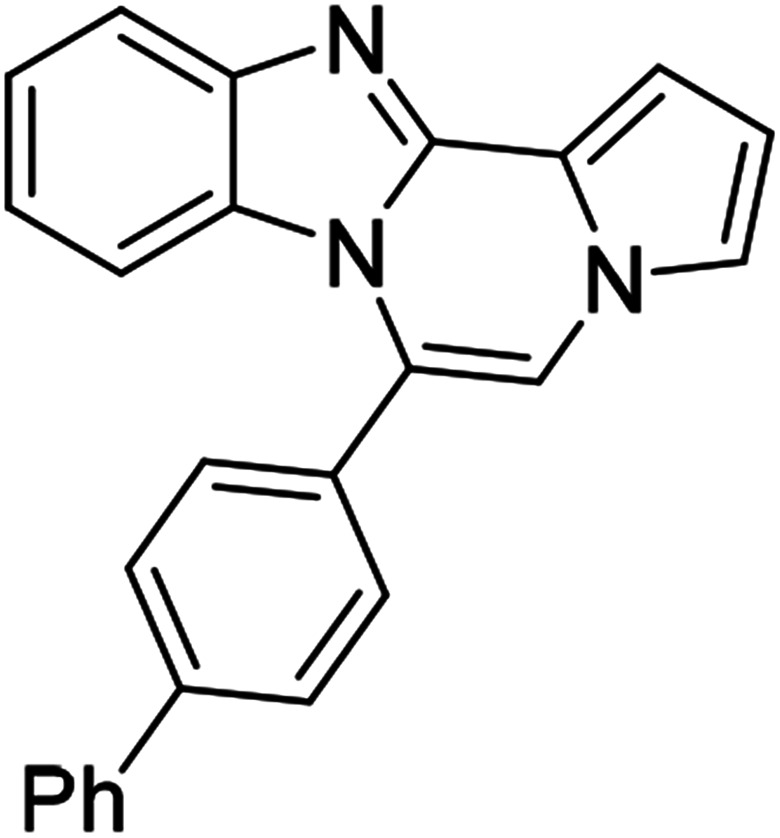	8ac	85
5g	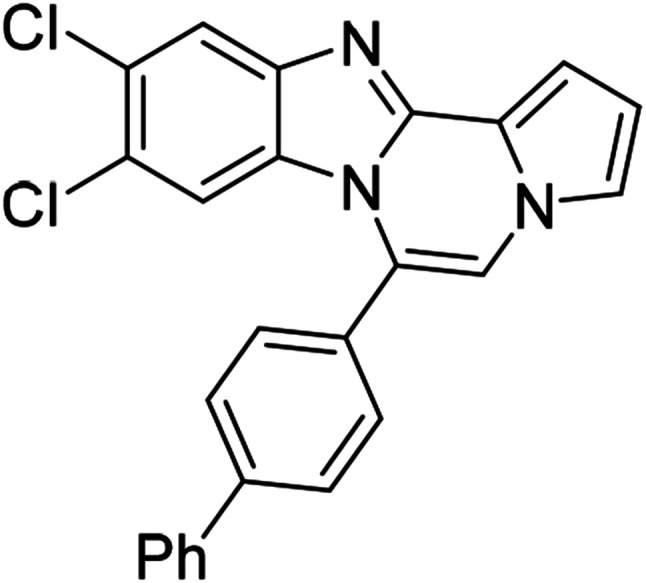	8ad	58
5g	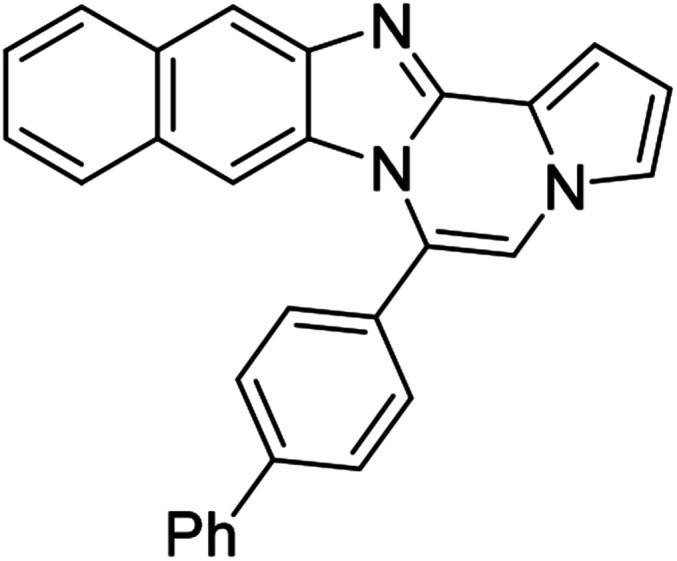	8ae	63
5h (R^2^ = 2-naphthalene)	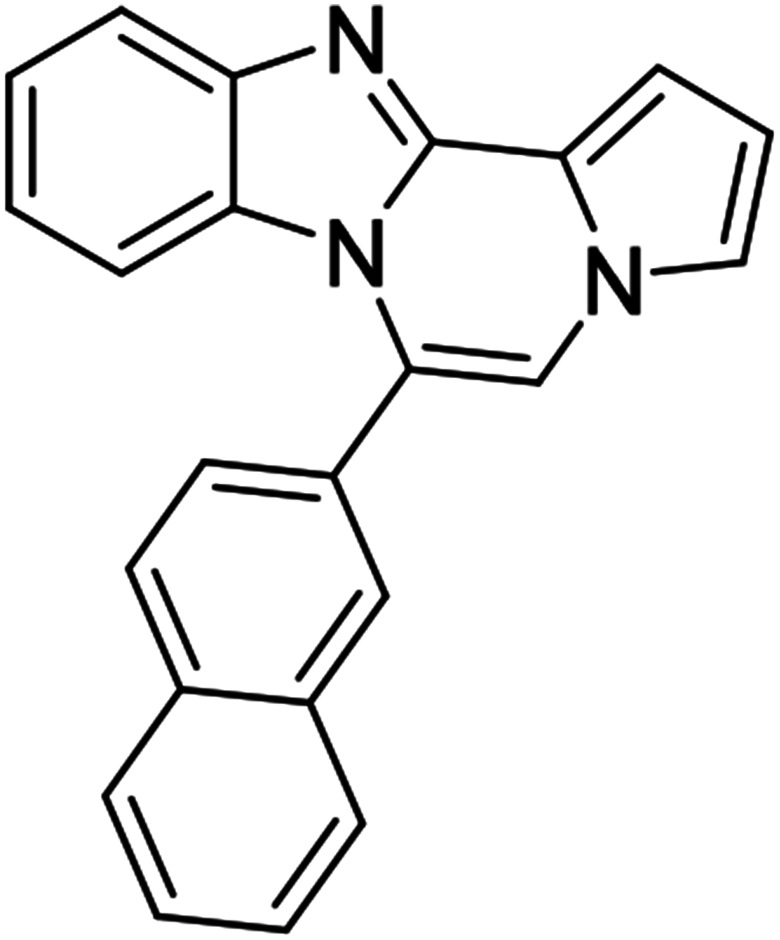	8af	75
5h	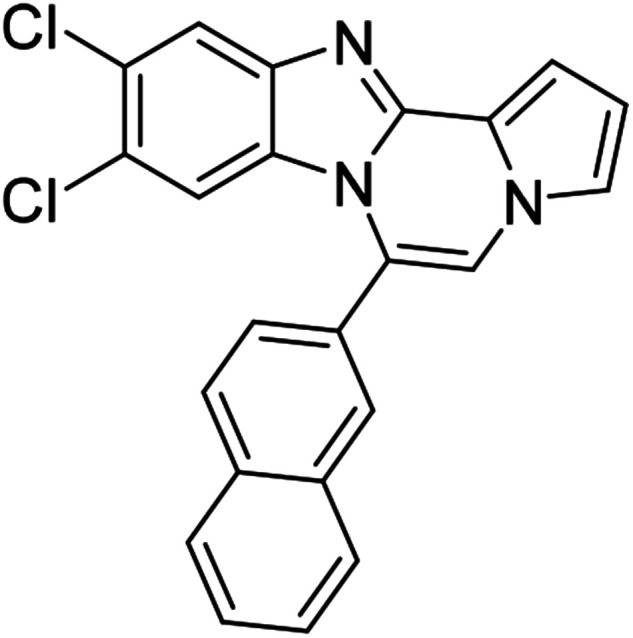	8ag	57
5h	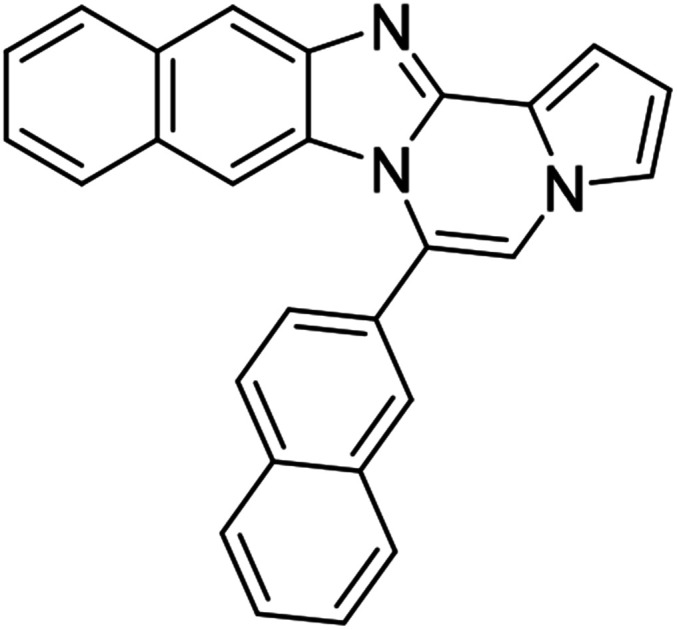	8ah	79
5i (R^2^ = Me)	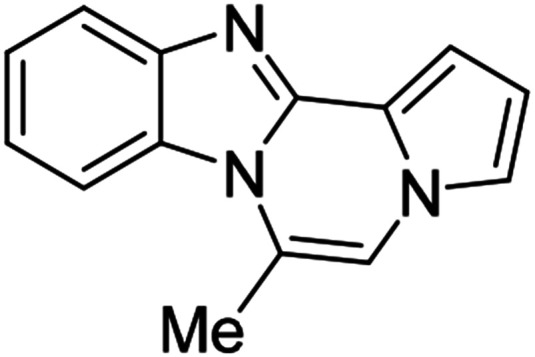	8ai	80

a After a mixture of 5 (0.188 mmol) and *o*-phenylenediamine (0.244 mmol, 1.3 equiv.) in TFA (0.056 mmol, 0.3 equiv.)/DMSO (1 mL) was stirred at rt for 8 h, the reaction mixture was stirred at 65 °C for an additional 3 h.

b Isolated yield (%).

c After a mixture of 5 (0.188 mmol), *o*-phenylenediamine (0.244 mmol, 1.3 equiv.), and DBSA (0.0188 mmol, 0.1 equiv.) in toluene (1 mL) was stirred at 130 °C for 12 h.

**Scheme 2:**
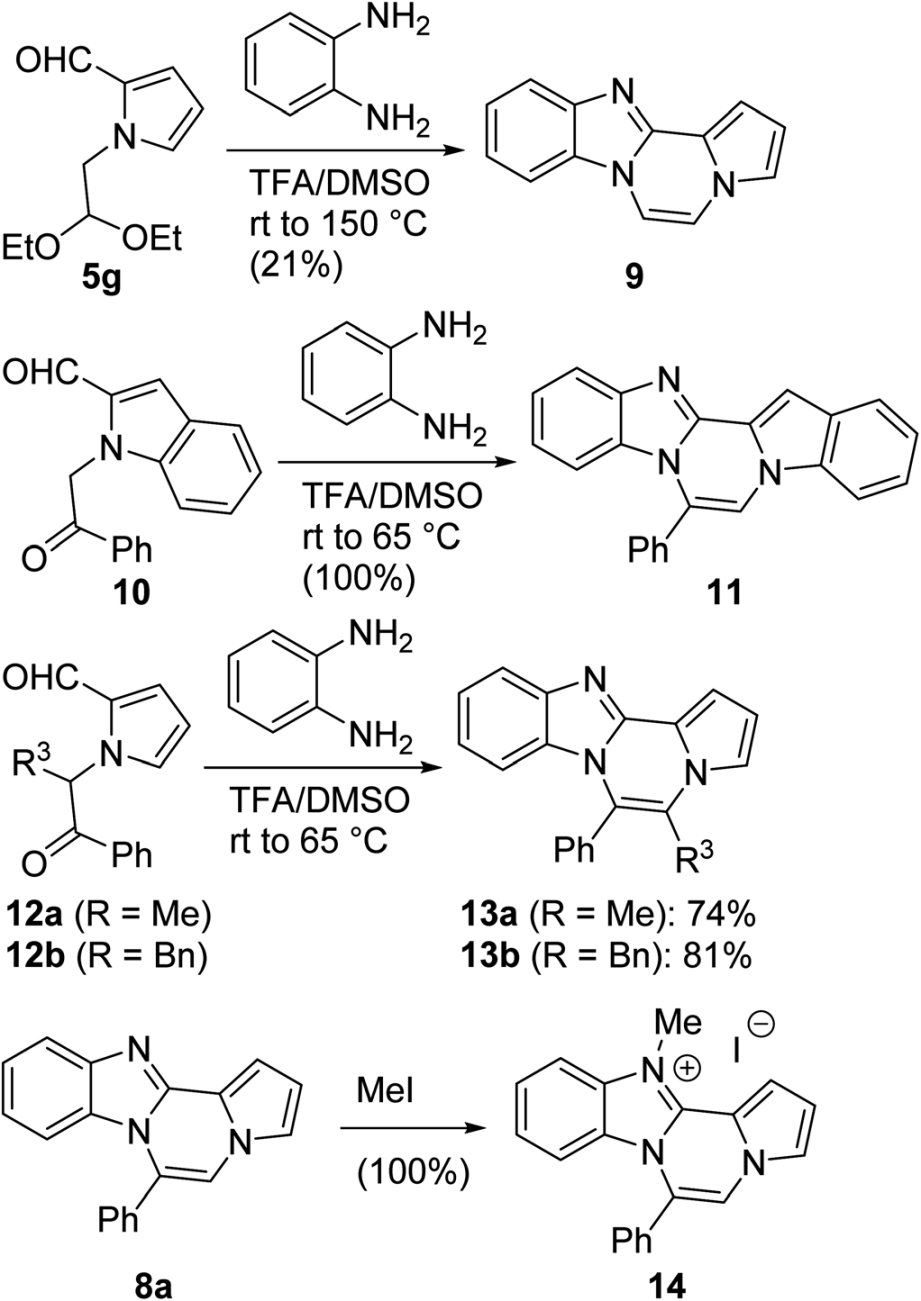
Synthesis of 9, 11, 13, and 14.

The basic tetracyclic hybrid skeleton 9 was also obtained, although conversion of 5g to 9 required a higher reaction temperature (Scheme 2). When 10, derived from indole-2-carbaldehyde, was allowed to react with *o*-phenylenediamine, the corresponding hexacyclic 11 was obtained in quantitative yield. As noted in the Introduction, benzo[*d*]imidazole-pyrrolo[1,2-*a*]pyrazines 13a–b bearing two substituents at the C5 and C6 sites were constructed upon exposure of 12a–b to the optimal reaction conditions, demonstrating the benefits of our protocol over the previous approach. In addition, further elaboration of the resulting 8a was conducted: *N*-alkylation of 8a with methyl iodide delivered the corresponding salt 14 in quantitative yield.

### Optical properties and structure–property relationship analysis

**Fig. 3:**
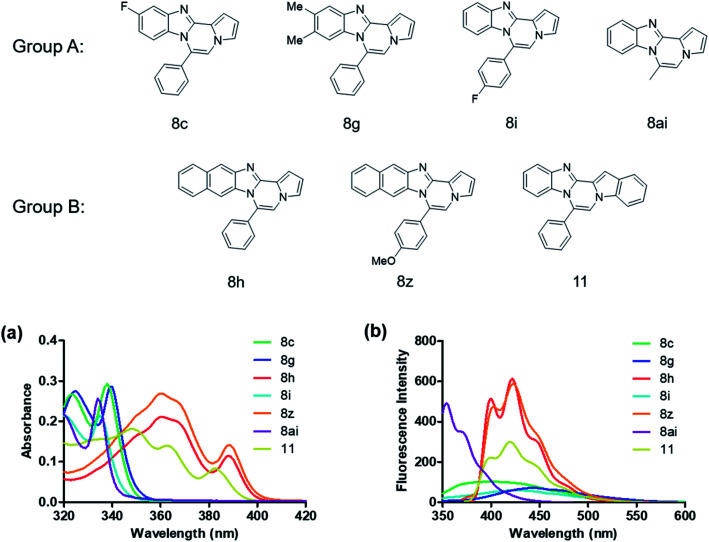
Absorption spectra (a) of 10 μM and emission spectra (b) of 1 μM 8c, 8g, 8h, 8i, 8z, 8ai, and 11 measured in DMSO.

We examined the optical properties of the synthesized compounds, as shown in Fig. 3 and Table S1.† The compounds exhibited absorption maxima at approximately 320–360 nm and emission maxima at 400–450 nm in DMSO. Based on the optical properties, the compounds were categorized into two groups. Group A compounds possess a benzo[4,5]imidazo[1,2-*a*]pyrrolo[2,1-*c*]pyrazine scaffold (named 4BP), whereas Group B compounds include 6-phenylnaphtho[2′,3′:4,5]imidazo[1,2-*a*]pyrrolo[2,1-*c*]pyrazines and 7-phenylbenzo[4′,5′]imidazo[2′,1′:3,4]pyrazino[1,2-*a*]indoles (named 5BP). Representative compounds are shown in Fig. 3 along with their absorption and emission spectra. In Group A, pyrrolo[1,2-*a*]pyrazines without an aromatic ring at the C6 position showed strong blue emission in solution (8ai), but they were not emissive in the solid state due to the ACQ effect. The addition of benzene or naphthalene groups at the C6 position decreased the emission intensity, but the emission maxima were red-shifted (Table S1†).

Further structure–property relationship analysis revealed that the fluorescence intensity and emission maxima were significantly affected by attaching different substituents to the R^1^ and R^2^ positions of the benzo[4,5]imidazo[1,2-*a*]pyrrolo[2,1-*c*]pyrazine scaffold (Fig. S1a†). When attaching an electron-withdrawing halide to the R^1^ position (8d, Fig. S1b†), the fluorescence intensity increased, and a hypsochromic shift was observed relative to 8a (Fig. S1a†). However, by attaching an electron-donating –CH_3_ group to R^1^, as shown for 8g (Fig. S1c†), the opposite effect occurred, that is, a bathochromic shift. 8m with 4-ClC_6_H_4_ group at the C6 position (Fig. S1d†) exhibited a slight bathochromic shift (∼10 nm) compared to 8a, while 8u with 4-MeOC_6_H_4_ group at R^2^ (Fig. S1e†) showed blue-shifted emission with increased intensity. Overall, the electronic effect from the electron-withdrawing R^1^ and the electron-donating substituent on the phenyl ring of R^2^ was found to be important for enhancing the fluorescence intensity in Group A.

Extension with an additional benzene ring in Group B, which includes 6-phenylnaphtho[2′,3′:4,5]imidazo[1,2-*a*]pyrrolo[2,1-*c*]pyrazines (8h) and 7-phenylbenzo[4′,5′]imidazo[2′,1′:3,4]pyrazino[1,2-*a*]indole (11, Fig. S2†), remarkably enhanced the fluorescence intensity compared to that from Group A.

### Aggregation induced blue-shifted emission from Group A

**Fig. 4:**
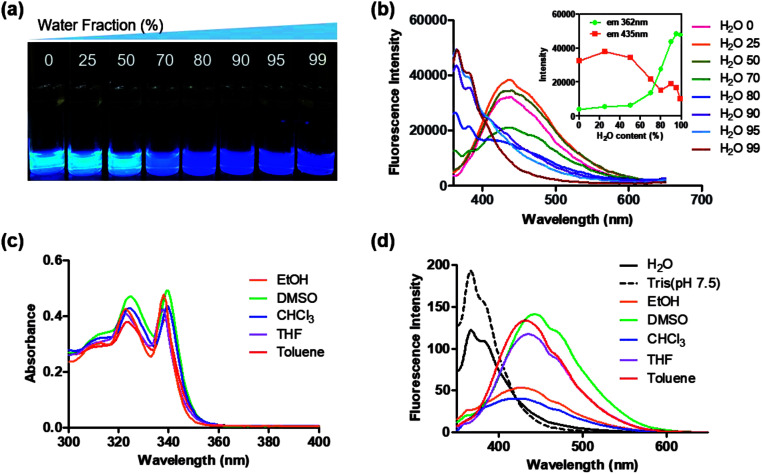
(a) Photographs of 10 μM 8g (0–99%) under UV light (*λ*_ex_ = 312 nm). (b) Fluorescence spectra of 10 μM 8g in THF/water mixture (0–99%) excited at 339 nm; inset: plots of fluorescence intensity at 362 nm and 435 nm. Absorption spectra of 8g in 20 μM (c) and emission spectra in 2 μM (d) measured in various solvents.

**Fig. 5:**
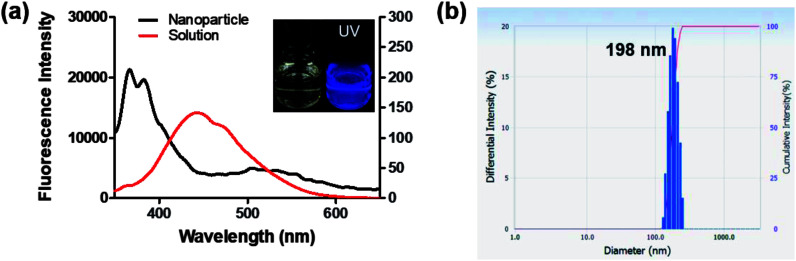
(a) Emission spectra of 8g 10 μM nanoparticles (water : THF = 99 : 1, v/v) and 2 μM in DMSO (*λ*_ex_ = 339 nm); inset: photographs of 8g nanoparticles under UV light (*λ*_ex_ = 312 nm). (b) Particle size intensity distribution of 8g nanoparticles.

Interestingly, 8c, 8g, and 8i showed an unusual hyposochromic shift in water. By increasing the water content in THF, blue-shifted emission at 362 nm was observed as the emission at 435 nm gradually decreased (Fig. 4a, b and S3†). As shown in Fig. 4c and d, the absorption and emission spectra of 8g were insensitive to solvent polarity. Therefore, the observed hyposochromic shift in aqueous solutions was suggested to be more related to the aggregated state emission. Indeed, the intensity of deep-blue emission at 362 nm in 95% water in THF solution increased by 12-fold compared to that in a pure THF solution (Fig. 4b, inset). The observed blue shift was interesting since a bathochromic shift in the aggregated state is more common.^35^ With the addition of DMSO stock to water during sonication, moderately uniform nanoparticles were formed with a diameter of 198 nm (Fig. 5). Compared to the emission of 8g in DMSO, the emission of 8g as nanoparticles exhibited a significant blue shift by approximately 80 nm.

### Bioimaging application with highly fluorescent 5BP series (Group B)

5BP derivatives with an additional aromatic ring exhibited strong fluorescence intensity in solution, as shown in Table 4. Their fluorescence quantum yields in solution were measured with anthracene (*Φ* = 0.27 in EtOH) as a standard and were as high as 56%. 7-Phenylbenzo[4′,5′]imidazo[2′,1′:3,4]pyrazino[1,2-*a*]indole (11, Fig. S2d†) showed comparable spectral features to 6-phenylnaphtho[2′,3′:4,5]imidazo[1,2-*a*]pyrrolo[2,1-*c*]pyrazine (8h, Fig. S2a†) but with a weaker intensity than that of 8h.

**Table 4:** Optical properties and quantum yield of 8h, 8k, 8z, 8ab, 8ae, 8ah, 9, and 11

Compound	*λ*_abs_ (nm)	*λ*_em_ (nm)			*ε*_max_ (M^−1^ cm^−1^)	*Φ*_f_
		H_2_O	EtOH	DMSO		
8h	360	414.0	412.5	422.0	19 900	0.39
8k	361	413.5	412.5	421.5	18 600	0.56
8z	360	416.5	413.0	423.0	25 500	0.43
8ab	361	413.0	412.0	420.5	25 900	0.42
8ae	360	415.5	416.0	425.5	16 900	0.37
8ah	361	416.0	415.5	425.5	19 800	0.34
9	334	355.0	353.0	356.5	29 600	0.15
11	348	421.5	414.0	419.0	17 300	0.25

**Fig. 6:**
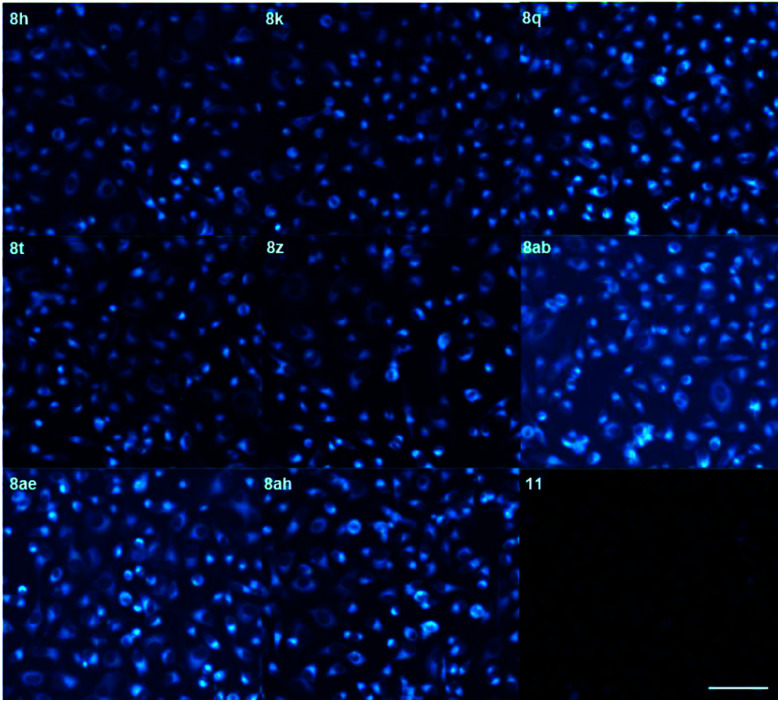
Live-cell imaging and fluorescence of HeLa cells stained with 10 μM 8h, 8k, 8q, 8t, 8z, 8ab, 8ae, 8ah and 11 for 1 h from DAPI channel. Data were collected at 410–530 nm and images were obtained by Operetta high-content imaging system. Scale bar = 100 μm.

The 5BP compounds were also tested for live cell imaging in HeLa cells using the Operetta high-content imaging system (Fig. 6). Extension with an additional aromatic ring in 8h, 8k, 8q, 8t, 8z, 8ab, 8ae, and 8ah showed bright blue fluorescence in the Operetta high-content screening system, demonstrating good cell permeability and potential for bioimaging applications. Meanwhile, extension with an additional benzene fused to the pyrrole side (11) did not induce significant cellular fluorescence. The phototoxicities of 8a, 8h, 8z, and 11 were negligible in HeLa cells, as shown in Fig. S4.†

### Solid state emission and XRD analysis

**Fig. 7:**
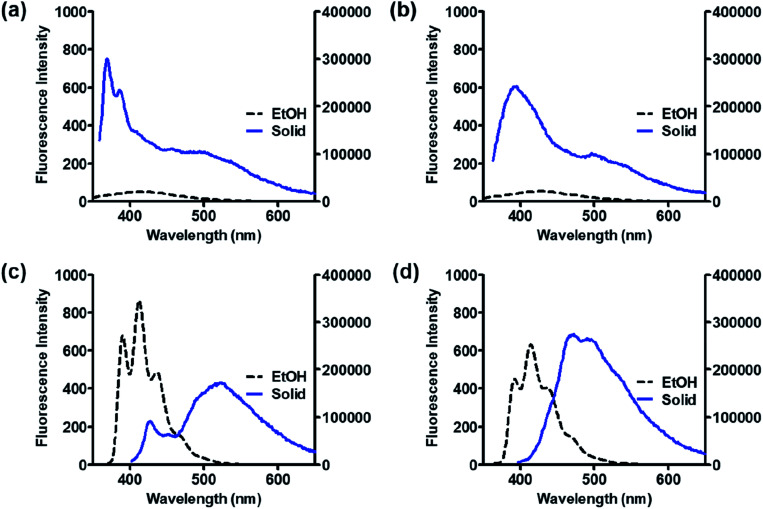
Fluorescence spectra of 8a (a), 8g (b), 8h (c), and 11 (d) in EtOH (2 μM, black) and solid states (blue).

As organic optoelectronic materials work in the solid state, the optical properties of BP scaffolds in the solid state were investigated and are summarized in Table 5. 8h and 11 exhibited significant red shifts of approximately 100 nm in their solid-state emission compared to those in solution, whereas 8a and 8g showed blue shifts in the solid state (Fig. 7).

**Table 5:** Photophysical properties of 8a, 8c, 8g, 8h, and 11 in solid state

Compound	*λ*_ex_ (nm)	*λ*_em_ (nm)	*τ*_f_ (ns)
8a	349	369, 495	7.11
8c	353	370, 481	5.53
8g	353	396, 496	8.31
8h	382	521	4.32
11	377	492	8.95

**Fig. 8:**
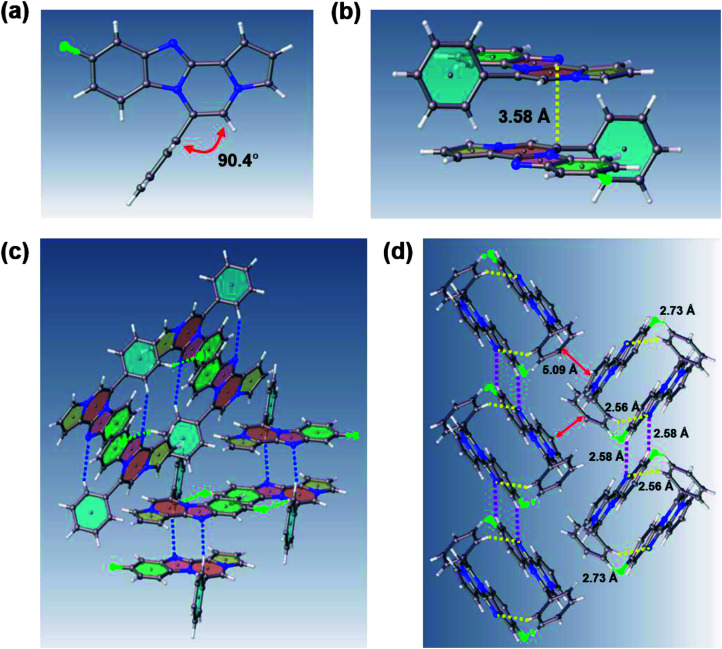
(a) Crystal structure of 8c with a torsion angle of 90.4°. (b) Antiparallel 8c molecules aligned from a side view. (c) Antiparallel molecular packing diagram with intermolecular interactions. (d) Packing structure of 8c including intermolecular interactions; yellow: CH⋯N bond (2.56 Å); purple: CH⋯N bond (2.58 Å); green: CH⋯F bond (2.73 Å).

Intrigued by different patterns of emission spectra induced by aggregation, we investigated the intermolecular interactions of 8c that contributed to the blue emission in the solid and nanoaggregated states. The geometry and packing arrangements were analyzed in crystal states using single-crystal X-ray diffraction (XRD) measurements (Fig. 8).

The single crystal of 8c was depicted as a monomer, two of which were paired in an anti-parallel alignment. Notably, the torsion angle between the phenyl group and benzo[4,5]imidazo[1,2-*a*]pyrrolo[2,1-*c*]pyrazine was 90.4°, suppressing the conjugation between the phenyl ring and ABCD ring (Fig. 8a). As shown in Fig. 8b and c, the molecules were arranged in an antiparallel mode and columnar stacked array with an intermolecular vertical distance of 3.58 Å between two adjacent planes. Each assembled pair was separated from neighboring pairs with a contact distance of 5.09 Å between the phenyl and pyrrole plane centroids (Fig. 8d). Between the antiparallel monomers, there were intermolecular CH⋯N interactions (CH⋯N bond: 2.56 Å) with the phenyl ring and the nitrogen of the imidazole B ring (Fig. 8d), as shown by yellow lines. With the columnar stacked array, the intercolumnar contact between the imidazole (B ring) and fluorobenzene (A ring) formed intermolecular CH⋯N interactions of 2.58 Å, as shown by purple lines. Additionally, nonclassical hydrogen bonds of 8c (CH⋯F bond: 2.73 Å) incorporated the intermolecular network, rigidifying the molecular conformation, which contributed to the aggregation-induced blue shift (Fig. 8d).

Generally, in the aggregated or solid state, the emission is red-shifted by the geometry change in π-stacks from charge transfer or the formation of J-aggregates.^36,37^ On the other hand, solid emission at shorter wavelengths can be induced by modifying the linkage position of substituents, reducing the conjugation effect, or twisting the conformation.^11^ In the case of 8c, the observed blue-shifted emission was ascribed to the weakened intermolecular π–π interactions due to the longer distance between two planes in the crystal packing.^38^ Conformational twisting and spatial restraint also inhibited planarization. Overall, with increasing distance, the conjugation degree was reduced, which caused a blue shift in the solid and nanoparticle states of 8c.

## Conclusions

In conclusion, a novel benzo[*d*]imidazole-pyrrolo[1,2-*a*]pyrazine hybrid system, benzo[4,5]imidazo[1,2-*a*]pyrrolo[2,1-*c*]pyrazine, was designed and synthesized *via* a cascade reaction consisting of double cyclodehydration and aromatization as part of our continued efforts to expand pyrrolo[1,2-*a*]pyrazine-based chemical space. A wide range of derivatives were readily accessed with high atom efficiency by this modular approach under mild reaction conditions. Optical characterization of the synthesized polycyclic N-fused aromatics revealed that the fluorescence intensity and emission properties were significantly affected by attaching different substituents to the R^1^ and R^2^ positions of the benzo[4,5]imidazo[1,2-*a*]pyrrolo[2,1-*c*]pyrazine scaffold. Among the synthesized compounds, 8c, 8g, and 8i considerably showed aggregation-induced blue-shifted emission in the solid and nanoaggregated states, which would be valuable for organic light-emitting diode (OLED) applications. Fusion with an additional benzene ring into a benzo[4,5]imidazo[1,2-*a*]pyrrolo[2,1-*c*]pyrazine scaffold resulted in a remarkable increase in blue fluorescence in solution along with good cell permeability, demonstrating potential for bioimaging applications.

## Experimental section

### General methods

Unless specified, all reagents and starting materials were purchased from commercial sources and used as received without purification. “Concentrated” refers to the removal of volatile solvents *via* distillation using a rotary evaporator. “Dried” refers to pouring onto, or passing through, anhydrous magnesium sulfate followed by filtration. Flash chromatography was performed using silica gel (230–400 mesh) with hexanes, ethyl acetate, and dichloromethane as the eluents. All reactions were monitored by thin-layer chromatography on 0.25 mm silica plates (F-254) visualized with UV light. Melting points were measured using a capillary melting point apparatus. ^1^H and ^13^C NMR spectra were recorded on a 400 MHz NMR spectrometer and were described as chemical shifts, multiplicity (s, singlet; d, doublet; t, triplet; q, quartet; m, multiplet), coupling constant in hertz (Hz), and number of protons. HRMS were measured with an electrospray ionization (ESI) and Q-TOF mass analyzer.

### General procedure A for the synthesis of 8

After a solution of 5 (0.188 mmol) and *o*-phenylenediamine (0.244 mmol, 1.3 equiv.) in TFA (0.056 mmol, 0.3 equiv.)/DMSO (1 mL) was stirred at room temperature for 8 h, the reaction mixture was stirred at 65 °C for additional 3 h. The reaction mixture was quenched with H_2_O (10 mL) and extracted with ethyl acetate (5 mL × 3). The organic layer was dried over MgSO_4_ and concentrated under reduced pressure to give the crude residue, which was purified by silica gel column chromatography (hexane/EtOAc/dichloromethane = 10 : 1 : 2) to afford 8.

### General procedure B for the synthesis of 8

After a solution of 5 (0.188 mmol), *o*-phenylenediamine (0.244 mmol, 1.3 equiv.), and DBSA (0.0188 mmol, 0.1 equiv.) in toluene (1 mL) was stirred at 130 °C for 12 h, the reaction mixture was concentrated *in vacuo*, diluted with H_2_O (10 mL), and extracted with dichloromethane (5 mL × 3). The organic layer was dried over MgSO_4_ and concentrated under reduced pressure to give the crude residue, which was purified by silica gel column chromatography (hexane/EtOAc/dichloromethane = 10 : 1 : 2) to afford 8.

#### 6-Phenylbenzo[4,5]imidazo[1,2-*a*]pyrrolo[2,1-*c*]pyrazine (8a)


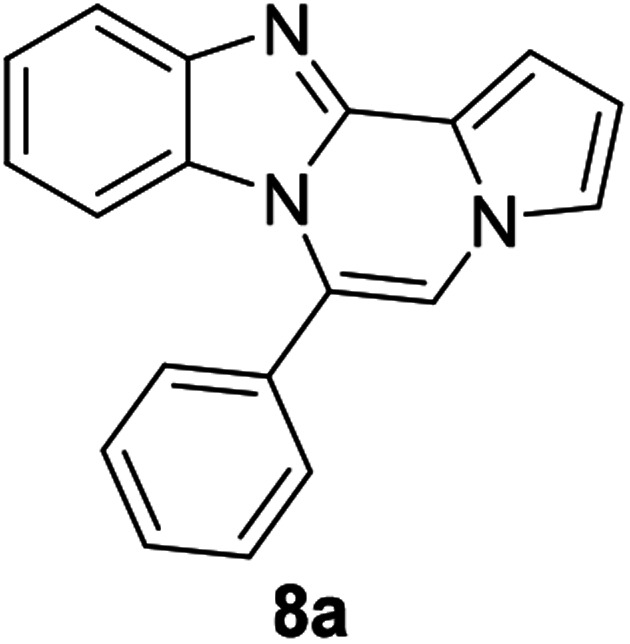
White solid, mp: 219.2–220.8 °C (44.7 mg, 80%); ^1^H NMR (400 MHz, CDCl_3_) *δ* 7.84 (d, *J* = 8.0 Hz, 1H), 7.64–7.57 (m, 5H), 7.34 (d, *J* = 2.8 Hz, 1H), 7.29 (d, *J* = 7.2 Hz, 1H), 7.26 (s, 1H), 7.20 (s, 1H), 6.93 (t, *J* = 7.8 Hz, 1H), 6.74 (t, *J* = 3.0 Hz, 1H), 6.33 (d, *J* = 8.4 Hz, 1H); ^13^C{^1^H} NMR (100 MHz, CDCl_3_) *δ* 144.5, 143.1, 131.3, 130.5, 130.2, 130.1, 128.9, 124.4, 123.7, 121.4, 120.9, 119.4, 117.8, 113.2, 113.0, 111.7, 106.4; HRMS (ESI-QTOF) *m*/*z* [M + H]^+^ calcd for C_19_H_14_N_3_ 284.1182, found 284.1183.

#### 2-(2-(1*H*-Benzo[*d*]imidazol-2-yl)-1*H*-pyrrol-1-yl)-1-phenylethan-1-one (I)


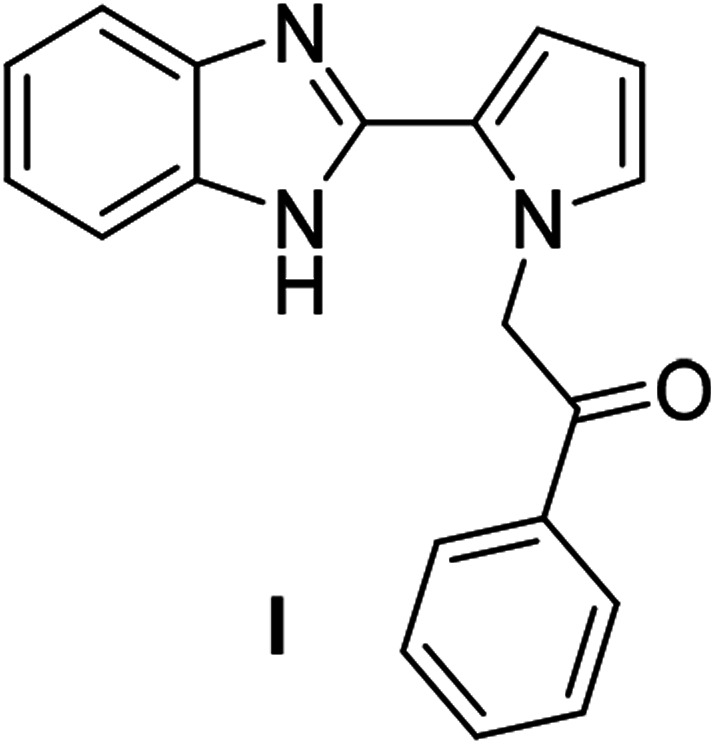
Pale yellow liquid (46.5 mg, 82%); ^1^H NMR (400 MHz, CDCl_3_) *δ* 8.29 (s, 1H), 8.00 (d, *J* = 7.6 Hz, 2H), 7.60 (t, *J* = 7.2 Hz, 1H), 7.48 (d, *J* = 7.6 Hz, 2H), 6.92 (d, *J* = 7.6 Hz, 1H), 6.85–6.84 (m, 2H), 6.75 (d, *J* = 2.4 Hz, 1H), 6.66 (t, *J* = 7.6 Hz, 1H), 6.54 (d, *J* = 7.6 Hz, 1H), 6.34 (t, *J* = 2.8 Hz, 1H), 5.97 (s, 2H); ^13^C{^1^H} NMR (100 MHz, CDCl_3_) *δ* 193.6, 149.6, 141.3, 138.4, 134.8, 133.8, 130.3, 129.6, 128.9, 128.0, 126.4, 120.2, 118.4, 117.3, 114.9, 109.6, 55.7; HRMS (ESI-QTOF) *m*/*z* [M + H]^+^ calcd for C_19_H_16_N_3_O 320.1288, found 302.1289.

#### 9,10-Difluoro-6-phenylbenzo[4,5]imidazo[1,2-*a*]pyrrolo[2,1-*c*]pyrazine (8b)


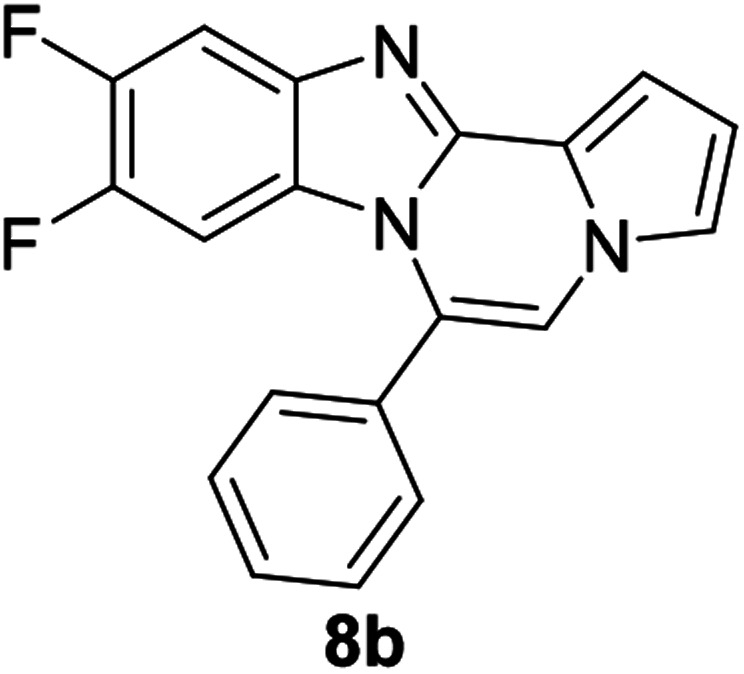
White solid, mp: 223–224 °C (39.6 mg, 66%); ^1^H NMR (400 MHz, CDCl_3_) *δ* 7.68–7.52 (m, 6H), 7.29 (d, *J* = 2.8H, 1H), 7.26 (s, 1H), 7.22 (s, 1H), 6.75–6.73 (m, 1H), 6.06 (dd, *J* = 11,2, 7.4 Hz, 1H); ^13^C{^1^H} NMR (100 MHz, CDCl_3_) *δ* 149.6 (d, *J*_C–F_ = 14.5 Hz), 147.4 (dd, *J*_C–F_ = 37.9, 14.8 Hz), 145.1 (d, *J*_C–F_ = 14.9 Hz), 144.4, 140.1 (d, *J*_C–F_ = 7.9 Hz), 130.6, 130.2, 129.2, 123.3, 118.1, 117.3, 113.4, 112.1, 107.5, 106.6, 106.3 (d, *J*_C–F_ = 19.8 Hz), 101.2 (d, *J*_C–F_ = 24.7 Hz); HRMS (ESI-QTOF) *m*/*z* [M + Na]^+^ calcd for C_19_H_11_F_2_N_3_Na 342.0813, found 342.0812.

#### 10-Fluoro-6-phenylbenzo[4,5]imidazo[1,2-*a*]pyrrolo[2,1-*c*]pyrazine (8c)


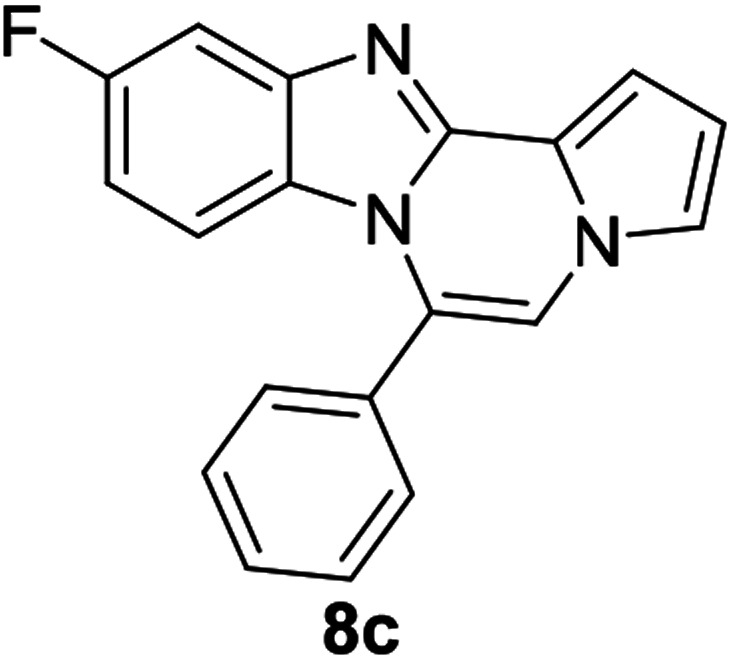
Pale yellow solid, mp: 193.7–194.5 °C (34.6 mg, 61%); ^1^H NMR (400 MHz, CDCl_3_) *δ* 7.64–7.57 (m, 5H), 7.47 (dd, *J* = 9.2, 2.4 Hz, 1H), 7.33 (d, *J* = 3.6 Hz, 1H), 7.28 (t, *J* = 1.2 Hz, 1H), 7.23 (s, 1H), 6.76–6.74 (m, 1H), 6.67 (td, *J* = 9.2, 1.2 Hz, 1H), 6.24–6.20 (m, 1H); ^13^C{^1^H} NMR (100 MHz, CDCl_3_) *δ* 159.4 (d, *J*_C–F_ = 238.5 Hz), 145.4 (d, *J*_C–F_ = 12.9 Hz), 144.3, 131.0, 130.3 (d, *J*_C–F_ = 5.4 Hz), 129.0, 127.1, 124.1, 120.7, 117.9, 113.3 (d, *J*_C–F_ = 4.0 Hz), 113.2, 111.8, 109.6, 109.4, 106.6, 105.0 (d, *J*_C–F_ = 24.0 Hz); HRMS (ESI-QTOF) *m*/*z* [M + Na]^+^ calcd for C_19_H_12_FN_3_Na 324.0907, found 324.0912.

#### 9,10-Dichloro-6-phenylbenzo[4,5]imidazo[1,2-*a*]pyrrolo[2,1-*c*]pyrazine (8d)


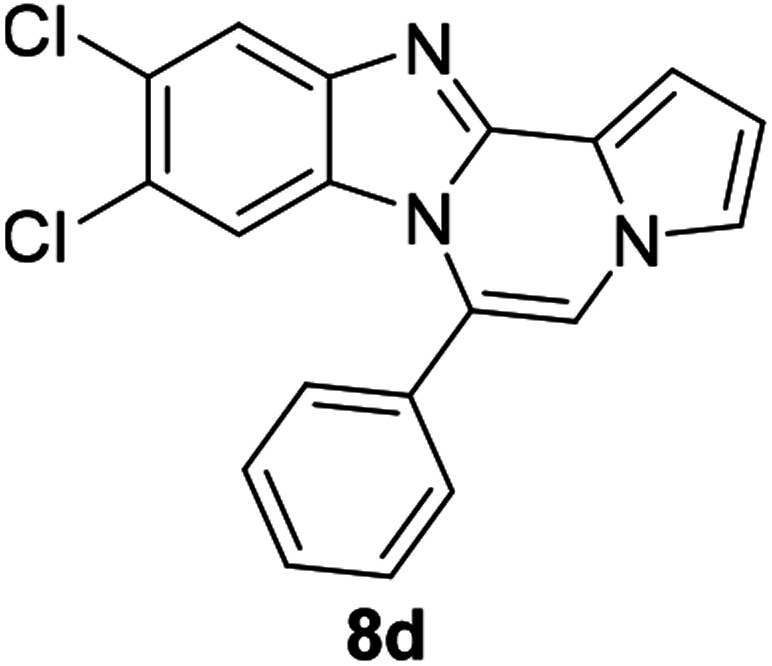
Yellow solid, mp: 256.2–257.1 °C (50.2 mg, 76%); ^1^H NMR (400 MHz, CDCl_3_) *δ* 7.87 (s, 1H), 7.68 (d, *J* = 7.1 Hz, 1H), 7.62 (t, *J* = 7.4 Hz, 2H), 7.57 (d, *J* = 7.2 Hz, 2H), 7.35 (d, *J* = 3.6 Hz, 1H), 7.30 (s, 1H), 7.27 (s, 1H), 6.75 (t, *J* = 7.0 Hz, 1H), 6.35 (s, 1H); ^13^C{^1^H} NMR (100 MHz, CDCl_3_) *δ* 144.6, 143.9, 130.6, 130.4, 130.1, 129.5, 129.2, 127.8, 125.0, 123.9, 120.3, 120.0, 118.5, 114.2, 113.6, 112.2, 107.4; HRMS (ESI-QTOF) *m*/*z* [M + H]^+^ calcd for C_19_H_12_Cl_2_N_3_ 352.0403, found 352.0406.

#### 10-Chloro-6-phenylbenzo[4,5]imidazo[1,2-*a*]pyrrolo[2,1-*c*]pyrazine (8e)


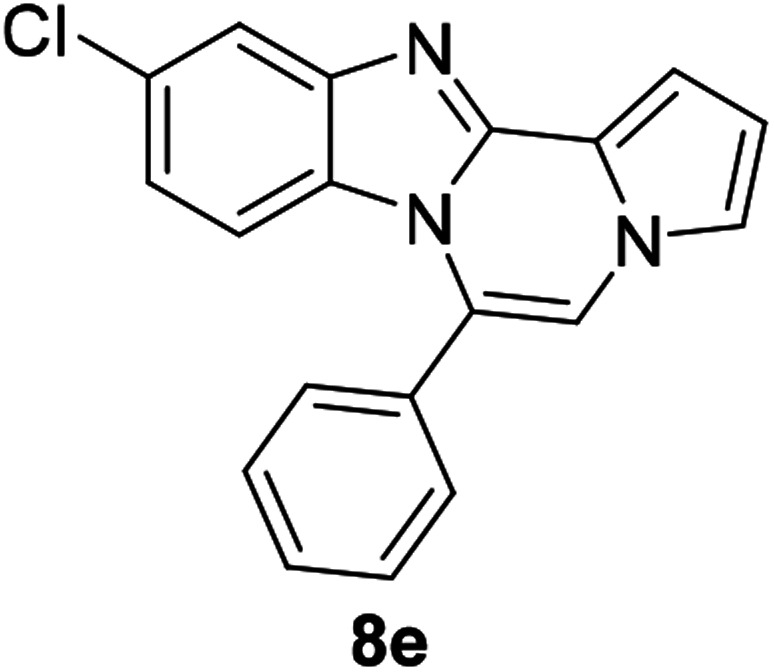
White solid, mp: 169.3–170.1 °C (35.1 mg, 56%); ^1^H NMR (400 MHz, CDCl_3_) *δ* 7.78 (d, *J* = 1.6 Hz, 1H), 7.64–7.55 (m, 5H), 7.34 (d, *J* = 3.6 Hz, 1H), 7.28 (d, *J* = 1.2 Hz, 1H), 7.24 (s, 1H), 6.89 (dd, *J* = 9.0, 1.8 Hz, 1H), 6.76 (t, *J* = 3.2 Hz, 1H), 6.21 (d, *J* = 9.2 Hz, 1H); ^13^C{^1^H} NMR (100 MHz, CDCl_3_) *δ* 145.5, 144.1, 131.0, 130.3, 130.2, 129.3, 129.1, 129.05, 124.1, 121.7, 120.6, 119.0, 118.1, 113.5, 113.4, 112.0, 106.9; HRMS (ESI-QTOF) *m*/*z* [M + H]^+^ calcd for C_19_H_13_ClN_3_ 318.0793, found 318.0791.

#### 9,10-Dibromo-6-phenylbenzo[4,5]imidazo[1,2-*a*]pyrrolo[2,1-*c*]pyrazine (8f)


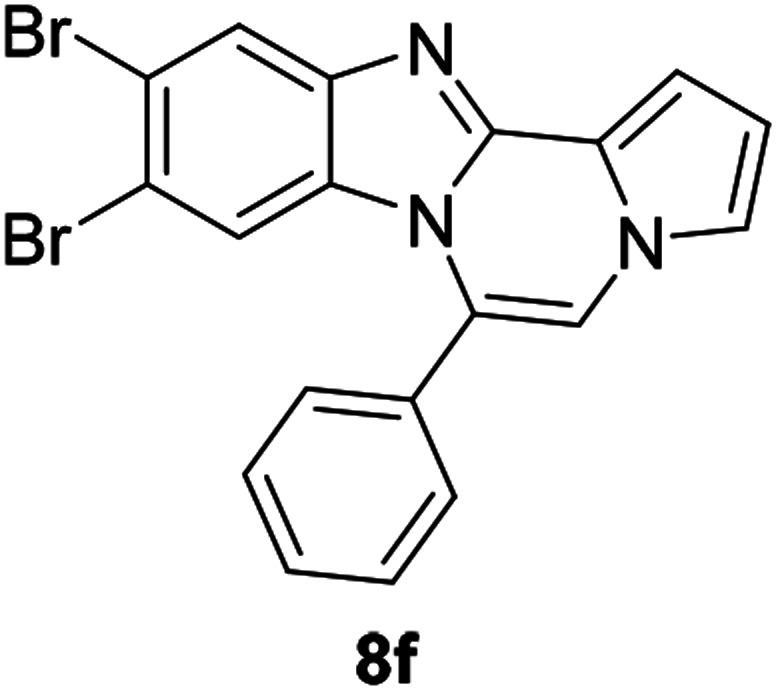
White solid, mp: 289.3–290.2 °C (51.5 mg, 62%); ^1^H NMR (400 MHz, CDCl_3_) *δ* 8.06 (s, 1H), 7.68 (t, *J* = 7.4 Hz, 1H), 7.62 (t, *J* = 7.4 Hz, 2H), 7.56 (d, *J* = 7.2 Hz, 2H), 7.35 (d, *J* = 4.0 Hz, 1H), 7.31–7.30 (m, 1H), 7.26 (s, 1H), 6.78–6.77 (m, 1H), 6.52 (s, 1H); ^13^C{^1^H} NMR (100 MHz, CDCl_3_) *δ* 144.8, 144.4, 130.6, 130.2, 129.2, 123.9, 123.3, 123.29, 120.2, 119.1, 118.6, 117.3, 117.3, 116.2, 113.6, 112.3, 107.5; HRMS (ESI-QTOF) *m*/*z* [M + H]^+^ calcd for C_19_H_12_Br_2_N_3_ 439.9392, found 439.9391.

#### 9,10-Dimethyl-6-phenylbenzo[4,5]imidazo[1,2-*a*]pyrrolo[2,1-*c*]pyrazine (8g)


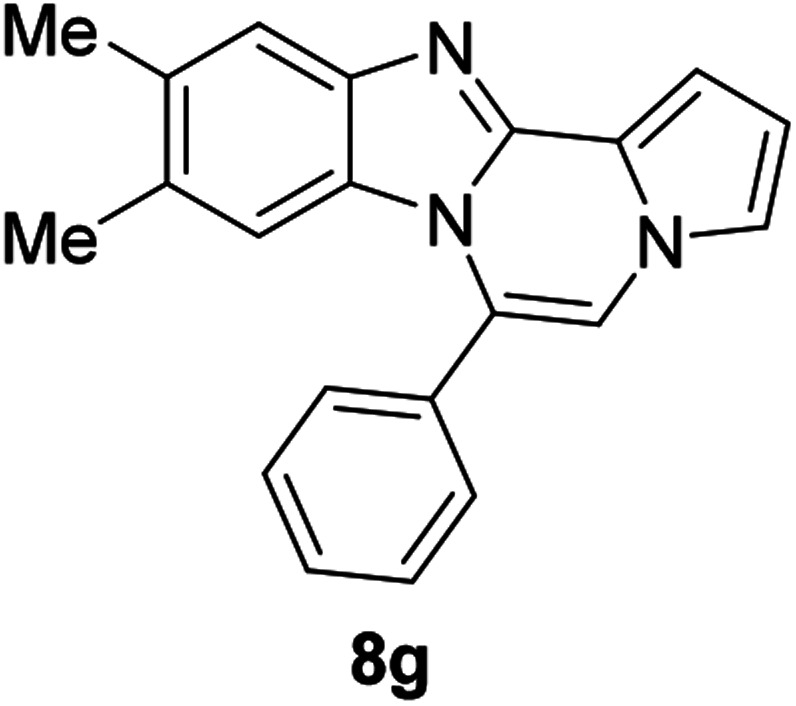
White solid, mp: 230–231.8 °C (47.0 mg, 80%); ^1^H NMR (400 MHz, CDCl_3_) *δ* 7.64–7.56 (m, 6H), 7.29 (d, *J* = 3.6 Hz, 1H), 7.22 (d, *J* = 1.2 Hz, 1H), 7.16 (s, 1H), 6.72 (t, *J* = 3.0 Hz, 1H), 6.07 (s, 1H), 2.33 (s, 3H), 2.10 (s, 3H); ^13^C{^1^H} NMR (100 MHz, CDCl_3_) *δ* 143.1, 142.5, 132.6, 131.5, 130.3, 130.26, 130.0, 128.9, 128.8, 124.4, 121.2, 119.4, 117.4, 113.3, 112.9, 111.3, 105.7, 20.6, 20.2; HRMS (ESI-QTOF) *m*/*z* [M + Na]^+^ calcd for C_21_H_17_N_3_Na 334.1315, found 334.1314.

#### 6-Phenylnaphtho[2′,3′:4,5]imidazo[1,2-*a*]pyrrolo[2,1-*c*]pyrazine (8h)


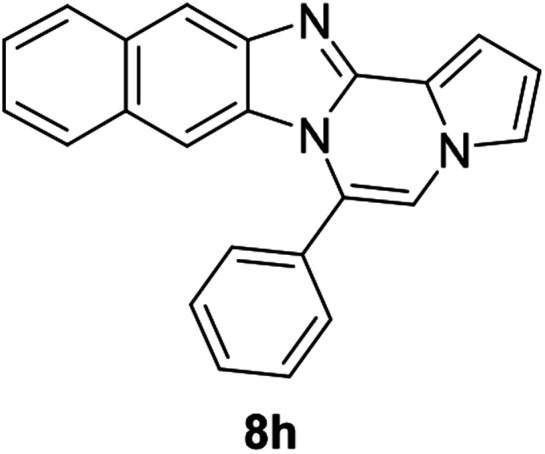
White solid, mp: 232–233 °C (49.6 mg, 79%); ^1^H NMR (400 MHz, CDCl_3_) *δ* 8.25 (s, 1H), 7.95 (d, *J* = 8.4 Hz, 1H), 7.72–7.60 (m, 5H), 7.50 (d, *J* = 8.0 Hz, 1H), 7.44 (d, *J* = 3.2 Hz, 1H), 7.38 (t, *J* = 7.4 Hz, 1H), 7.29 (t, *J* = 7.2 Hz, 2H), 7.20 (s, 1H), 6.77 (t, *J* = 3.2 Hz, 1H), 6.70 (s, 1H); ^13^C{^1^H} NMR (100 MHz, CDCl_3_) *δ* 146.0, 144.4, 131.3, 131.2, 130.8, 130.3, 130.2, 129.2, 129.0, 128.0, 127.8, 124.8, 124.2, 123.8, 120.5, 118.7, 115.4, 113.4, 111.0, 109.8, 108.0; HRMS (ESI-QTOF) *m*/*z* [M + Na]^+^ calcd for C_23_H_15_N_3_Na 356.1158, found 356.1154.

#### 6-(4-Fluorophenyl)benzo[4,5]imidazo[1,2-*a*]pyrrolo[2,1-*c*]pyrazine (8i)


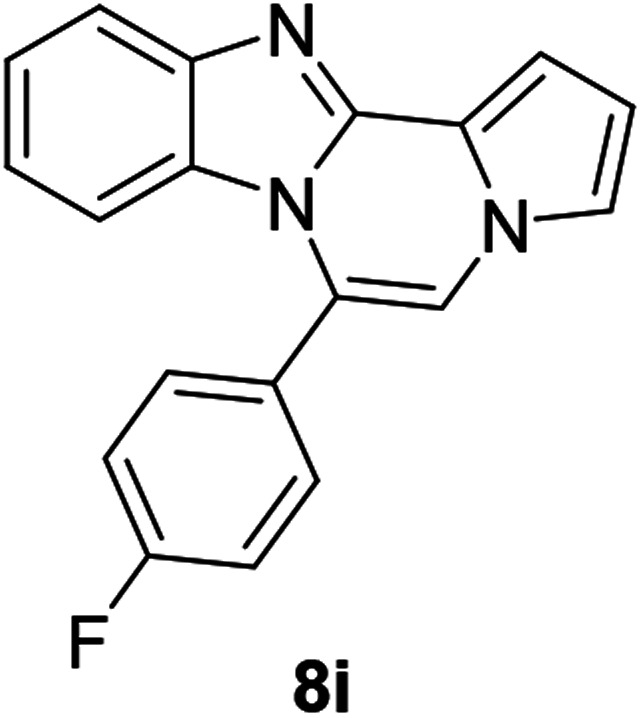
Brown solid, mp: 168–170 °C (36.9 mg, 65%); ^1^H NMR (400 MHz, CDCl_3_) *δ* 7.98 (d, *J* = 8.0 Hz, 1H), 7.63 (d, *J* = 4.8 Hz, 2H), 7.60 (s, 1H), 7.46 (s, 1H), 7.42–7.37 (m, 2H), 7.33 (t, *J* = 7.8 Hz, 2H), 7.08 (t, *J* = 7.8 Hz, 1H), 6.76 (s, 1H), 6.36 (d, *J* = 8.4 Hz, 1H); ^13^C{^1^H} NMR (100 MHz, CDCl_3_) *δ* 165.6 (d, *J*_C–F_ = 251.1 Hz), 147.3, 140.1, 132.5 (d, *J*_C–F_ = 8.5 Hz), 128.8, 126.0, 125.9, 123.5, 122.6, 120.0, 118.1, 117.2, 116.6 (d, *J*_C–F_ = 21.8 Hz), 115.1, 114.1, 113.4, 110.2; HRMS (ESI-QTOF) *m*/*z* [M + H]^+^ calcd for C_19_H_13_FN_3_ 302.1088, found 302.1085.

#### 9,10-Dichloro-6-(4-fluorophenyl)benzo[4,5]imidazo[1,2-*a*]pyrrolo[2,1-*c*]pyrazine (8j)


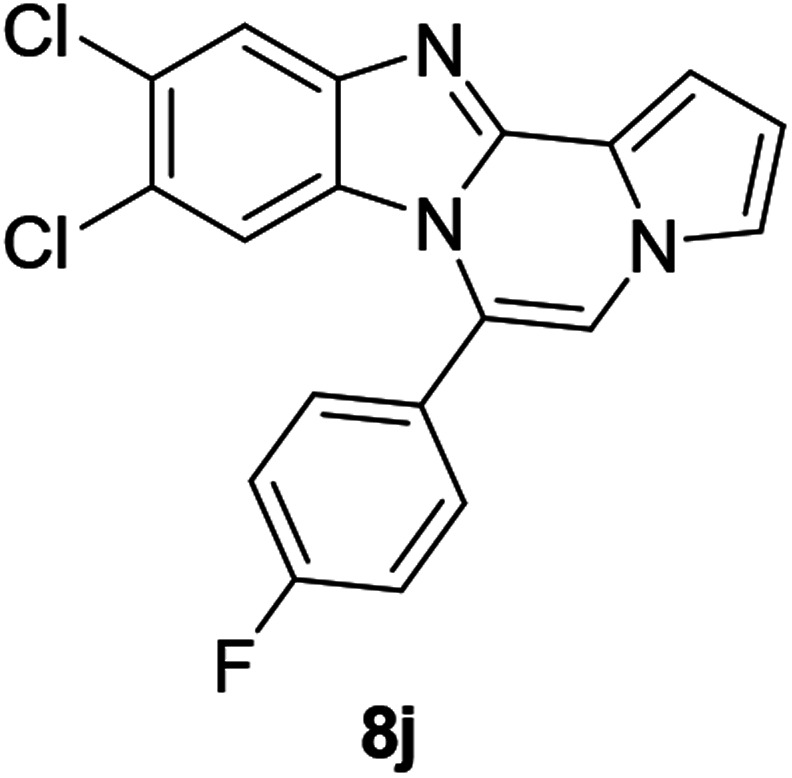
White solid, mp: 301.4–302.5 °C (31.3 mg, 45%); ^1^H NMR (400 MHz, CDCl_3_) *δ* 7.88 (s, 1H), 7.58–7.55 (m, 2H), 7.35–7.30 (m, 4H), 7.24 (s, 1H), 6.78 (t, *J* = 3.2 Hz, 1H), 6.40 (s, 1H); ^13^C{^1^H} NMR (100 MHz, CDCl_3_) *δ* 163.9 (d, *J*_C–F_ = 250.6 Hz), 144.5, 143.9, 132.2 (d, *J*_C–F_ = 8.3 Hz), 129.4, 127.9, 125.1, 122.8, 120.2, 118.6, 116.5 (d, *J*_C–F_ = 21.7 Hz), 116.3, 115.8, 113.8 (d, *J*_C–F_ = 2.9 Hz), 112.6, 107.5, 107.3; HRMS (ESI-QTOF) *m*/*z* [M + H]^+^ calcd for C_19_H_11_Cl_2_FN_3_ 370.0309, found 370.0311.

#### 6-(4-Fluorophenyl)naphtho[2′,3′:4,5]imidazo[1,2-*a*]pyrrolo[2,1-*c*]pyrazine (8k)


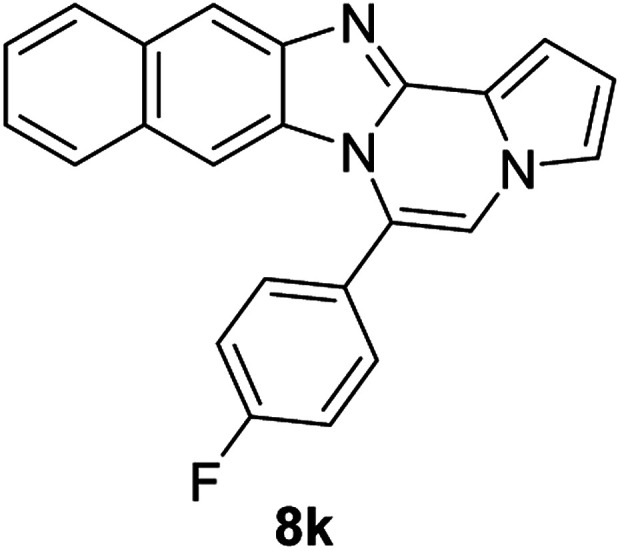
Pale brown solid, mp: 241.5–242.2 °C (57.4 mg, 87%); ^1^H NMR (400 MHz, CDCl_3_) *δ* 8.25 (s, 1H), 7.50 (d, *J* = 8.4 Hz, 1H), 7.63 (t, *J* = 6.8 Hz, 2H), 7.53 (d, *J* = 8.4 Hz, 1H), 7.43 (d, *J* = 3.2 Hz, 1H), 7.39 (t, *J* = 7.4 Hz, 1H), 7.35–7.28 (m, 4H), 7.17 (s, 1H), 6.76 (t, *J* = 2.8 Hz, 1H), 6.72 (s, 1H); ^13^C{^1^H} NMR (100 MHz, CDCl_3_) *δ* 163.8 (d, *J*_C–F_ = 249.5 Hz), 145.9, 144.3, 132.3 (d, *J*_C–F_ = 8.4 Hz), 131.1, 130.9, 129.2, 127.9, 127.8, 127.4, 124.3, 124.0, 123.7, 120.5, 118.8, 116.1 (d, *J*_C–F_ = 21.7 Hz), 115.6, 113.5, 111.3, 109.5, 108.1; HRMS (ESI-QTOF) *m*/*z* [M + H]^+^ calcd for C_23_H_15_FN_3_ 352.1245, found 352.1240.

#### 6-(4-Chlorophenyl)benzo[4,5]imidazo[1,2-*a*]pyrrolo[2,1-*c*]pyrazine (8m)


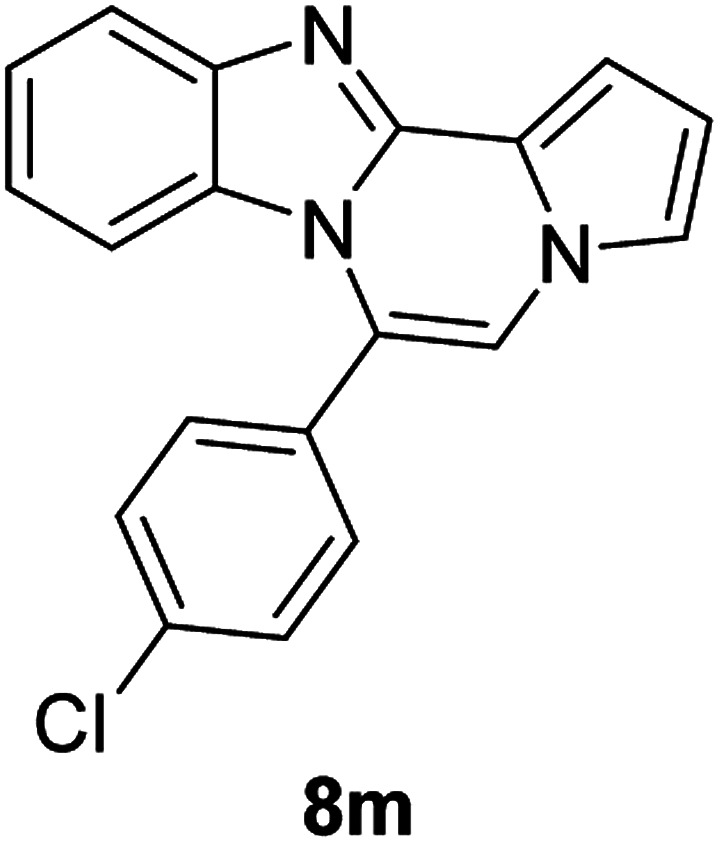
White solid, mp: 208–209.6 °C (51.1 mg, 86%); ^1^H NMR (400 MHz, CDCl_3_) *δ* 7.85 (d, *J* = 8.0 Hz, 1H), 7.58–7.52 (m, 4H), 7.35–7.28 (m, 3H), 7.20 (s, 1H), 7.00 (t, *J* = 8.0 Hz, 1H), 6.76 (t, *J* = 6.8 Hz, 1H), 6.42 (d, *J* = 8.4 Hz, 1H); ^13^C{^1^H} NMR (100 MHz, CDCl_3_) *δ* 144.5, 143.0, 136.3, 131.5, 130.3, 129.8, 129.3, 123.9, 123.2, 121.6, 120.9, 119.5, 117.9, 113.4, 112.8, 112.0, 106.6; HRMS (ESI-QTOF) *m*/*z* [M + H]^+^ calcd for C_19_H_13_ClN_3_ 318.0793, found 318.0798.

#### 6-(4-Chlorophenyl)-9,10-difluorobenzo[4,5]imidazo[1,2-*a*]pyrrolo[2,1-*c*]pyrazine (8n)


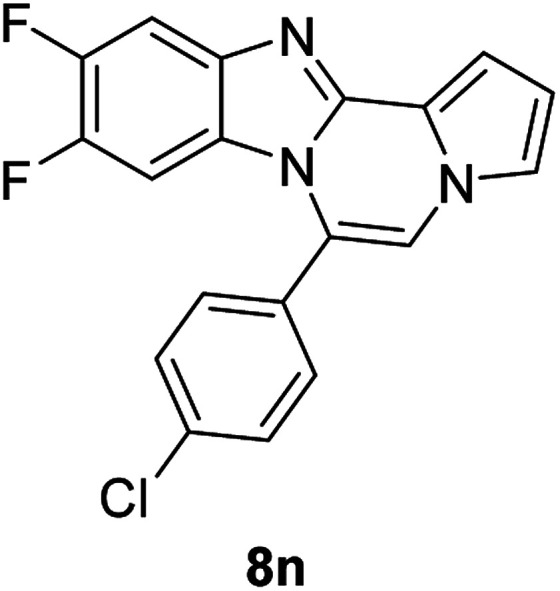
White solid, mp: 232.7–233.4 °C (46.3 mg, 70%); ^1^H NMR (400 MHz, CDCl_3_) *δ* 7.60–7.51 (m, 5H), 7.30 (m, 2H), 7.21 (s, 1H), 6.76 (s, 1H), 6.20 (t, *J* = 8.4 Hz, 1H); ^13^C{^1^H} NMR (100 MHz, CDCl_3_) *δ* 149.6 (d, *J*_C–F_ = 14.1 Hz), 147.4 (dd, *J*_C–F_ = 14.7, 14.4 Hz), 145.2 (d, *J*_C–F_ = 14.9 Hz), 144.3(d, *J*_C–F_ = 2.7 Hz), 140.1 (d, *J*_C–F_ = 10.4 Hz), 136.8, 131.4, 129.5, 128.9, 122.6, 120.5, 118.2, 113.6, 112.4, 106.8, 106.4 (d, *J*_C–F_ = 19.3 Hz), 101.0 (d, *J*_C–F_ = 24.5 Hz); HRMS (ESI-QTOF) *m*/*z* [M + H]^+^ calcd for C_19_H_11_ClF_2_N_3_ 354.0604, found 345.0608.

#### 9,10-Dichloro-6-(4-chlorophenyl)benzo[4,5]imidazo[1,2-*a*]pyrrolo[2,1-*c*]pyrazine (8o)


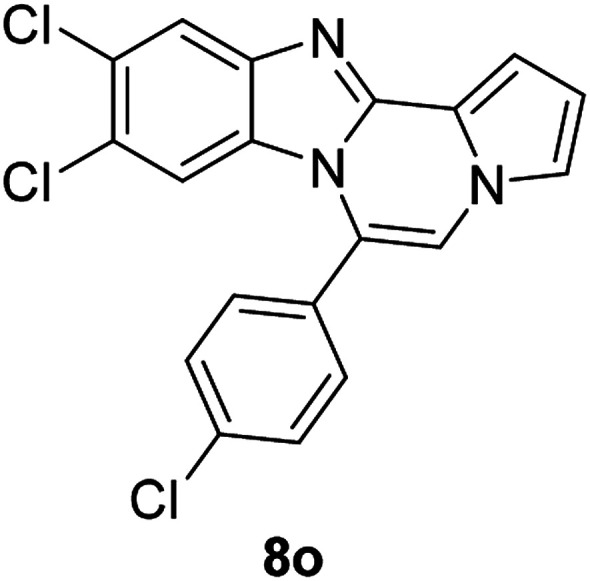
White solid, mp: 294.2–294.4 °C (58.8 mg, 81%); ^1^H NMR (400 MHz, CDCl_3_) *δ* 7.89 (s, 1H), 7.61 (d, *J* = 8.4 Hz, 2H), 7.52 (d, *J* = 8.4 Hz, 2H), 7.34 (d, *J* = 3.2 Hz, 1H), 7.30 (s, 1H), 7.22 (s, 1H), 6.78 (t, *J* = 3.4 Hz, 1H), 6.50 (s, 1H); ^13^C{^1^H} NMR (100 MHz, CDCl_3_) *δ* 144.5, 143.9, 136.9, 131.4, 129.5, 129.3, 128.9, 128.0, 125.2, 122.7, 120.2, 118.6, 113.9, 113.8, 112.6, 107.6; HRMS (ESI-QTOF) *m*/*z* [M + H]^+^ calcd for C_19_H_11_Cl_3_N_3_ 386.0013, found 386.0019.

#### 6-(4-Chlorophenyl)-9,10-dimethylbenzo[4,5]imidazo[1,2-*a*]pyrrolo[2,1-*c*]pyrazine (8p)


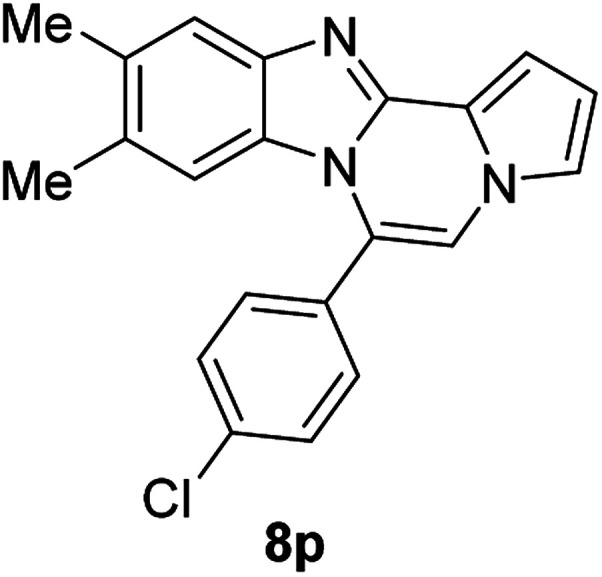
Pale yellow solid, mp: 256.8–257.0 °C (40.8 mg, 63%); ^1^H NMR (400 MHz, CDCl_3_) *δ* 7.60–7.51 (m, 5H), 7.28 (d, *J* = 3.2 Hz, 1H), 7.23 (s, 1H), 7.15 (s, 1H), 6.73 (t, *J* = 3.0 Hz, 1H), 6.18 (s, 1H), 2.34 (s, 3H), 2.15 (s, 3H); ^13^C{^1^H} NMR (100 MHz, CDCl_3_) *δ* 143.1, 142.4, 136.2, 132.8, 131.5, 130.6, 130.0, 129.1, 128.7, 123.2, 121.1, 119.6, 117.5, 113.2, 113.0, 111.6, 106.0, 20.7, 20.6; HRMS (ESI-QTOF) *m*/*z* [M + H]^+^ calcd for C_21_H_17_ClN_3_ 346.1106, found 346.1110.

#### 6-(4-Chlorophenyl)naphtho[2′,3′:4,5]imidazo[1,2-*a*]pyrrolo[2,1-*c*]pyrazine (8q)


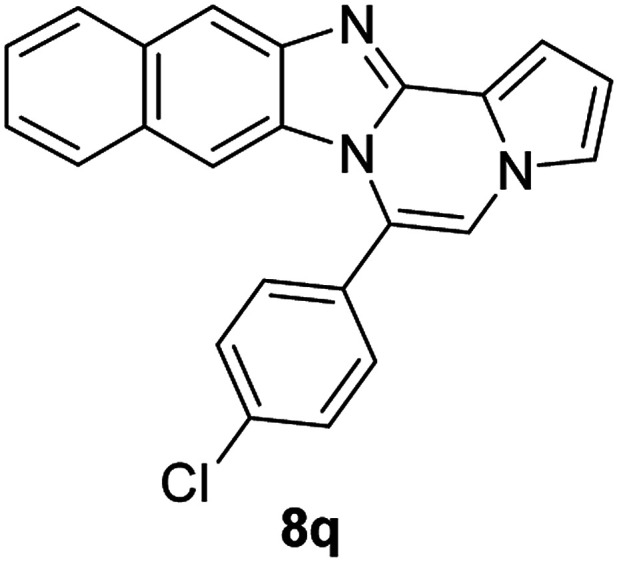
Pale brown solid, mp: 278.2–278.4 °C (49.9 mg, 72%); ^1^H NMR (400 MHz, CDCl_3_) *δ* 8.29 (s, 1H), 7.96 (d, *J* = 8.0 Hz, 1H), 7.64–7.57 (m, 5H), 7.51 (s, 1H), 7.41 (t, *J* = 7.2 Hz, 1H), 7.33 (s, 2H), 7.23 (s, 1H), 6.80 (s, 2H); ^13^C{^1^H} NMR (100 MHz, CDCl_3_) *δ* 136.7, 131.6, 131.0, 130.5, 129.4, 129.3, 128.3, 128.0, 127.8, 124.8, 124.7, 124.3, 123.5, 119.3, 118.3, 117.3, 115.2, 114.0, 111.9, 109.8, 109.1; HRMS (ESI-QTOF) *m*/*z* [M + H]^+^ calcd for C_23_H_15_ClN_3_ 368.0949, found 368.0950.

#### 6-(4-Bromophenyl)benzo[4,5]imidazo[1,2-*a*]pyrrolo[2,1-*c*]pyrazine (8r)


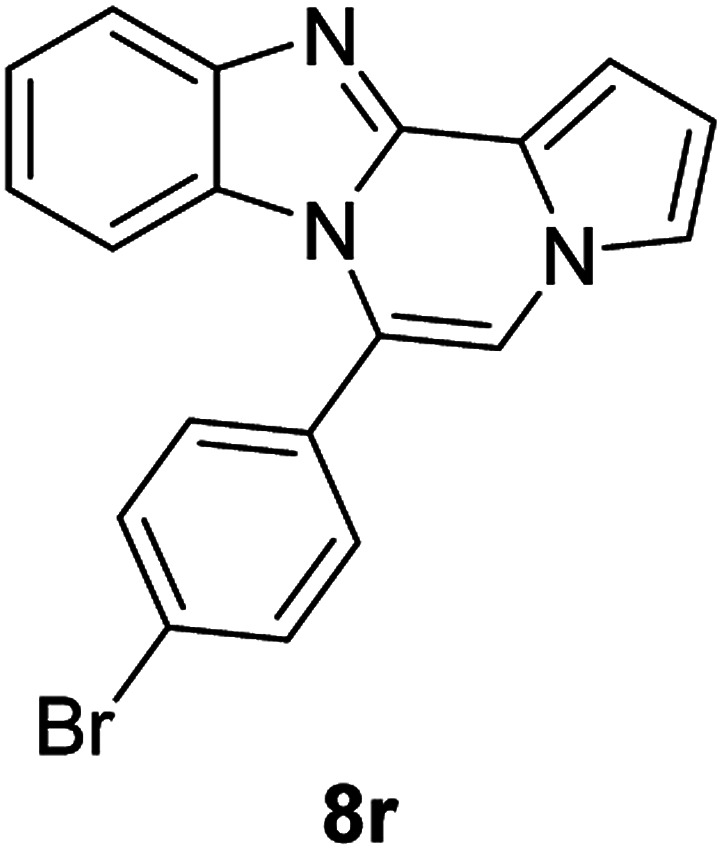
White solid, mp: 197.7–198.4 °C (46 mg, 68%); ^1^H NMR (400 MHz, CDCl_3_) *δ* 7.84 (d, *J* = 8.4 Hz, 1H), 7.72 (d, *J* = 8.0 Hz, 2H), 7.46 (d, *J* = 8.4 Hz, 2H), 7.33 (d, *J* = 3.2 Hz, 1H), 7.30 (d, *J* = 3.6 Hz, 1H), 7.26 (s, 1H), 7.19 (s, 1H), 7.00 (t, *J* = 7.6 Hz, 1H), 6.75 (s, 1H), 6.43 (d, *J* = 8.4 Hz, 1H); ^13^C{^1^H} NMR (100 MHz, CDCl_3_) *δ* 144.5, 143.0, 132.2, 131.7, 130.28, 130.27, 124.5, 123.9, 123.2, 121.6, 120.9, 119.5, 117.9, 113.4, 112.8, 112.0, 106.6; HRMS (ESI-QTOF) *m*/*z* [M + H]^+^ calcd for C_19_H_13_BrN_3_ found 362.0287, found 362.0289.

#### 6-(4-Bromophenyl)-9,10-dichlorobenzo[4,5]imidazo[1,2-*a*]pyrrolo[2,1-*c*]pyrazine (8s)


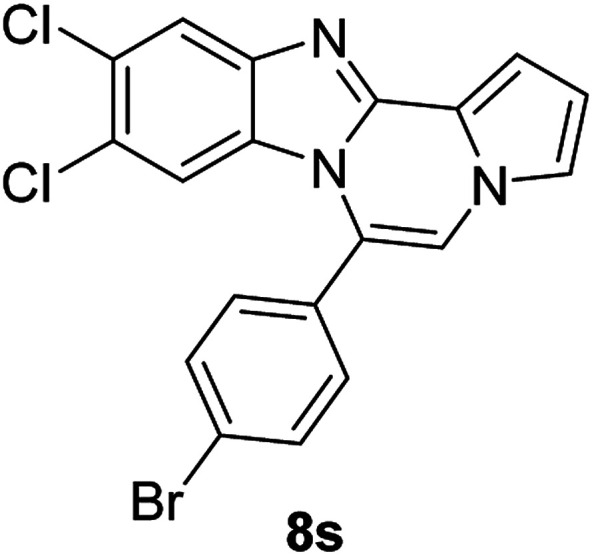
Yellow solid, mp: 266.3–266.9 °C (63.2 mg, 78%); ^1^H NMR (400 MHz, CDCl_3_) *δ* 7.88 (s, 1H), 7.76 (d, *J* = 8.4 Hz, 2H), 7.46 (d, *J* = 8.0 Hz, 2H), 7.34 (d, *J* = 4.0 Hz, 1H), 7.29 (t, *J* = 1.2 Hz, 1H), 7.22 (s, 1H), 6.78–6.77 (m, 1H), 6.50 (s, 1H); ^13^C{^1^H} NMR (100 MHz, CDCl_3_) *δ* 144.7, 144.1, 132.7, 131.7, 129.5, 128.1, 125.6, 125.4, 125.2, 122.9, 120.4, 119.2, 118.8, 114.1, 114.0, 112.8, 107.8; HRMS (ESI-QTOF) *m*/*z* [M + H]^+^ calcd for C_19_H_11_BrCl_2_N_3_ 429.9508, found 429.9507.

#### 6-(4-Bromophenyl)naphtho[2′,3′:4,5]imidazo[1,2-*a*]pyrrolo[2,1-*c*]pyrazine (8t)


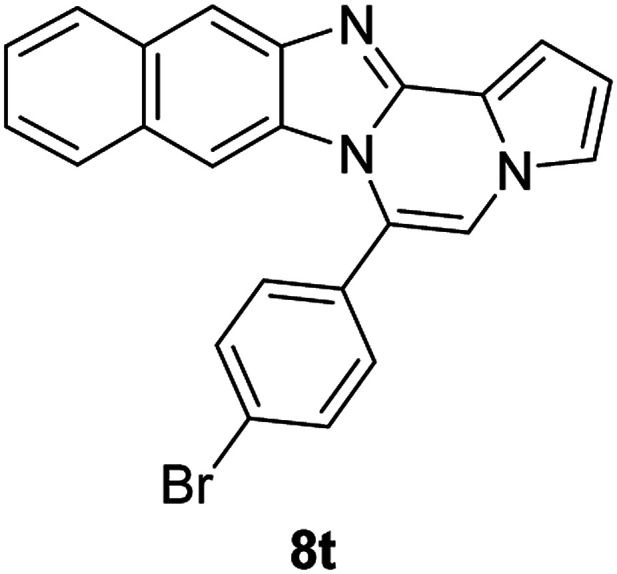
Brown solid, mp: 273.9–274.4 °C (64.4 mg, 84%); ^1^H NMR (400 MHz, CDCl_3_) *δ* 8.27 (s, 1H), 7.95 (d, *J* = 8.0 Hz, 1H), 7.78 (d, *J* = 8.4 Hz, 2H), 7.58–7.52 (m, 3H), 7.48 (d, *J* = 3.6 Hz, 1H), 7.40 (t, *J* = 7.2 Hz, 1H), 7.34 (d, *J* = 7.6 Hz, 1H), 7.30 (s, 1H), 7.21 (s, 1H), 6.80 (s, 1H), 6.76 (t, *J* = 3.2 Hz, 1H); ^13^C{^1^H} NMR (100 MHz, CDCl_3_) *δ* 132.3, 131.8, 131.0, 130.3, 129.8, 129.3, 128.1, 128.0, 127.8, 124.88, 124.86, 124.8, 124.4, 123.5, 119.5, 115.1, 114.2, 112.0, 109.9, 109.4, 109.3; HRMS (ESI-QTOF) *m*/*z* [M + H]^+^ calcd for C_23_H_15_BrN_3_ 412.0444, found 412.0445.

#### 6-(4-Methoxyphenyl)benzo[4,5]imidazo[1,2-*a*]pyrrolo[2,1-*c*]pyrazine (8u)


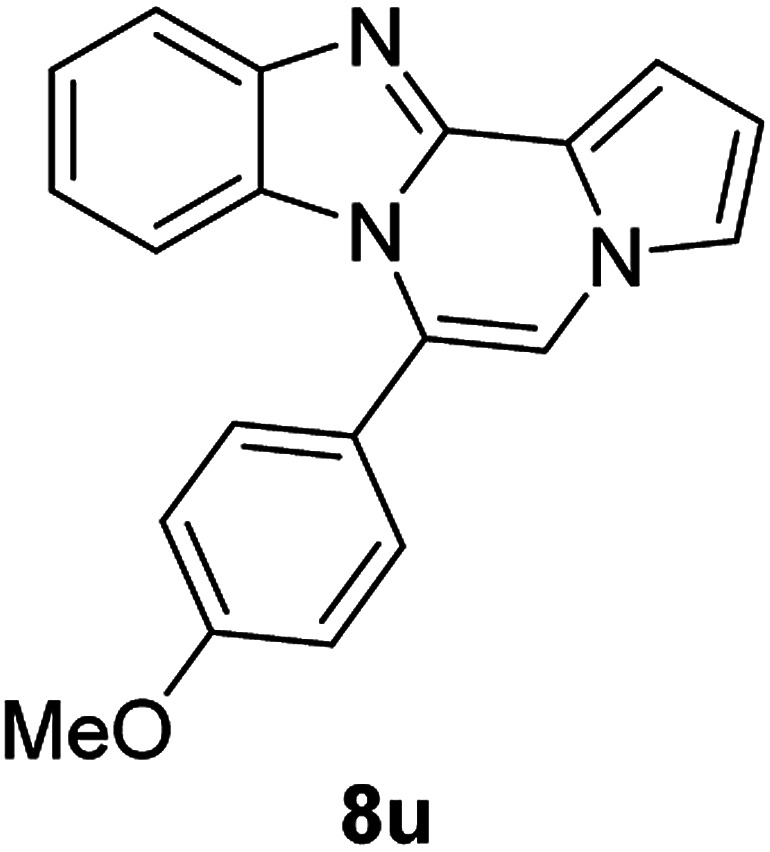
White solid, mp: 206.5–207.2 °C(43.0 mg, 73%); ^1^H NMR (400 MHz, CDCl_3_) *δ* 7.83 (d, *J* = 8.0 Hz, 1H), 7.47 (d, *J* = 8.4 Hz, 2H), 7.32–7.28 (m, 2H), 7.24 (s, 1H), 7.16 (s, 1H), 7.06 (d, *J* = 8.4 Hz, 2H), 6.95 (t, *J* = 7.8 Hz, 1H), 6.72 (t, *J* = 3.0 Hz, 1H), 6.39 (d, *J* = 8.0 Hz, 1H), 3.93 (s, 3H); ^13^C{^1^H} NMR (100 MHz, CDCl_3_) *δ* 160.9, 144.5, 143.1, 131.6, 130.6, 124.1, 123.6, 123.5, 121.4, 120.9, 119.3, 117.7, 114.2, 113.1, 113.0, 111.7, 106.2, 55.5; HRMS (ESI-QTOF) *m*/*z* [M + H]^+^ calcd for C_20_H_16_N_3_O 314.1288, found 314.1287.

#### 9,10-Difluoro-6-(4-methoxyphenyl)benzo[4,5]imidazo[1,2-*a*]pyrrolo[2,1-*c*]pyrazine (8v)


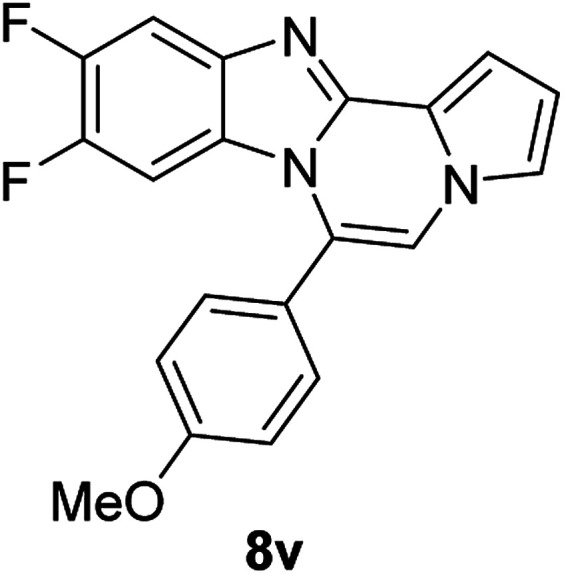
White solid, mp: 206.3–208.2 °C (35.3 mg, 54%); ^1^H NMR (400 MHz, CDCl_3_) *δ* 7.56–7.52 (m, 1H), 7.46 (d, *J* = 8.8 Hz, 2H), 7.27 (d, *J* = 3.6 Hz, 1H), 7.25 (s, 1H), 7.18 (s, 1H), 7.09 (d, *J* = 8.4 Hz, 2H), 6.72 (t, *J* = 3.0 Hz, 1H), 6.18–6.13 (m, 1H), 3.95 (s, 3H); ^13^C{^1^H} NMR (100 MHz, CDCl_3_) *δ* 161.1, 149.5 (d, *J*_C–F_ = 14.6 Hz), 147.2 (dd, *J*_C–F_ = 39.2, 15.0 Hz), 145.1 (d, *J*_C–F_ = 14.8 Hz), 144.4, 140.0 (d, *J*_C–F_ = 10.5 Hz), 131.6, 125.5 (d, *J*_C–F_ = 11.3 Hz), 123.5, 122.5, 120.5, 118.0, 114.5, 112.6 (d, *J*_C–F_ = 120.5), 106.4, 106.1 (d, *J*_C–F_ = 19.5 Hz), 101.2 (d, *J*_C–F_ = 24.7 Hz), 55.5; HRMS (ESI-QTOF) *m*/*z* [M + H]^+^ calcd for C_20_H_14_F_2_N_3_O 350.1099, found 350.1095.

#### 9,10-Dichloro-6-(4-methoxyphenyl)benzo[4,5]imidazo[1,2-*a*]pyrrolo[2,1-*c*]pyrazine (8w)


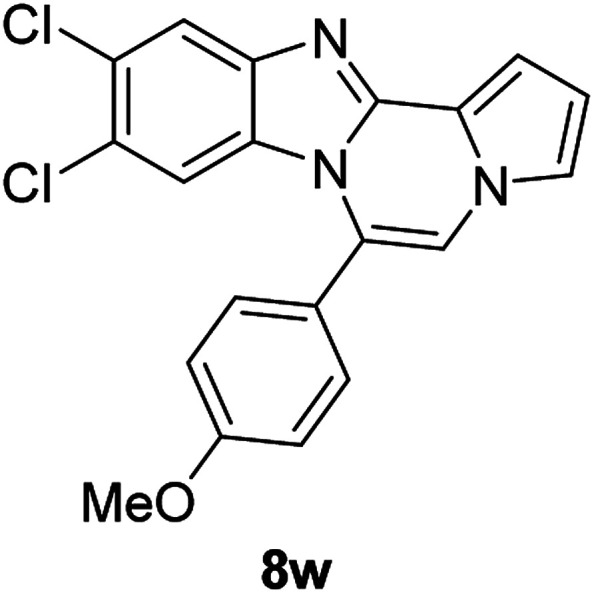
White solid, mp: 226–227.5 °C (65.1 mg, 91%); ^1^H NMR (400 MHz, CDCl_3_) *δ* 7.84 (s, 1H), 7.47 (d, *J* = 8.4 Hz, 2H), 7.30 (d, *J* = 3.2 Hz, 1H), 7.26 (s, 1H), 7.20 (s, 1H), 7.10 (d, *J* = 8.4 Hz, 2H), 6.74 (t, *J* = 2.8 Hz, 1H), 6.44 (s, 1H), 3.95 (s, 3H); ^13^C{^1^H} NMR (100 MHz, CDCl_3_) *δ* 161.2, 144.5, 143.9, 131.5, 129.6, 127.6, 124.9, 123.6, 122.5, 120.2, 119.9, 118.4, 114.5, 114.2, 113.5, 112.3, 107.2, 55.6; HRMS (ESI-QTOF) *m*/*z* [M + H]^+^ calcd for C_20_H_14_Cl_2_N_3_O 382.0508, found 382.0504.

#### 9,10-Dibromo-6-(4-methoxyphenyl)benzo[4,5]imidazo[1,2-*a*]pyrrolo[2,1-*c*]pyrazine (8x)


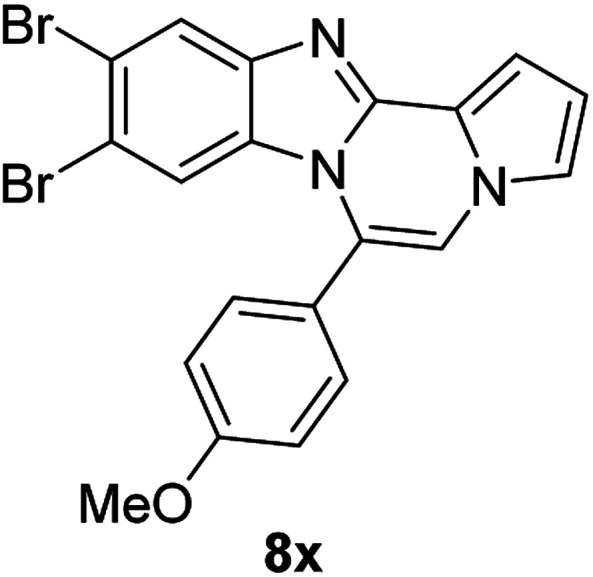
White solid, mp: 217.0–217.2 °C (44.8 mg, 51%); ^1^H NMR (400 MHz, CDCl_3_) *δ* 8.03 (s, 1H), 7.46 (d, *J* = 7.6 Hz, 2H), 7.31 (d, *J* = 3.6 Hz, 1H), 7.27 (s, 1H), 7.20 (s, 1H), 7.11 (d, *J* = 4.4 Hz, 2H), 6.75 (s, 1H), 6.61 (s, 1H), 3.95 (s, 3H); ^13^C{^1^H} NMR (100 MHz, CDCl_3_) *δ* 161.3, 144.8, 144.4, 131.5, 130.5, 123.6, 123.2, 122.5, 120.2, 119.0, 118.4, 117.4, 116.2, 114.5, 113.5, 112.3, 107.3, 55.6; HRMS (ESI-QTOF) *m*/*z* [M + H]^+^ calcd for C_20_H_14_Br_2_N_3_O 469.9498, found 469.9500.

#### 6-(4-Methoxyphenyl)-9,10-dimethylbenzo[4,5]imidazo[1,2-*a*]pyrrolo[2,1-*c*]pyrazine (8y)


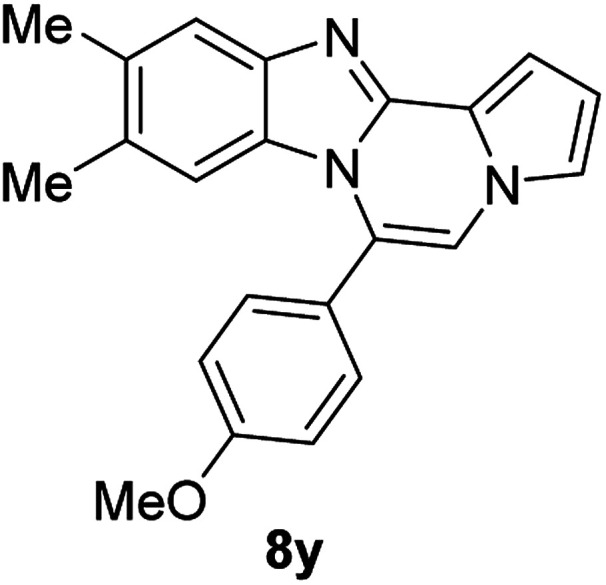
Pale yellow solid, mp: 227.1–228.1 °C (40.7 mg, 63%); ^1^H NMR (400 MHz, CDCl_3_) *δ* 7.59 (s, 1H), 7.48 (d, *J* = 8.4 Hz, 2H), 7.28 (d, *J* = 3.6 Hz, 1H), 7.22 (s, 1H), 7.14 (s, 1H), 7.08 (d, *J* = 8.4 Hz, 2H), 6.71 (s, 1H), 6.18 (s, 1H), 3.95 (s, 3H), 2.34 (s, 3H), 2.13 (s, 3H); ^13^C{^1^H} NMR (100 MHz, CDCl_3_) *δ* 160.8, 143.1, 142.5, 132.6, 131.6, 130.3, 129.0, 124.2, 123.8, 121.2, 119.4, 117.3, 114.1, 113.3, 112.9, 111.3, 105.6, 55.5, 20.7, 20.3; HRMS (ESI-QTOF) *m*/*z* [M + H]^+^calcd for C_22_H_20_N_3_O 342.1601, found 342.1606.

#### 6-(4-Methoxyphenyl)naphtho[2′,3′:4,5]imidazo[1,2-*a*]pyrrolo[2,1-*c*]pyrazine (8z)


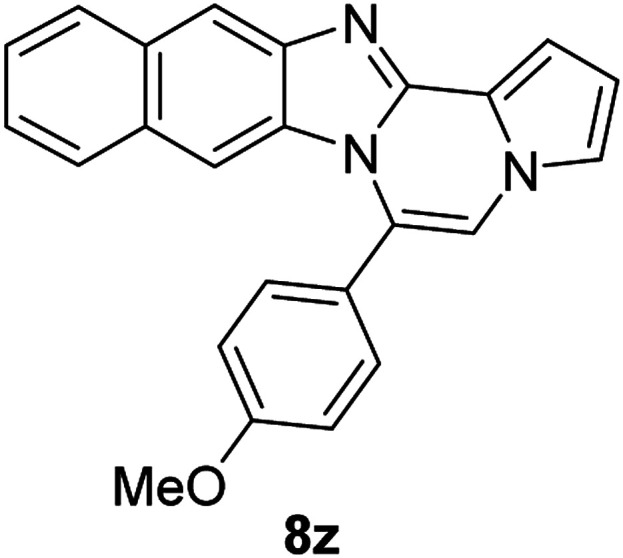
Brown solid, mp: 230.2–231.2 °C (55.3 mg, 81%); ^1^H NMR (400 MHz, CDCl_3_) *δ* 8.24 (s, 1H), 7.95 (d, *J* = 8.4 Hz, 1H), 7.55 (t, *J* = 8.2 Hz, 3H), 7.42 (d, *J* = 2.8 Hz, 1H), 7.38 (t, *J* = 8.4 Hz, 1H), 7.31 (d, *J* = 7.2 Hz, 1H), 7.28 (s, 1H), 7.16 (s, 1H), 7.12 (d, *J* = 8.0 Hz, 2H), 6.80 (s, 1H), 6.75 (s, 1H), 3.98 (s, 3H); ^13^C{^1^H} NMR (100 MHz, CDCl_3_) *δ* 161.0, 146.1, 144.3, 131.7, 131.3, 130.8, 129.2, 128.0, 127.8, 124.6, 124.1, 123.8, 123.4, 120.5, 118.6, 115.4, 114.3, 113.3, 111.0, 109.8, 107.9, 55.6; HRMS (ESI-QTOF) *m*/*z* [M + H]^+^ calcd for C_24_H_18_N_3_O 364.1444, found 364.1448.

#### 6-(2,5-Dimethoxyphenyl)benzo[4,5]imidazo[1,2-*a*]pyrrolo[2,1-*c*]pyrazine (8aa)


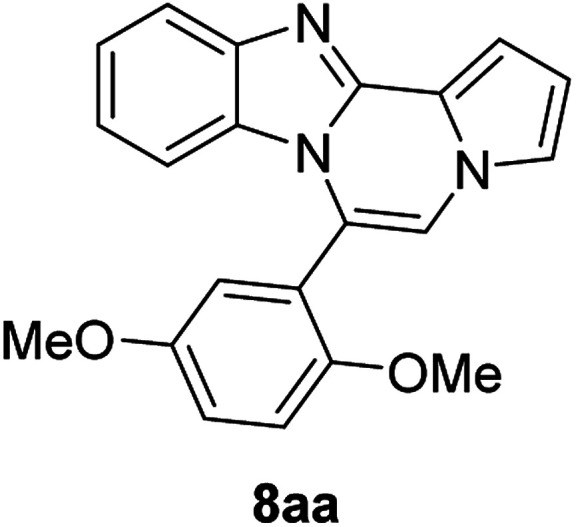
White solid, mp: 187.3–188.2 °C (50.6 mg, 78%); ^1^H NMR (400 MHz, CDCl_3_) *δ* 7.83 (d, *J* = 8.0 Hz, 1H), 7.34–7.28 (m, 2H), 7.26 (s, 1H), 7.23 (s, 1H), 7.16–7.15 (m, 1H), 7.04–6.94 (m, 3H), 6.74 (t, *J* = 3.2 Hz, 1H), 6.44 (d, *J* = 8.0 Hz, 1H), 3.83 (s, 3H), 3.58 (s, 3H); ^13^C{^1^H} NMR (100 MHz, CDCl_3_) *δ* 153.6, 153.0, 144.3, 142.9, 130.9, 123.5, 121.6, 121.4, 121.1, 120.9, 119.2, 117.7, 117.6, 116.6, 113.0, 112.1, 111.9, 111.86, 106.1, 55.9, 55.88; HRMS (ESI-QTOF) *m*/*z* [M + Na]^+^ calcd for C_21_H_17_N_3_NaO_2_ 366.1213, found 366.1211.

#### 6-(2,5-Dimethoxyphenyl)naphtho[2′,3′:4,5]imidazo[1,2-*a*]pyrrolo[2,1-*c*]pyrazine (8ab)


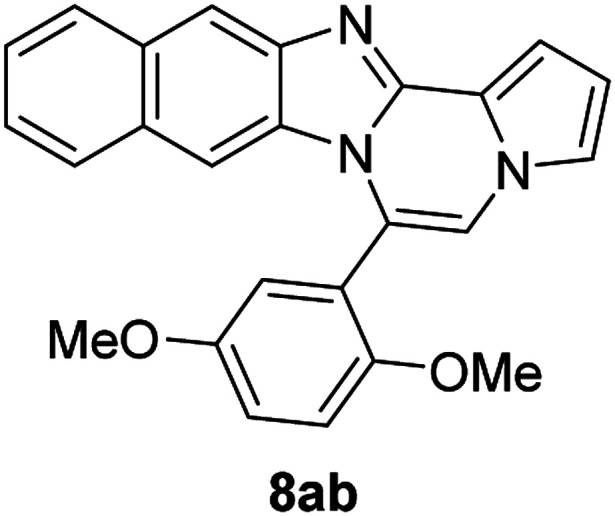
Pale brown solid, mp: 186.5–187.2 °C (60.0 mg, 81%); ^1^H NMR (400 MHz, CDCl_3_) *δ* 8.25 (s, 1H), 7.97 (d, *J* = 8.4 Hz, 1H), 7.56 (d, *J* = 8.0 Hz, 1H), 7.44 (d, *J* = 3.6 Hz, 1H), 7.38 (t, *J* = 7.4 Hz, 1H), 7.31–7.28 (m, 2H), 7.23 (s, 1H), 7.21 (d, *J* = 2.8 Hz, 1H), 7.10 (d, *J* = 2.8 Hz, 1H), 7.05 (d, *J* = 9.2 Hz, 1H), 6.83 (s, 1H), 6.77 (t, *J* = 2.6 Hz, 1H), 3.86 (s, 3H), 3.54 (s, 3H); ^13^C{^1^H} NMR (100 MHz, CDCl_3_) *δ* 153.7, 153.1, 145.9, 144.2, 131.6, 130.9, 129.5, 127.9, 127.8, 124.0, 123.6, 121.9, 120.8, 120.6, 118.6, 117.7, 116.8, 115.2, 113.2, 111.9, 111.2, 108.7, 107.8, 56.0, 55.9; HRMS (ESI-QTOF) *m*/*z* [M + H]^+^ calcd for C_25_H_20_N_3_O_2_ 394.1550, found 394.1552.

#### 6-([1,1′-Biphenyl]-4-yl)benzo[4,5]imidazo[1,2-*a*]pyrrolo[2,1-*c*]pyrazine (8ac)


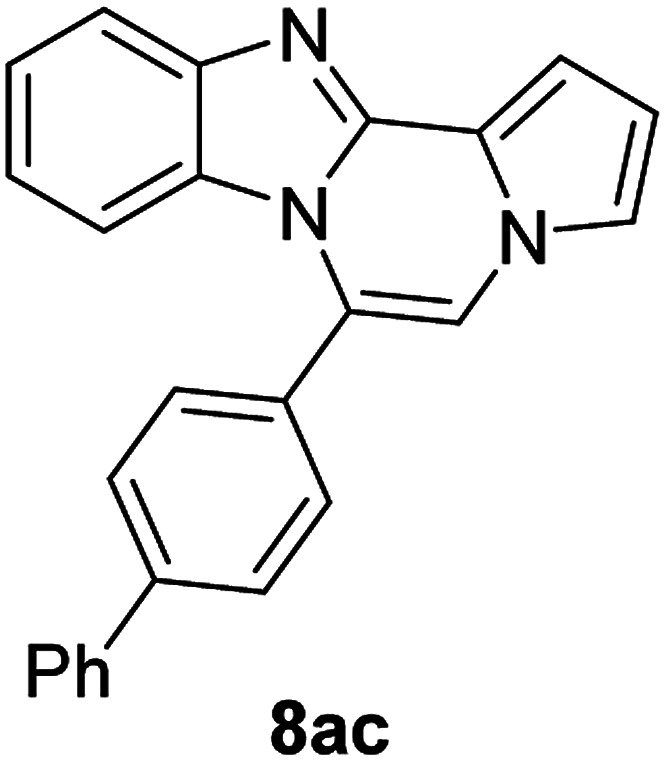
Pale yellow solid, mp: 177.0–177.7 °C (57.6 mg, 85%); ^1^H NMR (400 MHz, CDCl_3_) *δ* 7.86 (d, *J* = 8.4 Hz, 1H), 7.82 (d, *J* = 7.6 Hz, 2H), 7.75 (d, *J* = 8.0 Hz, 2H), 7.66 (d, *J* = 7.6 Hz, 2H), 7.53 (t, *J* = 7.6 Hz, 2H), 7.45 (d, *J* = 7.6 Hz, 1H), 7.36 (d, *J* = 4.0 Hz, 1H), 7.33–7.29 (m, 3H), 6.97 (t, *J* = 7.8 Hz, 1H), 6.76 (t, *J* = 2.8 Hz, 1H), 6.53 (d, *J* = 8.4 Hz, 1H); ^13^C{^1^H} NMR (100 MHz, CDCl_3_) *δ* 144.6, 143.1, 142.9, 139.9, 130.6, 130.5, 130.2, 129.0, 128.0, 127.5, 127.1, 127.0, 124.1, 123.7, 121.5, 121.0, 119.4, 117.8, 113.2, 113.1, 111.8, 106.4; HRMS (ESI-QTOF) *m*/*z* [M + H]^+^ calcd for C_25_H_18_N_3_ 360.1495, found 360.1499.

#### 6-([1,1′-Biphenyl]-4-yl)-9,10-dichlorobenzo[4,5]imidazo[1,2-*a*]pyrrolo[2,1-*c*]pyrazine (8ad)


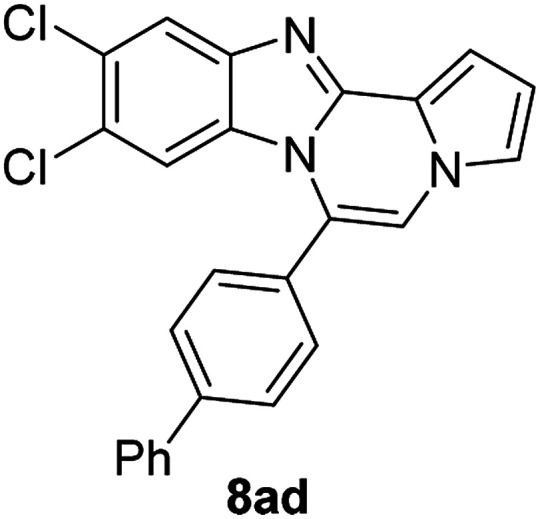
Pale yellow solid, mp: 253.2–254.6 °C (46.4 mg, 58%); ^1^H NMR (400 MHz, CDCl_3_) *δ* 7.87 (s, 1H), 7.83 (d, *J* = 8.0 Hz, 2H), 7.73 (d, *J* = 7.2 Hz, 2H), 7.63 (d, *J* = 8.0 Hz, 2H), 7.53 (t, *J* = 7.6 Hz, 2H), 7.46 (d, *J* = 7.2 Hz, 1H), 7.34 (d, *J* = 3.2 Hz, 1H), 7.28 (d, *J* = 8.4 Hz, 2H), 6.77 (s, 1H), 6.53 (s, 1H); ^13^C{^1^H} NMR (100 MHz, CDCl_3_) *δ* 144.6, 144.0, 143.5, 139.8, 130.5, 129.5, 129.2, 129.0, 128.2, 127.8, 127.2, 125.0, 123.6, 120.3, 120.1, 118.5, 114.3, 113.6, 112.4, 107.4; HRMS (ESI-QTOF) *m*/*z* [M + H]^+^ calcd for C_25_H_16_Cl_2_N_3_ 428.0716, found 428.0719.

#### 6-([1,1′-biphenyl]-4-yl)naphtho[2′,3′:4,5]imidazo[1,2-*a*]pyrrolo[2,1-*c*]pyrazine (8ae)


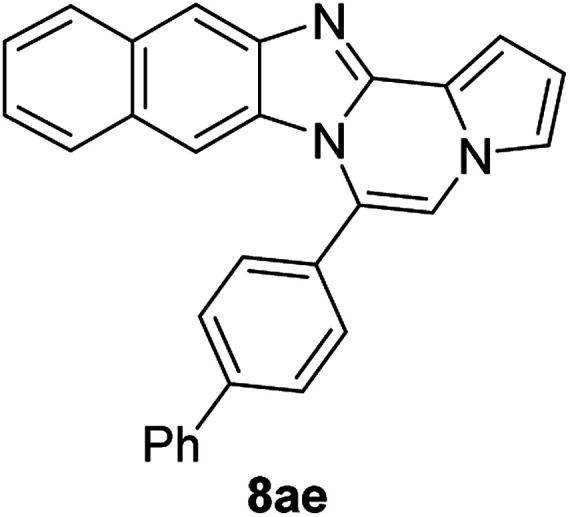
Brown solid, mp: 246.6–245.2 °C (48.3 mg, 63%); ^1^H NMR (400 MHz, CDCl_3_) *δ* 8.27 (s, 1H), 7.97 (d, *J* = 8.4 Hz, 1H), 7.86 (d, *J* = 7.6 Hz, 2H), 7.78 (d, *J* = 7.2 Hz, 2H), 7.72 (d, *J* = 8.0 Hz, 2H), 7.56 (t, *J* = 7.8 Hz, 2H), 7.52–7.45 (m, 3H), 7.38 (t, *J* = 7.4 Hz, 1H), 7.32 (s, 1H), 7.29 (d, *J* = 7.2 Hz, 1H), 7.25 (s, 1H), 6.88 (s, 1H), 6.79 (t, *J* = 3.2 Hz, 1H); ^13^C{^1^H} NMR (100 MHz, CDCl_3_) *δ* 146.1, 144.4, 143.0, 140.0, 131.2, 130.9, 130.6, 130.1, 129.2, 129.1, 128.1, 128.0, 127.8, 127.5, 127.2, 124.6, 124.2, 123.9, 120.6, 118.8, 115.5, 113.4, 111.2, 109.8, 108.0; HRMS (ESI-QTOF) *m*/*z* [M + H]^+^ calcd for C_29_H_20_N_3_ 410.1652, found 410.1651.

#### 6-(Naphthalen-2-yl)benzo[4,5]imidazo[1,2-*a*]pyrrolo[2,1-*c*]pyrazine (8af)


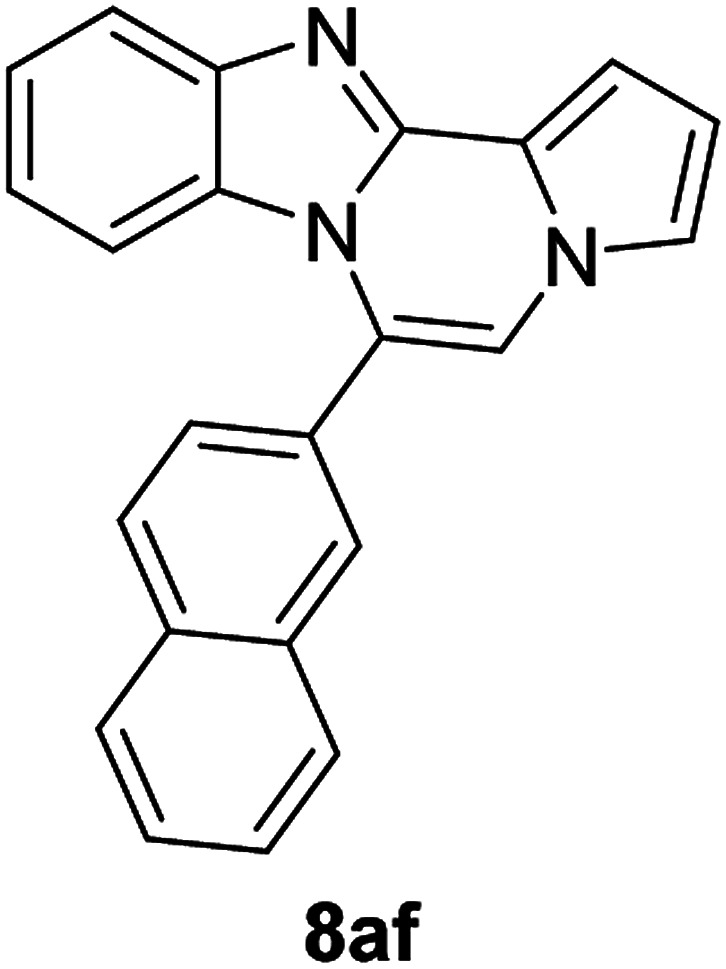
Pale yellow solid, mp: 165.9–166.3 °C (47.8 mg, 75%); ^1^H NMR (400 MHz, CDCl_3_) *δ* 8.13 (s, 1H), 8.06–8.00 (m, 2H) 7.94 (d, *J* = 9.2 Hz, 2H), 7.70–7.61 (m, 3H), 7.53 (s, 1H), 7.39 (s, 1H), 7.37 (s, 1H), 7.32 (t, *J* = 7.8 Hz, 1H), 6.91 (t, *J* = 7.8 Hz, 1H), 6.82 (s, 1H), 6.39 (d, *J* = 7.2 Hz, 1H); ^13^C{^1^H} NMR (100 MHz, CDCl_3_) *δ* 138.7, 133.8, 133.0, 129.9, 129.6, 128.8, 128.4, 128.17, 128.15, 128.0, 127.7, 127.3, 127.1, 127.08, 124.6, 124.59, 124.2, 122.3, 118.7, 118.5, 114.1, 113.4, 112.9; HRMS (ESI-QTOF) *m*/*z* [M + H]^+^ calcd for C_23_H_16_N_3_ 334.1339, found 334.1332.

#### 9,10-Dichloro-6-(naphthalen-2-yl)benzo[4,5]imidazo[1,2-*a*]pyrrolo[2,1-*c*]pyrazine (8ag)


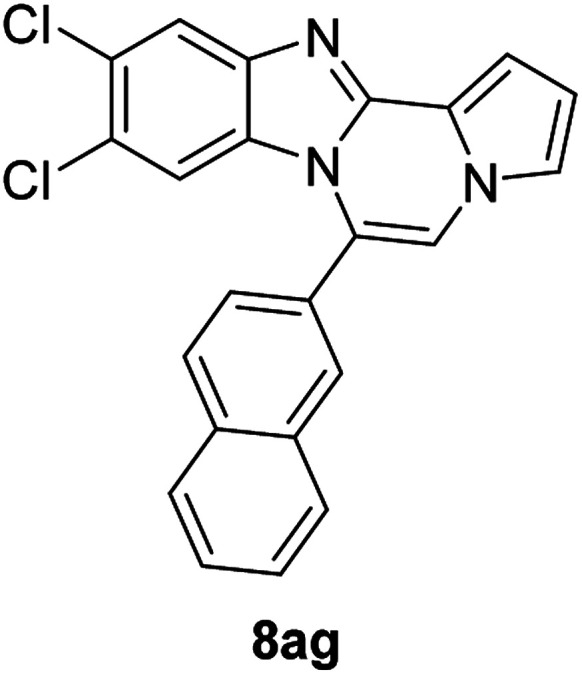
White solid, mp: 273.3–273.8 °C (42.7 mg, 57%); ^1^H NMR (400 MHz, CDCl_3_) *δ* 8.10 (s, 1H), 8.06 (d, *J* = 8.4 Hz, 1H), 8.02 (d, *J* = 7.6 Hz, 1H), 7.96 (d, *J* = 7.2 Hz, 1H), 7.89 (d, *J* = 1.6 Hz, 1H), 7.66 (t, *J* = 5.8 Hz, 2H), 7.59 (d, *J* = 8.4 Hz, 1H), 7.37 (s, 1H), 7.32 (s, 2H), 6.78 (d, *J* = 2.8 Hz, 1H), 6.50 (d, *J* = 1.2 Hz, 1H); ^13^C{^1^H} NMR (100 MHz, CDCl_3_) *δ* 144.6, 144.0, 133.8, 133.0, 129.6, 129.4, 128.9, 128.8, 128.3, 128.1, 127.8, 127.76, 127.7, 127.4, 126.7, 125.0, 124.0, 120.1, 118.5, 114.3, 113.7, 112.7, 107.4; HRMS (ESI-QTOF) *m*/*z* [M + H]^+^ calcd for C_23_H_14_Cl_2_N_3_ 402.0599, found 402.0593.

#### 6-(Naphthalen-2-yl)naphtho[2′,3′:4,5]imidazo[1,2-*a*]pyrrolo[2,1-*c*]pyrazine (8ah)


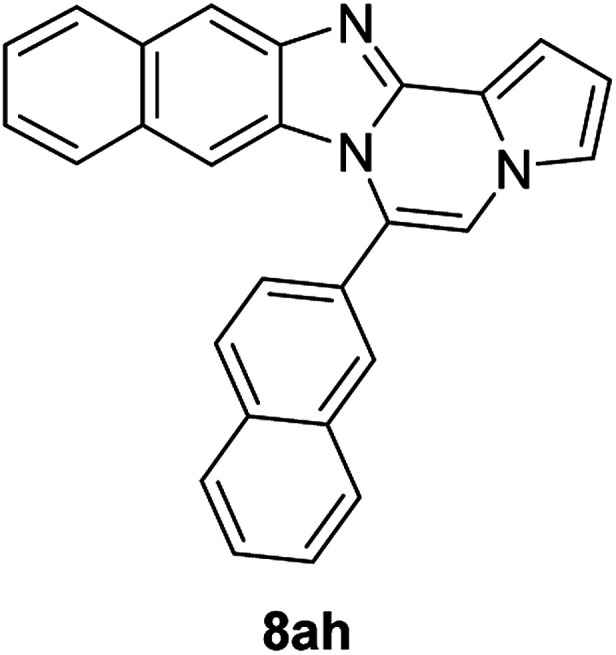
Yellow solid, mp: 151–152 °C (57.0 mg, 79%); ^1^H NMR (400 MHz, DMSO-d_6_) *δ* 8.39 (s, 1H), 8.25 (s, 1H), 8.21 (d, *J* = 8.4 Hz, 1H), 8.17 (d, *J* = 7.6 Hz, 1H), 8.10 (d, *J* = 8.0 Hz, 1H), 7.99 (d, *J* = 8.4 Hz, 1H), 7.96 (s, 1H), 7.85 (d, *J* = 8.4 Hz, 1H) 7.76–7.68 (m, 3H), 7.36 (t, *J* = 7.2 Hz, 1H), 7.32 (d, *J* = 3.2 Hz, 1H), 7.28–7.21 (m, 2H), 6.84–6.82 (m, 1H), 6.70 (s, 1H); ^13^C{^1^H} NMR (100 MHz, DMSO-d_6_) *δ* 146.1, 144.6, 135.6, 133.8, 133.1, 130.7, 129.7, 129.0, 128.8, 128.79, 128.7, 128.4, 128.0, 127.9, 127.6, 124.6, 124.4, 124.3, 120.6, 120.1, 115.2, 113.6, 112.5, 109.6, 109.0, 108.9, 107.9; HRMS (ESI-QTOF) *m*/*z* [M + H]^+^ calcd for C_27_H_18_N_3_ 384.1495, found 384.1494.

#### 6-Methylbenzo[4,5]imidazo[1,2-*a*]pyrrolo[2,1-*c*]pyrazine (8ai)


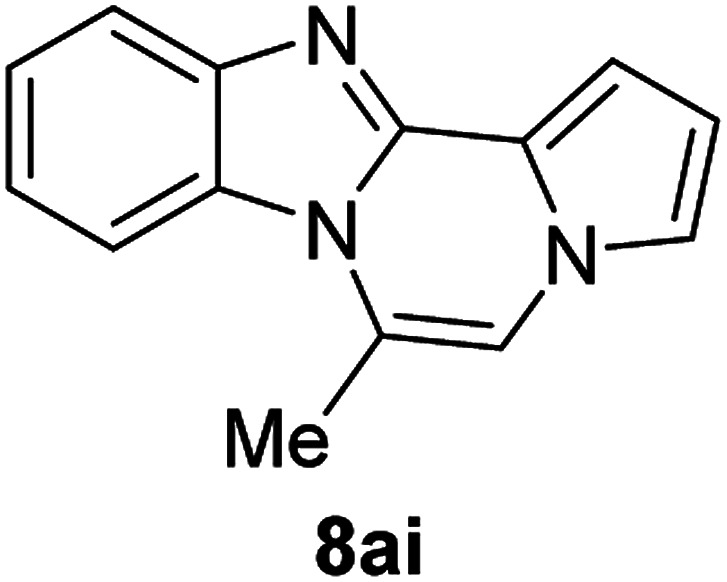
Brown solid, mp: 153.4–153.6 °C (33.6 mg, 80%); ^1^H NMR (400 MHz, CDCl_3_) *δ* 7.86 (d, *J* = 8.0 Hz, 1H), 7.81 (d, *J* = 8.4 Hz, 1H), 7.36 (t, *J* = 7.6 Hz, 1H), 7.25–7.20 (m, 2H), 7.13 (t, *J* = 1.2 Hz, 1H), 6.99 (s, 1H), 6.65 (t, *J* = 3.0 Hz, 1H), 2.75 (s, 3H); ^13^C{^1^H} NMR (100 MHz, CDCl_3_) *δ* 139.3, 137.9, 125.9, 118.5, 116.6, 115.4, 115.3, 114.3, 111.8, 107.3, 107.29, 104.5, 100.7, 12.5; HRMS (ESI-QTOF) *m*/*z* [M + H]^+^ calcd for C_14_H_12_N_3_ 222.1026, found 222.1025.

Compounds 9, 11, and 13 were synthesized by following the same procedure for the synthesis of 8 except that a higher reaction temperature was needed in case of 9.

#### Benzo[4,5]imidazo[1,2-*a*]pyrrolo[2,1-*c*]pyrazine (9)


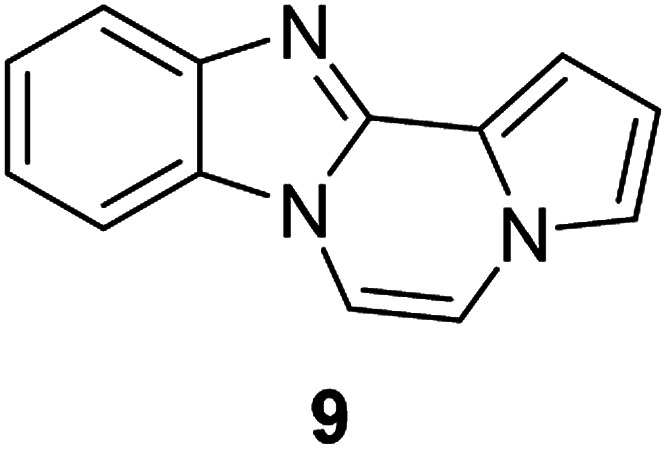
Pale yellow solid, mp: 148–148.9 °C (10.5 mg, 21%); ^1^H NMR (400 MHz, CDCl_3_) *δ* 7.87 (d, *J* = 8.0 Hz, 1H), 7.59 (d, *J* = 7.6 Hz, 1H), 7.42–7.30 (m, 6H), 6.71 (d, *J* = 1.6 Hz, 1H); ^13^C{^1^H} NMR (100 MHz, CDCl_3_) *δ* 144.1, 142.5, 141.9, 130.0, 124.1, 122.0, 119.5, 118.3, 113.0, 112.4, 109.0, 108.2, 106.6; HRMS (ESI-QTOF) *m*/*z* [M + H]^+^ calcd for C_13_H_10_N_3_ 208.0869, found 208.0868.

#### 7-Phenylbenzo[4′,5′]imidazo[2′,1′:3,4]pyrazino[1,2-*a*]indole (11)


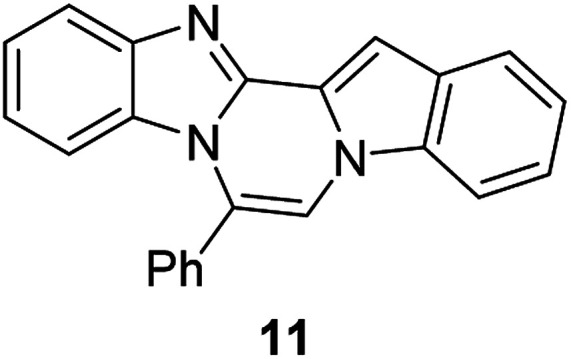
Pale yellow solid, mp: 189.7–190.2 °C (62.7 mg, 100%); ^1^H NMR (400 MHz, CDCl_3_) *δ* 7.91 (t, *J* = 7.2 Hz, 2H), 7.71 (d, *J* = 5.6 Hz, 1H), 7.66–7.59 (m, 6H), 7.52 (d, *J* = 2.8 Hz, 1H), 7.42–7.31 (m, 3H), 7.03–6.99 (m, 1H), 6.43 (d, *J* = 8.4 Hz, 1H); ^13^C{^1^H} NMR (100 MHz, CDCl_3_) *δ* 144.4, 142.7, 132.4, 131.6, 130.8, 130.4, 130.1, 128.9, 128.8, 125.2, 123.8, 123.2, 122.7, 122.4, 122.3, 122.0, 119.8, 113.2, 109.8, 109.5, 99.1; HRMS (ESI-QTOF) *m*/*z* [M + H]^+^ calcd for C_23_H_16_N_3_ 334.1339, found 334.1334.

#### 5-Methyl-6-phenylbenzo[4,5]imidazo[1,2-*a*]pyrrolo[2,1-*c*]pyrazine (13a)


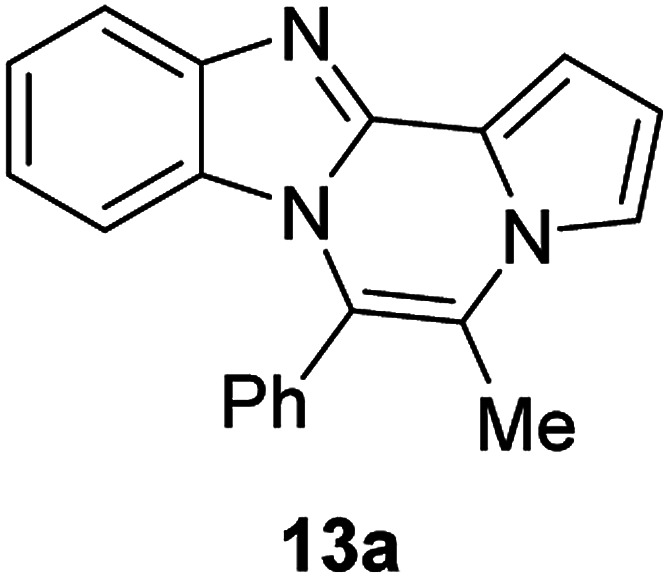
Pale yellow solid, mp: 188.0–188.6 °C (41.1 mg, 74%); ^1^H NMR (400 MHz, CDCl_3_) *δ* 7.81 (d, *J* = 8.0 Hz, 1H), 7.68–7.63 (m, 3H), 7.51 (d, *J* = 6.4 Hz, 2H), 7.43 (s, 1H), 7.34 (s, 1H), 7.24 (s, 1H), 6.88–6.82 (m, 2H), 5.87 (d, *J* = 8.4 Hz, 1H), 2.32 (s, 3H); ^13^C{^1^H} NMR (100 MHz, CDCl_3_) 144.2, 142.5, 132.0, 131.2, 130.6, 130.1, 129.4, 128.9, 126.7, 123.5, 121.3, 121.1, 120.7, 119.0, 117.8, 115.8, 113.1, 112.7, 106.8, 14.3; HRMS (ESI-QTOF) *m*/*z* [M + Na]^+^ calcd for C_20_H_15_N_3_Na 320.1158, found 320.1157.

#### 5-Benzyl-6-phenylbenzo[4,5]imidazo[1,2-*a*]pyrrolo[2,1-*c*]pyrazine (13b)


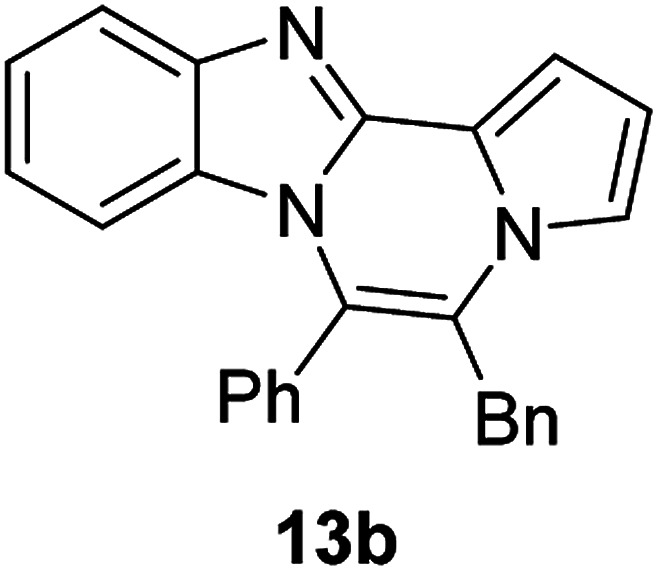
Yellow solid, mp: 183.1–183.5 °C (40.6 mg, 81%); ^1^H NMR (400 MHz, CDCl_3_) *δ* 7.86 (d, *J* = 8.0 Hz, 1H), 7.66 (d, *J* = 6.8 Hz, 1H), 7.63–7.57 (m, 4H), 7.42 (d, *J* = 2.8 Hz, 1H), 7.34–7.29 (m, 4H), 7.22 (d, *J* = 7.2 Hz, 2H), 7.13 (s, 1H), 6.91 (t, *J* = 7.8 Hz, 1H), 6.68 (s, 1H), 5.88 (d, *J* = 7.6 Hz, 1H), 4.12 (s, 2H); ^13^C{^1^H} NMR (100 MHz, CDCl_3_) 144.4, 142.6, 136.4, 131.6, 130.9, 130.7, 130.3, 129.5, 129.0, 127.4, 127.0, 123.6, 122.7, 121.2, 119.3, 119.2, 117.2, 113.0, 112.7, 106.6, 34.0; HRMS (ESI-QTOF) *m*/*z* [M + Na]^+^ calcd for C_26_H_19_N_3_Na 396.1471, found 396.1477.

### Synthesis of 14

After a mixture of 8a (0.0388 mmol) and iodomethane (0.5 mL) in CH_2_Cl_2_ (0.5 mL) was stirred at rt for 3 h, the reaction mixture was concentrated under reduced pressure to give 14 (23.8 mg, 100%).

#### 12-Methyl-6-phenylbenzo[4,5]imidazo[1,2-*a*]pyrrolo[2,1-*c*]pyrazin-12-ium iodide (14)


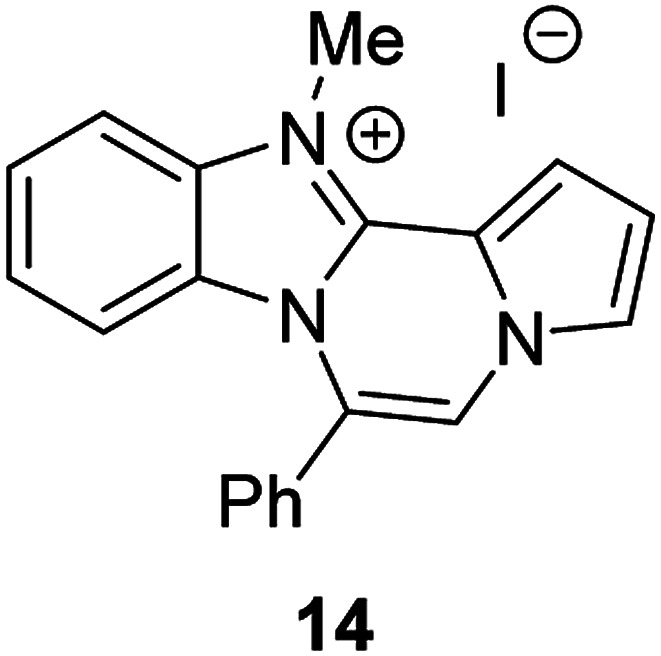
Yellow solid, mp: 297–298 °C (23.8 mg, 100%); ^1^H NMR (400 MHz, DMSO-d_6_) *δ* 8.43 (s, 1H), 8.14–8.13 (m, 2H), 7.95 (d, *J* = 4.0 Hz, 1H), 7.70–7.61 (m, 6H), 7.29 (t, *J* = 8.0 Hz, 1H), 7.20–7.18 (m, 1H), 6.24 (d, *J* = 8.8 Hz, 1H), 4.37 (s, 3H); ^13^C{^1^H} NMR (100 MHz, DMSO-d_6_) *δ* 139.0, 133.1, 131.6, 131.0, 129.85, 129.82, 127.7, 127.2, 125.4, 123.5, 123.3, 116.8, 116.4, 114.7, 114.2, 113.2, 112.6, 33.1; HRMS (ESI-QTOF) *m*/*z* [M + H]^+^ calcd for C_20_H_16_N_3_ 298.1339, found 298.1340.

## Conflicts of interest

There are no conflicts to declare.

## Supplementary Material

RA-010-D0RA01140A-s001

RA-010-D0RA01140A-s002
