# Supplementary material for: Facile approach to benzo[d]imidazole-pyrrolo[1,2-a]pyrazine hybrid structures through double cyclodehydration and aromatization and their unique optical properties with blue emission
Source: RSC Adv. 2020 Feb 18;10(12):7265–88. doi: 10.1039/d0ra01140a (PMC9049792; doi:10.1039/d0ra01140a)
Supplement: RA-010-D0RA01140A-s001 [file RA-010-D0RA01140A-s001.pdf]

Electronic Supplementary Material (ESI) for RSC Advances.
This journal is © The Royal Society of Chemistry 2020

Electronic Supplementary Information

Facile approach to benzo[*d*]imidazole-pyrrolo[1,2-*a*]pyrazine hybrid structures through double cyclodehydration and aromatization and their unique optical properties with blue emission

Gi Hun Bae,[a,‡] Suzi Kim,[b,‡] Na Keum Lee,[b] Anuradha Dagar,[a] Jeong Hwa Lee,[a] Jeeyeon Lee,*[,b] and Ikyon Kim*[,a]

[a] College of Pharmacy and Yonsei Institute of Pharmaceutical Sciences, Yonsei University 85 Songdogwahak-ro, Yeonsu-gu, Incheon, 21983, Republic of Korea

[b] College of Pharmacy, Research Institute of Pharmaceutical Sciences, Seoul National University, 1 Gwanak-ro, Gwanak-gu, Seoul, 08826, Republic of Korea

‡ These authors contributed equally.

* Corresponding authors.

Tel.: +82 32 749 4515; fax: +82 32 749 4105; e-mail: ikyonkim@yonsei.ac.kr

Tel.: +82 2 880 2471; fax: +82 2 884 8334; e-mail: jyleeut@snu.ac.kr

Table of Contents

Experimental section

^{1}H- and ^{13}C-NMR spectra of **8**	**S3-37**
^{1}H- and ^{13}C-NMR spectra of **9**	**S38**
^{1}H- and ^{13}C-NMR spectra of **11**	**S39**
^{1}H- and ^{13}C-NMR spectra of **13**	**S40-41**
^{1}H- and ^{13}C-NMR spectra of **14**	**S42**
Optical characterization	**S43**
Time-resolved fluorescence measurement	**S43**
Preparation and characterization of **8g** nanoparticles	**S43-44**
Image-Based Cell Screening	**S44**
Cytotoxicity Test	**S44-45**
Single Crystal X-ray Di□raction Studies	**S45**

Supplementary data

**Figure S1.**	Fluorescence spectra of **8a**, **8d**, **8g**, **8m** and **8u**	**S46**
**Figure S2.**	Fluorescence spectra of **8h**, **8q**, **8z** and **11**	**S47**
**Figure S3.**	Fluorescence spectra and photographs of **8c** and **8i** THF/Water mixture	**S47**
**Figure S4.**	Cytotoxicity of **8a**, **8h**, **8z**, and **11**	**S48**
**Table S1.**	Optical properties of 4BP and 5BP scaffolds	**S49**

Single crystal x-ray crystallographic data

**Table S2.**	Crystallographic data of **8c**	**S50**

^{1}H and ^{13}C NMR spectra of 8

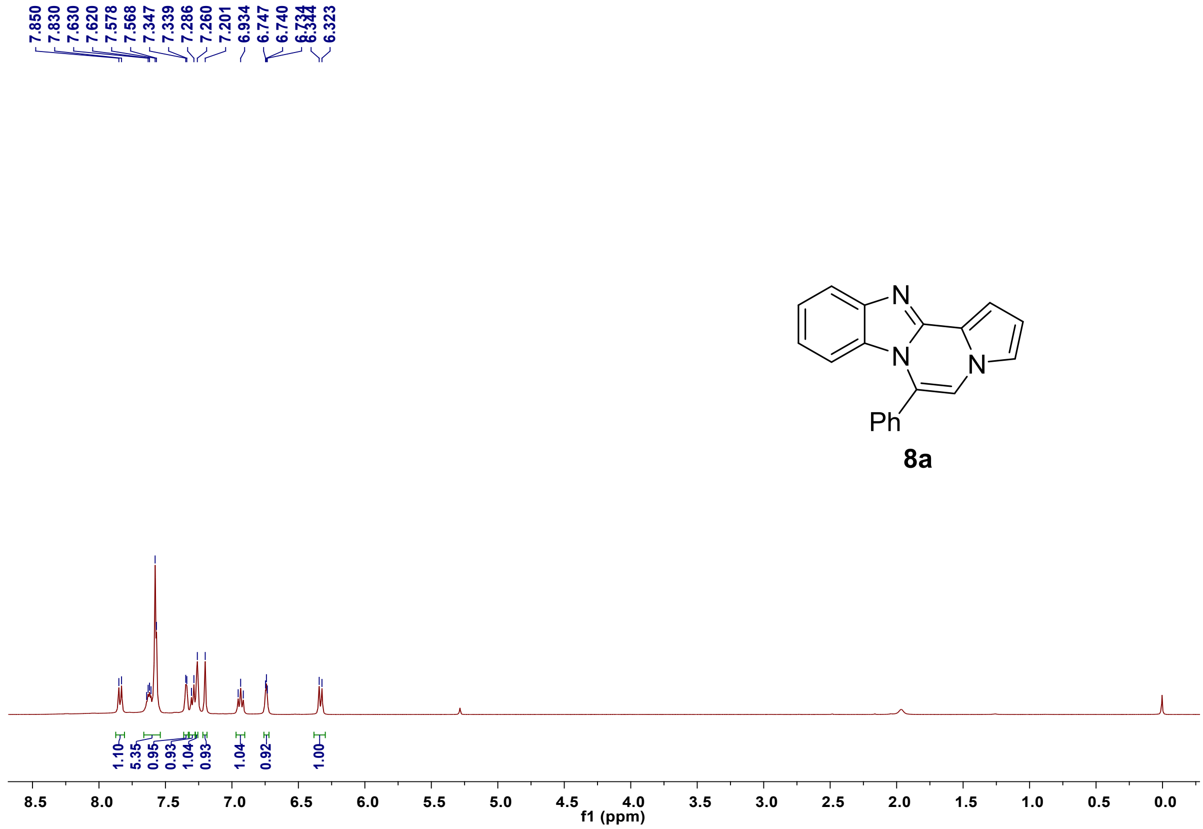

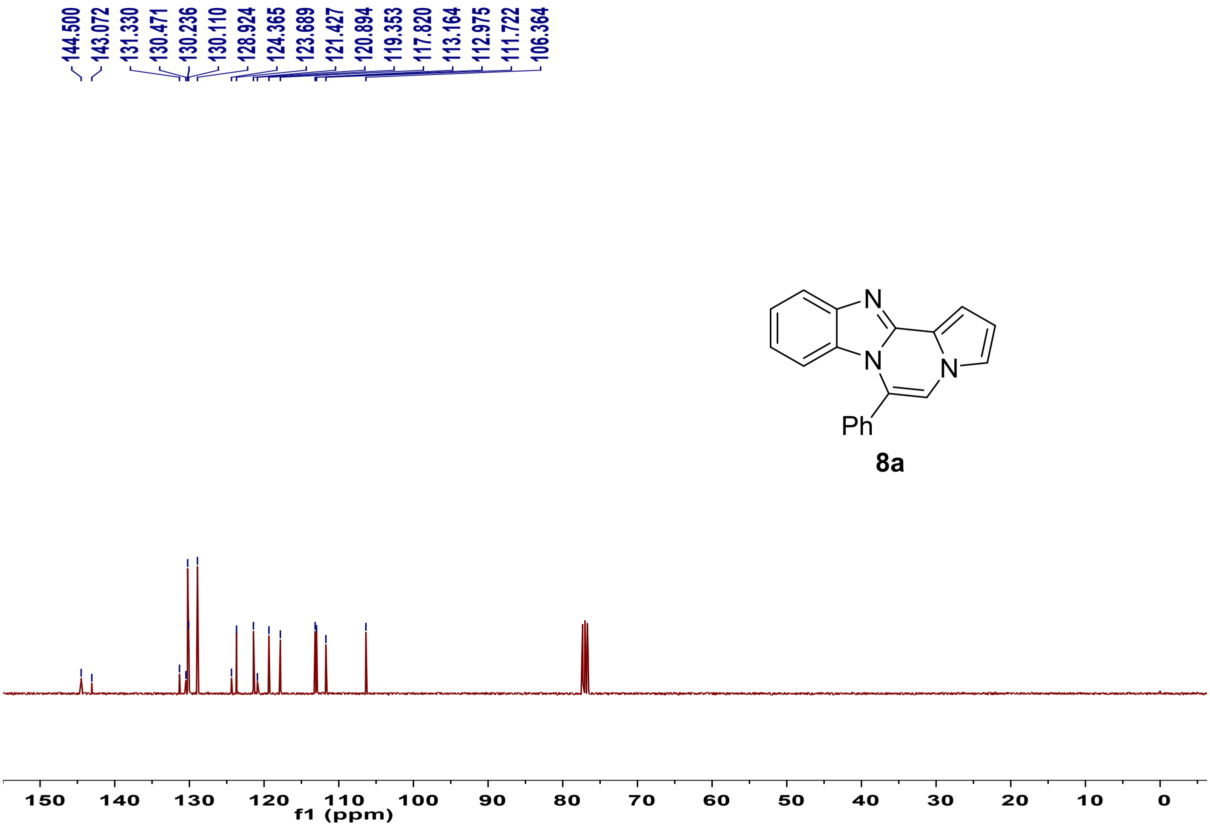

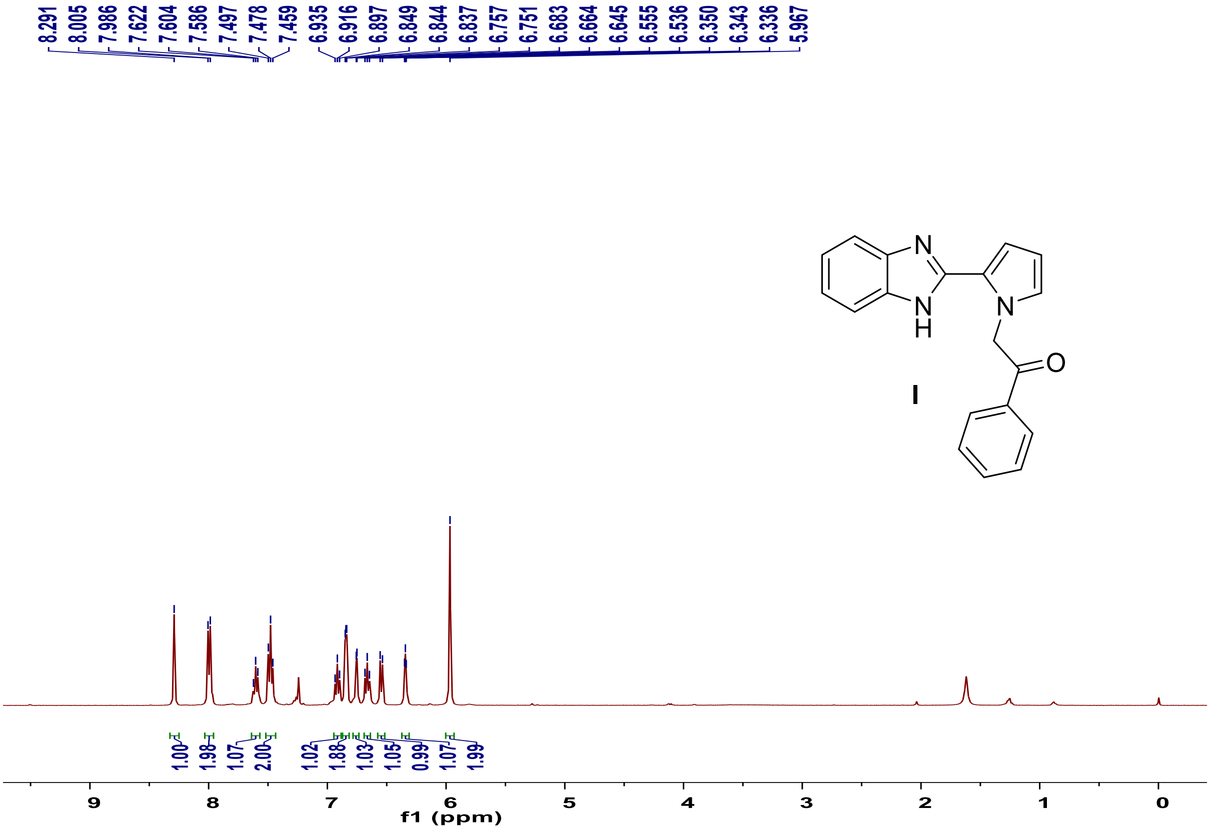
8.291
8.005
7.986
7.622
7.604
7.586
7.497
7.478
7.459
6.935
6.916
6.897
6.849
6.844
6.837
6.757
6.751
6.683
6.664
6.645
6.555
6.536
6.350
6.343
6.336
5.967
N
N
H
N
O
I
1.00
1.98
1.07
2.00
1.02
1.88
1.03
1.05
0.99
1.07
1.99
f1 (ppm)

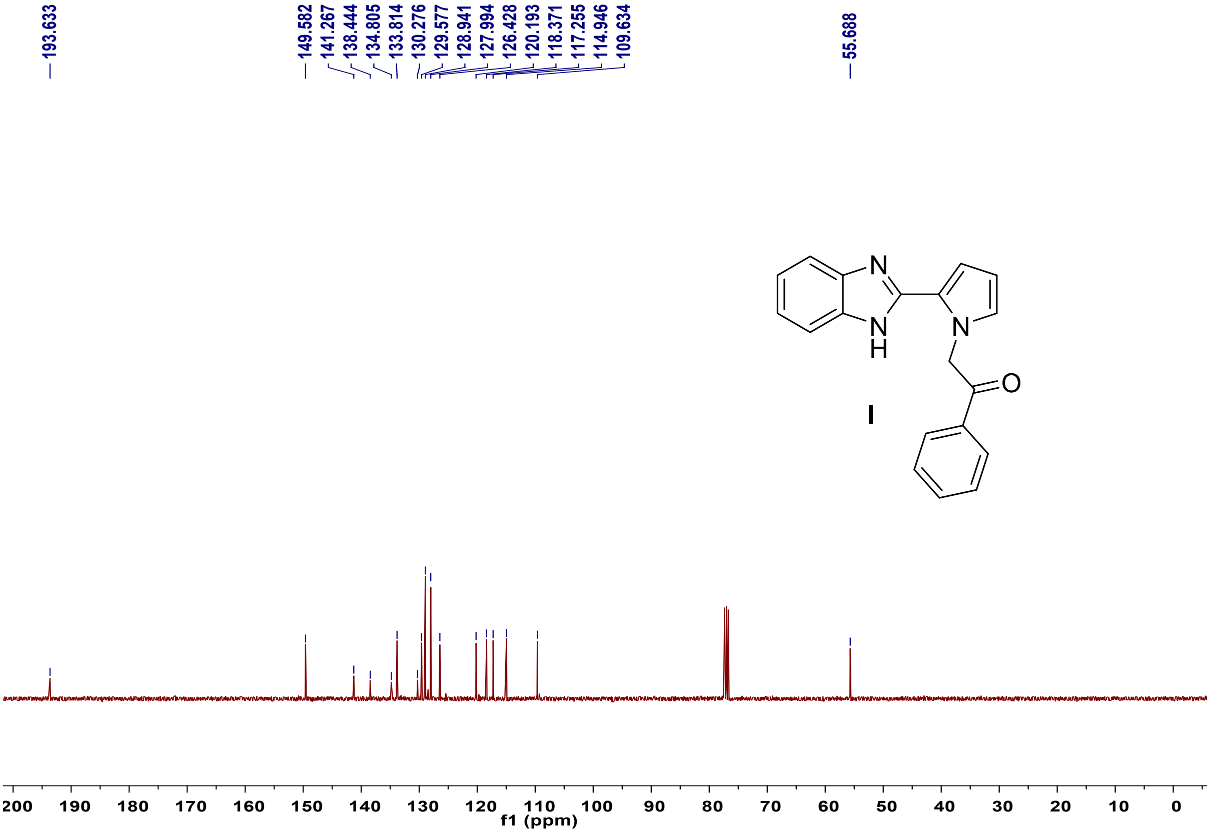
193.633
149.582
141.267
138.444
134.805
133.814
130.276
129.577
128.941
127.994
126.428
120.193
118.371
117.255
114.946
109.634
55.688
N
N
H
N
O
I
f1 (ppm)

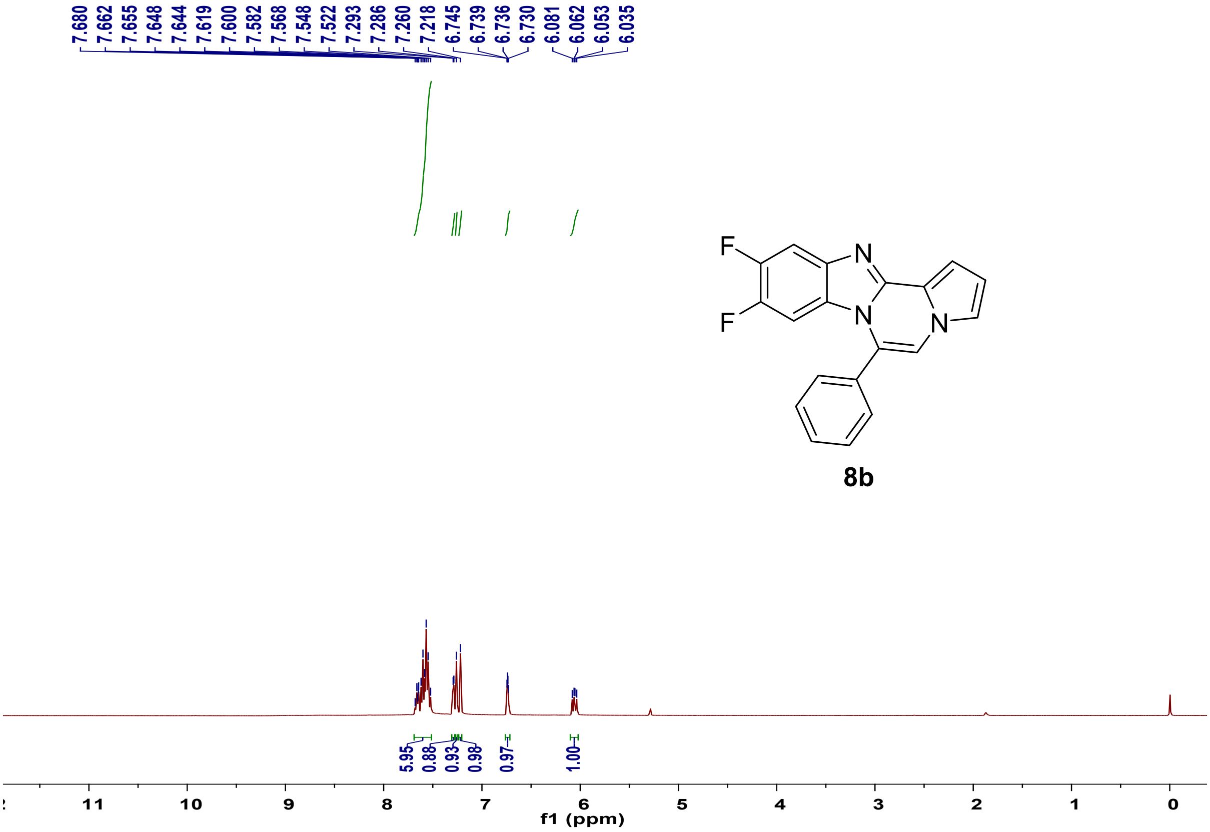

8b

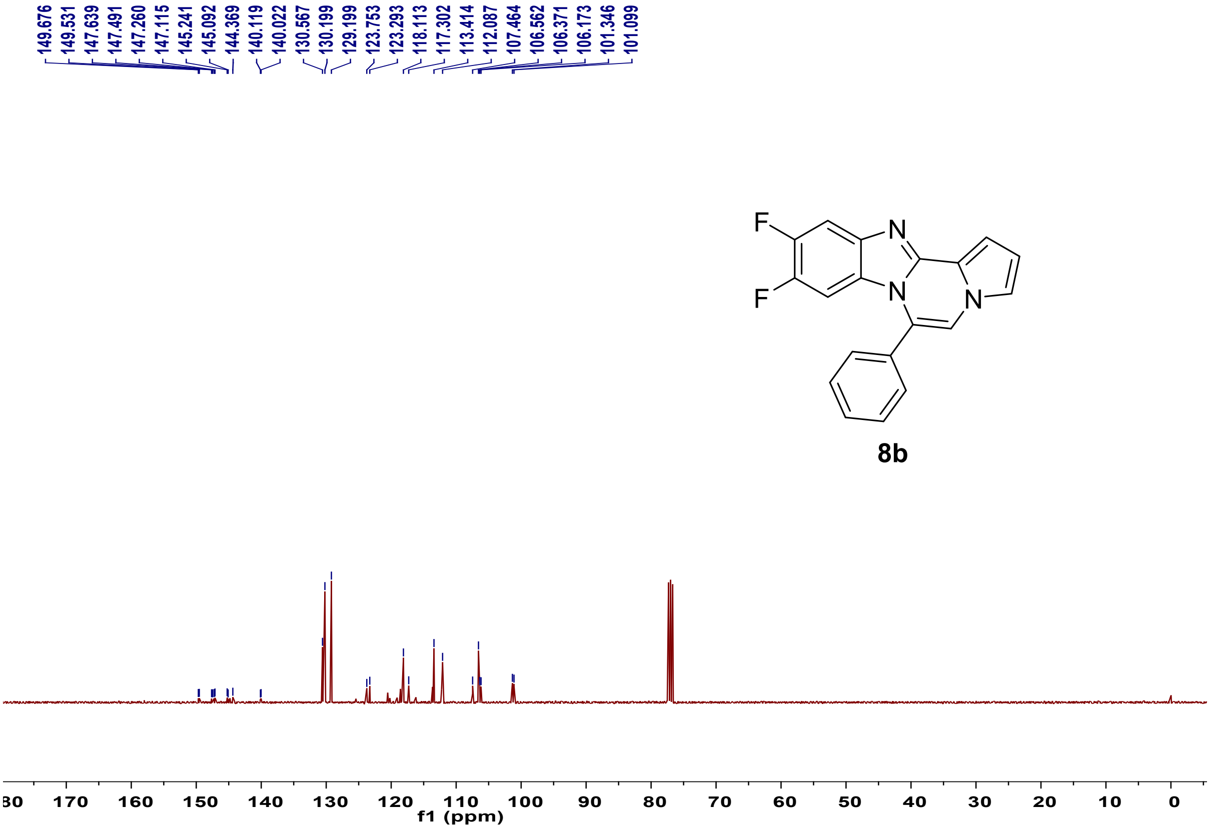

8b

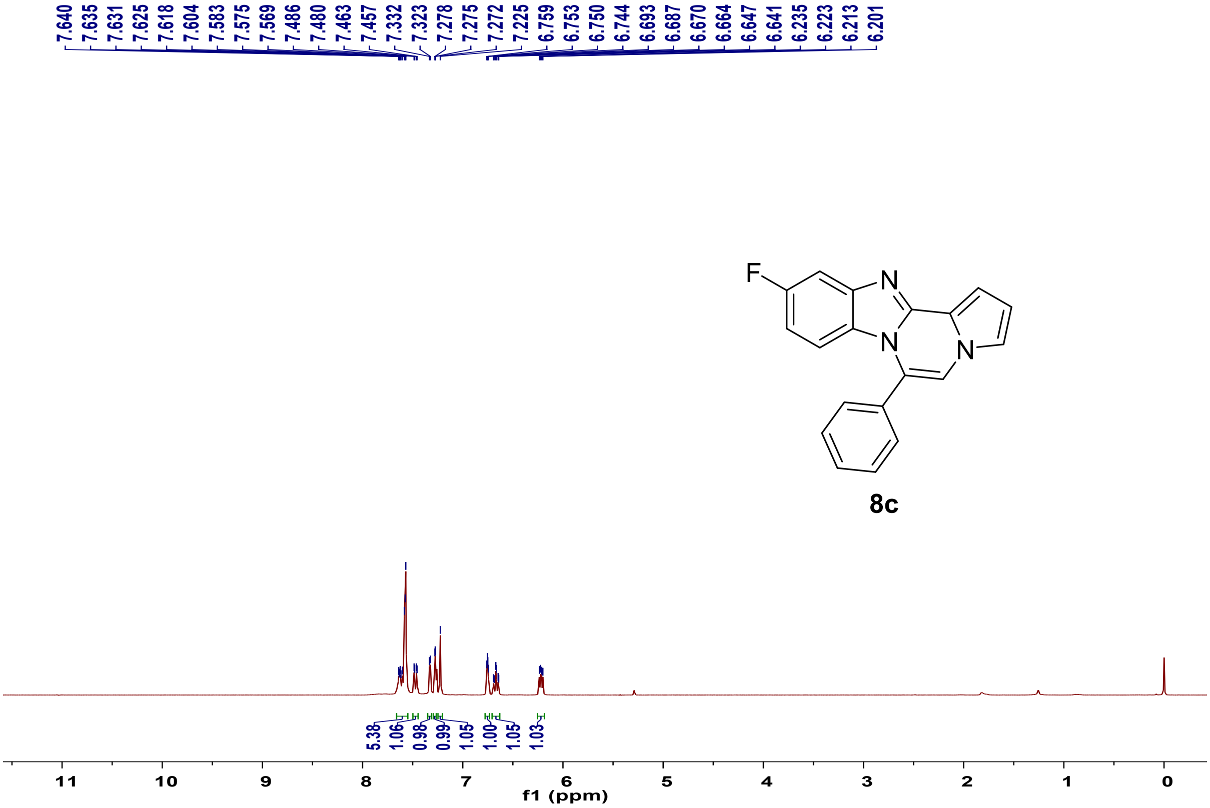

7.640
7.635
7.631
7.625
7.618
7.604
7.583
7.575
7.569
7.486
7.480
7.463
7.457
7.332
7.323
7.278
7.275
7.272
7.225
6.759
6.753
6.750
6.744
6.693
6.687
6.670
6.664
6.647
6.641
6.235
6.223
6.213
6.201
F
N
N
N
5.38
1.06
0.98
0.99
1.05
1.00
1.05
1.03
11 10 9 8 7 6 5 4 3 2 1 0
f1 (ppm)

8c

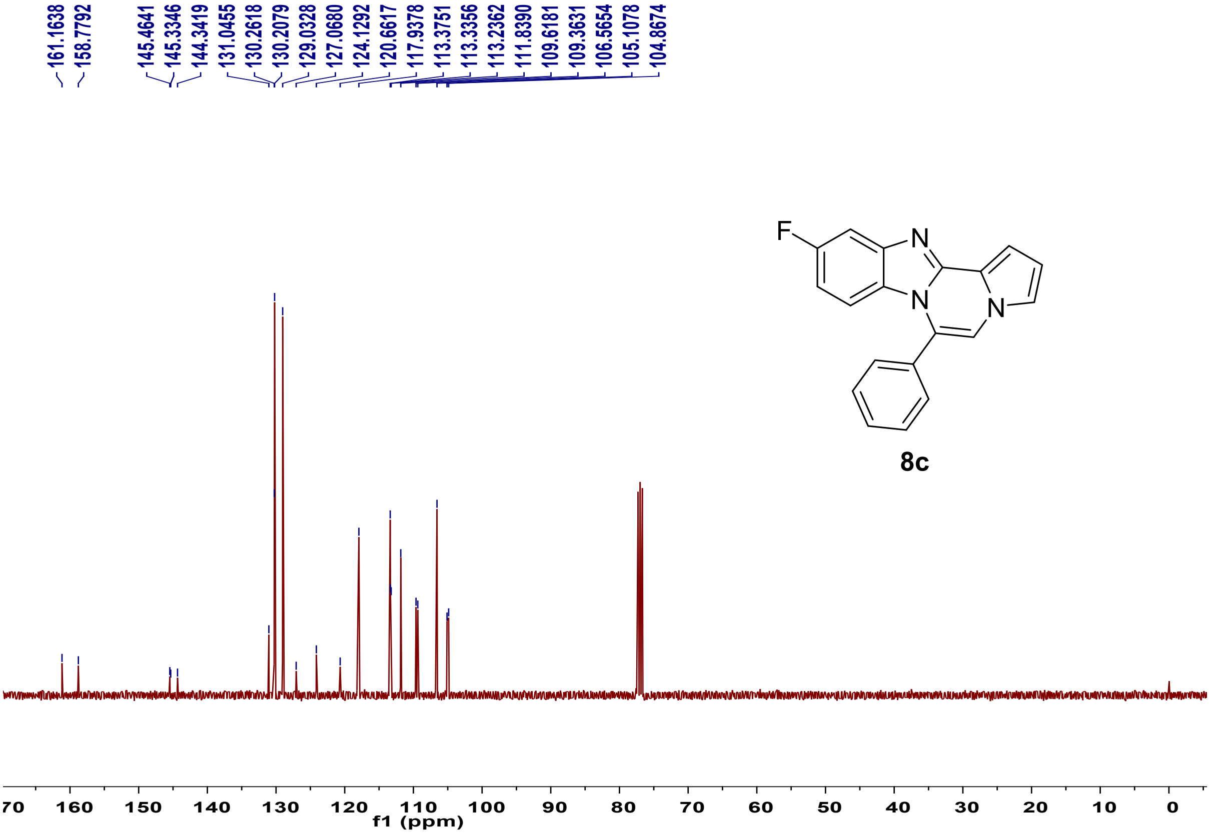

161.1638
158.7792
145.4641
145.3346
144.3419
131.0455
130.2618
130.2079
129.0328
127.0680
124.1292
120.6617
117.9378
113.3751
113.3356
113.2362
111.8390
109.6181
109.3631
106.5654
105.1078
104.8674
F
N
N
N
70 160 150 140 130 120 110 100 90 80 70 60 50 40 30 20 10 0
f1 (ppm)

8c

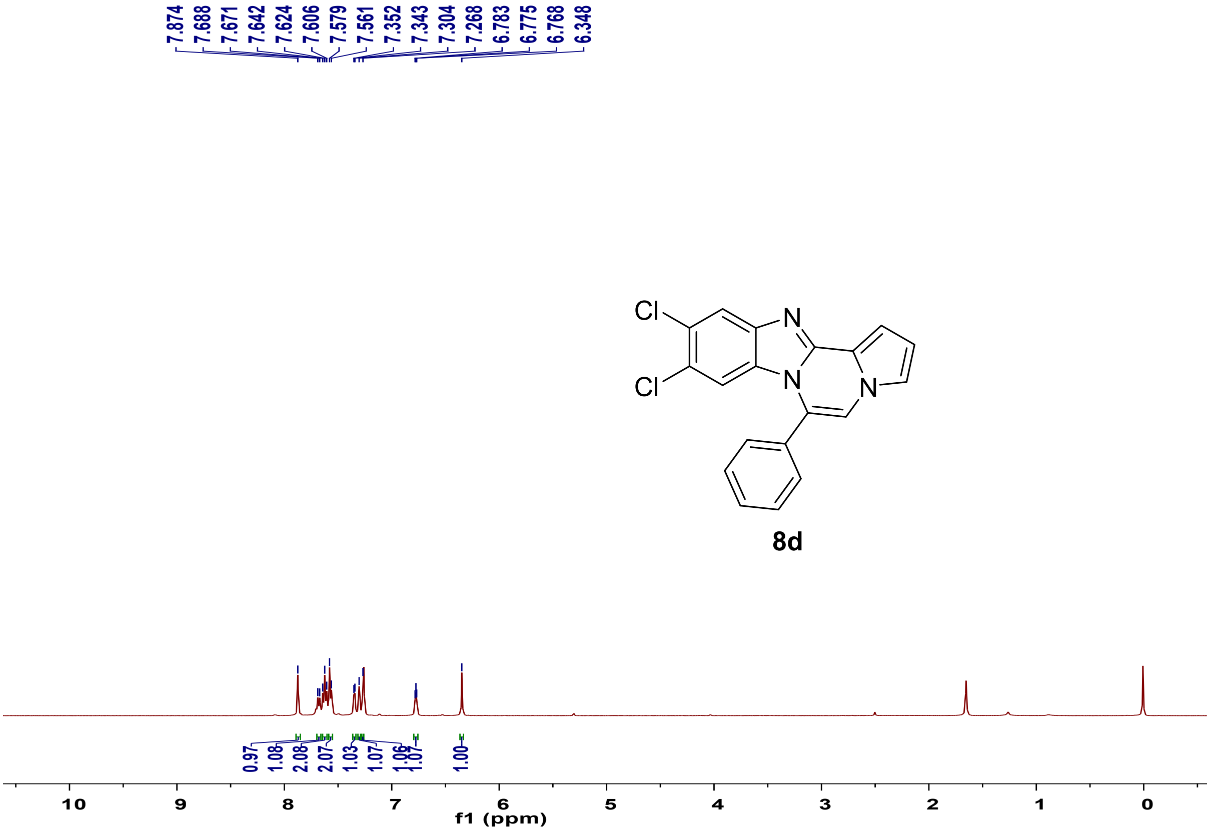
7.874
7.688
7.671
7.642
7.624
7.606
7.579
7.561
7.352
7.343
7.304
7.268
6.783
6.775
6.768
6.348
Cl
Cl
N
N
N
8d
0.97
1.08
2.08
2.07
1.03
1.07
1.06
1.07
1.00
10 9 8 7 6 5 4 3 2 1 0
f1 (ppm)

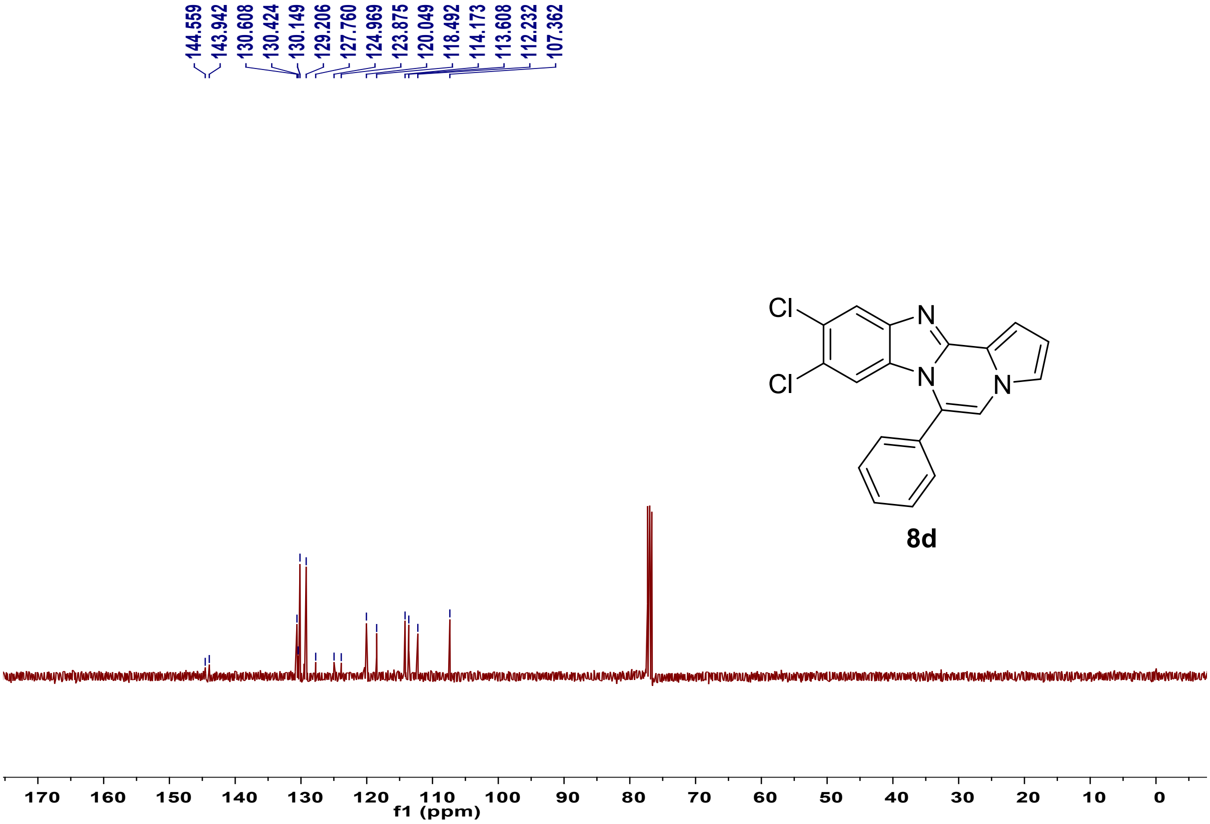
144.559
143.942
130.608
130.424
130.149
129.206
127.760
124.969
123.875
120.049
118.492
114.173
113.608
112.232
107.362
Cl
Cl
N
N
N
8d
170 160 150 140 130 120 110 100 90 80 70 60 50 40 30 20 10 0
f1 (ppm)

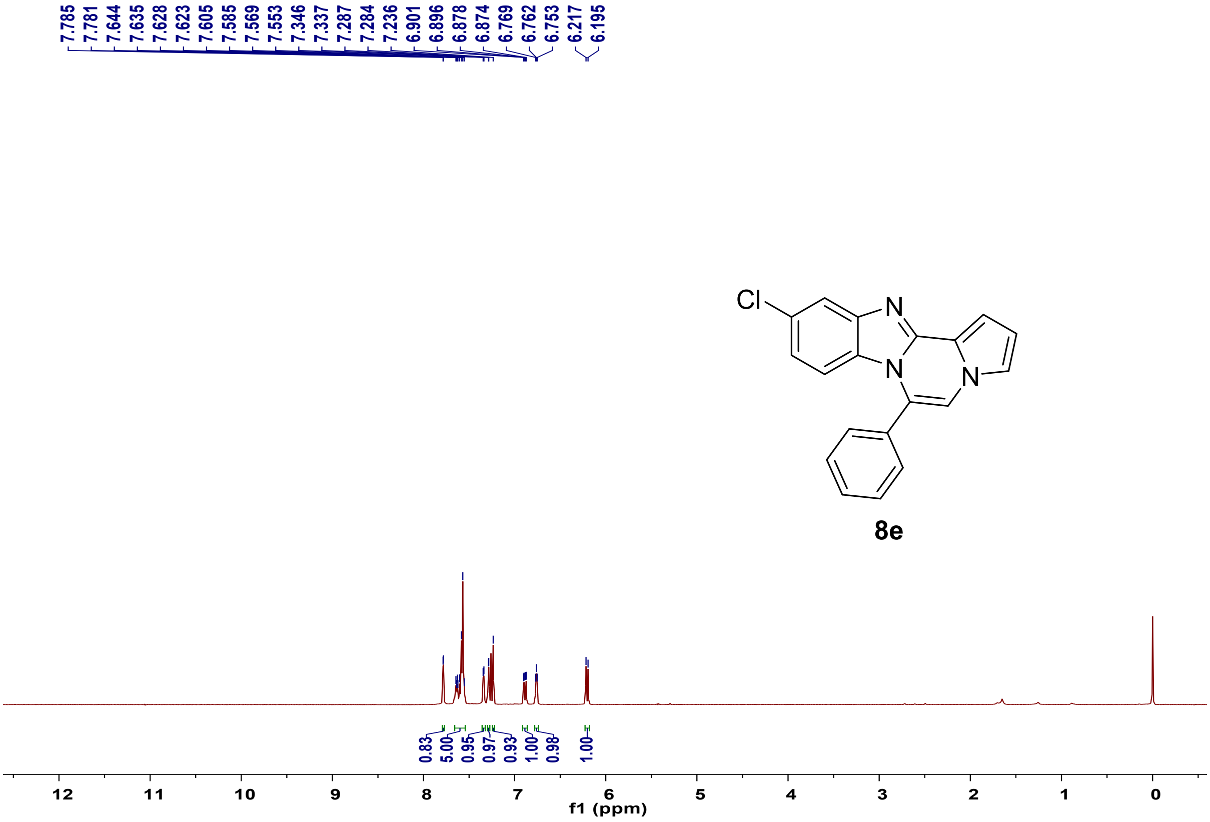
7.785
7.781
7.644
7.635
7.628
7.623
7.605
7.585
7.569
7.553
7.346
7.337
7.287
7.284
7.236
6.901
6.896
6.878
6.874
6.769
6.762
6.753
6.217
6.195
Cl
N
N
N
8e
0.83
5.00
0.95
0.97
0.93
1.00
0.98
1.00
f1 (ppm)

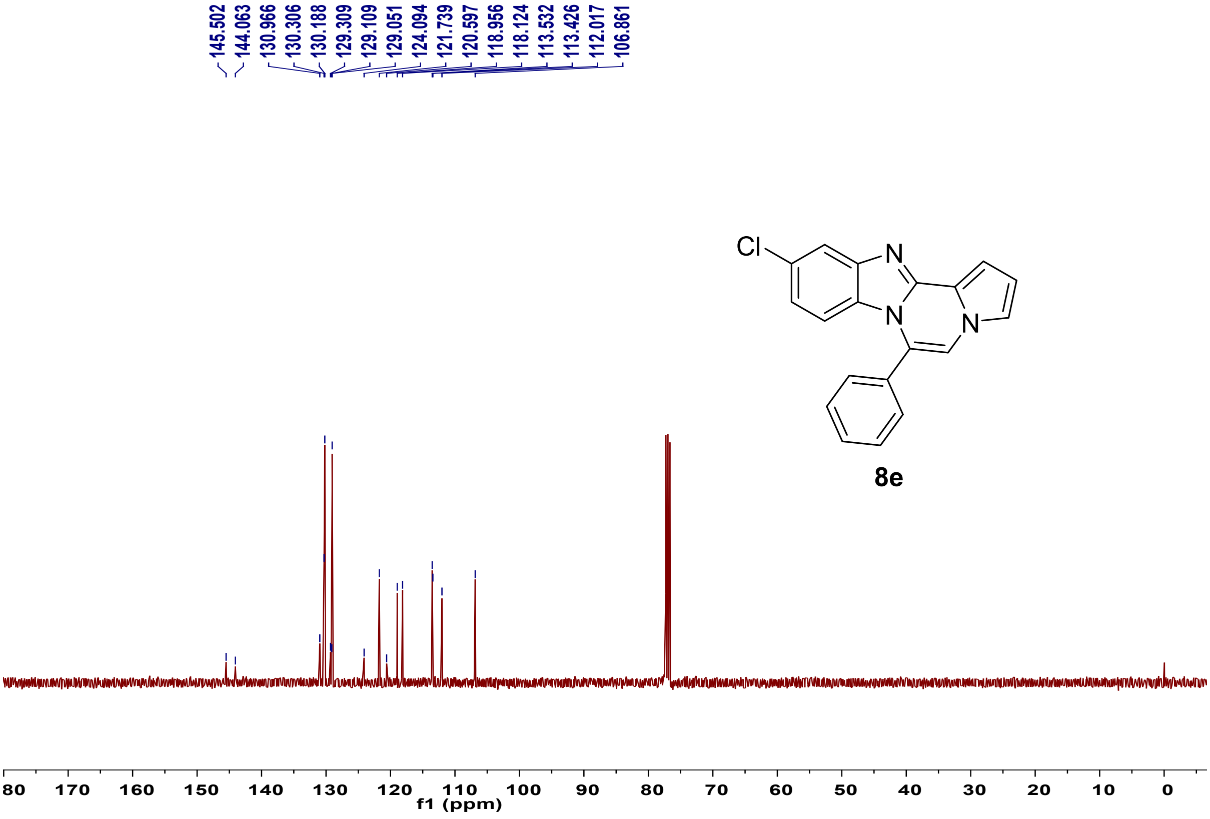
145.502
144.063
130.966
130.306
130.188
129.309
129.109
129.051
124.094
121.739
120.597
118.956
118.124
113.532
113.426
112.017
106.861
Cl
N
N
N
8e
f1 (ppm)

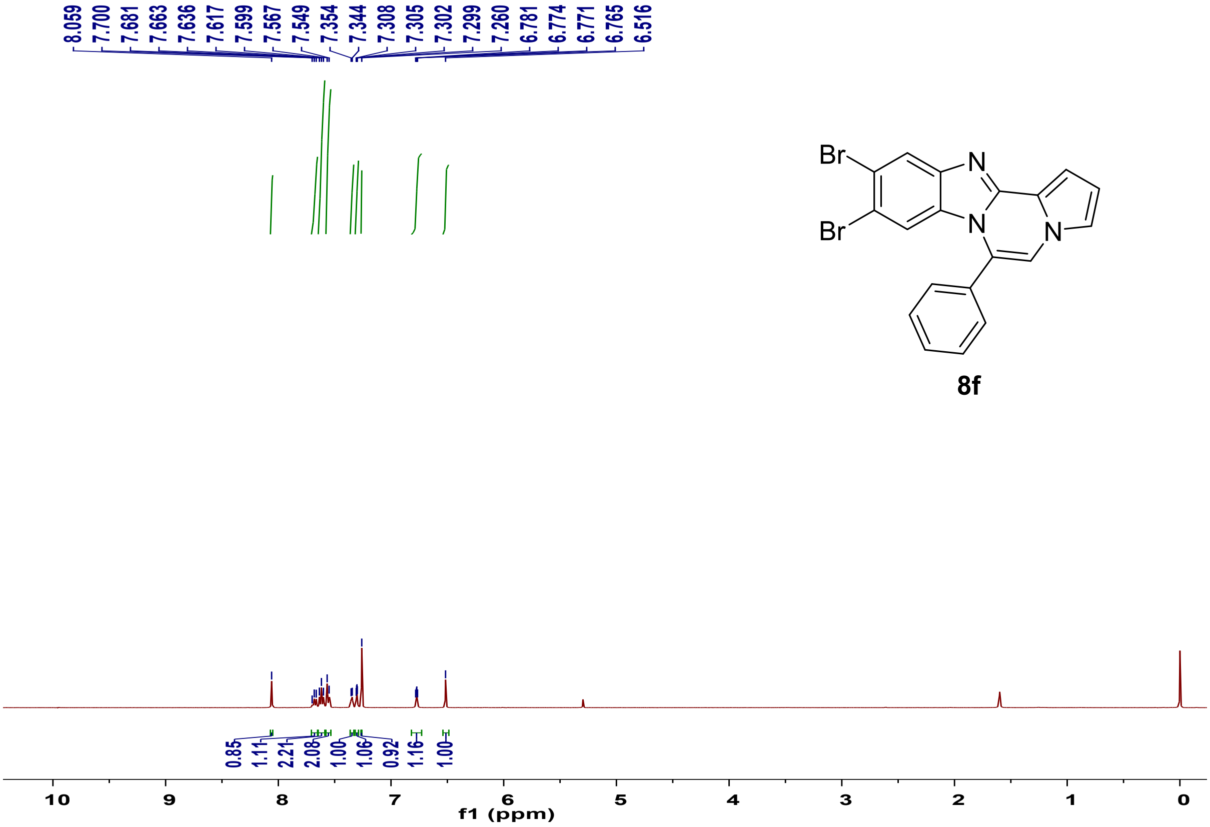
8.059
7.700
7.681
7.663
7.636
7.617
7.599
7.567
7.549
7.354
7.344
7.308
7.305
7.302
7.299
7.260
6.781
6.774
6.771
6.765
6.516
0.85
1.11
2.21
2.08
1.00
1.06
0.92
1.16
1.00
f1 (ppm)
Br
Br
N
N
N

8f

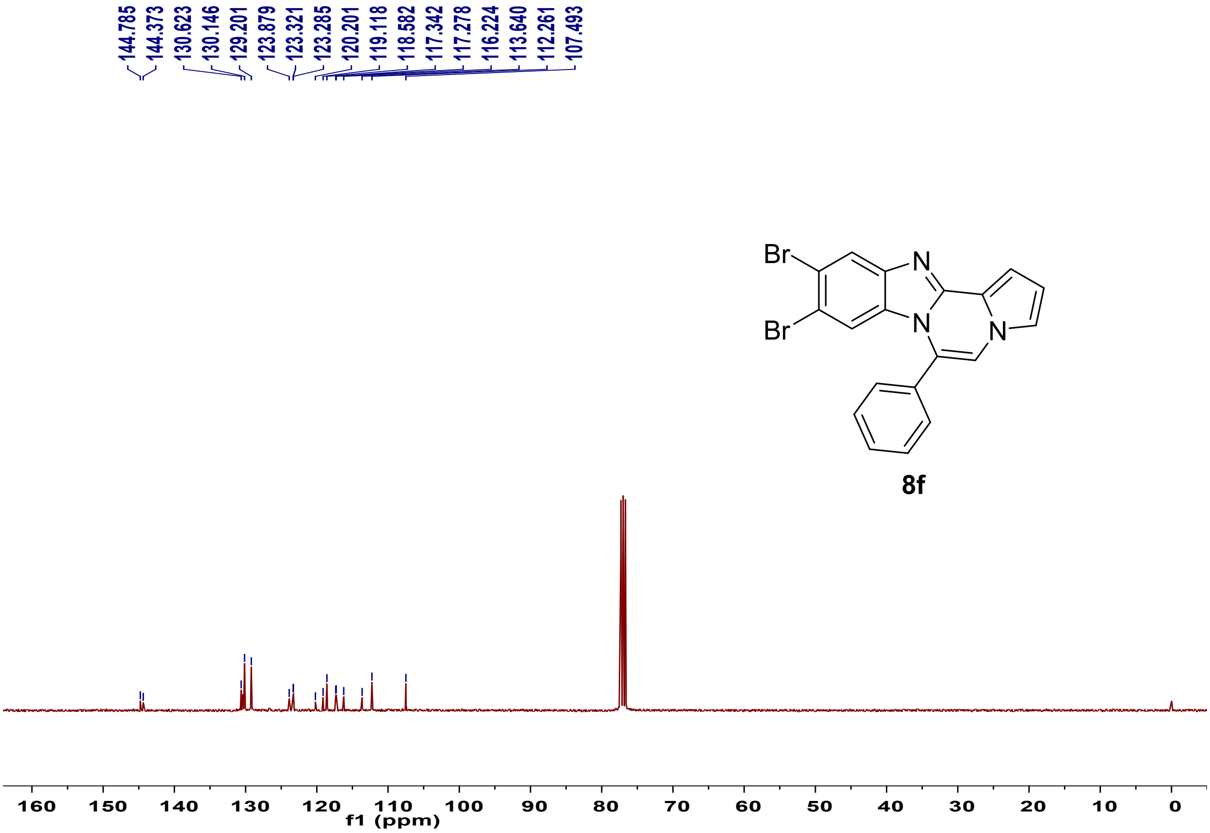
144.785
144.373
130.623
130.146
129.201
123.879
123.321
123.285
120.201
119.118
118.582
117.342
117.278
116.224
113.640
112.261
107.493
f1 (ppm)
Br
Br
N
N
N

8f

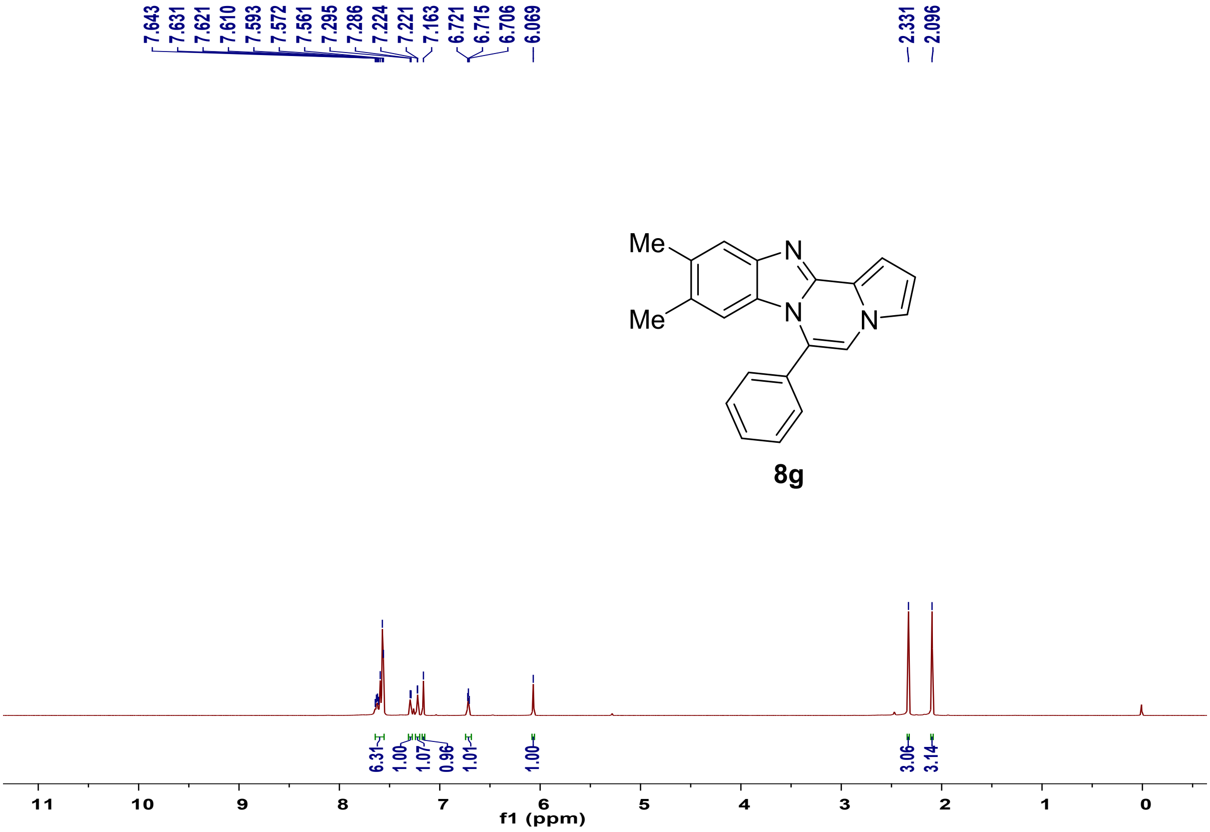
7.643
7.631
7.621
7.610
7.593
7.572
7.561
7.295
7.286
7.224
7.221
7.163
6.721
6.715
6.706
6.069
2.331
2.096
Me
Me
N
N
N
6.31
1.00
1.07
0.96
1.01
1.00
3.06
3.14
11
10
9
8
7
6
5
4
3
2
1
0
f1 (ppm)

8g

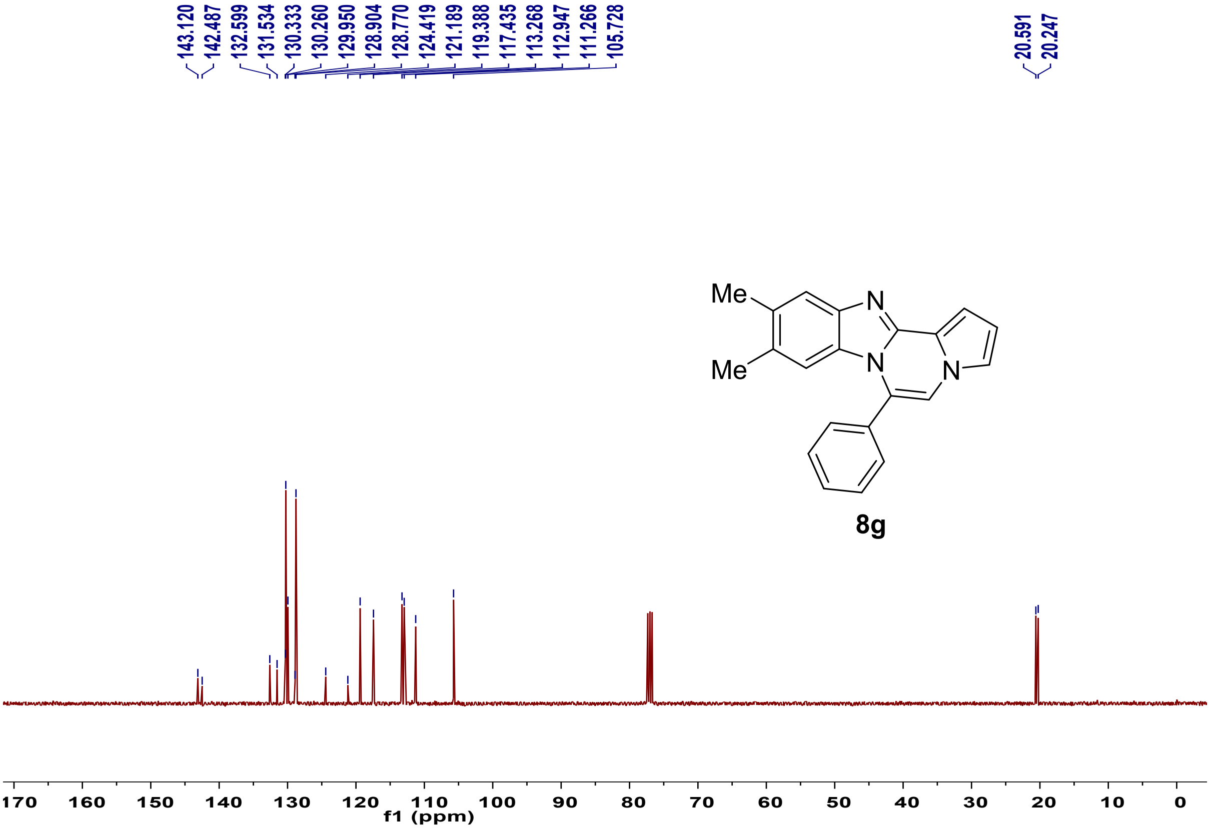
143.120
142.487
132.599
131.534
130.333
130.260
129.950
128.904
128.770
124.419
121.189
119.388
117.435
113.268
112.947
111.266
105.728
20.591
20.247
Me
Me
N
N
N
170
160
150
140
130
120
110
100
90
80
70
60
50
40
30
20
10
0
f1 (ppm)

8g

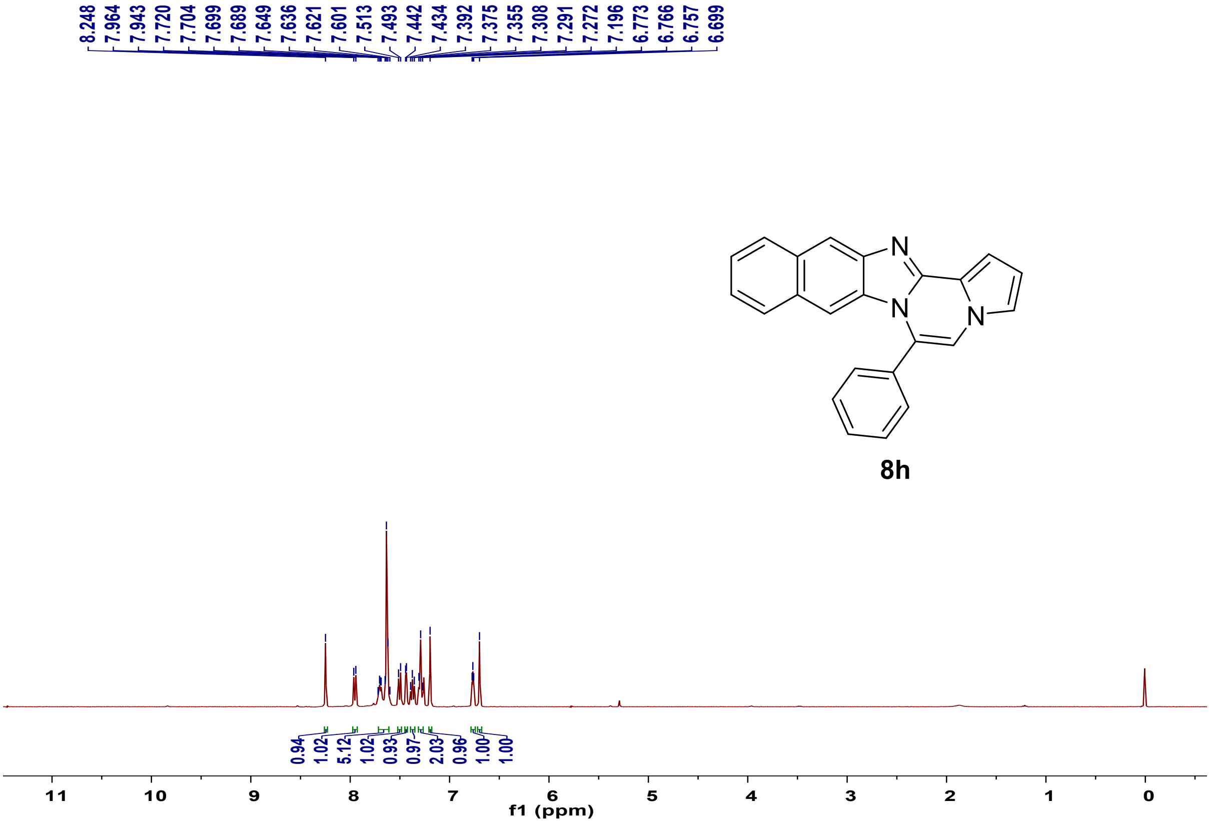

8.248
7.964
7.943
7.720
7.704
7.699
7.689
7.649
7.636
7.621
7.601
7.513
7.493
7.442
7.434
7.392
7.375
7.355
7.308
7.291
7.272
7.196
6.773
6.766
6.757
6.699
N
N
N
0.94
1.02
5.12
1.02
0.93
0.97
2.03
0.96
1.00
1.00
f1 (ppm)

8h

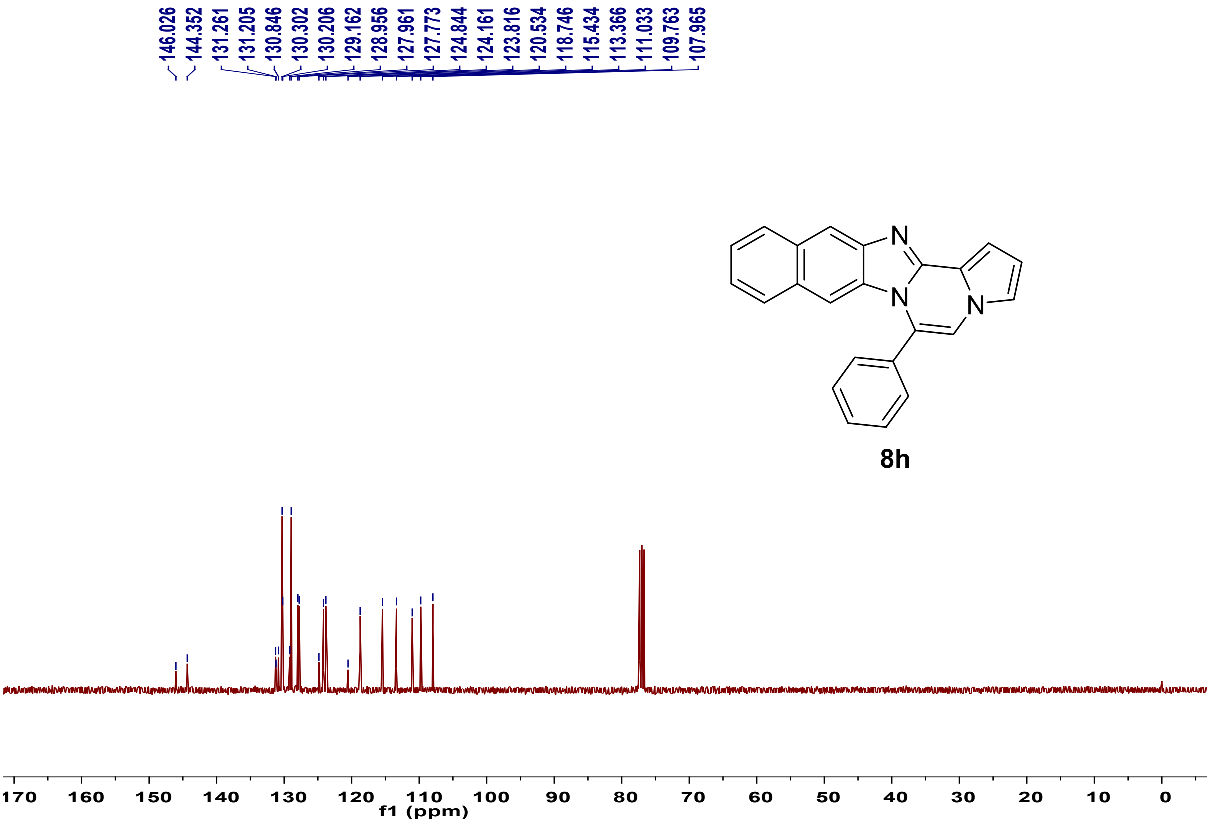

146.026
144.352
131.261
131.205
130.846
130.302
130.206
129.162
128.956
127.961
127.773
124.844
124.161
123.816
120.534
118.746
115.434
113.366
111.033
109.763
107.965
N
N
N
f1 (ppm)

8h

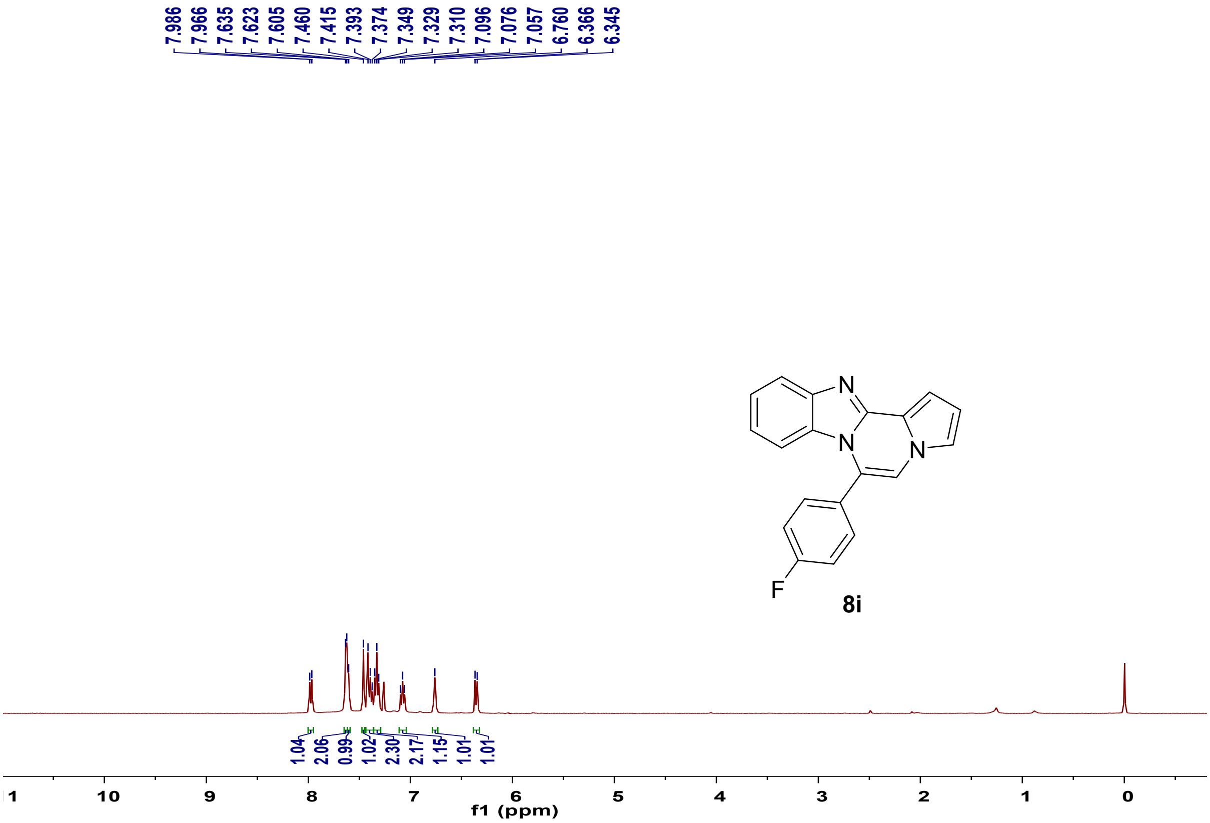
7.986
7.966
7.635
7.623
7.605
7.460
7.415
7.393
7.374
7.349
7.329
7.310
7.096
7.076
7.057
6.760
6.366
6.345
N
N
N
F
8i
1.04
2.06
0.99
1.02
2.30
2.17
1.15
1.01
1.01
f1 (ppm)

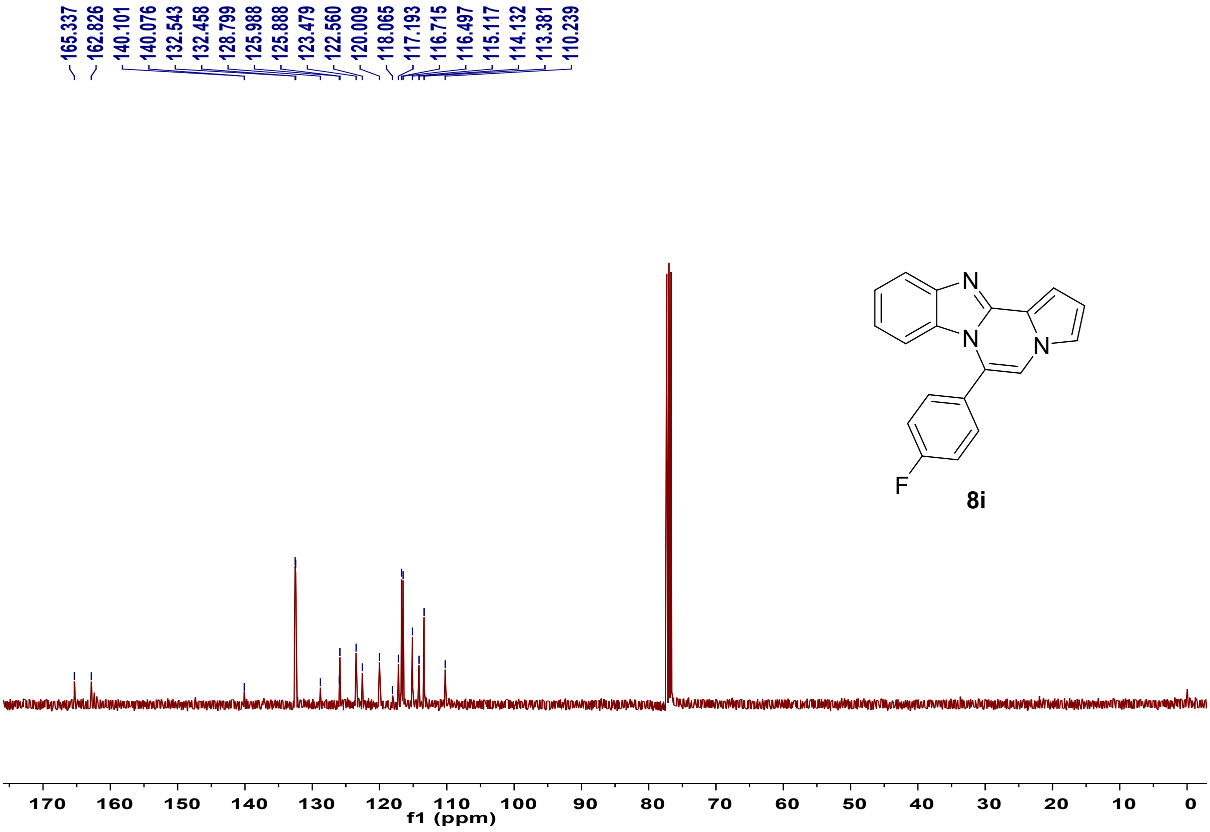
165.337
162.826
140.101
140.076
132.543
132.458
128.799
125.988
125.888
123.479
122.560
120.009
118.065
117.193
116.715
116.497
115.117
114.132
113.381
110.239
N
N
N
F
8i
f1 (ppm)

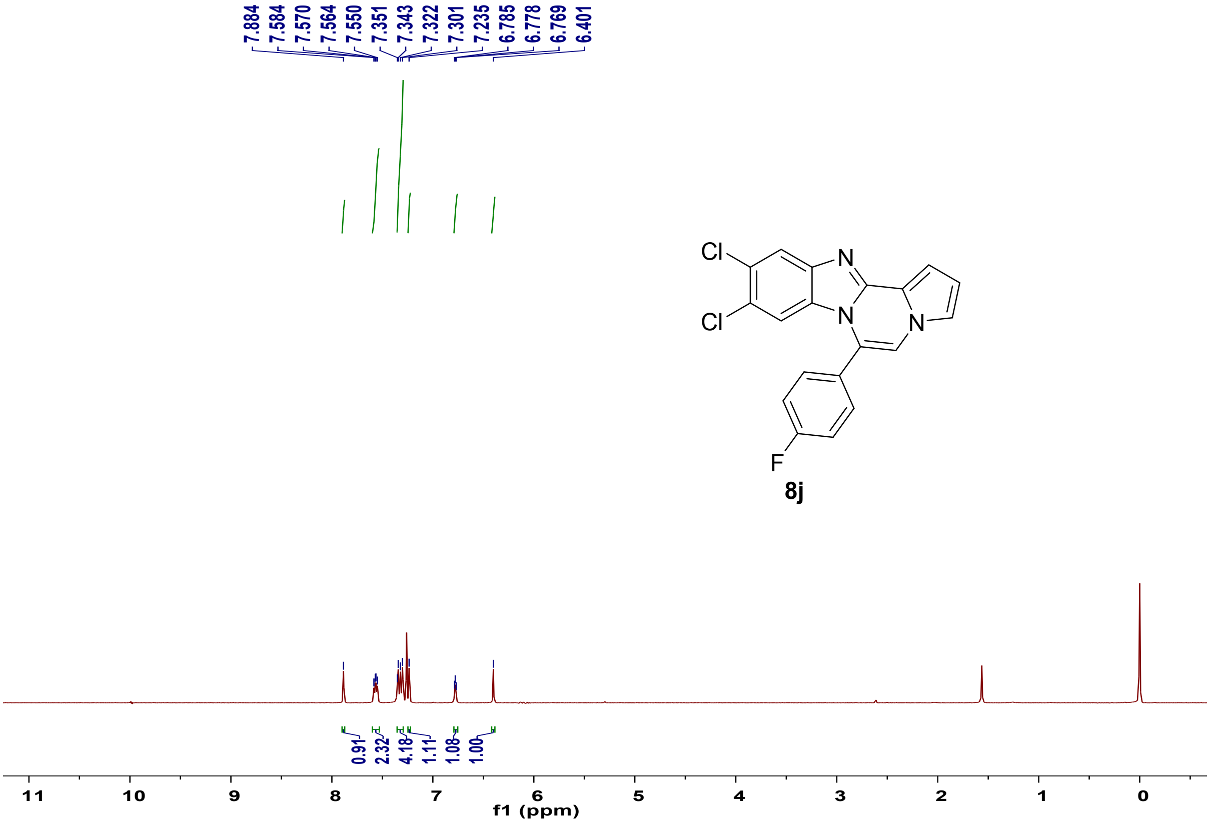
7.884
7.584
7.570
7.564
7.550
7.351
7.343
7.322
7.301
7.235
6.785
6.778
6.769
6.401
Cl
Cl
N
N
N
F
8j
0.91
2.32
4.18
1.11
1.08
1.00
11 10 9 8 7 6 5 4 3 2 1 0
f1 (ppm)

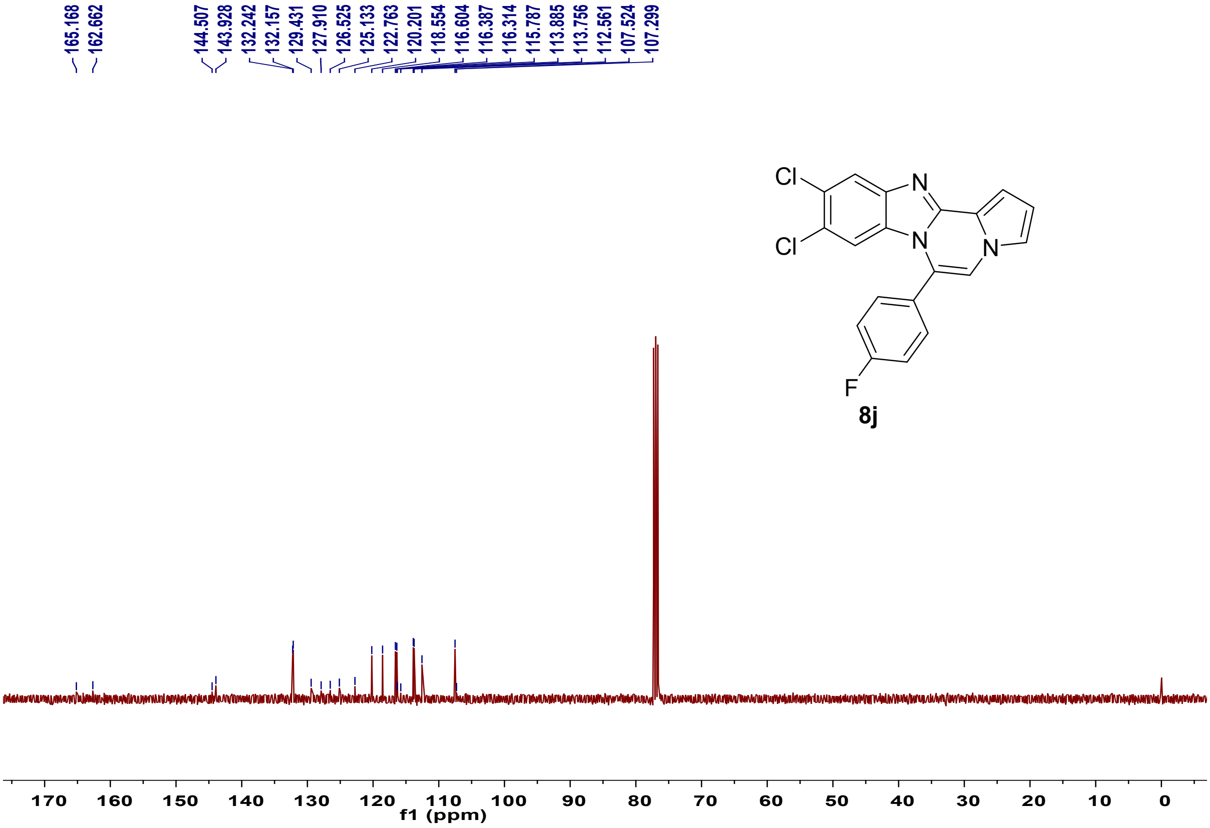
165.168
162.662
144.507
143.928
132.242
132.157
129.431
127.910
126.525
125.133
122.763
120.201
118.554
116.604
116.387
116.314
115.787
113.885
113.756
112.561
107.524
107.299
Cl
Cl
N
N
N
F
8j
170 160 150 140 130 120 110 100 90 80 70 60 50 40 30 20 10 0
f1 (ppm)

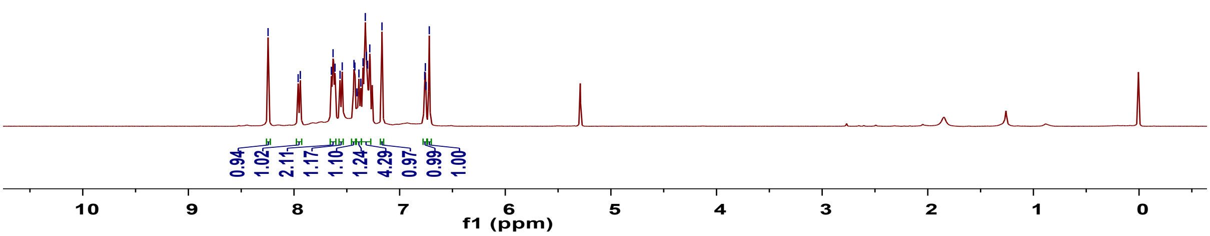
8.246
7.961
7.940
7.646
7.630
7.612
7.564
7.543
7.432
7.424
7.404
7.385
7.367
7.346
7.325
7.315
7.304
7.283
7.167
6.763
6.757
6.750
6.719
0.94
1.02
2.11
1.17
1.10
1.24
4.29
0.97
0.99
1.00
10 9 8 7 6 5 4 3 2 1 0
f1 (ppm)

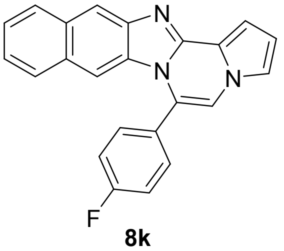
N
N
N
F

8k

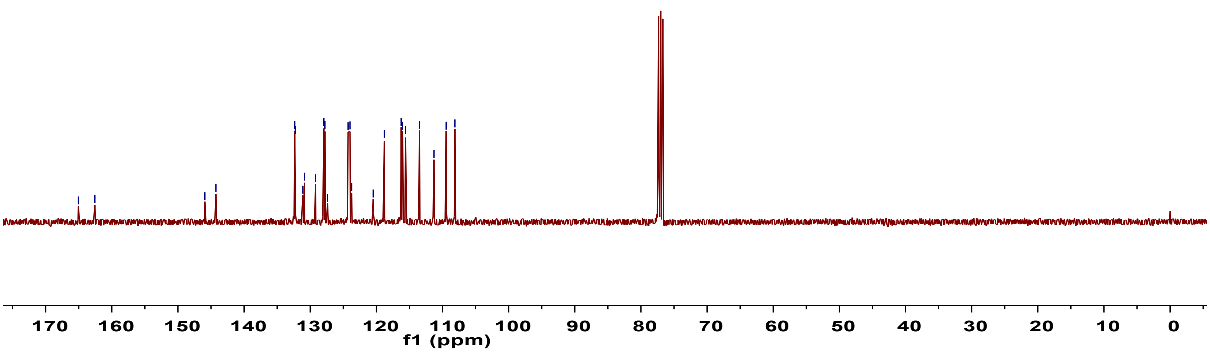
165.056
162.561
145.930
144.256
132.341
132.257
131.105
130.869
129.195
127.938
127.781
127.374
124.279
123.988
123.736
120.476
118.793
116.255
116.038
115.597
113.475
111.291
109.451
108.124
170 160 150 140 130 120 110 100 90 80 70 60 50 40 30 20 10 0
f1 (ppm)

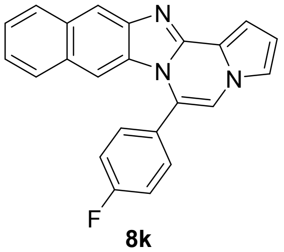
N
N
N
F

8k

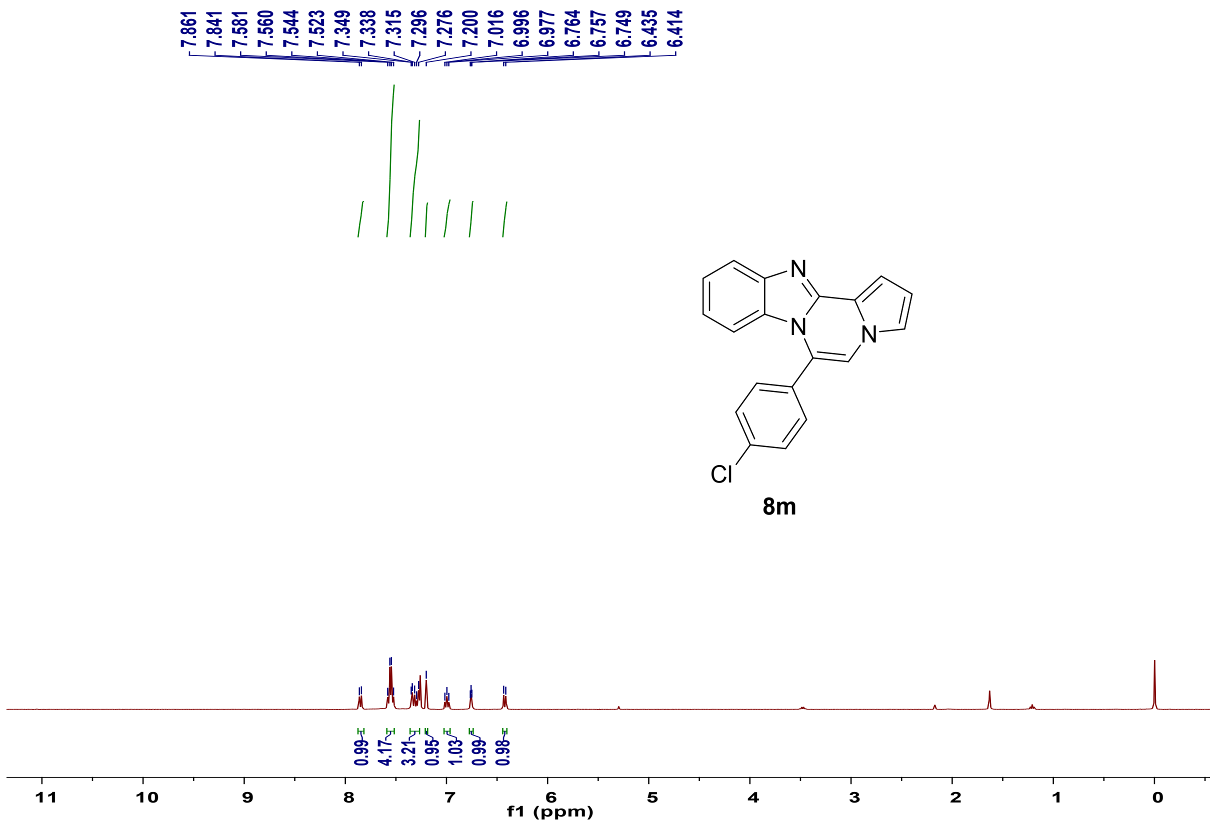
7.861
7.841
7.581
7.560
7.544
7.523
7.349
7.338
7.315
7.296
7.276
7.200
7.016
6.996
6.977
6.764
6.757
6.749
6.435
6.414
8m
0.99
4.17
3.21
0.95
1.03
0.99
0.98
11 10 9 8 7 6 5 4 3 2 1 0
f1 (ppm)

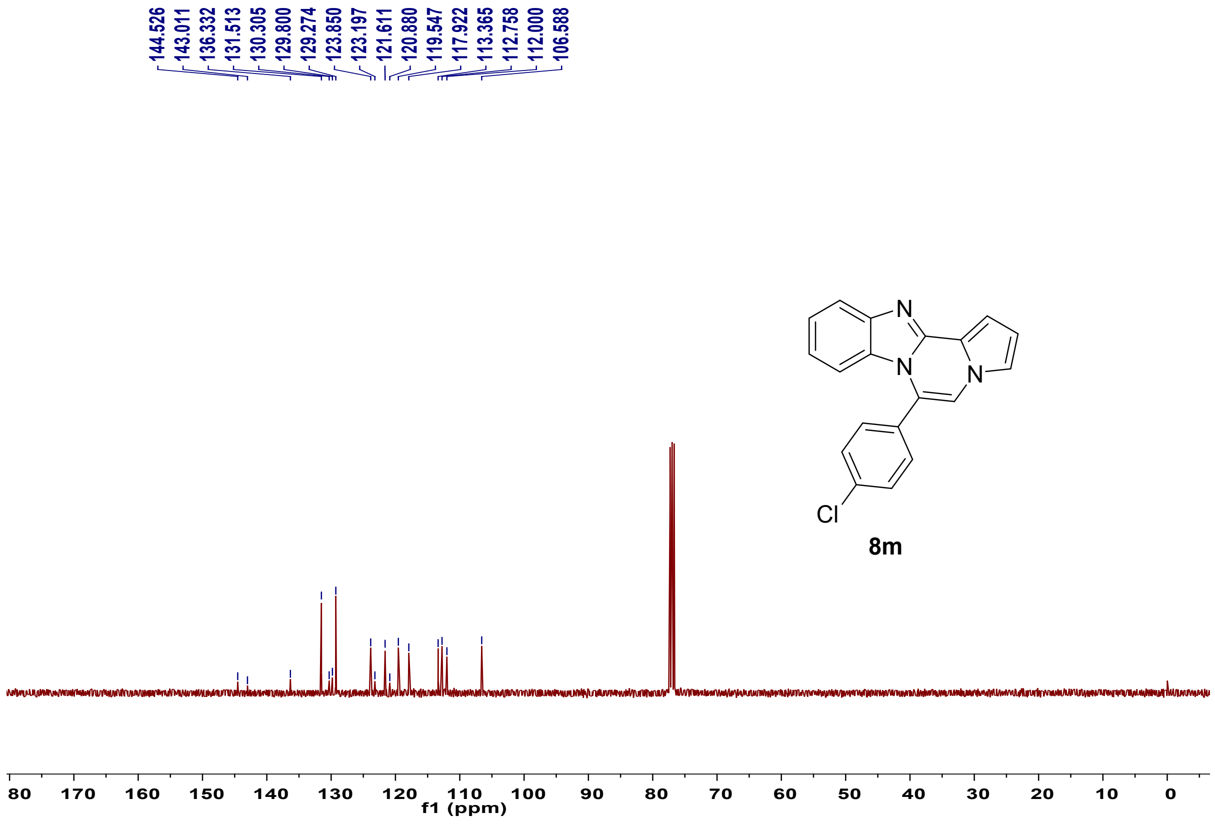
144.526
143.011
136.332
131.513
130.305
129.800
129.274
123.850
123.197
121.611
120.880
119.547
117.922
113.365
112.758
112.000
106.588
8m
80 170 160 150 140 130 120 110 100 90 80 70 60 50 40 30 20 10 0
f1 (ppm)

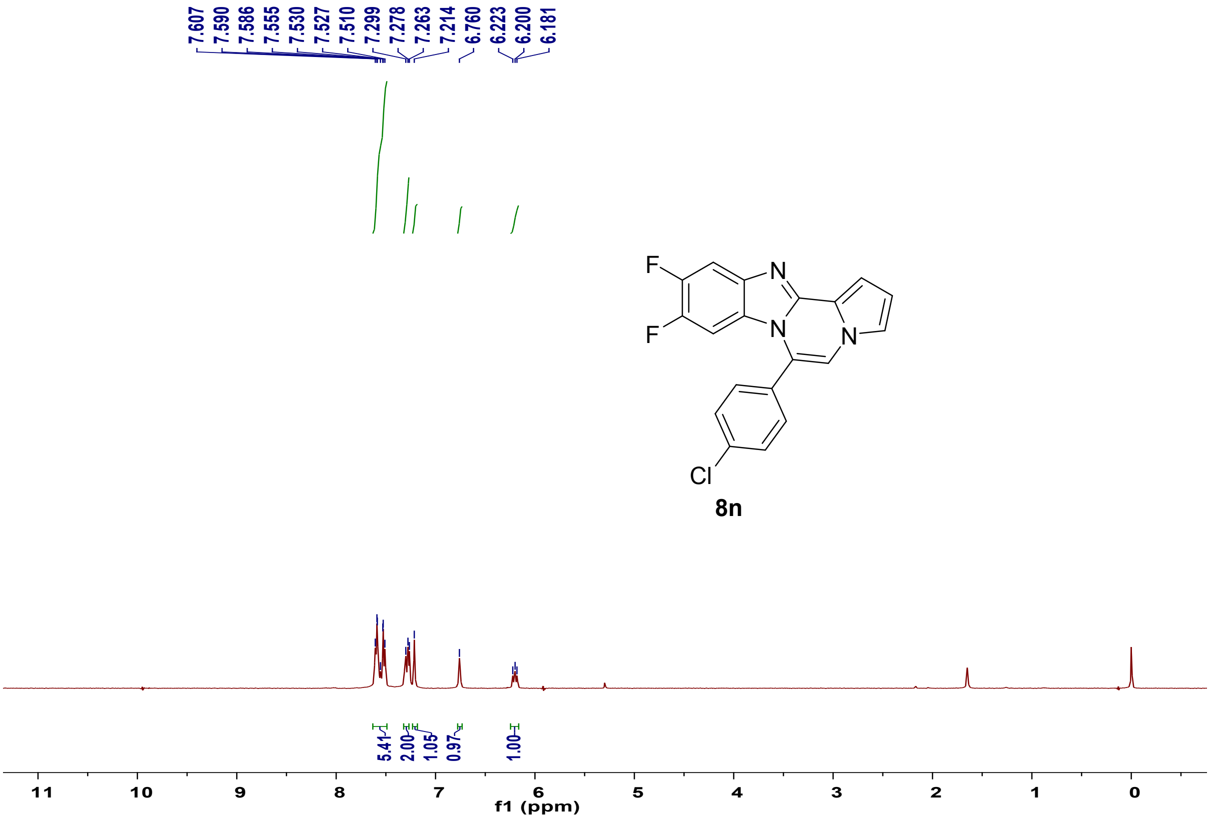
7.607
7.590
7.586
7.555
7.530
7.527
7.510
7.299
7.278
7.263
7.214
6.760
6.223
6.200
6.181
F
F
N
N
N
Cl
8n
5.41
2.00
1.05
0.97
1.00
f1 (ppm)

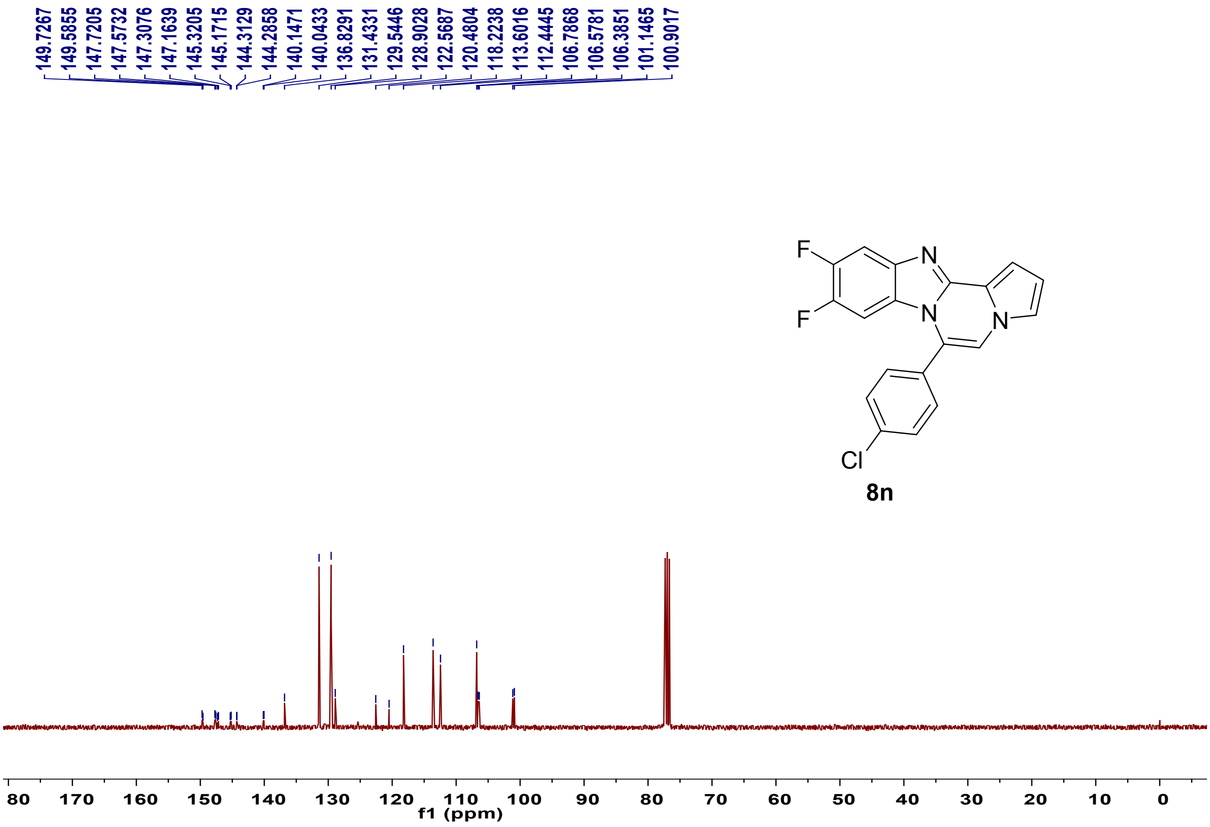
149.7267
149.5855
147.7205
147.5732
147.3076
147.1639
145.3205
145.1715
144.3129
144.2858
140.1471
140.0433
136.8291
131.4331
129.5446
128.9028
122.5687
120.4804
118.2238
113.6016
112.4445
106.7868
106.5781
106.3851
101.1465
100.9017
F
F
N
N
N
Cl
8n
f1 (ppm)

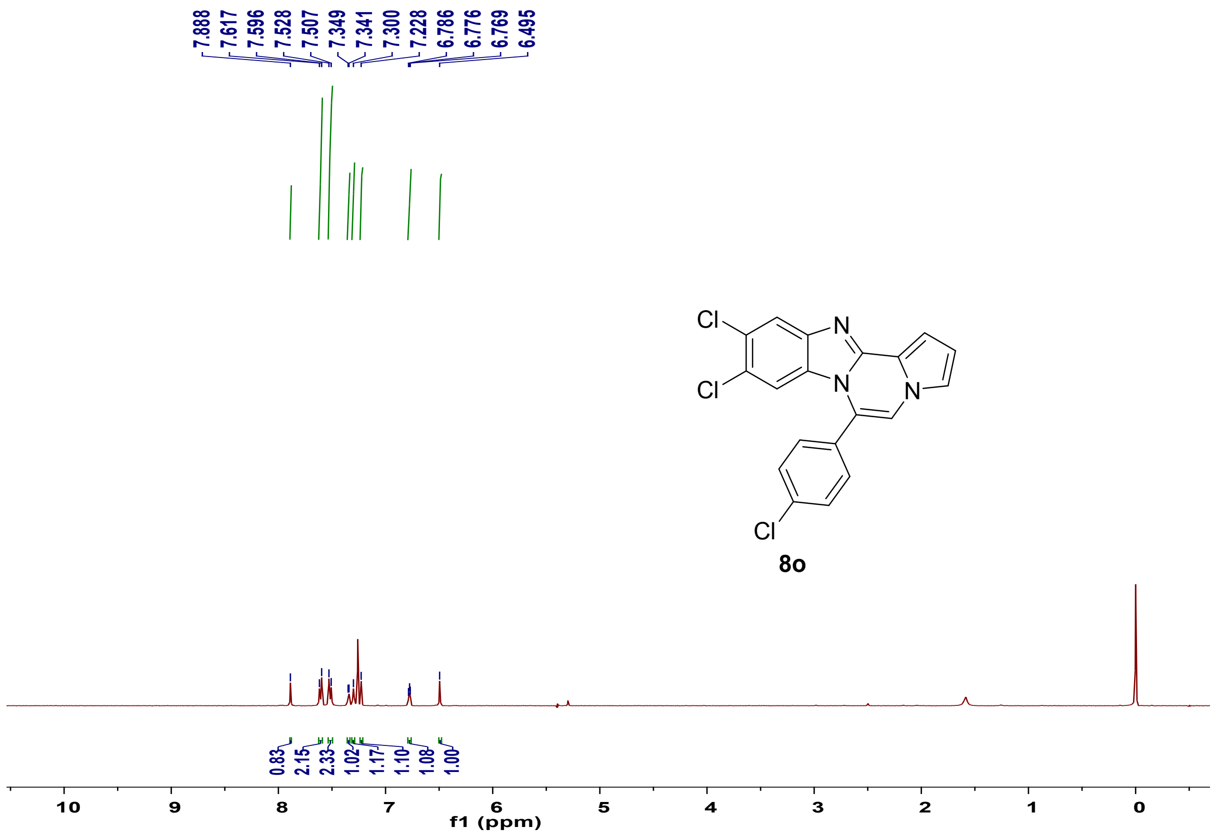
7.888
7.617
7.596
7.528
7.507
7.349
7.341
7.300
7.228
6.786
6.776
6.769
6.495
Cl
Cl
N
N
N
Cl
8o
0.83
2.15
2.33
1.02
1.17
1.10
1.08
1.00
10
9
8
7
6
5
4
3
2
1
0
f1 (ppm)

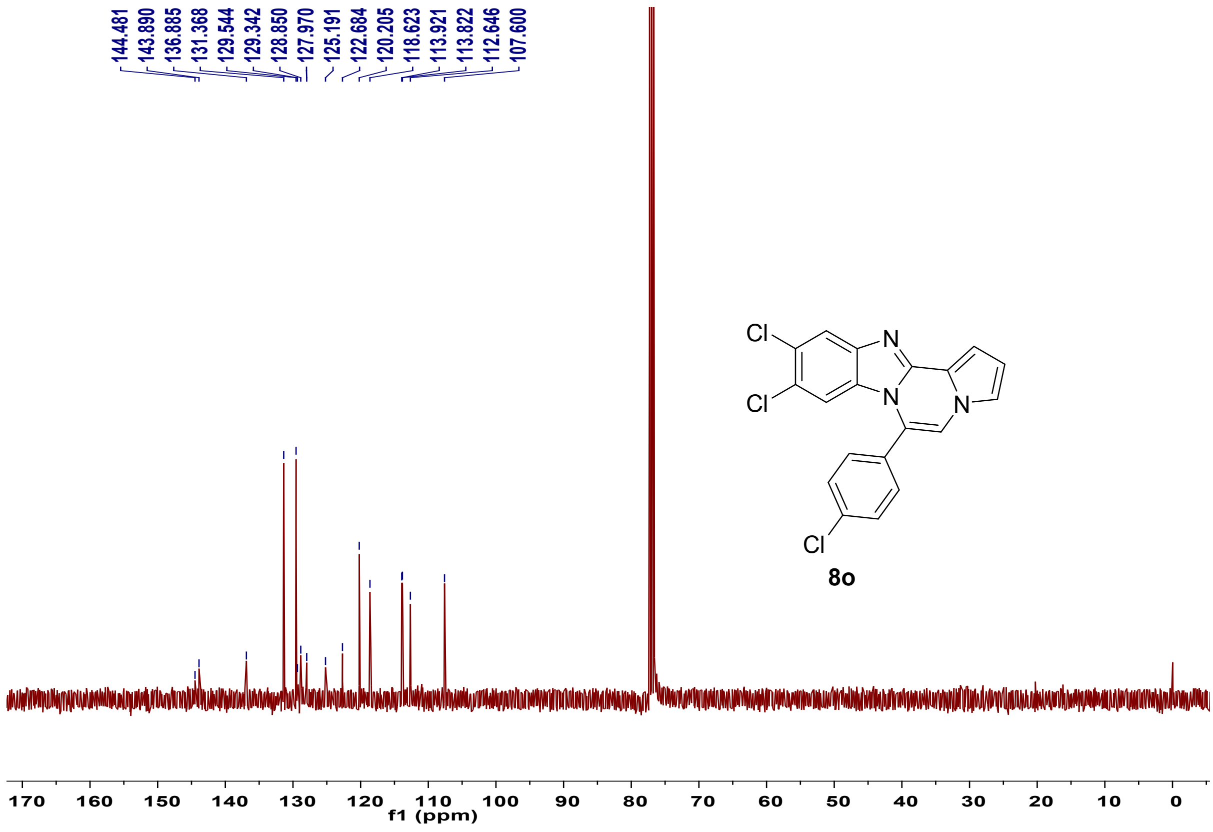
144.481
143.890
136.885
131.368
129.544
129.342
128.850
127.970
125.191
122.684
120.205
118.623
113.921
113.822
112.646
107.600
Cl
Cl
N
N
N
Cl
8o
170
160
150
140
130
120
110
100
90
80
70
60
50
40
30
20
10
0
f1 (ppm)

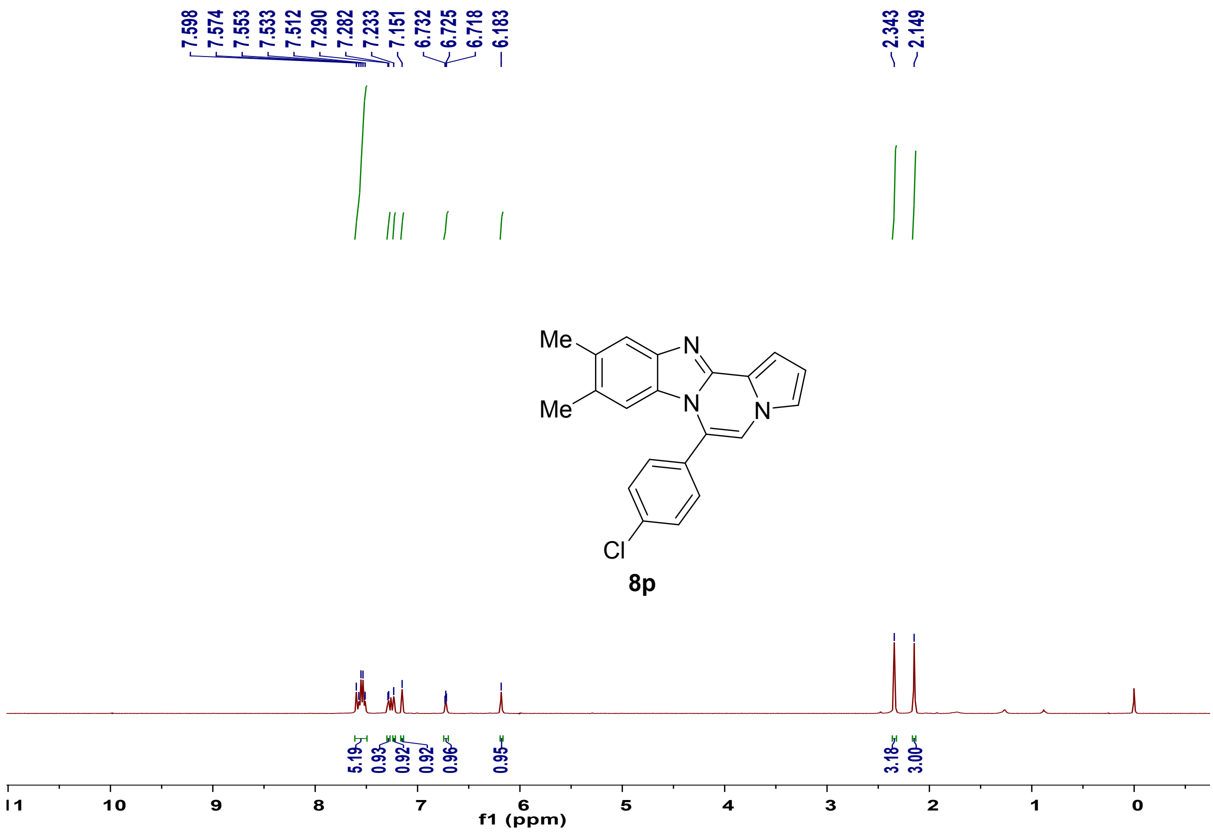
7.598
7.574
7.553
7.533
7.512
7.290
7.282
7.233
7.151
6.732
6.725
6.718
6.183
2.343
2.149
Me
Me
N
N
N
Cl
8p
5.19
0.93
0.92
0.92
0.96
0.95
3.18
3.00
f1 (ppm)

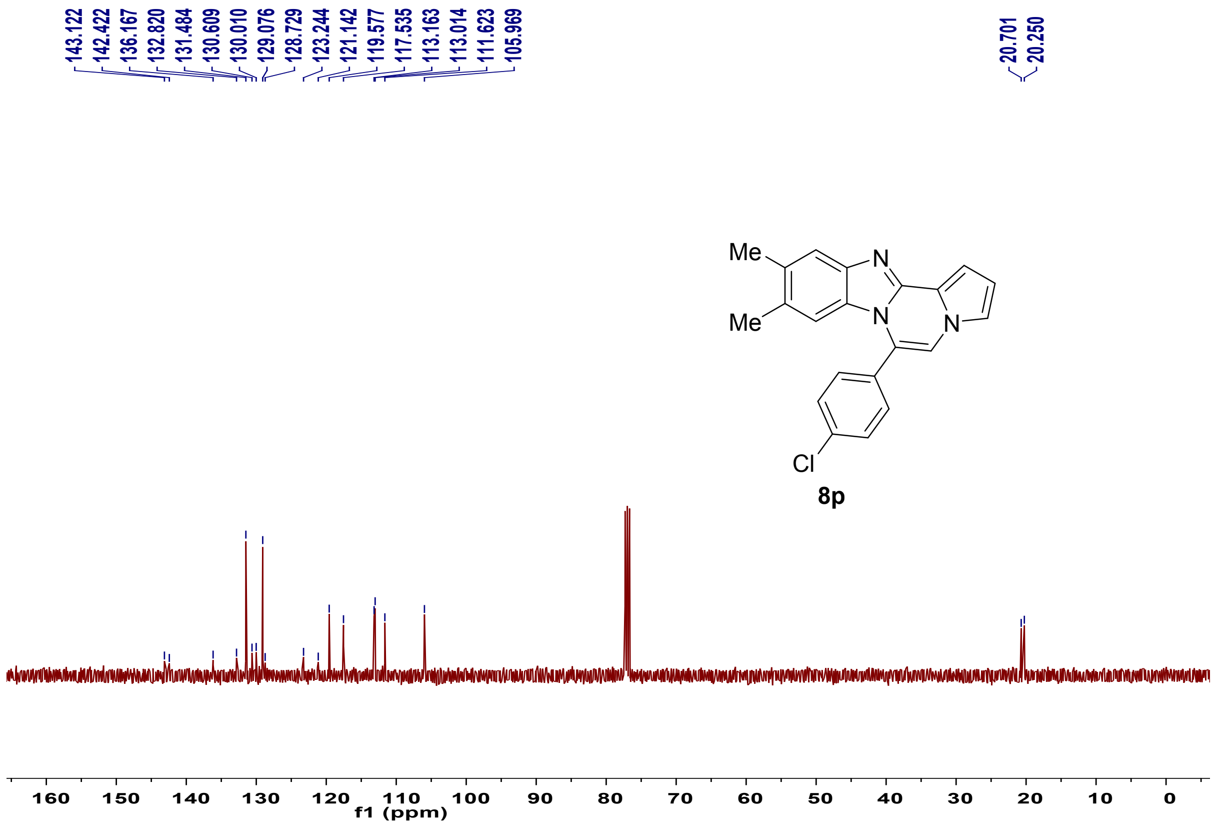
143.122
142.422
136.167
132.820
131.484
130.609
130.010
129.076
128.729
123.244
121.142
119.577
117.535
113.163
113.014
111.623
105.969
20.701
20.250
Me
Me
N
N
N
Cl
8p
f1 (ppm)

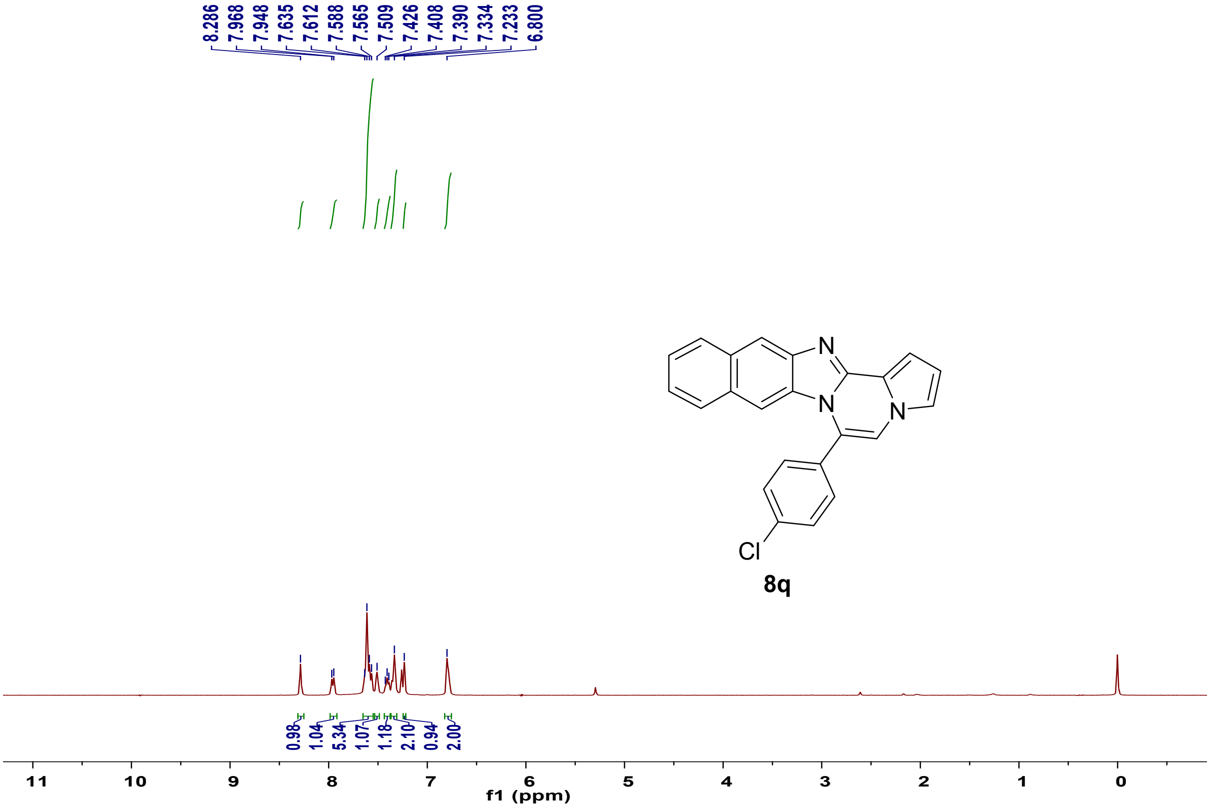
8.286
7.968
7.948
7.635
7.612
7.588
7.565
7.509
7.426
7.408
7.390
7.334
7.233
6.800
8q
0.98
1.04
5.34
1.07
1.18
2.10
0.94
2.00
f1 (ppm)

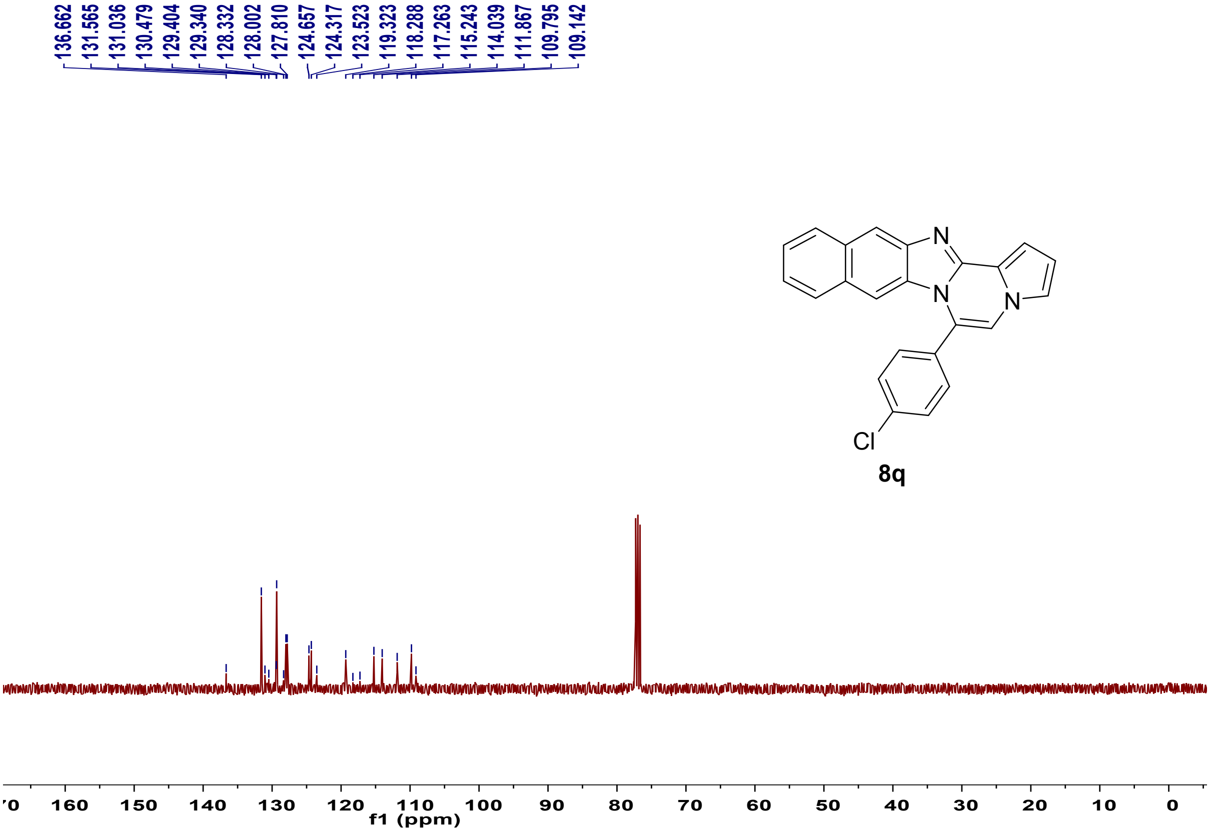
136.662
131.565
131.036
130.479
129.404
129.340
128.332
128.002
127.810
124.657
124.317
123.523
119.323
118.288
117.263
115.243
114.039
111.867
109.795
109.142
8q
f1 (ppm)

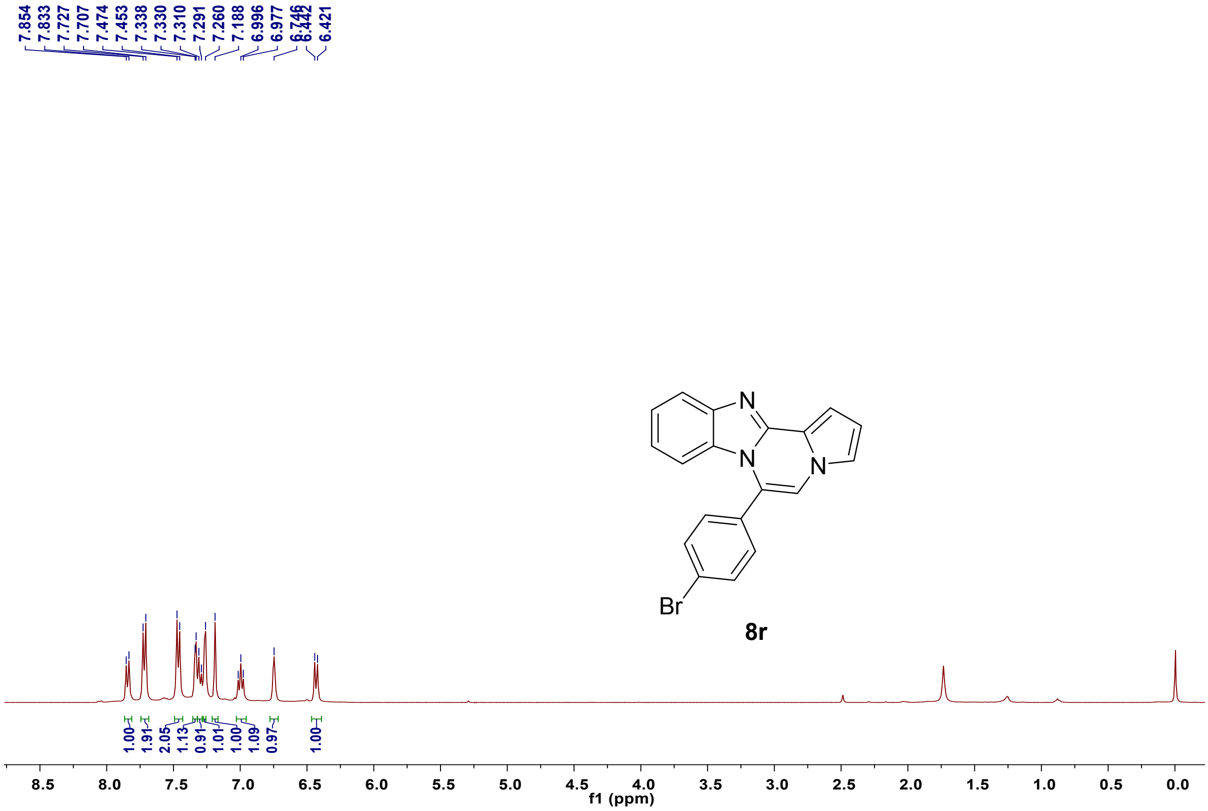
7.854
7.833
7.727
7.707
7.474
7.453
7.338
7.330
7.310
7.291
7.260
7.188
6.996
6.977
6.746
6.442
6.421
N
N
N
Br
8r
1.00
1.91
2.05
1.13
0.91
1.01
1.00
1.09
0.97
1.00
8.5 8.0 7.5 7.0 6.5 6.0 5.5 5.0 4.5 4.0 3.5 3.0 2.5 2.0 1.5 1.0 0.5 0.0
f1 (ppm)

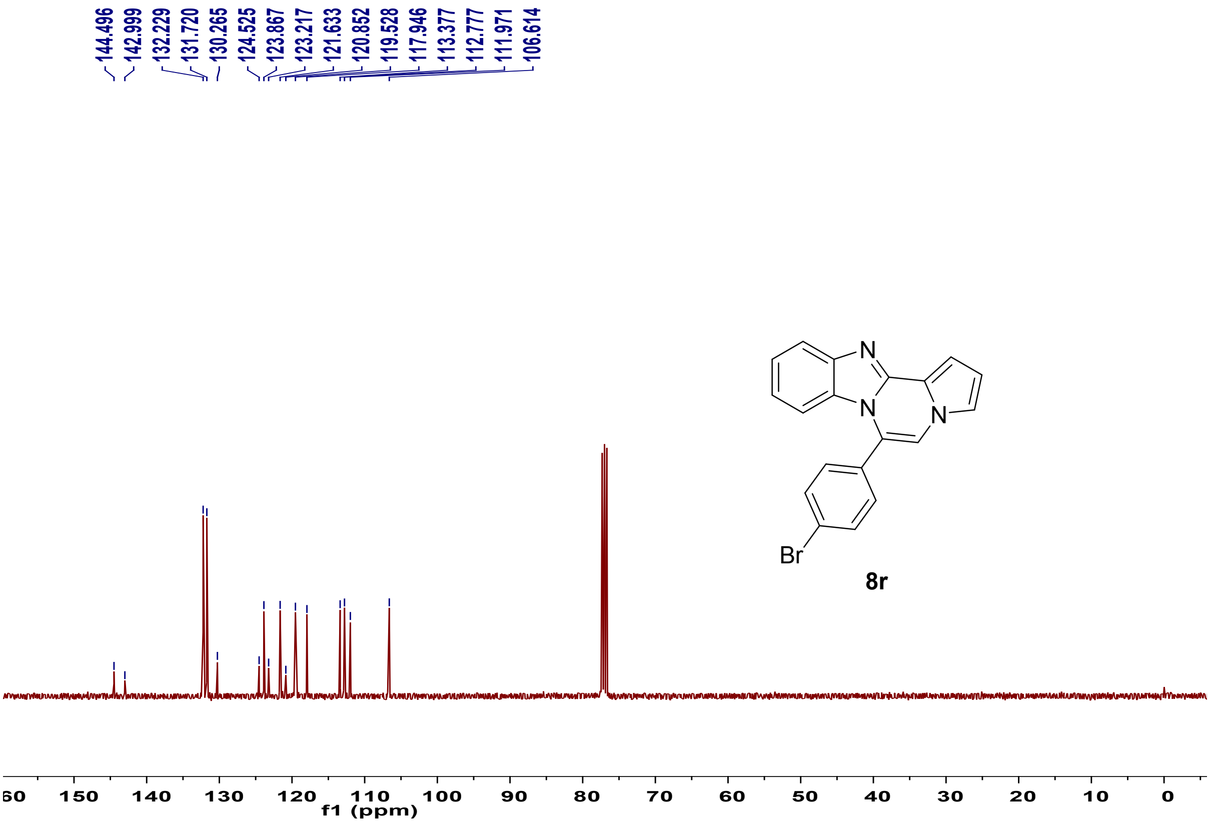
144.496
142.999
132.229
131.720
130.265
124.525
123.867
123.217
121.633
120.852
119.528
117.946
113.377
112.777
111.971
106.614
N
N
N
Br
8r
60 150 140 130 120 110 100 90 80 70 60 50 40 30 20 10 0
f1 (ppm)

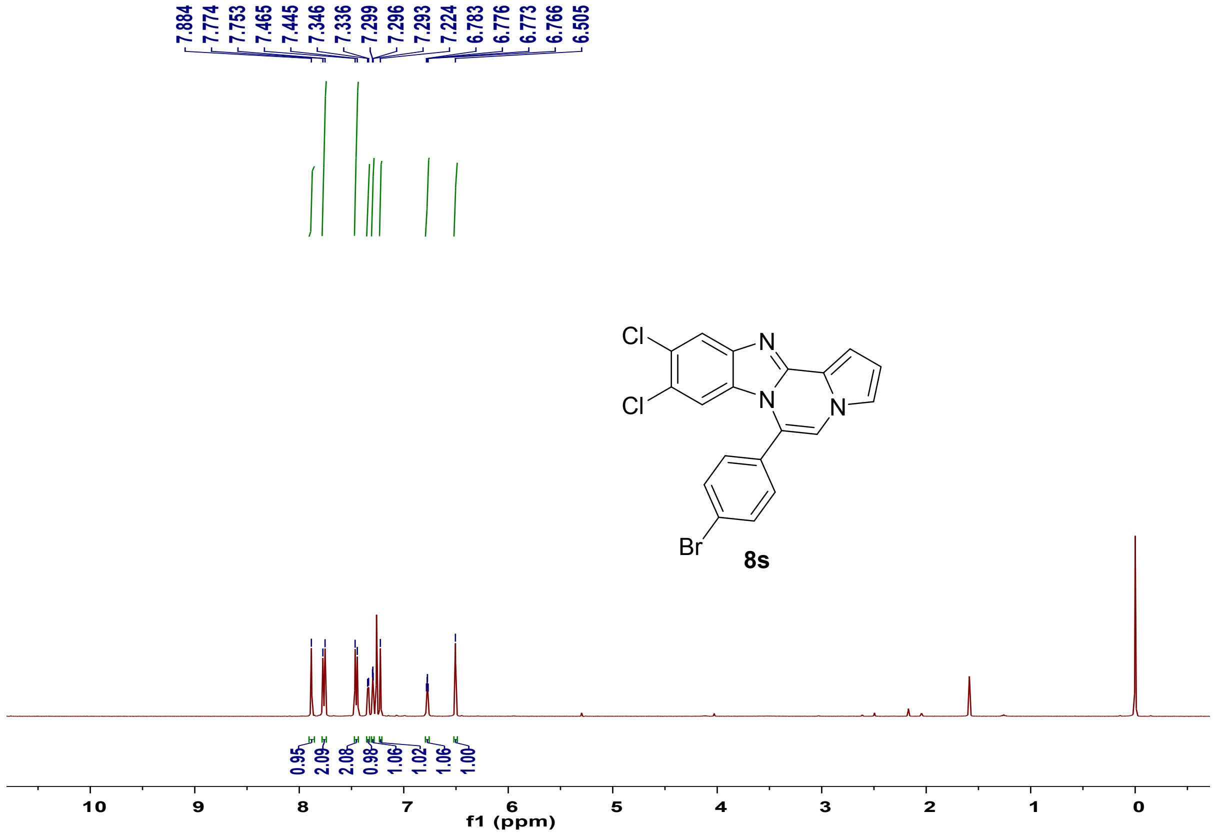
7.884
7.774
7.753
7.465
7.445
7.346
7.336
7.299
7.296
7.293
7.224
6.783
6.776
6.773
6.766
6.505
Cl
Cl
N
N
N
Br
8s
0.95
2.09
2.08
0.98
1.06
1.02
1.06
1.00
10
9
8
7
6
5
4
3
2
1
0
f1 (ppm)

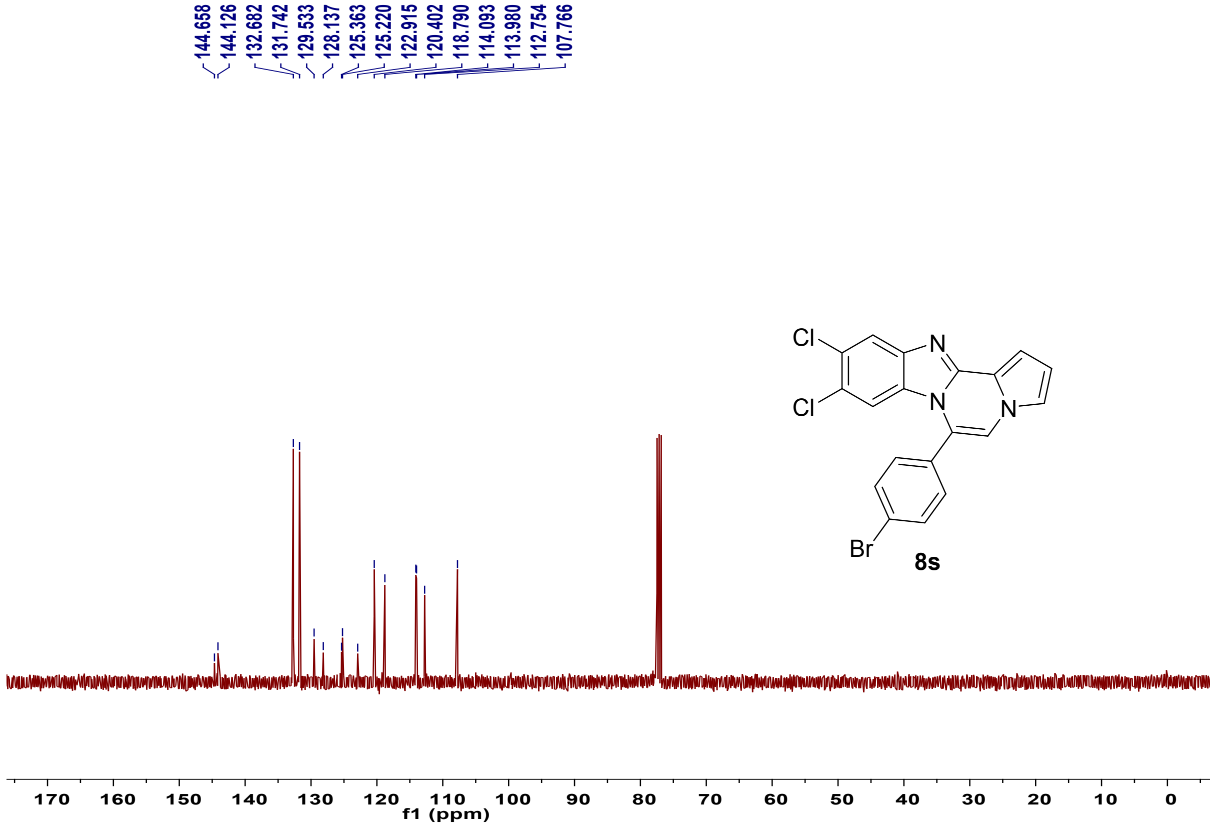
144.658
144.126
132.682
131.742
129.533
128.137
125.363
125.220
122.915
120.402
118.790
114.093
113.980
112.754
107.766
Cl
Cl
N
N
N
Br
8s
170
160
150
140
130
120
110
100
90
80
70
60
50
40
30
20
10
0
f1 (ppm)

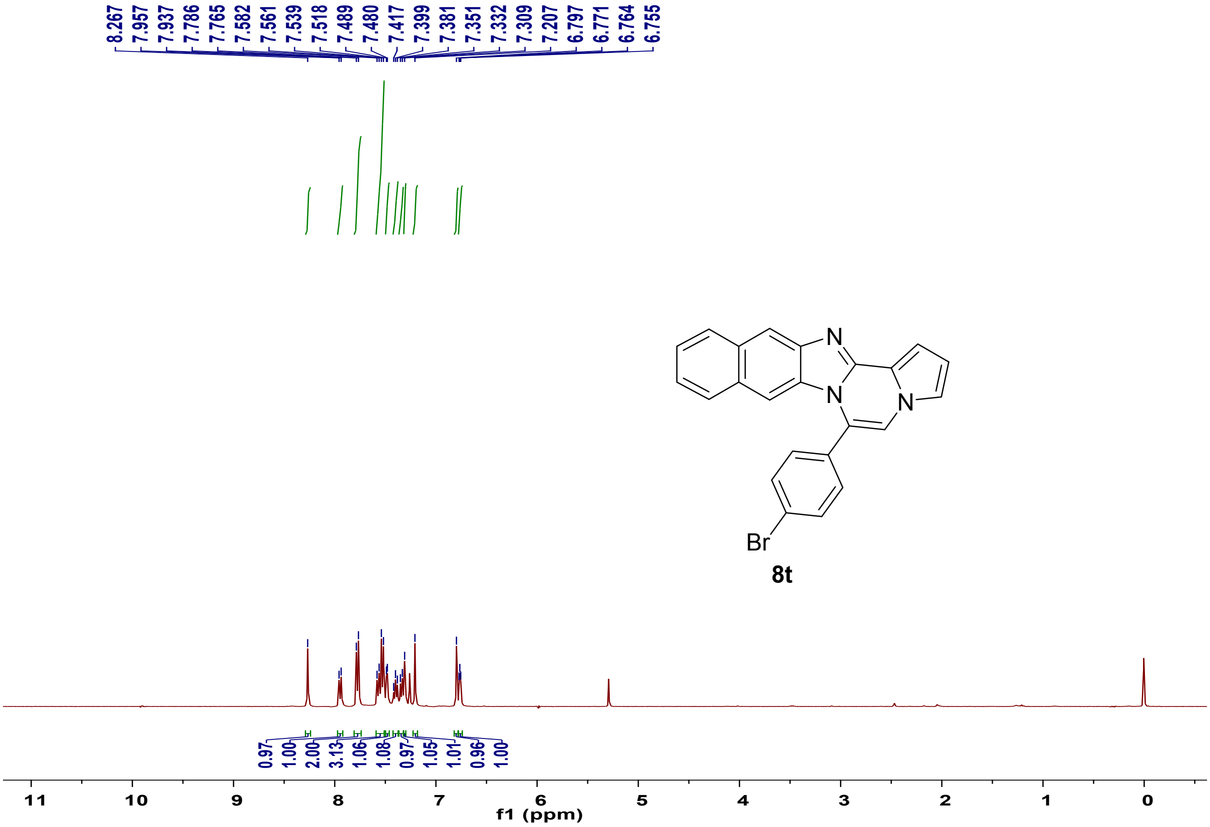

8.267
7.957
7.937
7.786
7.765
7.582
7.561
7.539
7.518
7.489
7.480
7.417
7.399
7.381
7.351
7.332
7.309
7.207
6.797
6.771
6.764
6.755
N
N
N
Br
8t
0.97
1.00
2.00
3.13
1.06
1.08
0.97
1.05
1.01
0.96
1.00
11 10 9 8 7 6 5 4 3 2 1 0
f1 (ppm)

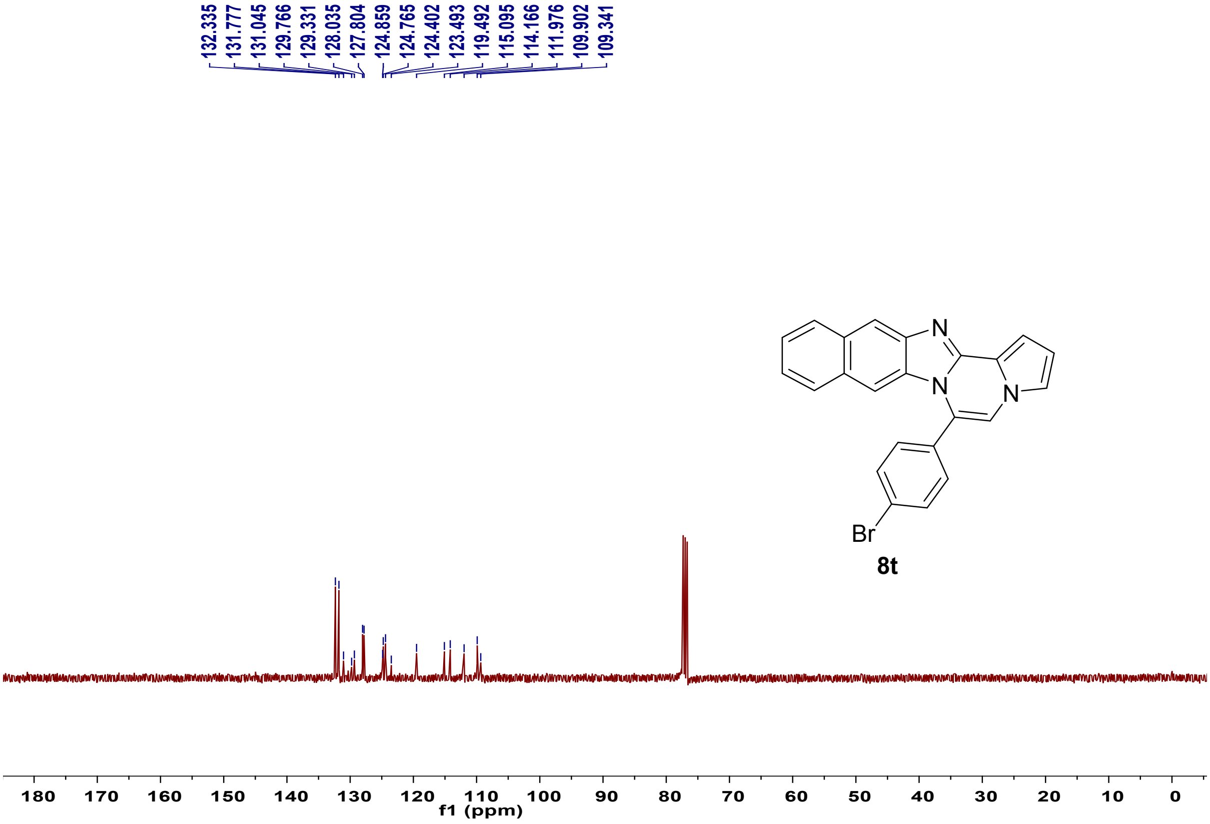

132.335
131.777
131.045
129.766
129.331
128.035
127.804
124.859
124.765
124.402
123.493
119.492
115.095
114.166
111.976
109.902
109.341
N
N
N
Br
8t
180 170 160 150 140 130 120 110 100 90 80 70 60 50 40 30 20 10 0
f1 (ppm)

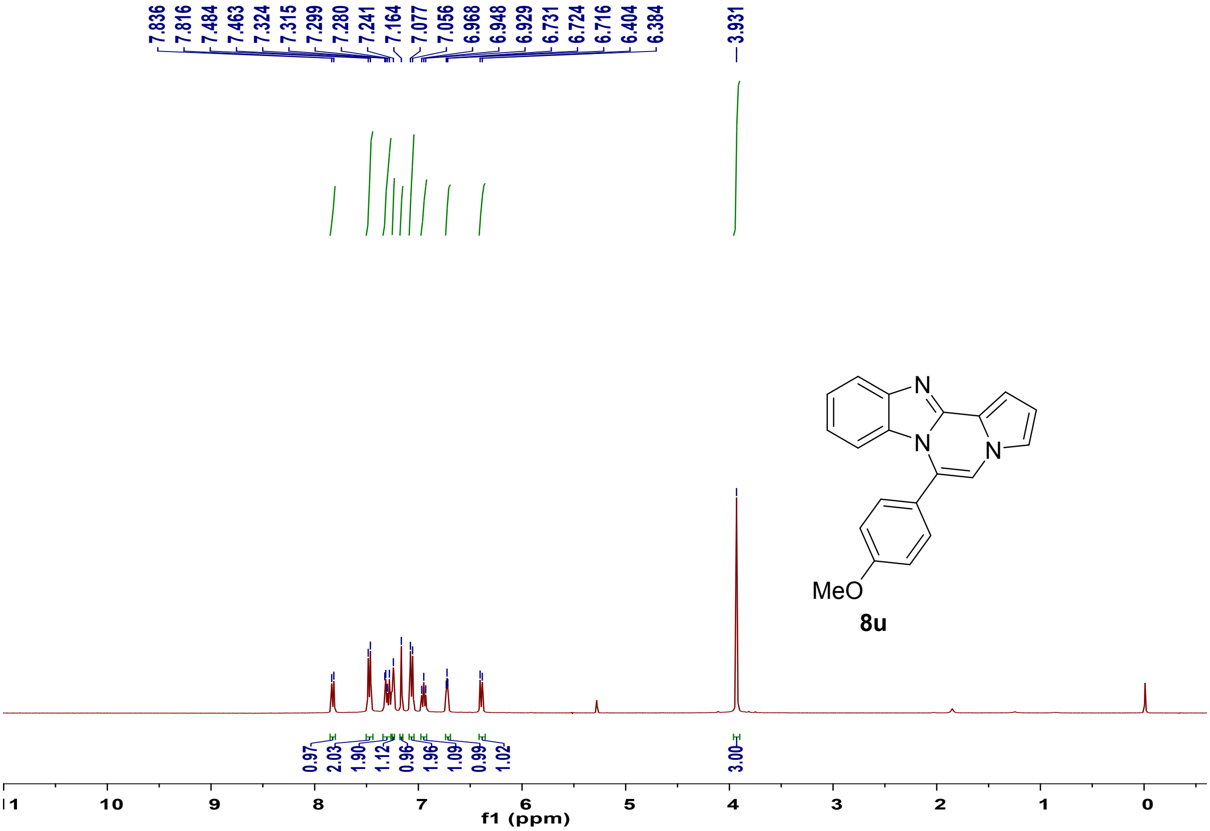
7.836
7.816
7.484
7.463
7.324
7.315
7.299
7.280
7.241
7.164
7.077
7.056
6.968
6.948
6.929
6.731
6.724
6.716
6.404
6.384
3.931
MeO
8u
0.97
2.03
1.90
1.12
0.96
1.96
1.09
0.99
1.02
3.00
f1 (ppm)

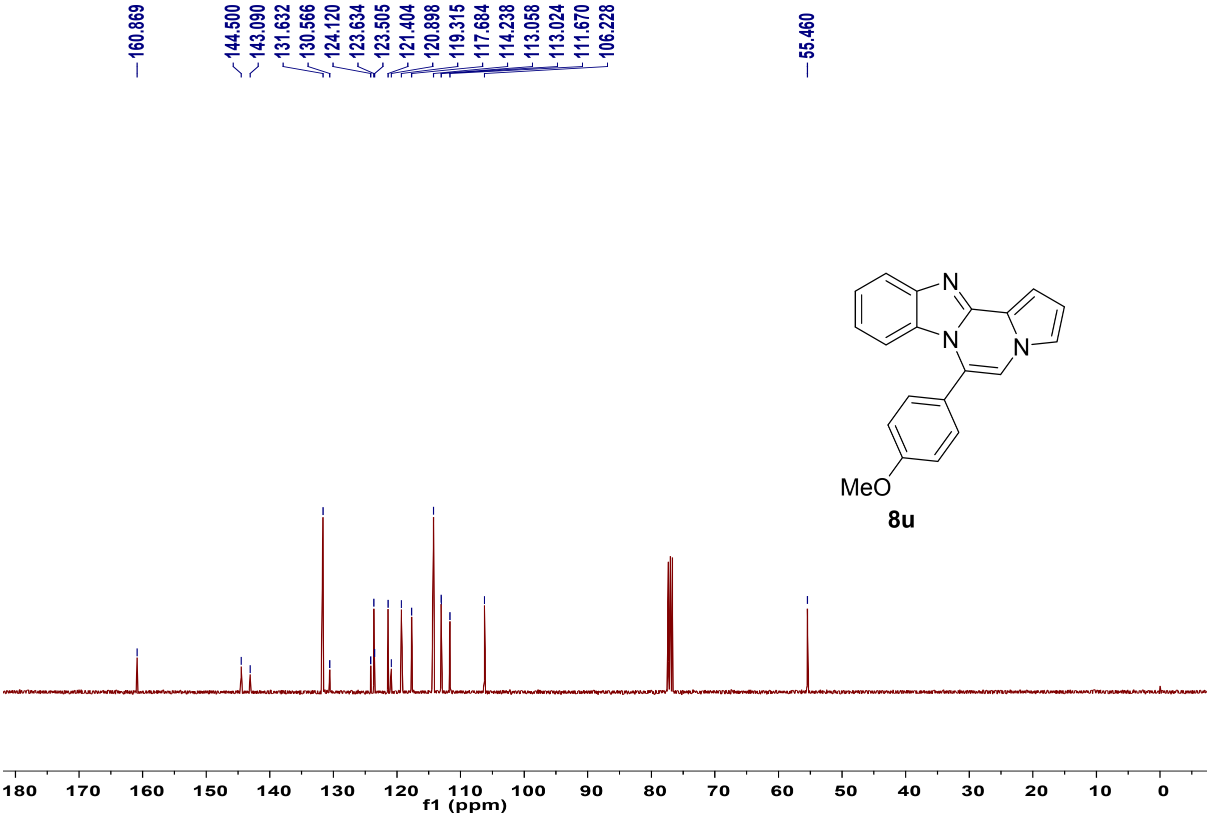
160.869
144.500
143.090
131.632
130.566
124.120
123.634
123.505
121.404
120.898
119.315
117.684
114.238
113.058
113.024
111.670
106.228
55.460
MeO
8u
f1 (ppm)

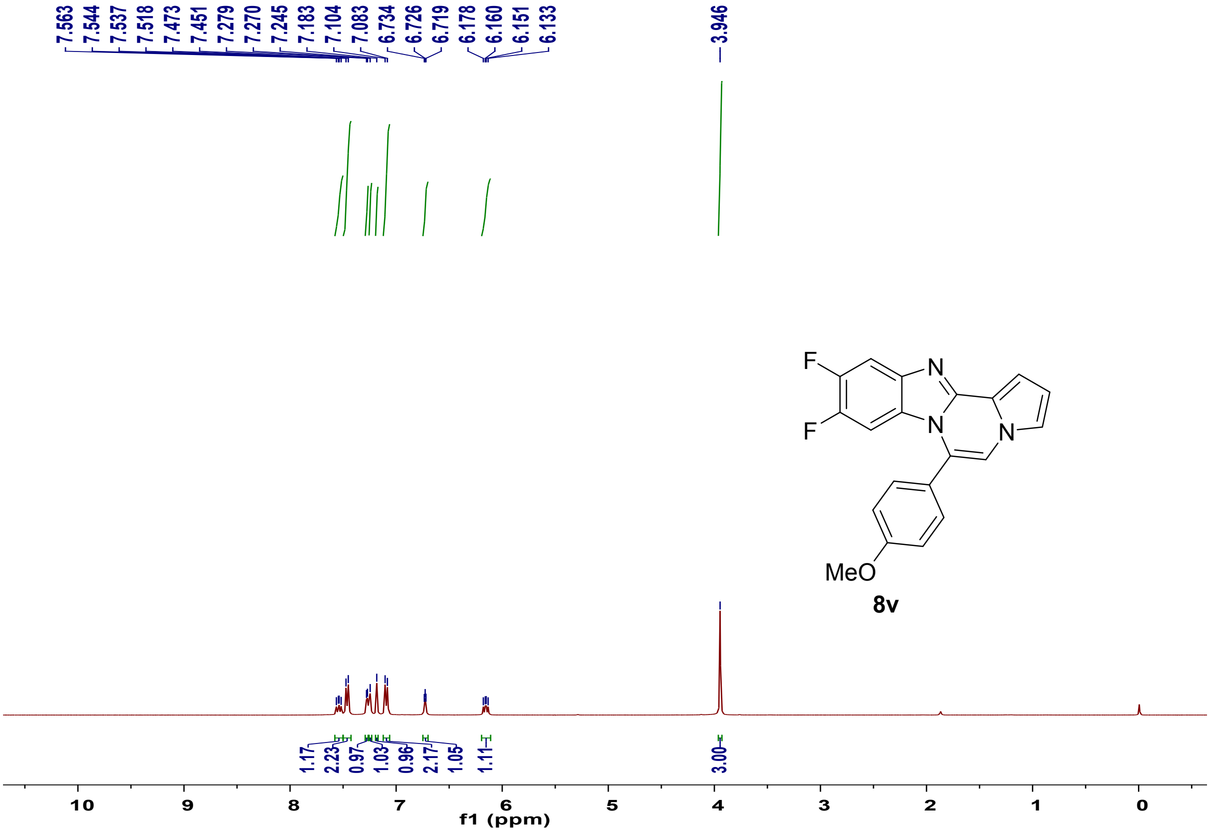

7.563
7.544
7.537
7.518
7.473
7.451
7.279
7.270
7.245
7.183
7.104
7.083
6.734
6.726
6.719
6.178
6.160
6.151
6.133
3.946
F
F
N
N
N
MeO
8v
1.17
2.23
0.97
1.03
0.96
2.17
1.05
1.11
3.00
f1 (ppm)

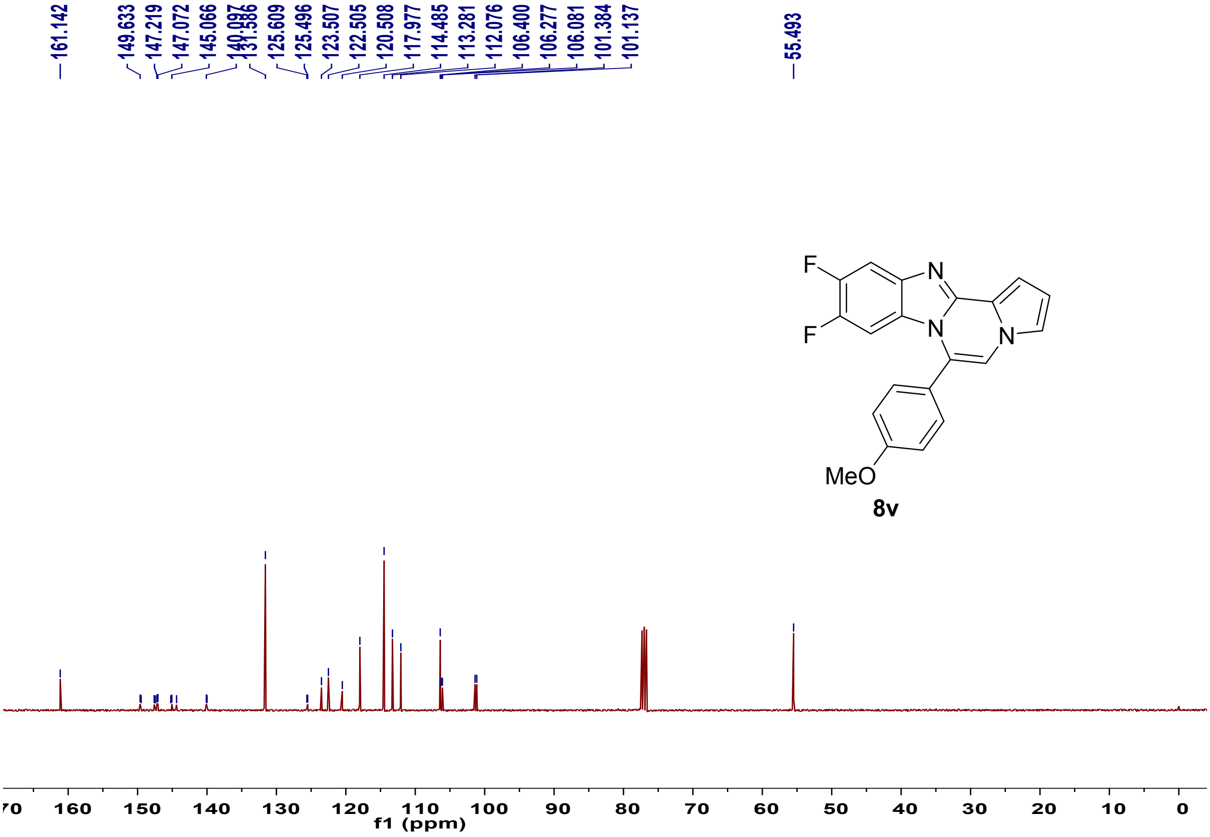

161.142
149.633
147.219
147.072
145.066
140.097
131.586
125.609
125.496
123.507
122.505
120.508
117.977
114.485
113.281
112.076
106.400
106.277
106.081
101.384
101.137
55.493
F
F
N
N
N
MeO
8v
f1 (ppm)

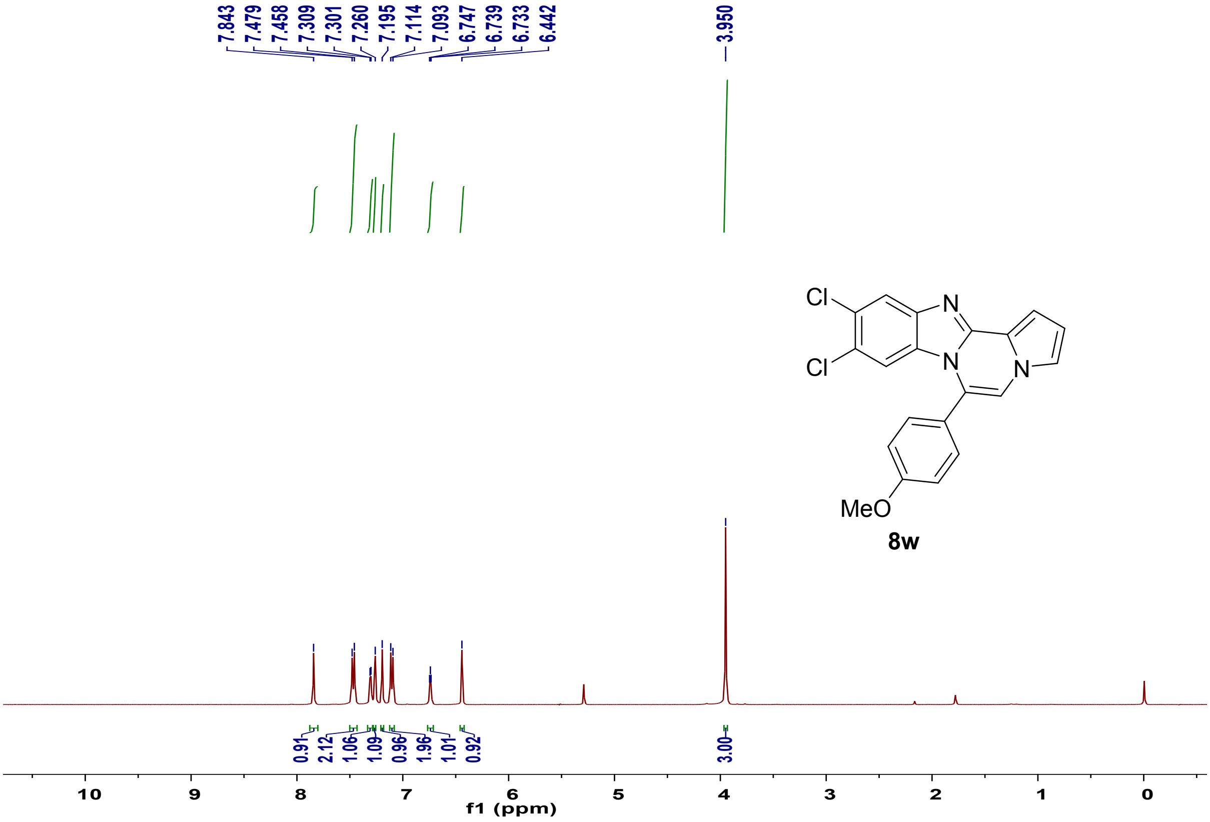

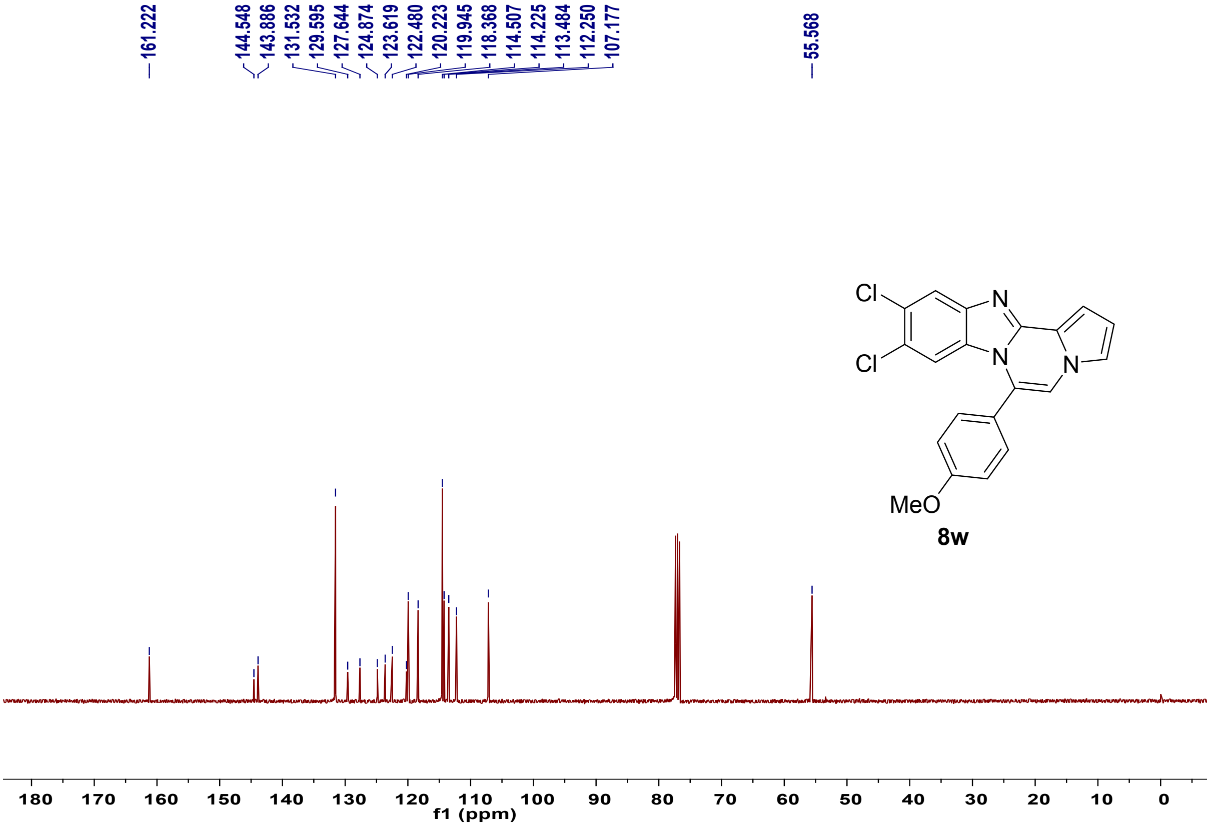

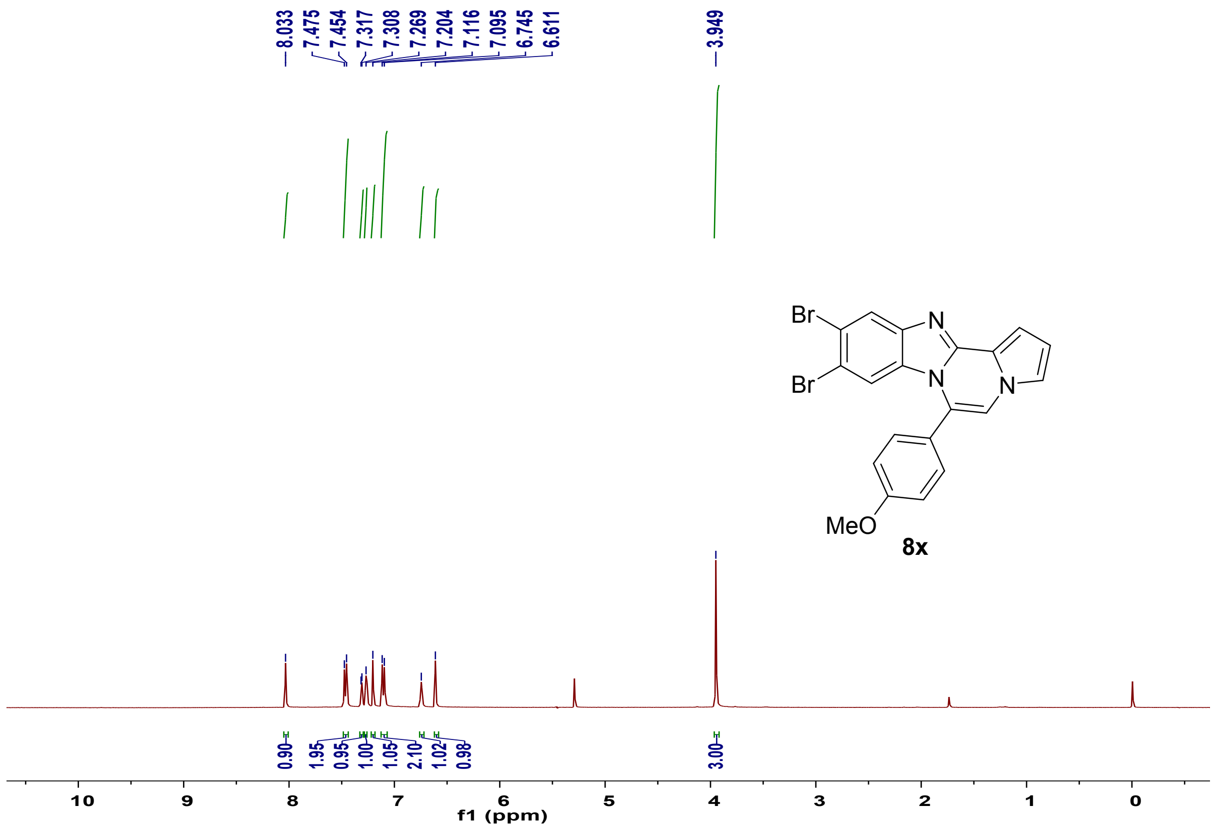
8.033
7.475
7.454
7.317
7.308
7.269
7.204
7.116
7.095
6.745
6.611
3.949
Br
Br
N
N
N
MeO
8x
0.90
1.95
0.95
1.00
1.05
2.10
1.02
0.98
3.00
f1 (ppm)

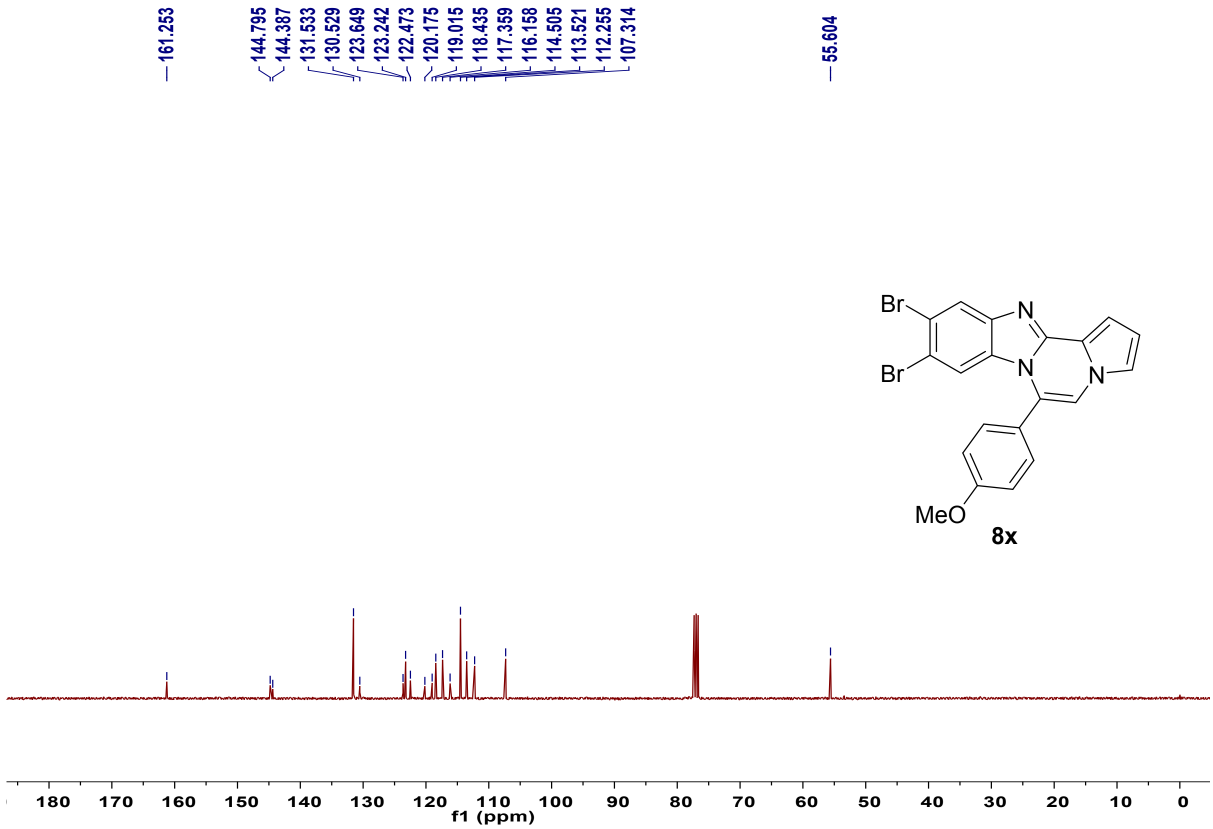
161.253
144.795
144.387
131.533
130.529
123.649
123.242
122.473
120.175
119.015
118.435
117.359
116.158
114.505
113.521
112.255
107.314
55.604
Br
Br
N
N
N
MeO
8x
f1 (ppm)

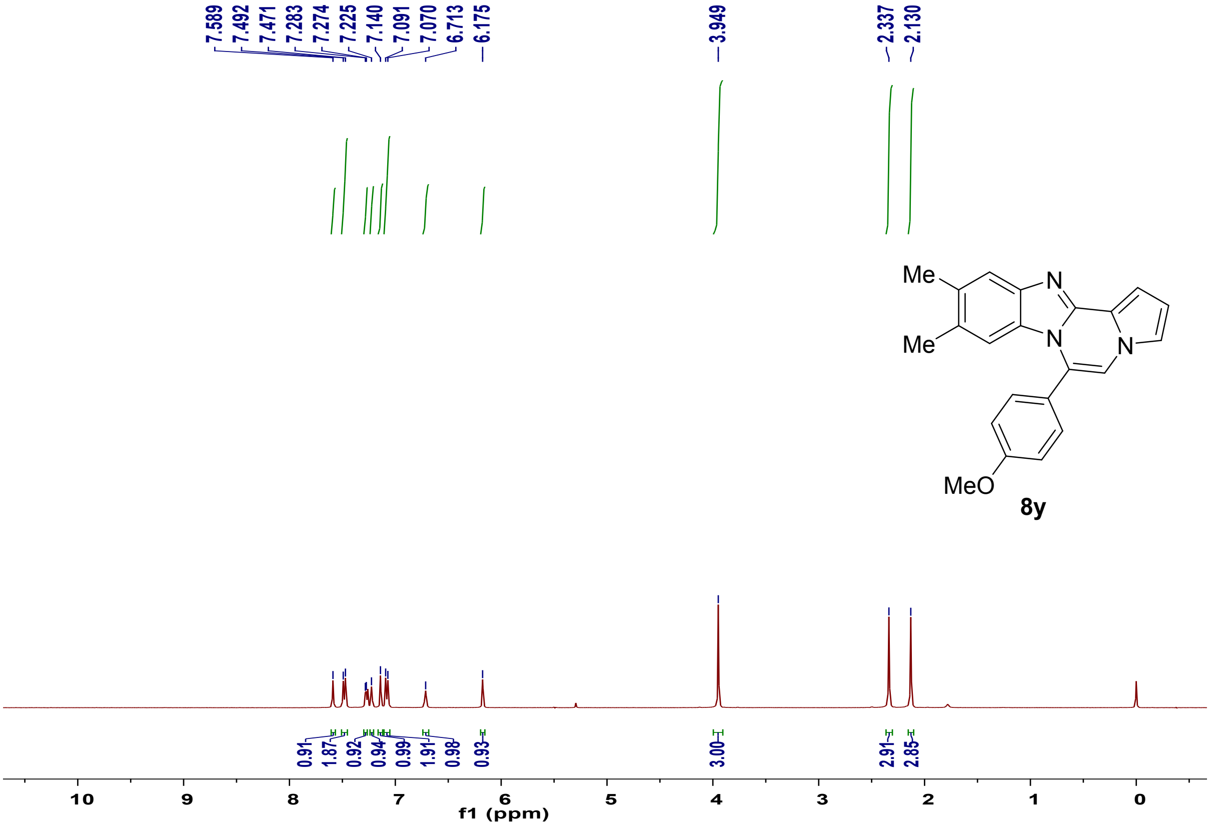
7.589
7.492
7.471
7.283
7.274
7.225
7.140
7.091
7.070
6.713
6.175
3.949
2.337
2.130
Me
Me
N
N
N
MeO
8y
0.91
1.87
0.92
0.94
0.99
1.91
0.98
0.93
3.00
2.91
2.85
f1 (ppm)

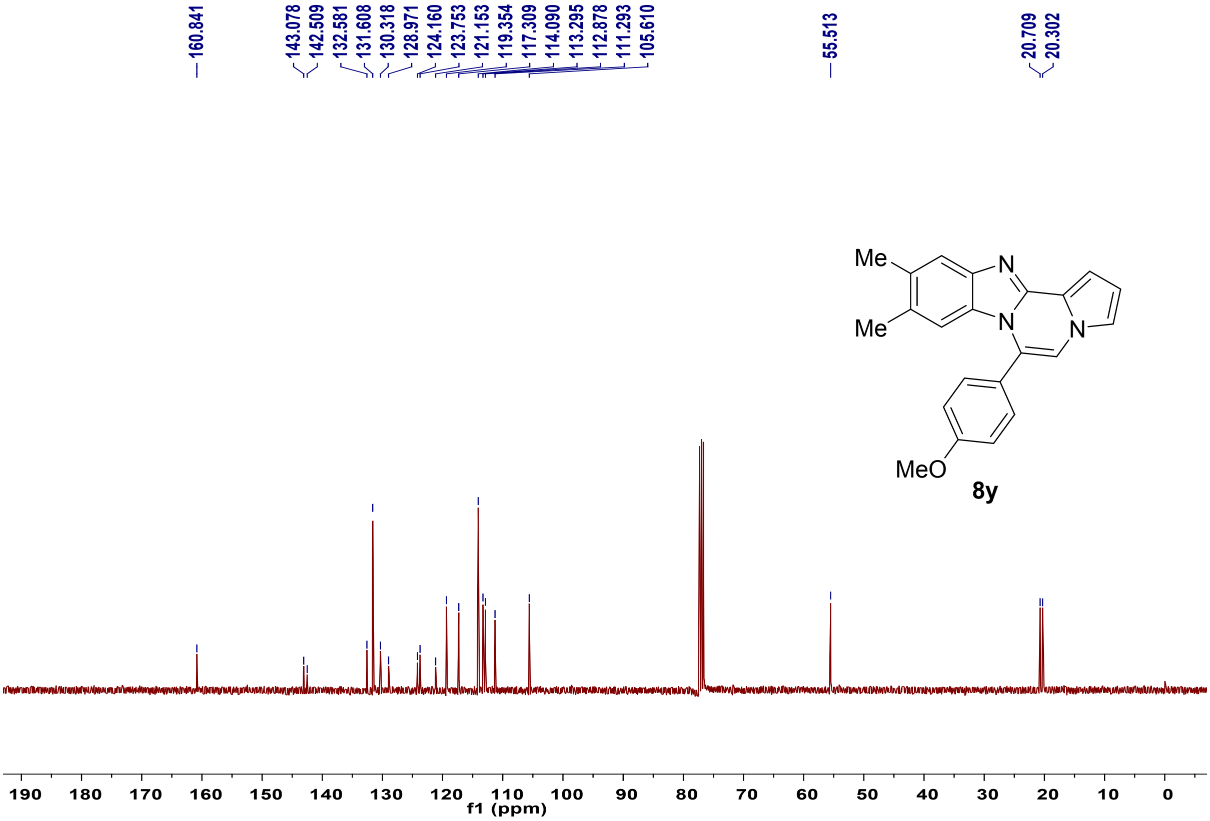
160.841
143.078
142.509
132.581
131.608
130.318
128.971
124.160
123.753
121.153
119.354
117.309
114.090
113.295
112.878
111.293
105.610
55.513
20.709
20.302
Me
Me
N
N
N
MeO
8y
f1 (ppm)

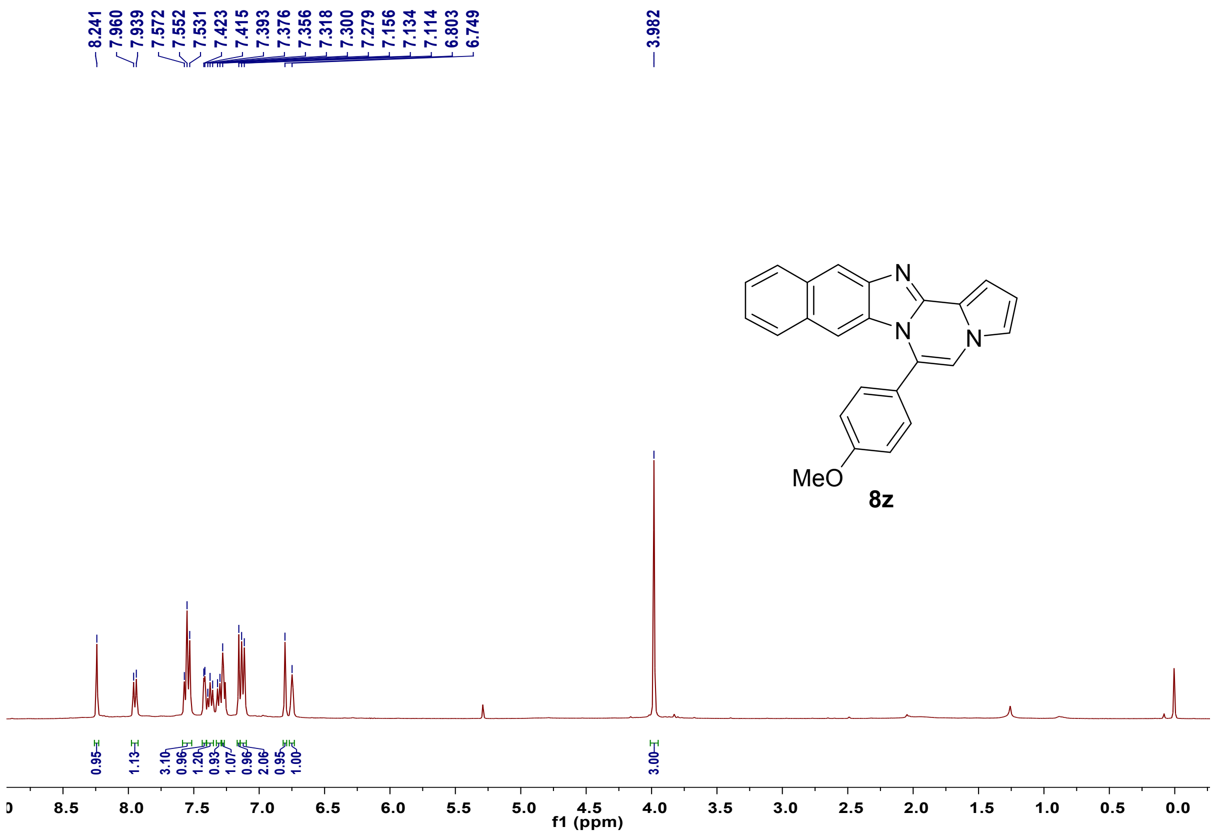
8.241
7.960
7.939
7.572
7.552
7.531
7.423
7.415
7.393
7.376
7.356
7.318
7.300
7.279
7.156
7.134
7.114
6.803
6.749
3.982
N
N
N
MeO
8z
0.95
1.13
3.10
0.96
1.20
0.93
1.07
0.96
2.06
0.95
1.00
3.00
f1 (ppm)

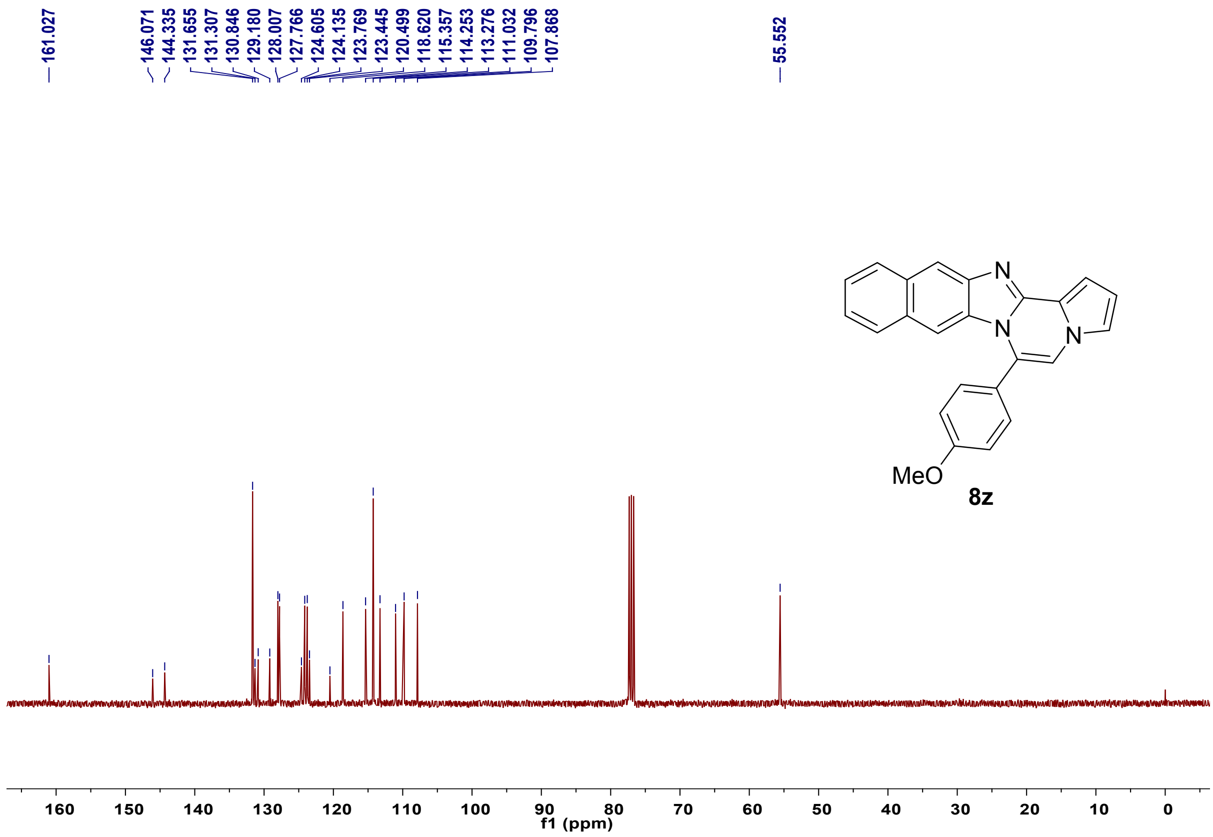
161.027
146.071
144.335
131.655
131.307
130.846
129.180
128.007
127.766
124.605
124.135
123.769
123.445
120.499
118.620
115.357
114.253
113.276
111.032
109.796
107.868
55.552
N
N
N
MeO
8z
f1 (ppm)

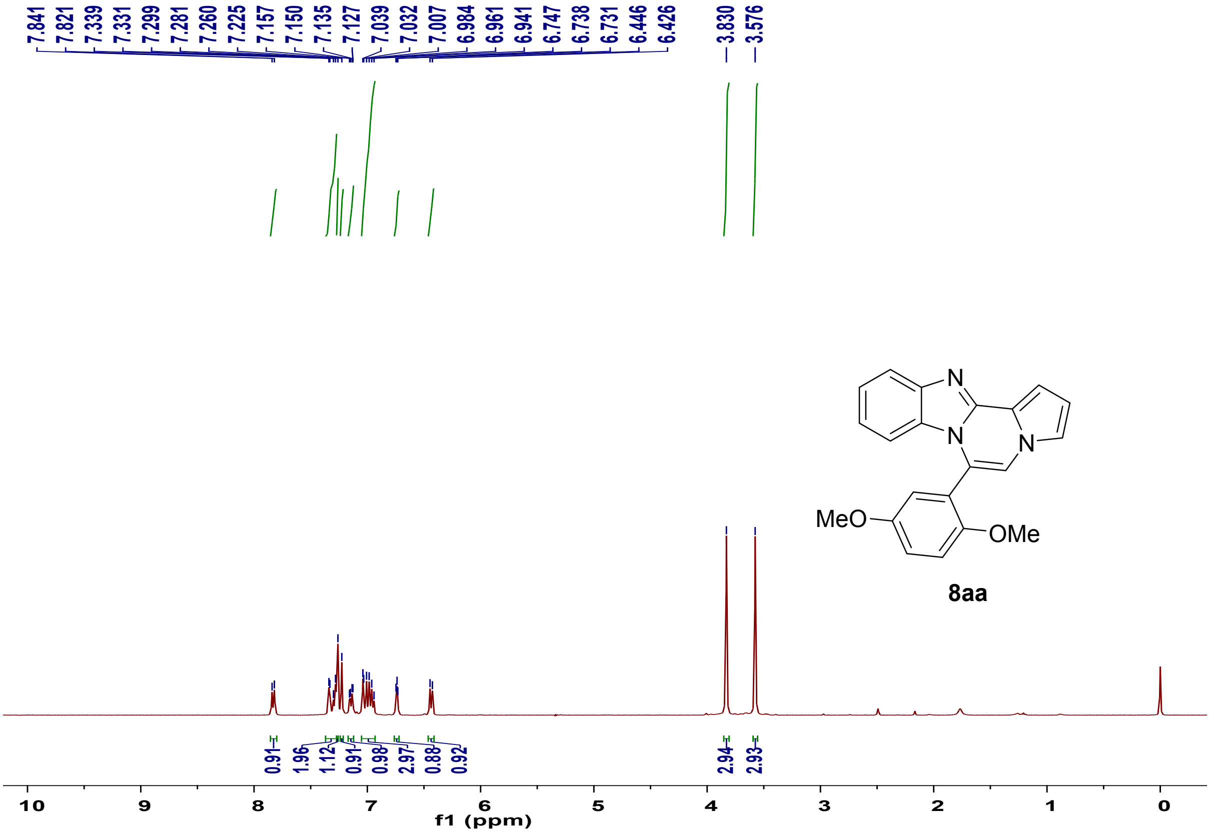
7.841
7.821
7.339
7.331
7.299
7.281
7.260
7.225
7.157
7.150
7.135
7.127
7.039
7.032
7.007
6.984
6.961
6.941
6.747
6.738
6.731
6.446
6.426
3.830
3.576
N
N
N
MeO
OMe
8aa
0.91
1.96
1.12
0.91
0.98
2.97
0.88
0.92
2.94
2.93
10
9
8
7
6
5
4
3
2
1
0
f1 (ppm)

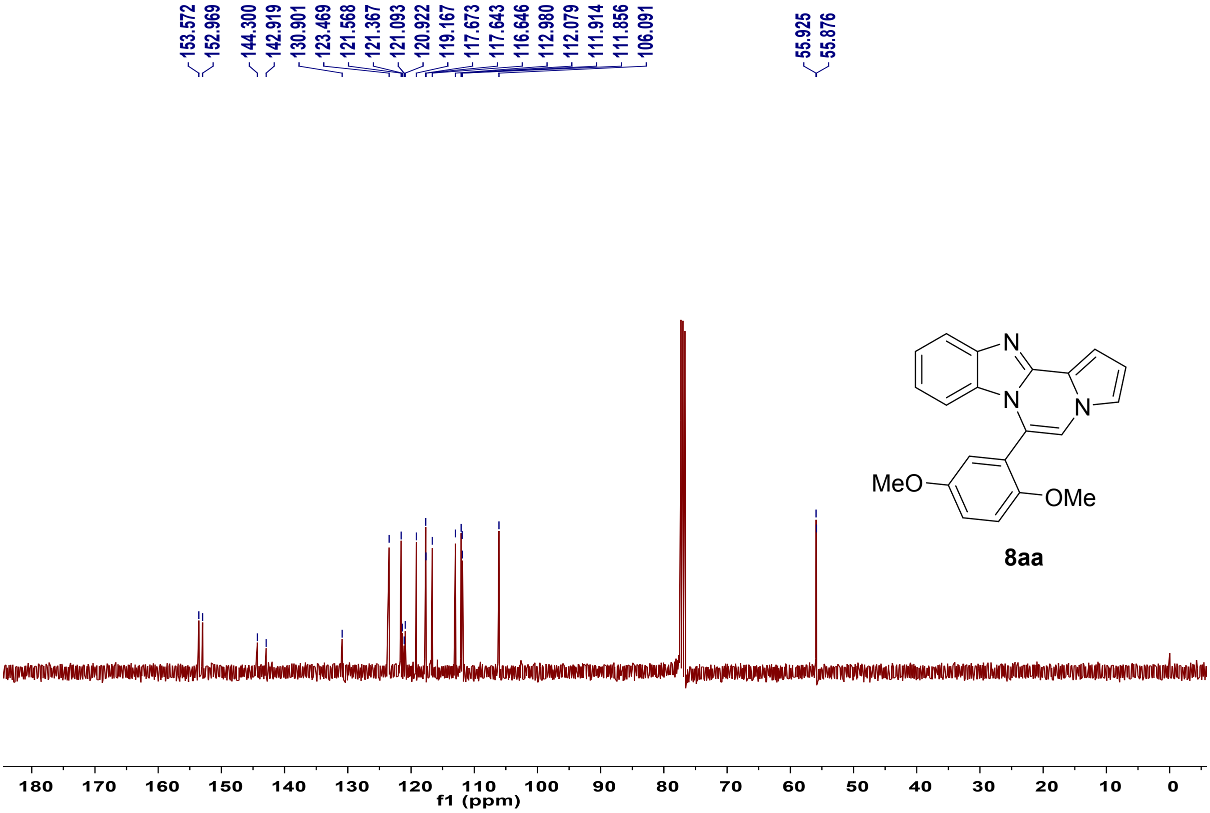
153.572
152.969
144.300
142.919
130.901
123.469
121.568
121.367
121.093
120.922
119.167
117.673
117.643
116.646
112.980
112.079
111.914
111.856
106.091
55.925
55.876
N
N
N
MeO
OMe
8aa
180
170
160
150
140
130
120
110
100
90
80
70
60
50
40
30
20
10
0
f1 (ppm)

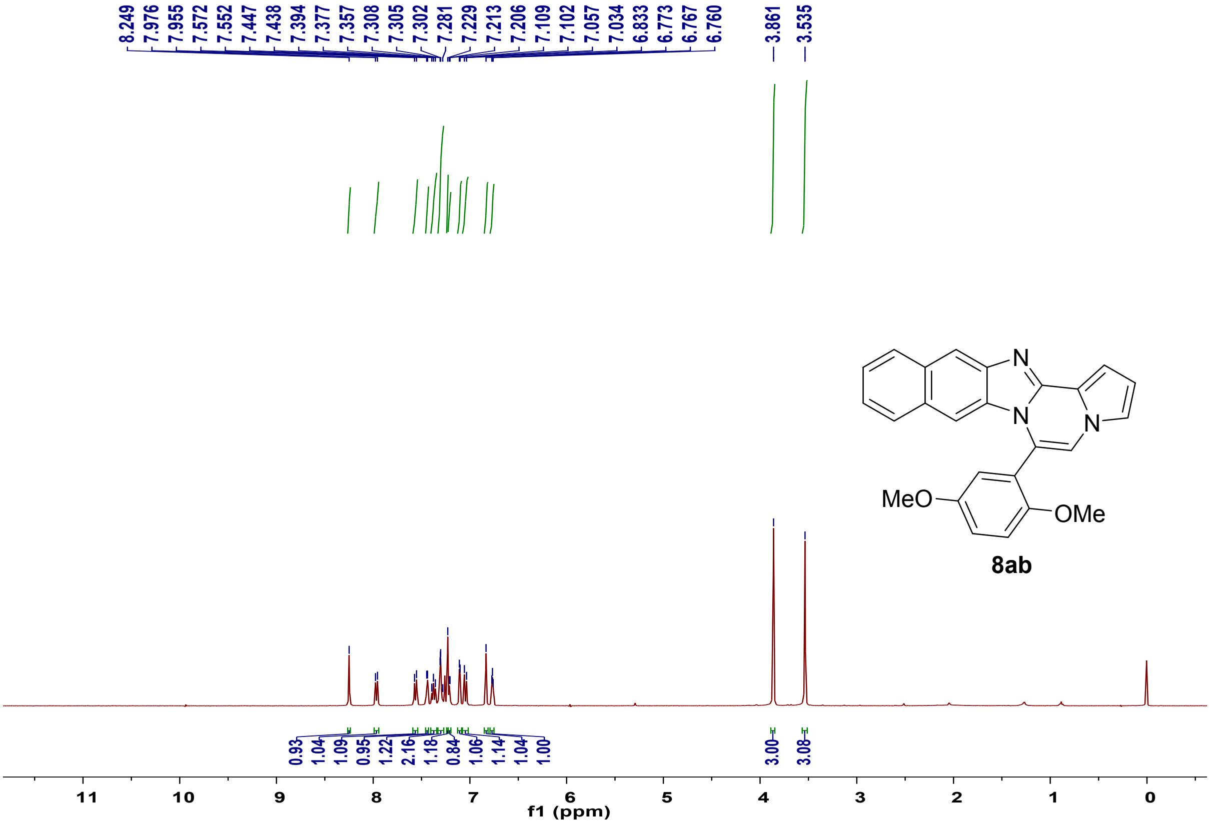
8.249 7.976 7.955 7.572 7.552 7.447 7.438 7.394 7.377 7.357 7.308 7.305 7.302 7.281 7.229 7.213 7.206 7.109 7.102 7.057 7.034 6.833 6.773 6.767 6.760
3.861
3.535
N
N
N
MeO
OMe
8ab
0.93 1.04 1.09 0.95 1.22 2.16 1.18 0.84 1.06 1.14 1.04 1.00
3.00
3.08
11 10 9 8 7 6 5 4 3 2 1 0
f1 (ppm)

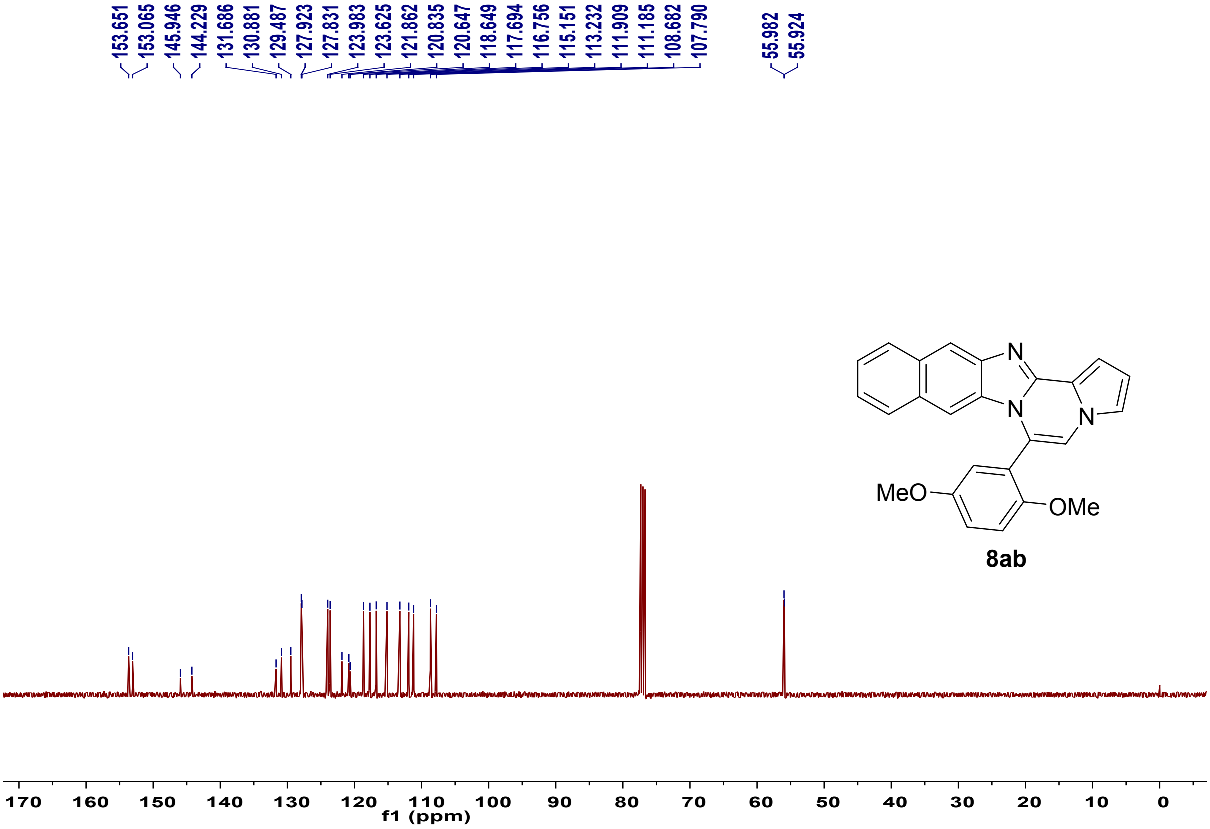
153.651 153.065 145.946 144.229 131.686 130.881 129.487 127.923 127.831 123.983 123.625 121.862 120.835 120.647 118.649 117.694 116.756 115.151 113.232 111.909 111.185 108.682 107.790
55.982
55.924
N
N
N
MeO
OMe
8ab
170 160 150 140 130 120 110 100 90 80 70 60 50 40 30 20 10 0
f1 (ppm)

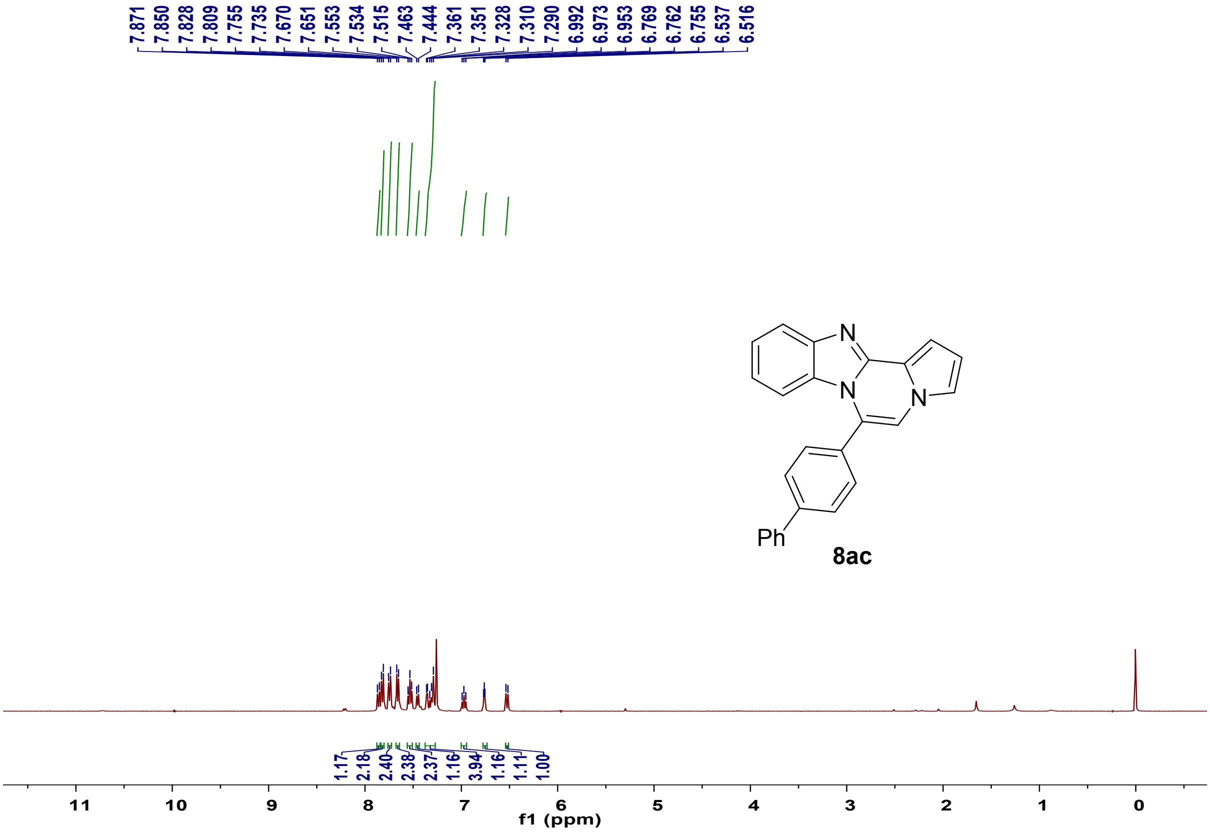
7.871 7.850 7.828 7.809 7.755 7.735 7.670 7.651 7.553 7.534 7.515 7.463 7.444 7.361 7.351 7.328 7.310 7.290 6.992 6.973 6.953 6.769 6.762 6.755 6.537 6.516
Ph
8ac
1.17 2.18 2.40 2.38 2.37 1.16 3.94 1.16 1.11 1.00
11 10 9 8 7 6 5 4 3 2 1 0
f1 (ppm)

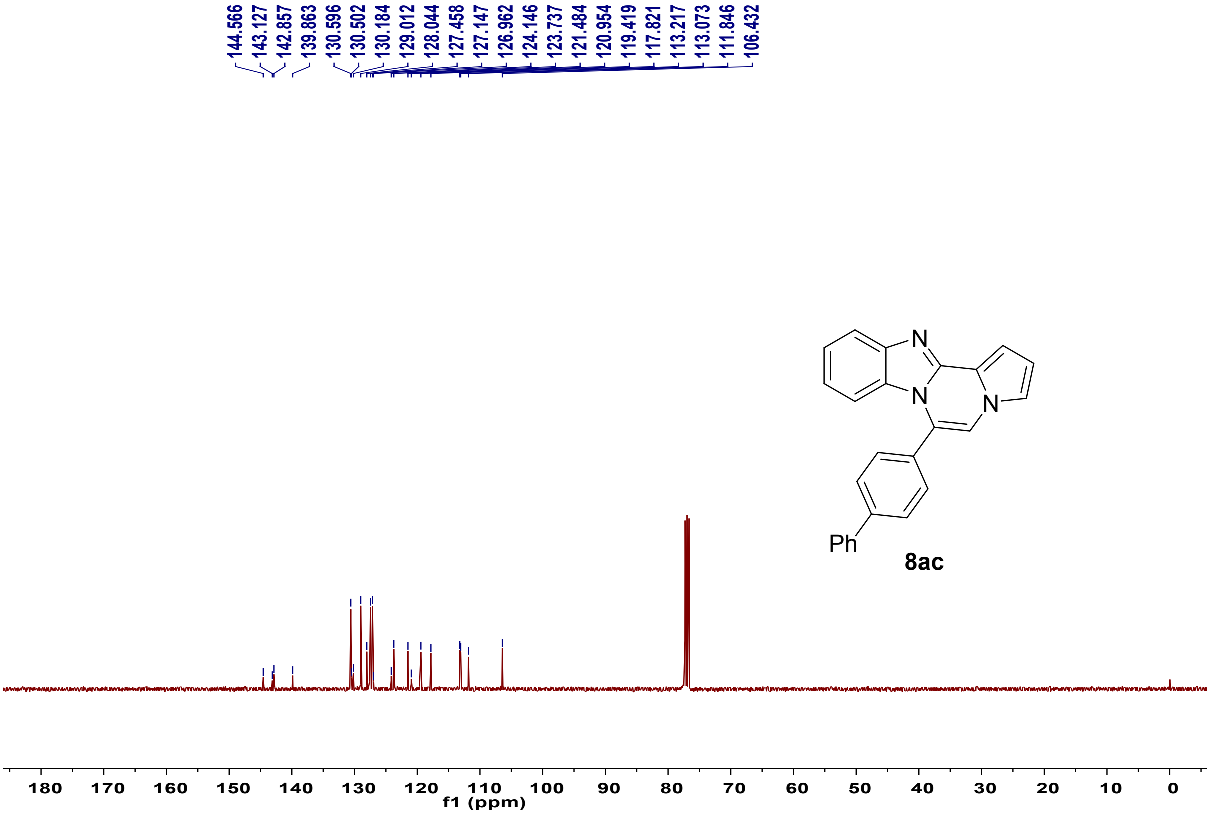
144.566 143.127 142.857 139.863 130.596 130.502 130.184 129.012 128.044 127.458 127.147 126.962 124.146 123.737 121.484 120.954 119.419 117.821 113.217 113.073 111.846 106.432
Ph
8ac
180 170 160 150 140 130 120 110 100 90 80 70 60 50 40 30 20 10 0
f1 (ppm)

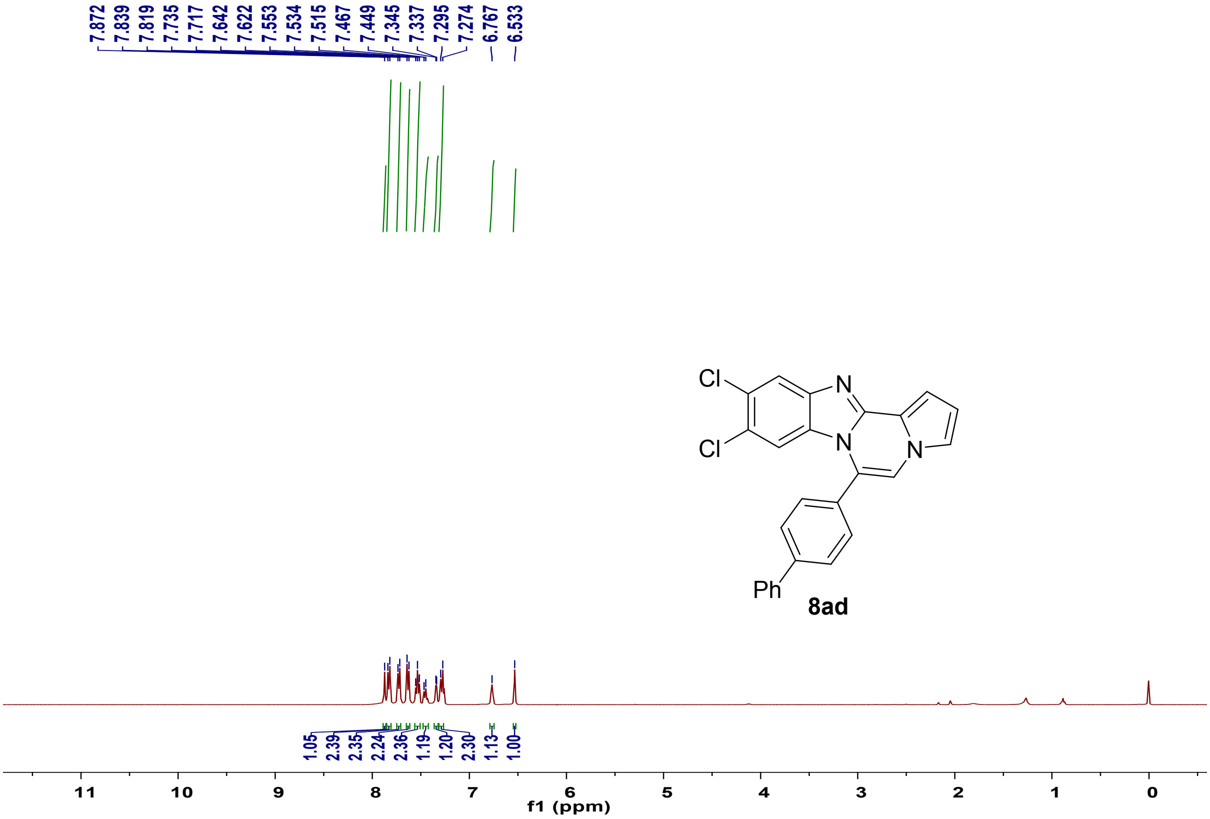
7.872
7.839
7.819
7.735
7.717
7.642
7.622
7.553
7.534
7.515
7.467
7.449
7.345
7.337
7.295
7.274
6.767
6.533
Cl
Cl
N
N
N
Ph
8ad
1.05
2.39
2.35
2.24
2.36
1.19
1.20
2.30
1.13
1.00
11 10 9 8 7 6 5 4 3 2 1 0
f1 (ppm)

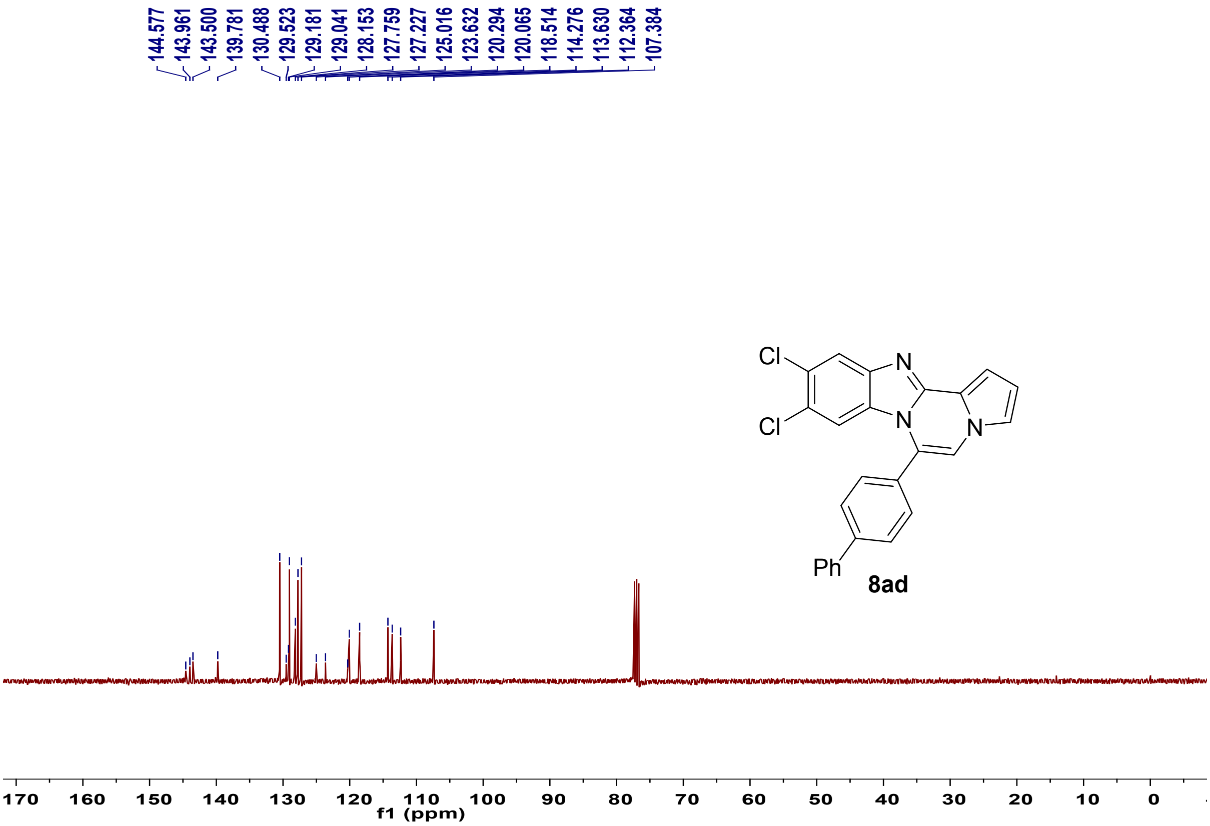
144.577
143.961
143.500
139.781
130.488
129.523
129.181
129.041
128.153
127.759
127.227
125.016
123.632
120.294
120.065
118.514
114.276
113.630
112.364
107.384
Cl
Cl
N
N
N
Ph
8ad
170 160 150 140 130 120 110 100 90 80 70 60 50 40 30 20 10 0
f1 (ppm)

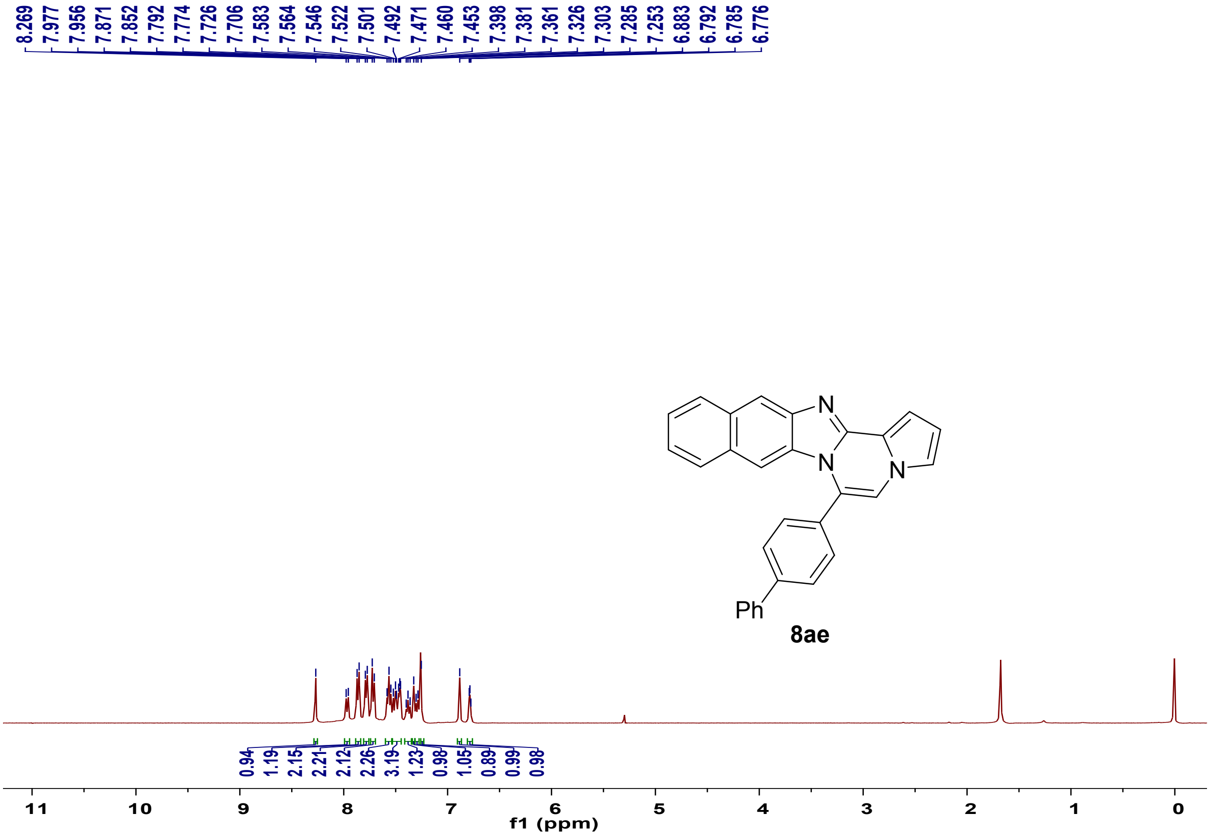
8.269 7.977 7.956 7.871 7.852 7.792 7.774 7.726 7.706 7.583 7.564 7.546 7.522 7.501 7.492 7.471 7.460 7.453 7.398 7.381 7.361 7.326 7.303 7.285 7.253 6.883 6.792 6.785 6.776
N
N
N
Ph
8ae
0.94 1.19 2.15 2.21 2.12 2.26 3.19 1.23 0.98 1.05 0.89 0.99 0.98
11 10 9 8 7 6 5 4 3 2 1 0
f1 (ppm)

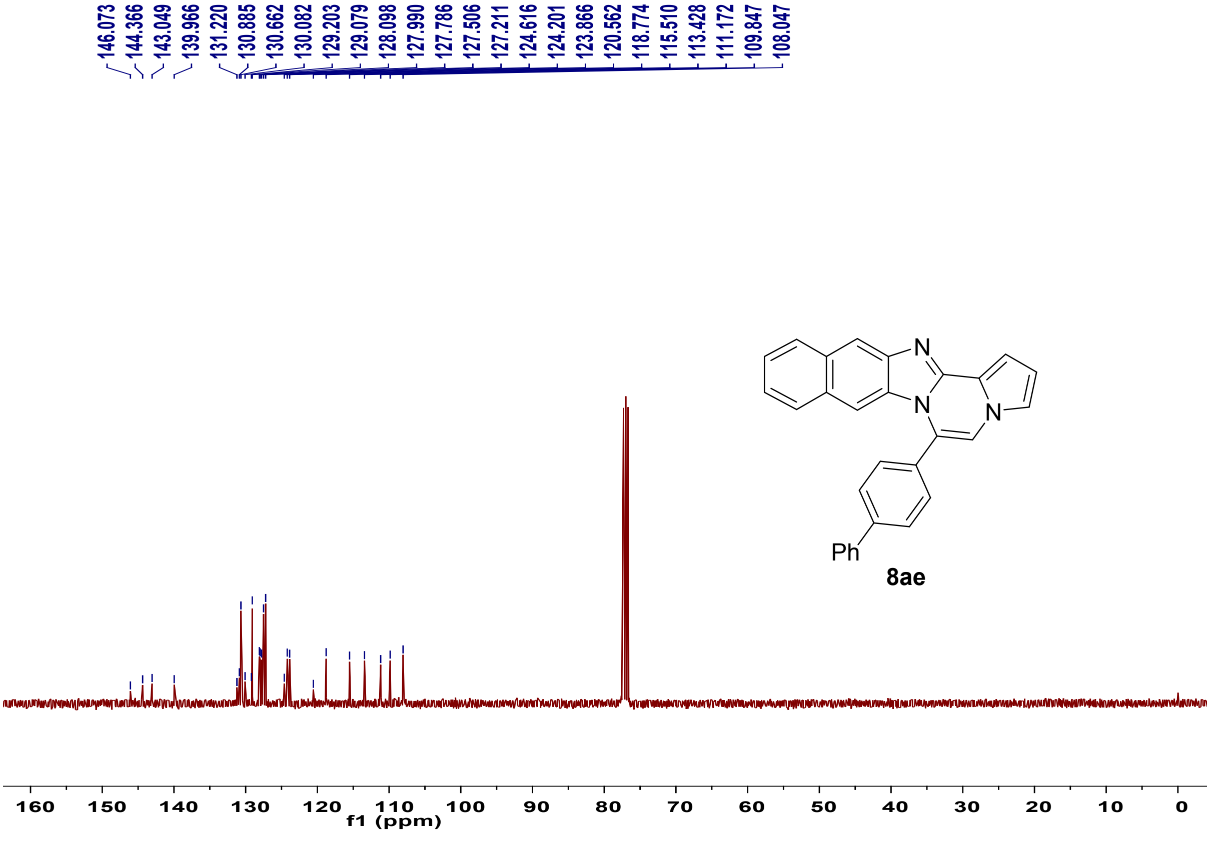
146.073 144.366 143.049 139.966 131.220 130.885 130.662 130.082 129.203 129.079 128.098 127.990 127.786 127.506 127.211 124.616 124.201 123.866 120.562 118.774 115.510 113.428 111.172 109.847 108.047
N
N
N
Ph
8ae
160 150 140 130 120 110 100 90 80 70 60 50 40 30 20 10 0
f1 (ppm)

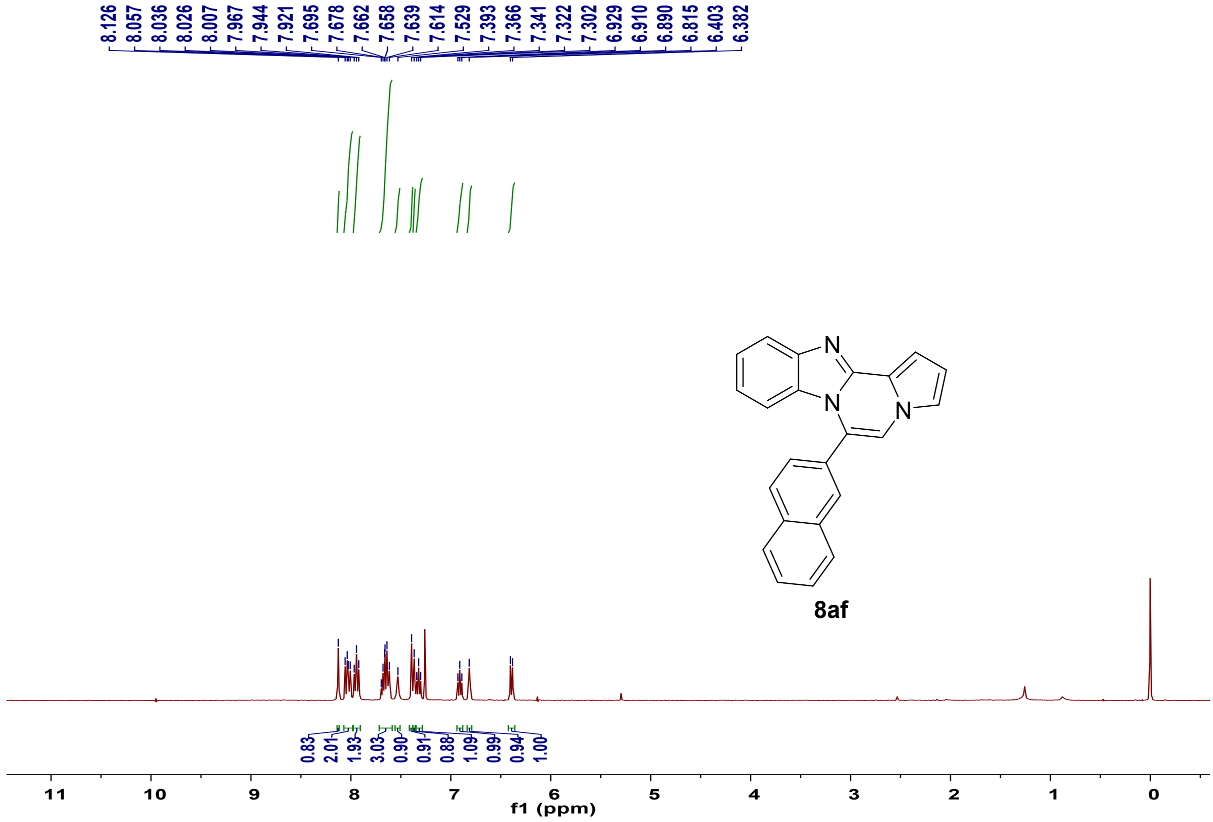
8.126
8.057
8.036
8.026
8.007
7.967
7.944
7.921
7.695
7.678
7.662
7.658
7.639
7.614
7.529
7.393
7.366
7.341
7.322
7.302
6.929
6.910
6.890
6.815
6.403
6.382
N
N
N
8af
0.83
2.01
1.93
3.03
0.90
0.91
0.88
1.09
0.99
0.94
1.00
11
10
9
8
7
6
5
4
3
2
1
0
f1 (ppm)

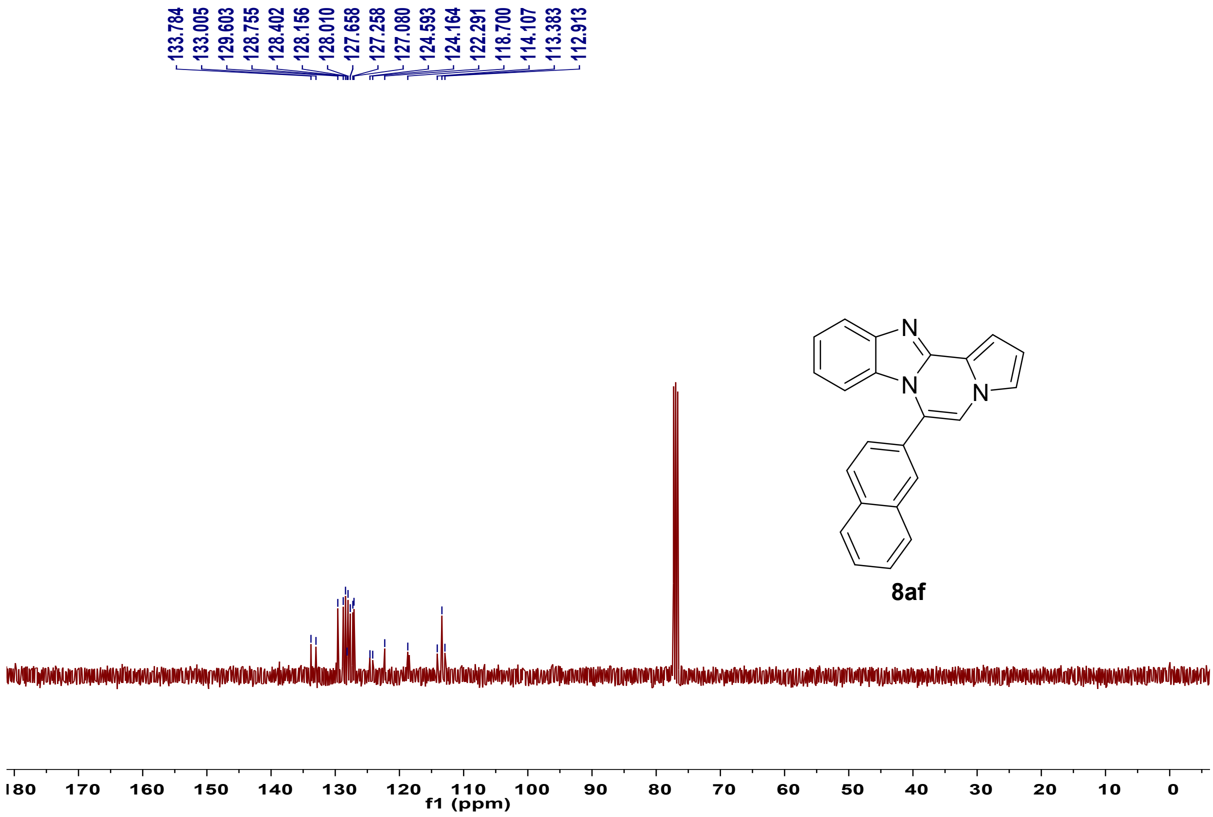
133.784
133.005
129.603
128.755
128.402
128.156
128.010
127.658
127.258
127.080
124.593
124.164
122.291
118.700
114.107
113.383
112.913
N
N
N
8af
180
170
160
150
140
130
120
110
100
90
80
70
60
50
40
30
20
10
0
f1 (ppm)

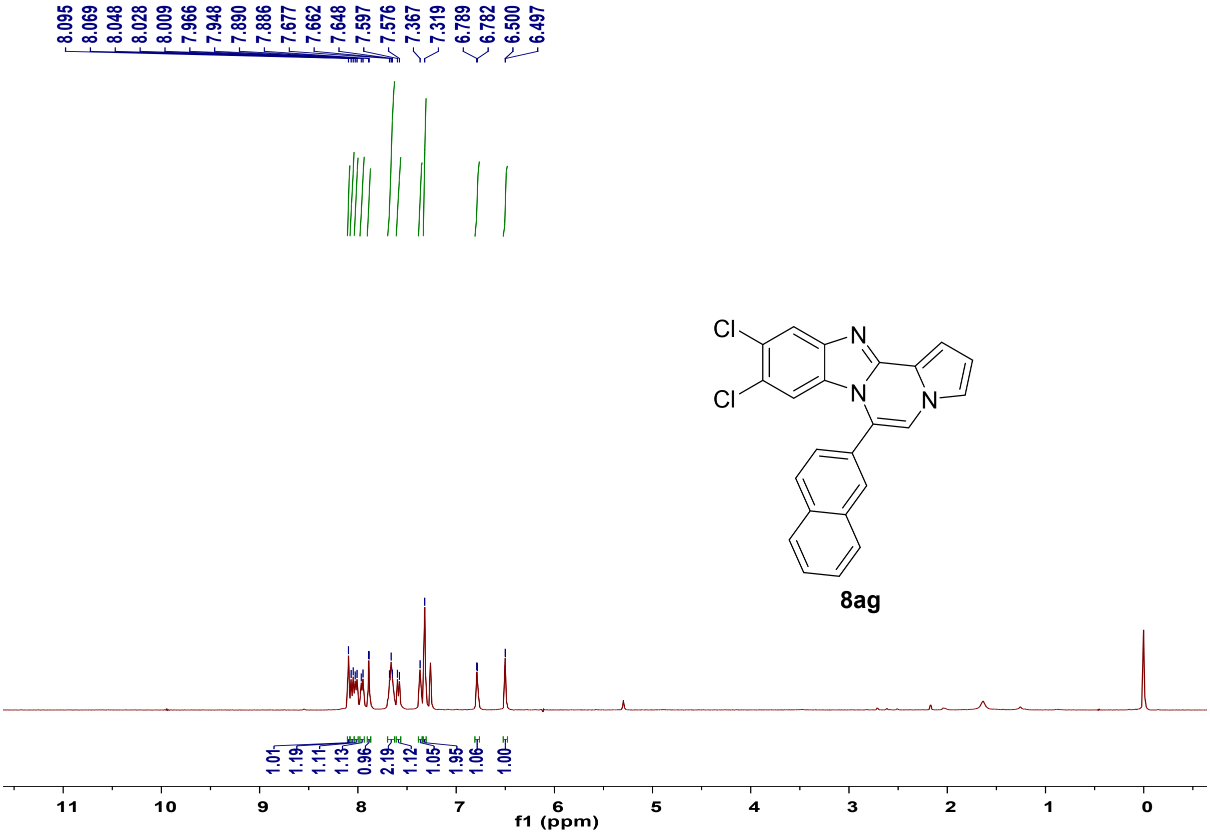
8.095
8.069
8.048
8.028
8.009
7.966
7.948
7.890
7.886
7.677
7.662
7.648
7.597
7.576
7.367
7.319
6.789
6.782
6.500
6.497
Cl
Cl
N
N
N
1.01
1.19
1.11
1.13
0.96
2.19
1.12
1.05
1.95
1.06
1.00
f1 (ppm)

8ag

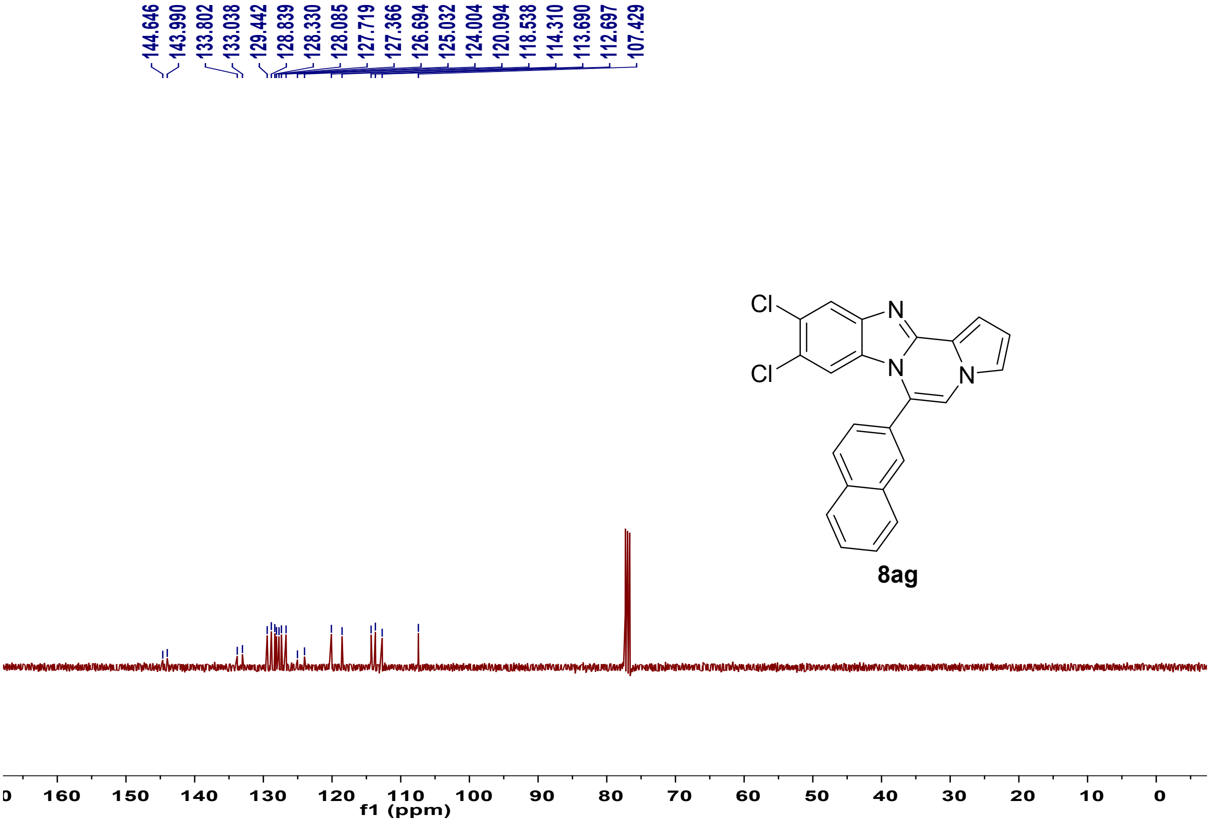
144.646
143.990
133.802
133.038
129.442
128.839
128.330
128.085
127.719
127.366
126.694
125.032
124.004
120.094
118.538
114.310
113.690
112.697
107.429
Cl
Cl
N
N
N
f1 (ppm)

8ag

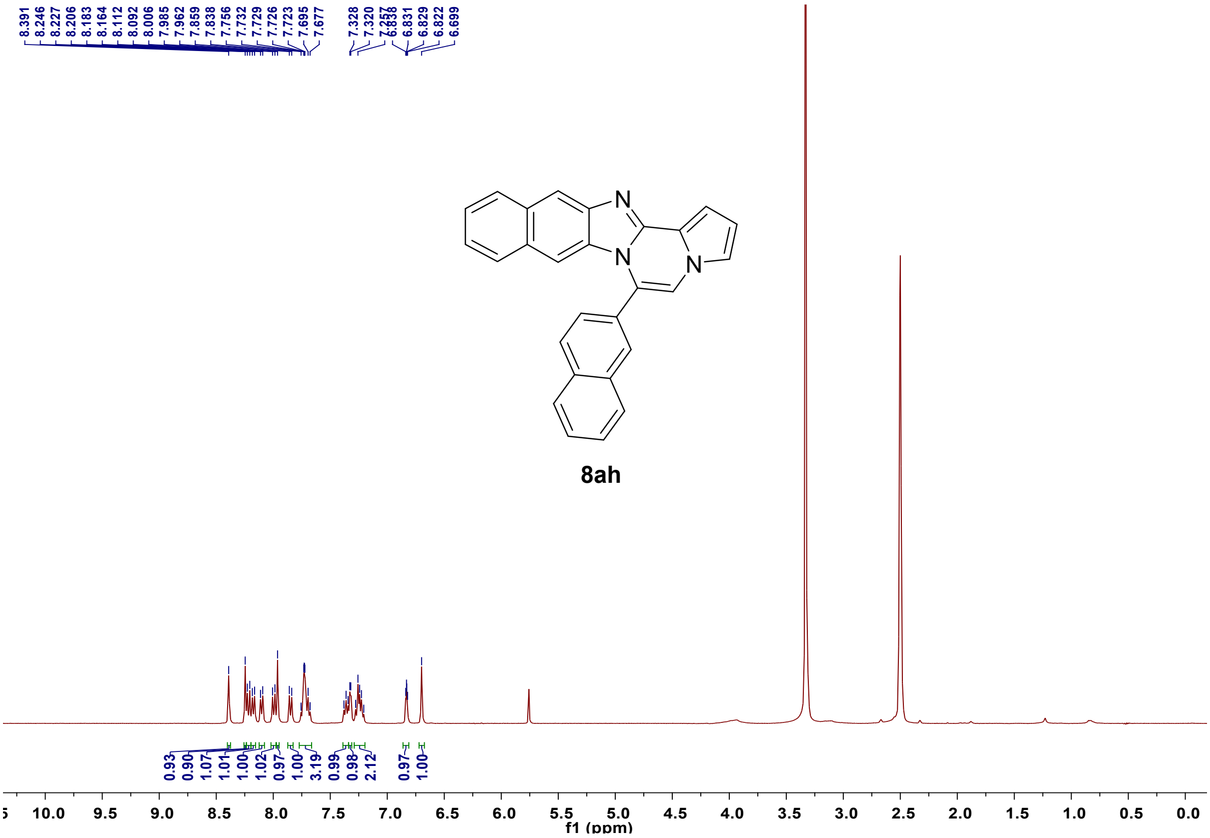
8.391
8.246
8.227
8.206
8.183
8.164
8.112
8.092
8.006
7.985
7.962
7.859
7.838
7.756
7.732
7.729
7.726
7.723
7.695
7.677
7.328
7.320
6.831
6.829
6.822
6.699
8ah
0.93
0.90
1.07
1.01
1.00
1.02
0.97
1.00
3.19
0.99
0.98
2.12
0.97
1.00
f1 (ppm)

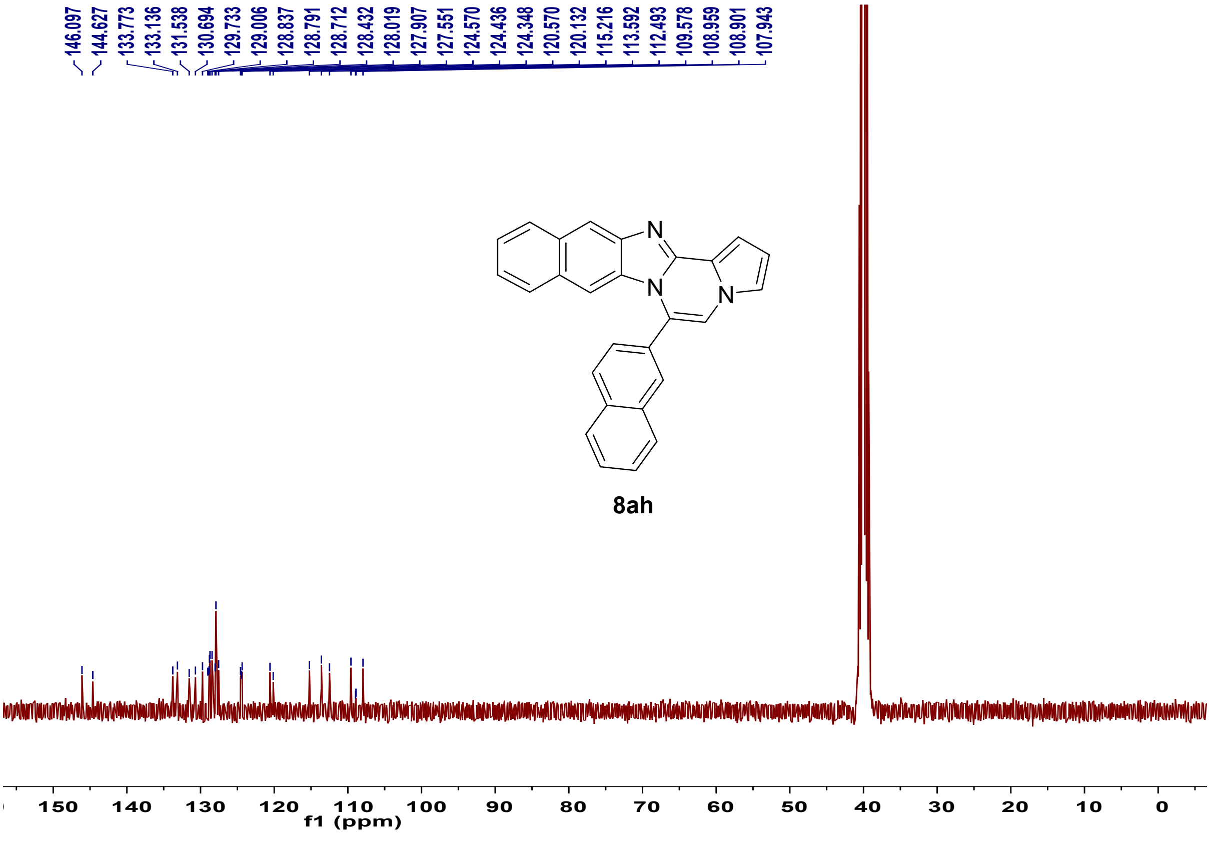
146.097
144.627
133.773
133.136
131.538
130.694
129.733
129.006
128.837
128.791
128.712
128.432
128.019
127.907
127.551
124.570
124.436
124.348
120.570
120.132
115.216
113.592
112.493
109.578
108.959
108.901
107.943
8ah
f1 (ppm)

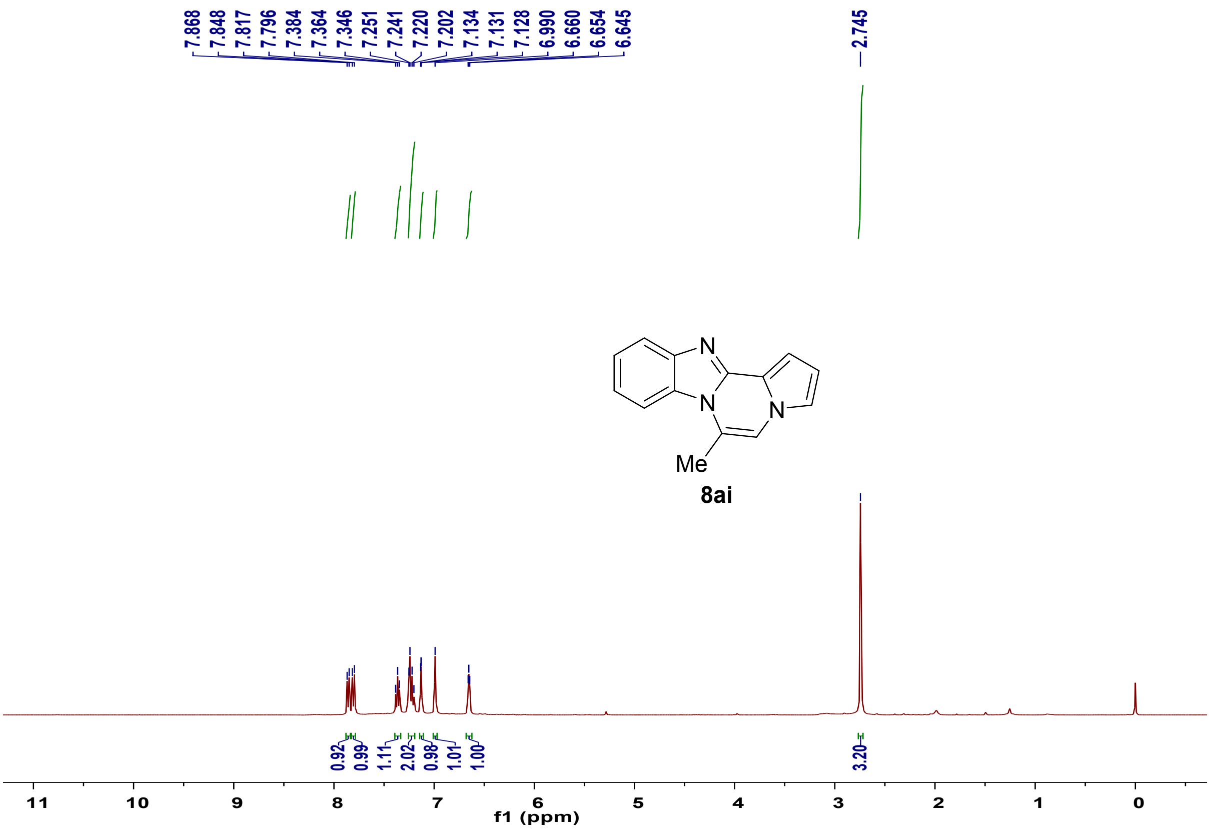
7.868
7.848
7.817
7.796
7.384
7.364
7.346
7.251
7.241
7.220
7.202
7.134
7.131
7.128
6.990
6.660
6.654
6.645
2.745
N
N
N
Me
8ai
0.92
0.99
1.11
2.02
0.98
1.01
1.00
3.20
11
10
9
8
7
6
5
4
3
2
1
0
f1 (ppm)

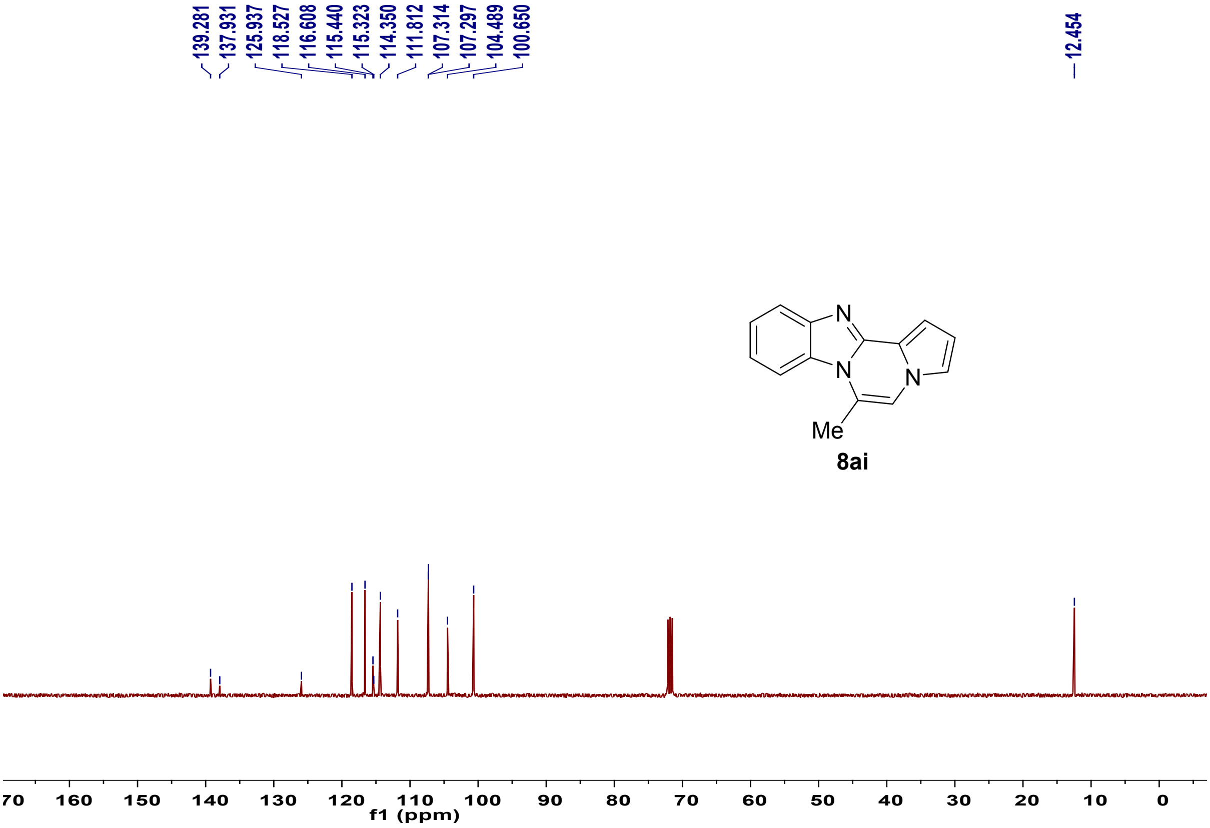
139.281
137.931
125.937
118.527
116.608
115.440
115.323
114.350
111.812
107.314
107.297
104.489
100.650
12.454
N
N
N
Me
8ai
70
160
150
140
130
120
110
100
90
80
70
60
50
40
30
20
10
0
f1 (ppm)

^{1}H and ^{13}C NMR spectra of 9

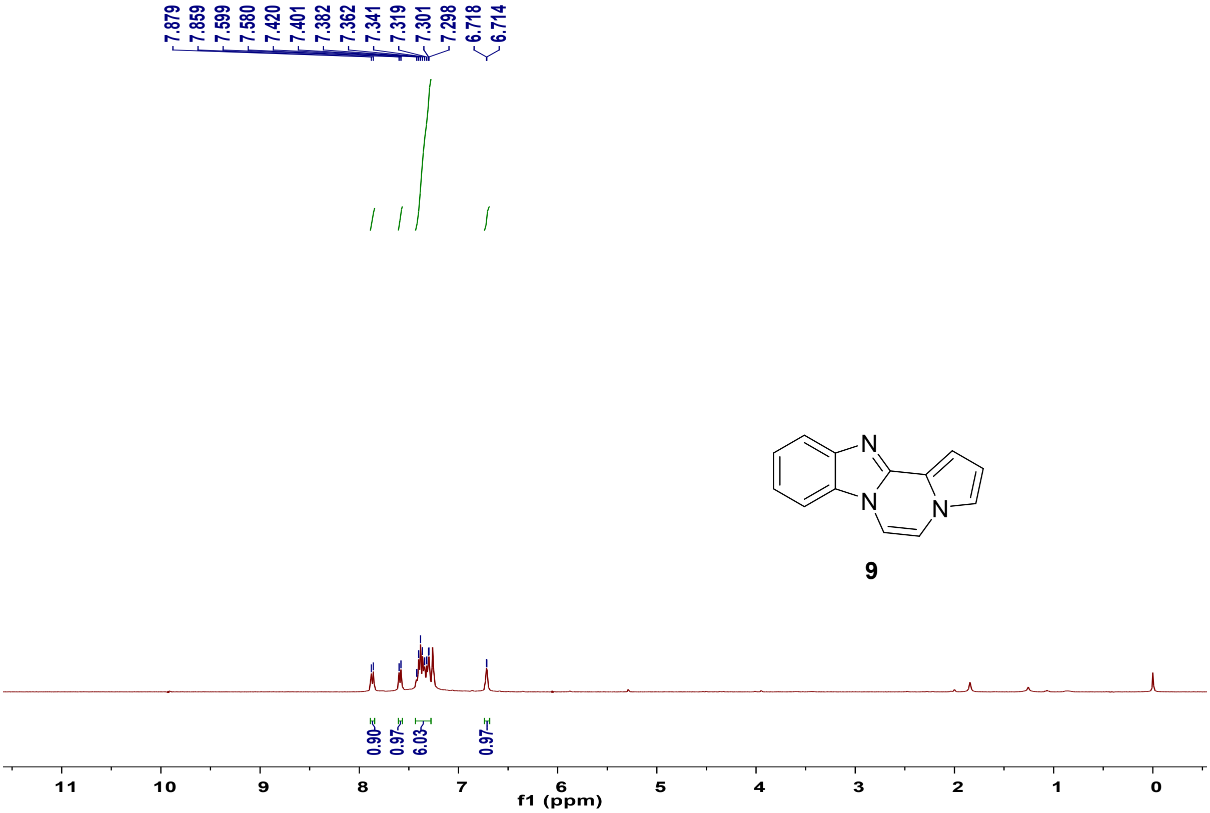

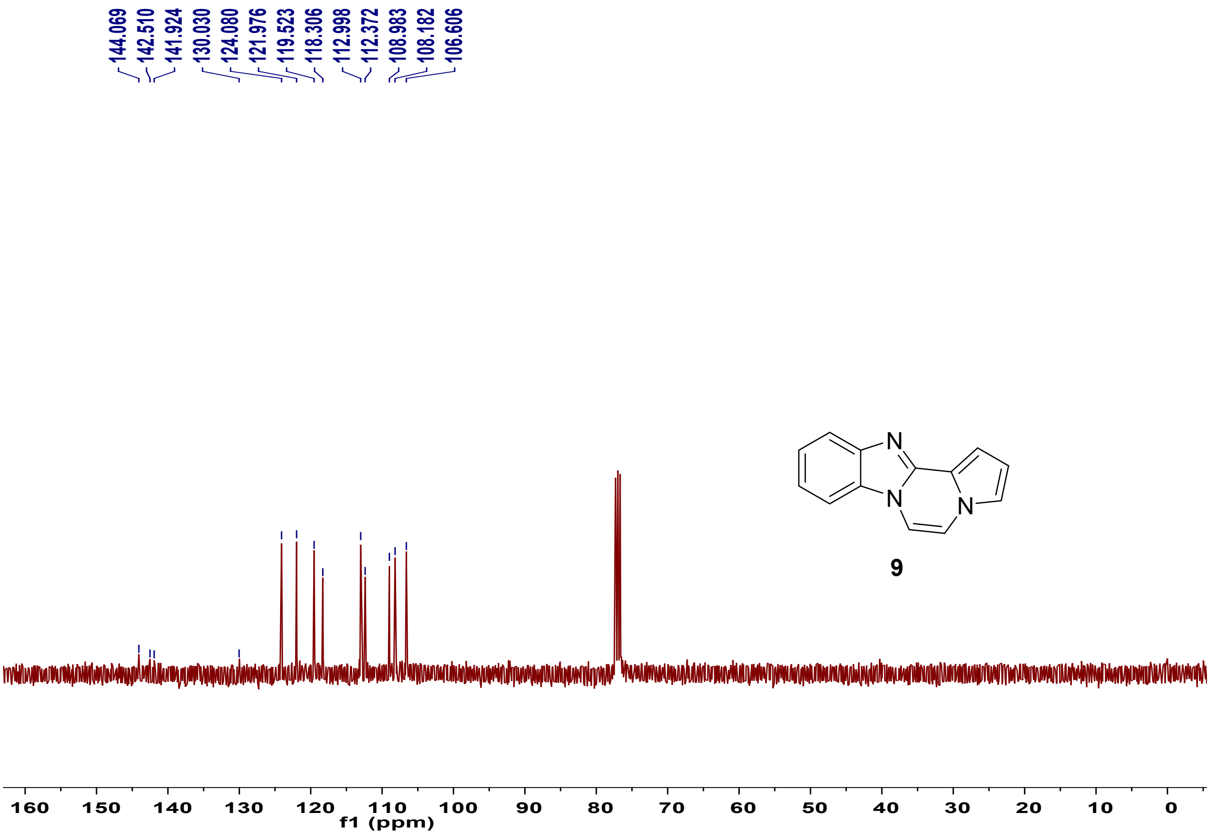

^{1}H and ^{13}C NMR spectra of 11

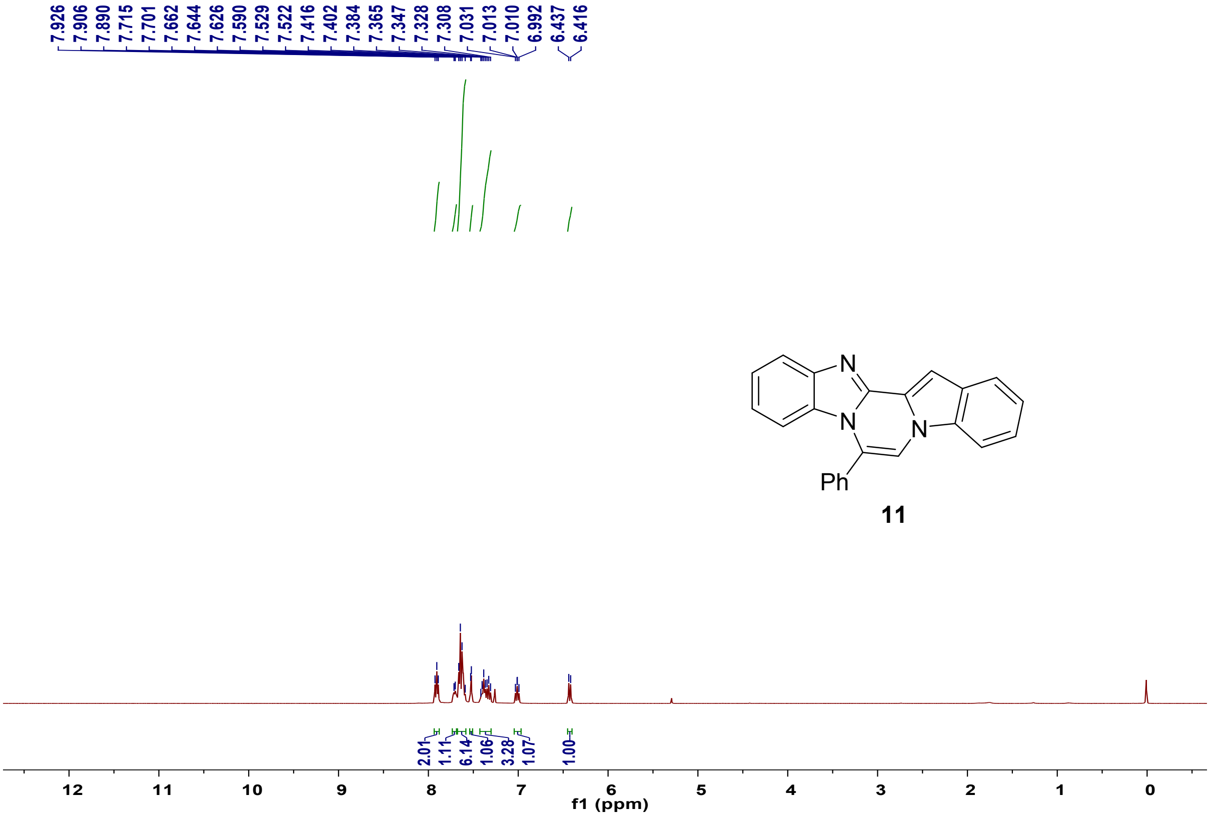

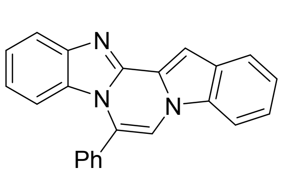

11

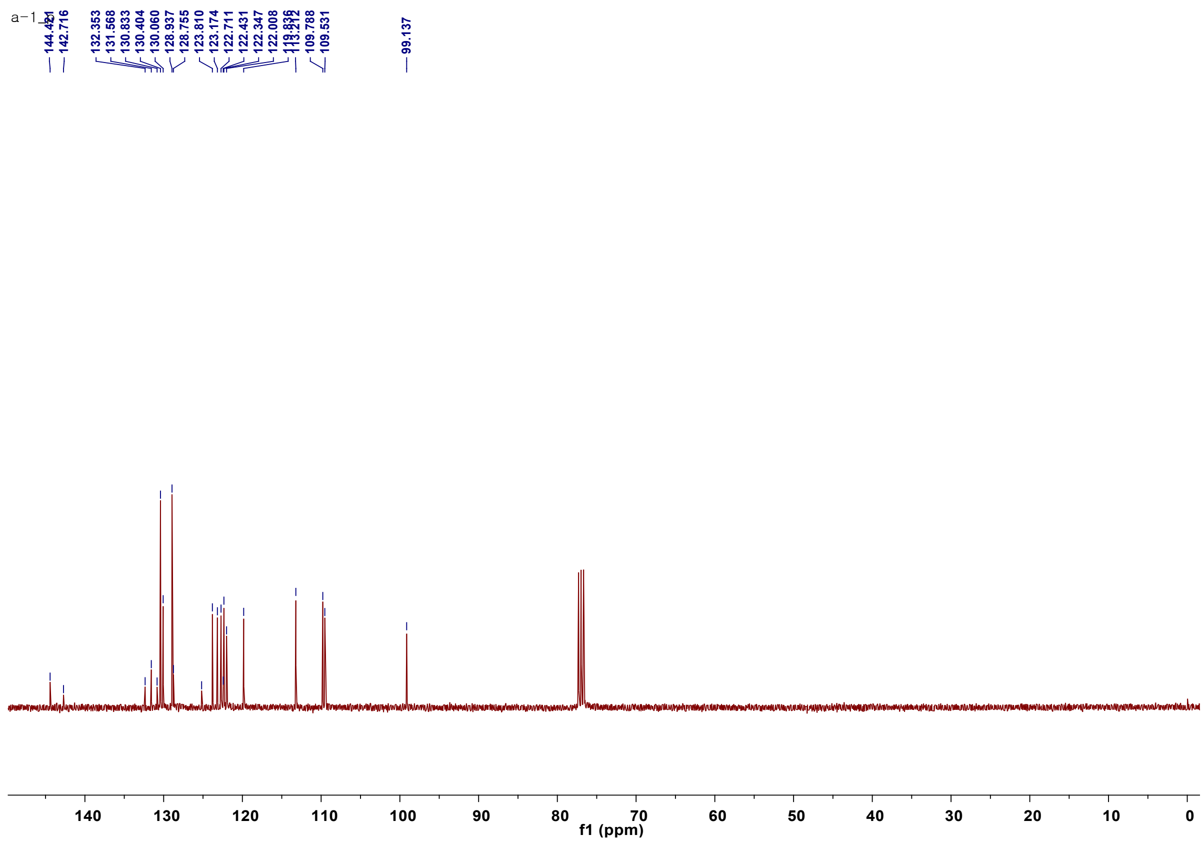

^{1}H and ^{13}C NMR spectra of 13

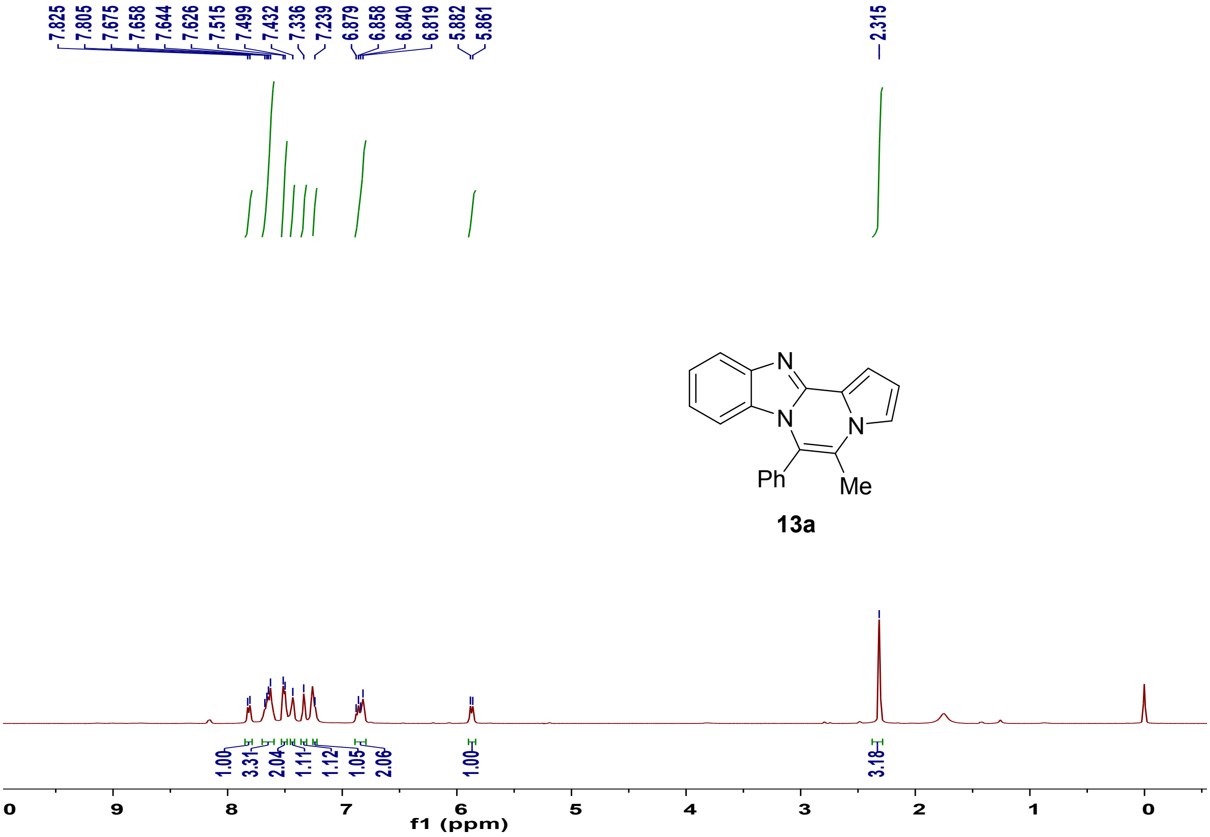

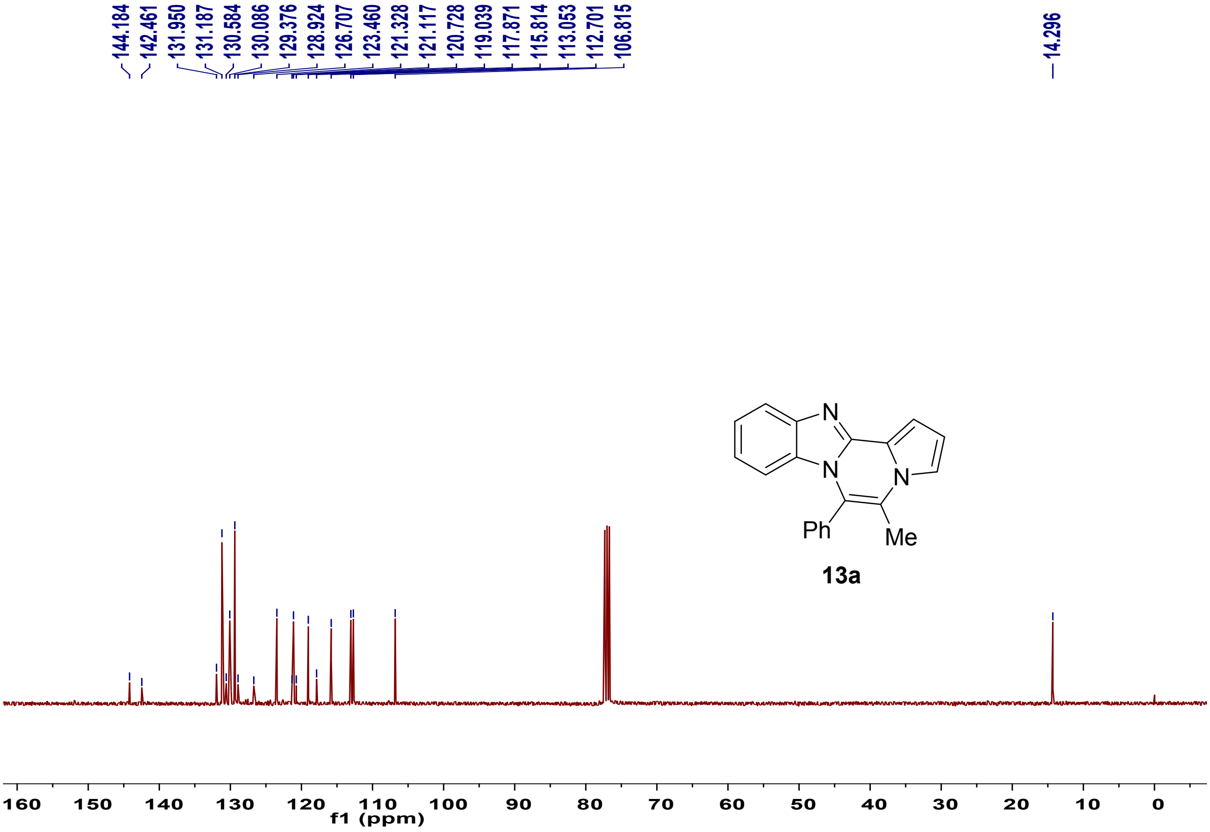
144.184
142.461
131.950
131.187
130.584
130.086
129.376
128.924
126.707
123.460
121.328
121.117
120.728
119.039
117.871
115.814
113.053
112.701
106.815
14.296
N
N
N
Ph
Me
13a
160 150 140 130 120 110 100 90 80 70 60 50 40 30 20 10 0
f1 (ppm)

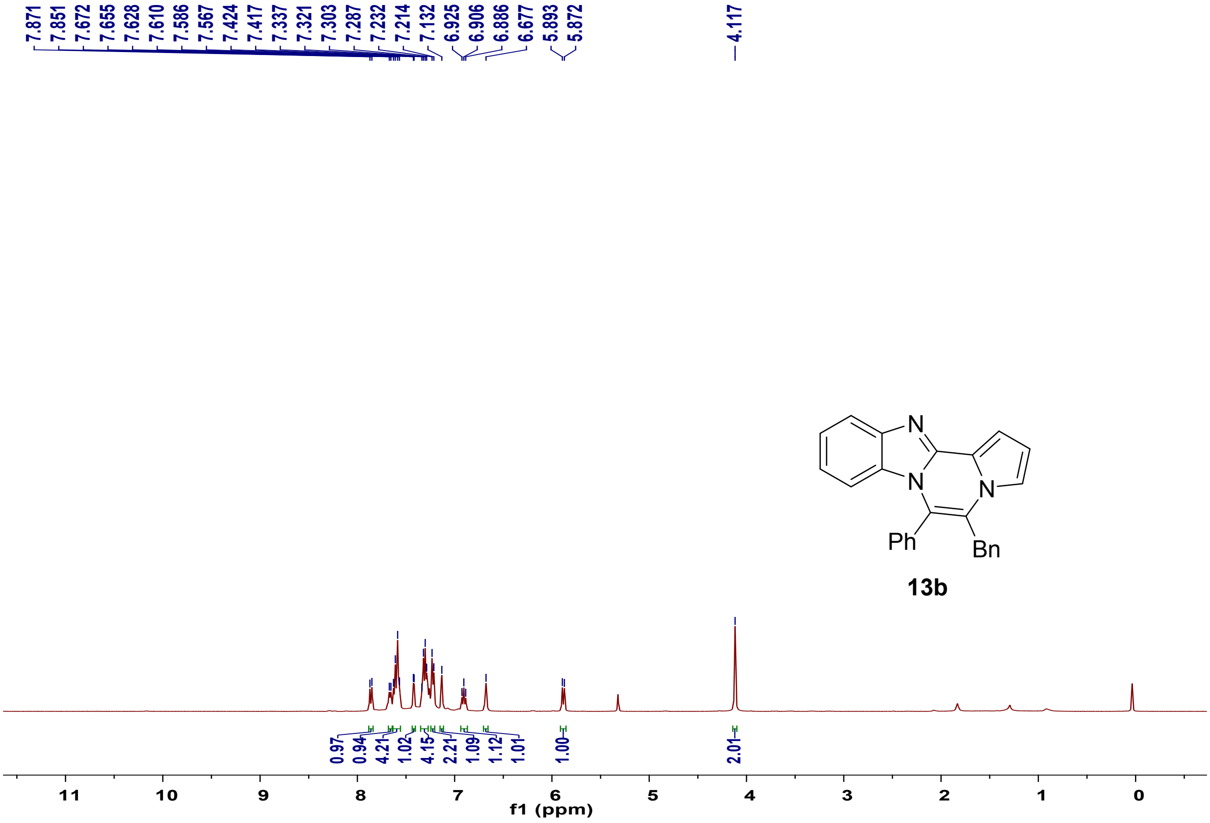
7.871
7.851
7.672
7.655
7.628
7.610
7.586
7.567
7.424
7.417
7.337
7.321
7.303
7.287
7.232
7.214
7.132
6.925
6.906
6.886
6.677
5.893
5.872
4.117
N
N
N
Ph
Bn
13b
0.97
0.94
4.21
1.02
4.15
2.21
1.09
1.12
1.01
1.00
2.01
11 10 9 8 7 6 5 4 3 2 1 0
f1 (ppm)

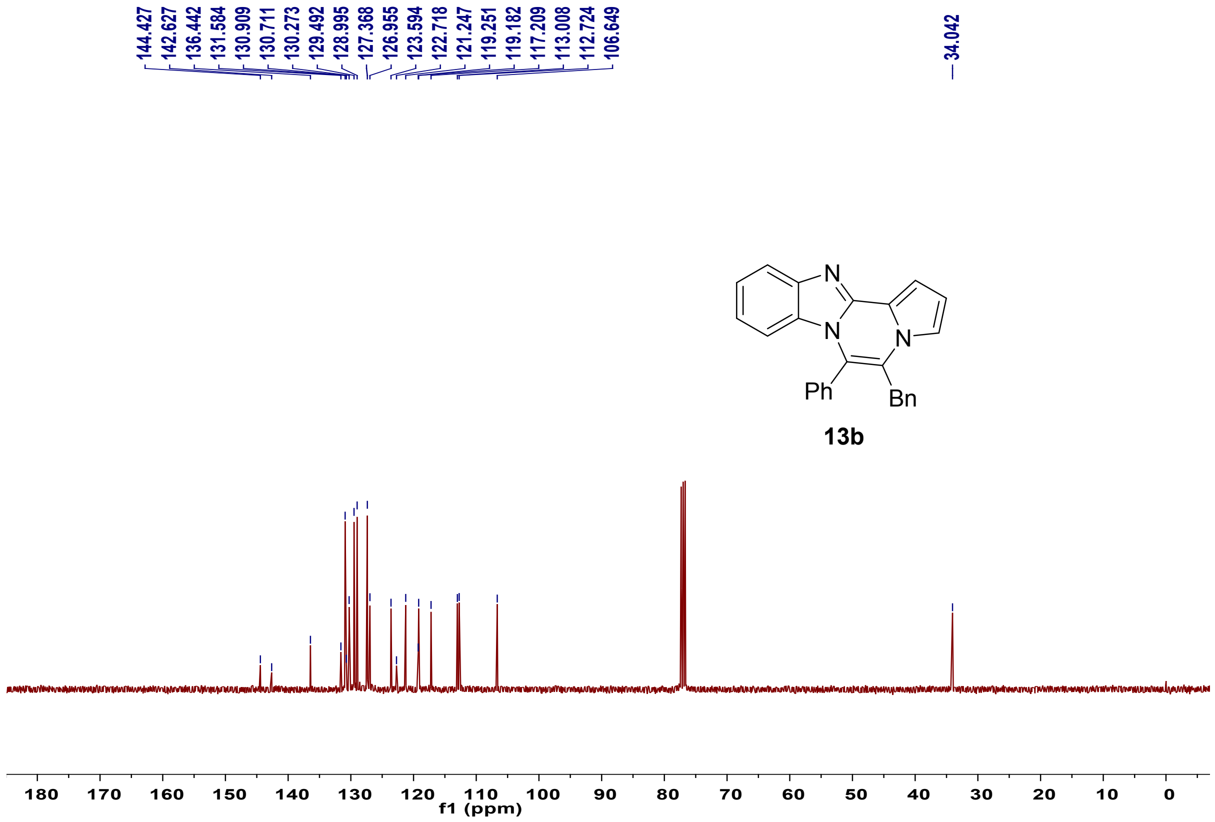

^{1}H and ^{13}C NMR spectra of 14

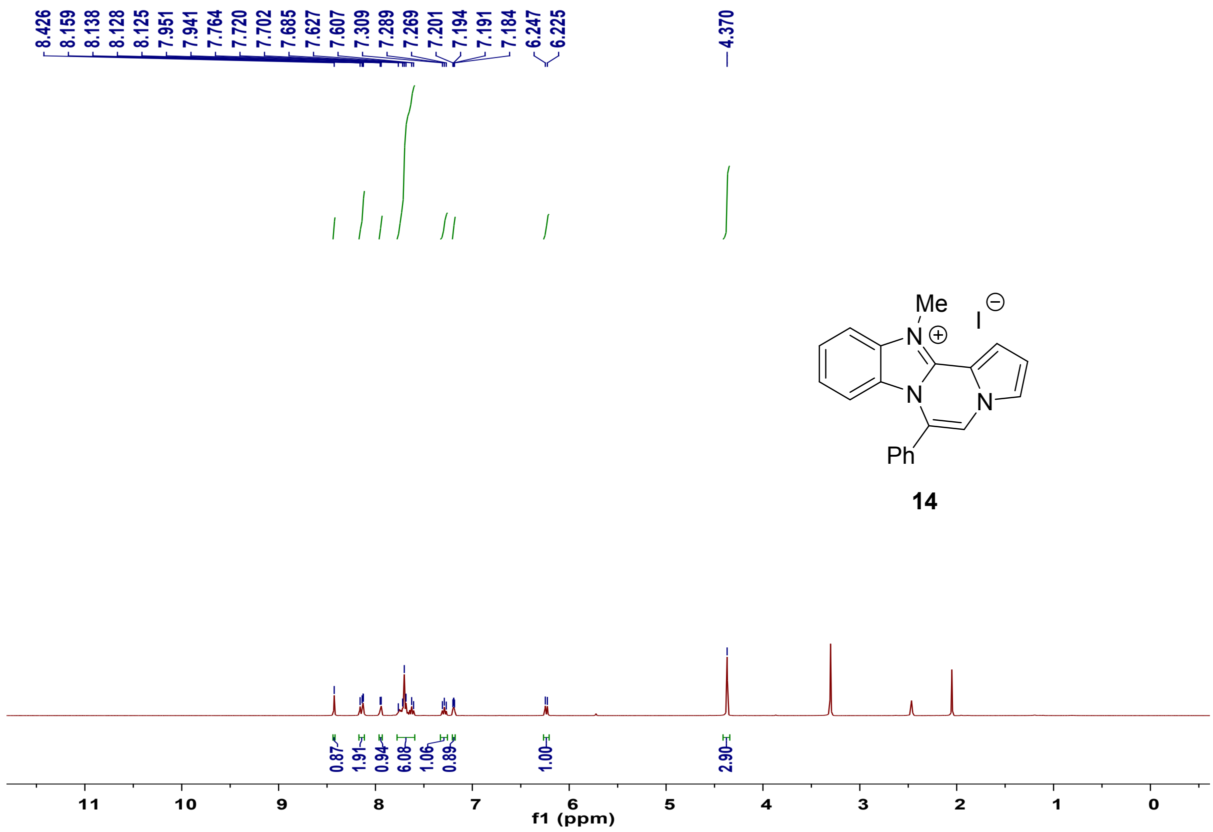

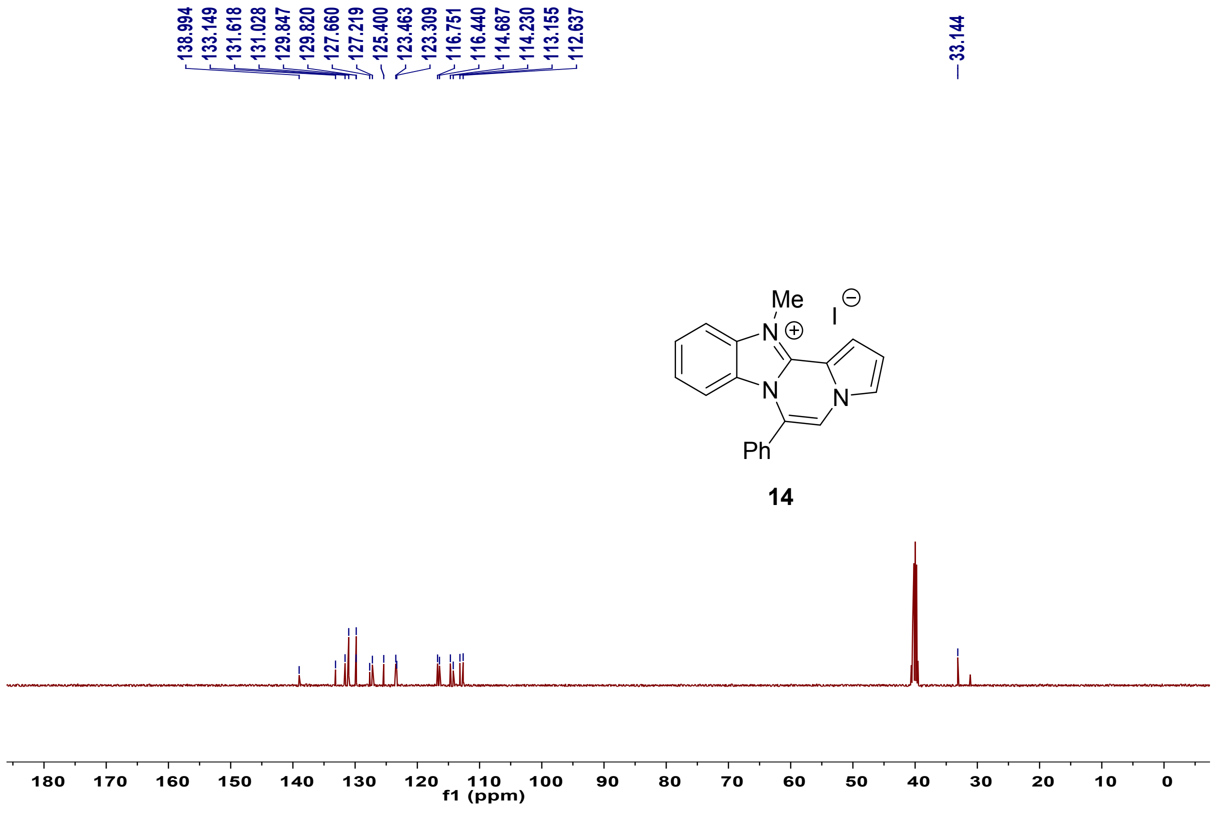

Optical characterization

The UV-vis absorption spectra and fluorescence emission spectra of the synthesized compounds were measured at ambient temperature with a Lambda25 (PerkinElmer, Waltham, MA, USA) and a FP-6500 spectrofluorometer (JASCO, Tokyo, Japan), respectively. Fluorescence spectra in solid state were measured with a slit width of 1 nm for both excitation and emission using a Fluoromax4 spectrofluorometer (Horiba Jobin Yvon, Kyoto, Japan). Fluorescence quantum yields (Φ_F) of the compounds in solution were obtained in ethanol by using anthracene (Φ = 0.27 in EtOH) as a reference compound.

Time-resolved fluorescence measurement.

Time-correlated single photon counting (TCSPC) technique with a DeltaTime on a FluoroMax-4 spectrofluorometer (Horiba Jobin Yvon, Kyoto, Japan) was used to measure fluorescence decays. The laser source for excitation was with a DeltaDiode (DD-470L, Horiba, Kyoto, Japan) for excitation wavelength of 464 nm. Fluorescence decay analysis software (DAS6 version 6.8, Horiba, Kyoto, Japan) was used for the analysis of the time domain fluorescence lifetime. Fluorescence decay components (τ) were reconvoluted with the instrumental response function (IRF) and fitted to the sum of exponential components. The weighted averages of the fluorescent decay components (τ_{avg}) were calculated using the following equation : $\tau_{avg} = \sum_{i=1}^{n} \alpha_i \tau_i$ (α = normalized pre-exponential values of each τ component).

Preparation and characterization of 8g nanoparticles.

8g nanoparticles were prepared using a sonication method by slow addition of stock solution to water as an anti-solvent. 1 mM stock solutions of **8g** were fully dissolved and prepared in anhydrous DMSO or THF. For the sonication method, 30 μL of **8g** stock solution was added

dropwise into the 3 mL of deionized water while sonicating. The suspended solution was sonicated for an additional 15 min at room temperature before measurement. Dynamic light scattering (DLS) was employed to characterize the particle size distribution of the **8g** nanoparticles (NP), using a ELSZ-2000 (Otsuka, chiyoda, Japan).

Image-based cell screening.

HeLa (human cervical cancer cell line) cells were seeded at $1.2x10^4$ cells per well in a Cellcarrier-96 black plate (PerkinElmer, Waltham, MA, United States) and incubated for 24 h at 37°C under 5% CO_2. After incubation, the cells were treated with the compounds for 1 h for live cell imaging and the screened images were acquired using an Operetta High-Content System (PerkinElmer, Waltham, MA, USA) with blue fluorescence 410-530 nm upon excitation at 360-400 nm with a 20x objective lens. The cell images were analysed using the Harmony software (PerkinElmer, Waltham, MA, USA).

Cytotoxicity Test.

MTT assay was used to evaluate the phototoxicity of the compounds. HeLa cells were used and 10000 cells were seeded per well in 96-well cell culture plates (SPL Life Science Co., Gyeonggi-do, Republic of Korea) then incubated for 24 h. After incubation, the cells were treated with compounds in different concentrations (0.5, 1, 2, 5, and 10 μM) and were incubated at 37°C under 5% CO_2 in the dark for 24 h. Then, the medium was replaced and 20 μM 3-(4,5-dimethylthiazol-2-yl)-2,5-diphenyltetrazolium bromide (MTT) (5 mg/mL) was added to each well. The cells were incubated for another 3 h and the medium was removed and DMSO (100 μL per well) was added to remove the formazan product. Mithras2 plate reader (Berthold

Technologies, Bad Wildbad, Germany) was used to evaluate cell viability by measuring the absorbance at 570 nm.

Single Crystal X-ray Diffraction Studies

Single crystals of **8c** were grown by the vapor diffusion method with a dimension of 0.407 × 0.281 × 0.247 mm^3 crystal size in monoclinic system. The suitable crystal was mounted on SuperNova, Dual, Cu at home/near and an AtlasS2 diffractometer (Agilent, Santa Clara, CA, United States). **8c** crystal data was collected using SuperNova dual source diffractometer operating with Cu Kα radiation (λ = 1.542 mm−1) at 292.2 K. The structure was solved by directs methods ShelXT software and refined by the least squares minimization using Olex2. CCDC 1919367 contains the supplementary crystallographic data for compound **8c**. These data can be obtained free of charge from The Cambridge Crystallographic Data Centre via www.ccdc.cam.ac.uk/data_request/cif. All copies of the data can be downloaded upon request to CCDC, 12 Union Road, Cambridge CB2 1EZ, U.K.

Supplementary data

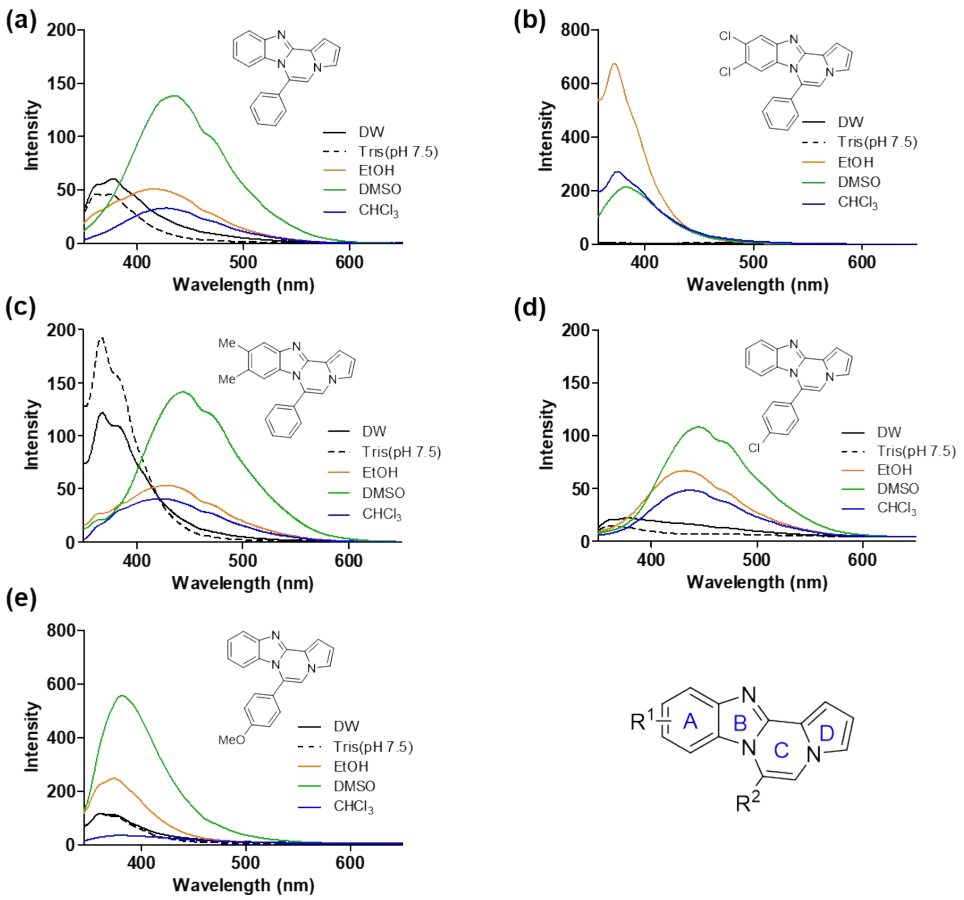

Figure S1. Fluorescence spectra of 2 μM **8a** (a), **8d** (b), **8g** (c), **8m** (d) and **8u** (e) measured in various solvents.

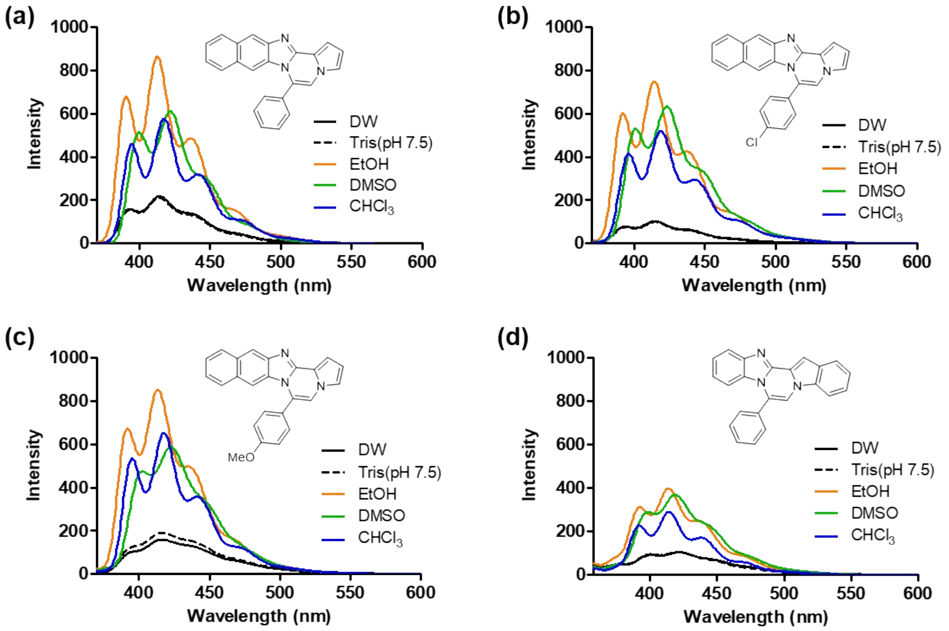

Figure S2. Fluorescence spectra of 1 μM **8h** (a), **8q** (b), **8z** (c) and **11** (d) measured in various solvents.

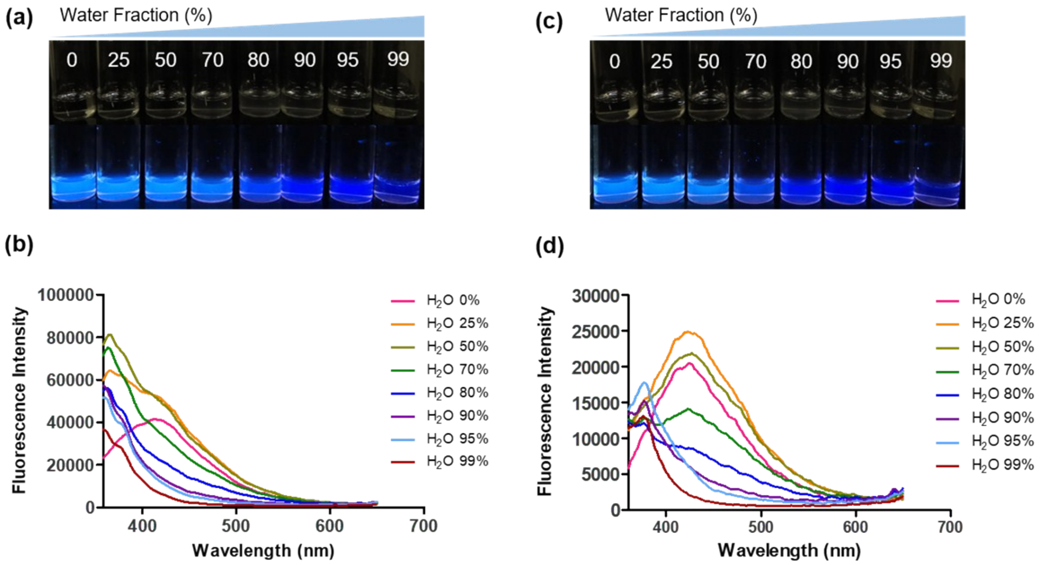

Figure S3. Photographs of 10 μM **8c** (a) and **8i** (c) in THF/Water mixture (0-99%) under UV light (λ_{ex}= 312 nm). Fluorescence spectra of 10 uM **8c** (b), and **8i** (d) in THF/Water mixture (0-99%) excited at 338 nm and 335 nm, respectively.

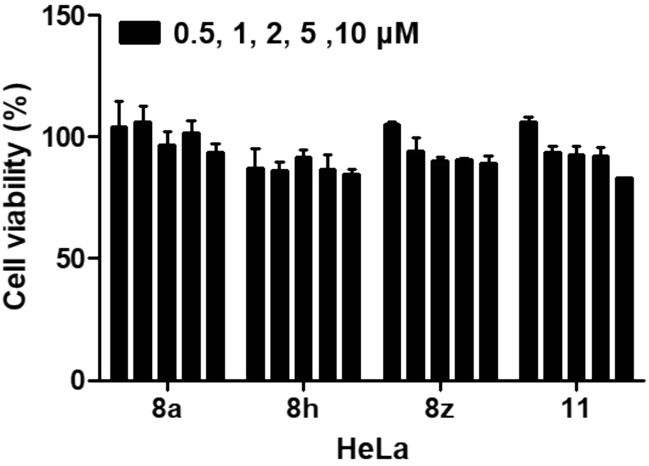

Figure S4. Cytotoxicity of **8a**, **8h**, **8z**, and **11** in HeLa cells incubated for 24 h in the dark and measured using the MTT assay.

Compound	λ_{abs} (nm)	λ_{em} (nm)			ε_{max} ($M^{-1}cm^{-1}$)
		H_2O	EtOH	DMSO	
**8a**	335	376.5	415.5	435.0	22900
**8b**	348	-	372.5	377.5	19800
**8c**	338	362.0	363.0	402.0	29500
**8d**	347	483.0	372.0	382.0	20800
**8e**	340	367.0	364.5	380.0	30500
**8f**	339	363.5	365.5	393.5	39600
**8g**	339	367.0	425.0	443.5	29200
**8i**	335	359.5	357.5	427.5	20200
**8j**	347	501.0	371.5	381.5	23000
**8m**	335	376.5	431.5	445.0	22500
**8n**	339	363.0	419.5	438.0	22600
**8o**	347	369.5	375.0	388.0	24300
**8p**	339	385.0	419.5	451.0	24000
**8q**	361	415.0	414.0	423.0	21300
**8r**	335	359.0	429.5	442.0	20300
**8s**	348	520.0	376.5	390.5	28500
**8t**	361	415.0	414.5	423.5	23000
**8u**	335	360.5	374.0	381.5	25300
**8v**	339	364.5	377.0	393.5	28900
**8w**	347	500.0	388.5	406.5	31500
**8x**	348	529.0	386.6	376.0	30700
**8y**	340	367.5	364.0	411.5	28800
**8aa**	335	359.0	375.5	390.5	25700
**8ac**	335	388.0	372.5	396.0	25400
**8ad**	347	522.5	388.0	402.5	30300
**8af**	335	479.0	433.0	446.0	18900
**8ag**	348	529.0	388.0	403.5	30700
**8ai**	334	360.0	355.0	358.0	13000
**13a**	336	361.0	357.5	366.0	26600
**13b**	336	362.5	358.5	361.0	22100
**14**	339	420.0	418.5	421.0	22900

Table S1. Optical properties of 4BP and 5BP scaffolds.

Single crystal x-ray crystallographic data

	8c
molecular formula	$C_{19}H_{12}FN_3$
formula weight	301.32
crystal system	monoclinic
space group	$P2_1/n$
a [Å]	10.5685(6)
b [Å]	8.4986(6)
c [Å]	16.7749(14)
α [°]	90
β [°]	96.930(6)
γ [°]	90
V [Å^3]	1495.67(19)
Z	4
ρ_{calcd} [g cm^{-3}]	1.338
μ [mm^{-1}]	0.090
F(000)	624.0
reflns measured	17305
unique reflns (R_{int})	3729 (0.0568)
R_1 [$I > 2\sigma(I)$]	0.0571
wR_2 (all data)	0.1534
Goodness of fit	1.030

Table S2. Crystallographic data of **8c**.
